# Spiro Indane-Based Phosphine-Oxazolines as Highly Efficient P,N Ligands for Enantioselective Pd-Catalyzed Allylic Alkylation of Indoles and Allylic Etherification

**DOI:** 10.3390/molecules24081575

**Published:** 2019-04-21

**Authors:** Zhongxuan Qiu, Rui Sun, Kun Yang, Dawei Teng

**Affiliations:** State Key laboratory Base of Eco-chemical Engineering, College of Chemical Engineering, Qingdao University of Science and Technology, Qingdao 266042, China; qiuzhognxuan21@163.com (Z.Q.); sunruiqdao@163.com (R.S.); yang802597641kun@163.com (K.Y.)

**Keywords:** asymmetric synthesis, indole, phosphine-oxazoline, spiro, palladium

## Abstract

A series of indane-based phosphine-oxazoline ligands with a spirocarbon stereogenic center were examined for palladium-catalyzed asymmetric allylic alkylation of indoles. Under optimized conditions, high yields (up to 98%) and enantioselectivities (up to 98% ee) were obtained with a broad scope of indole derivatives. The ligand was determined to be the most efficient P,N-ligand for this reaction. Moreover, the ligand was also efficient for Pd-catalyzed asymmetric allylic etherification with hard aliphatic alcohols as nucleophiles.

## 1. Introduction

**Figure 1:**
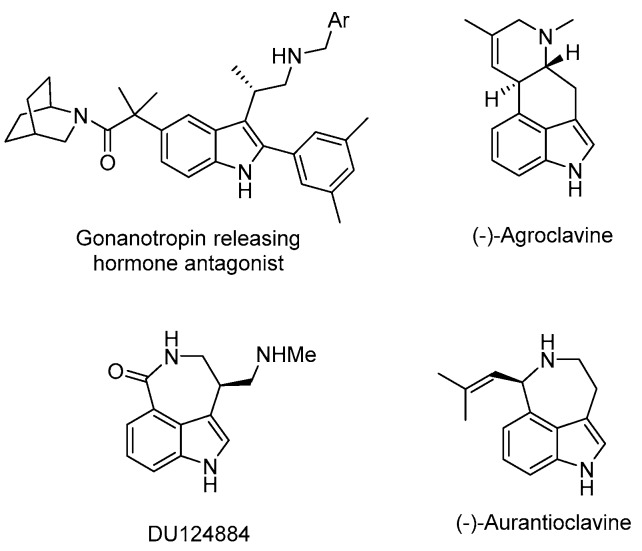
Selected natural products containing the indole moieties.

As a typical heterocyclic structural subunit, the indole scaffold is highly prevalent in biological compounds, pharmaceuticals, natural products and material science [1,2,3,4,5]. Notably, a variety of biologically active indole derivatives and indole-based drugs are enantiopure. Therefore, the synthesis and functionalization of chiral indole derivatives is meaningful. It is found that many 3-substitued indoles are important intermediates of natural products, such as (−)-agroclavine and (−)-aurantioclavine (Figure 1). Because of the biologically significant activities, including treatment of migraine headaches, antagonists, antiviral activity and agonist, functionalization of indoles on C-3 position has attracted much attention [6]. Enantioselective alkylation of indoles at the C-3 position by the Friedel–Crafts reaction with Lewis acid as the catalyst has been explored extensively [7,8,9,10]. However, high catalyst loading (10 %mol) is usually needed to obtain satisfactory yields and enantioselectivites. 

Recently, palladium-catalyzed allylic alkylation has been proven to be an attractive strategy to achieve enantiopure 3-substituted indoles [11,12,13,14] and diligent efforts have been made to develop novel and efficient chiral palladium-complexes using the asymmetric allylic alkylation of indoles as the model reaction. Chan reported that chiral ferrocenyl P/S ligands demonstrated good catalytic performance [15]. Hoshi reported that high yields and good enantioselectivities were obtained with sulphur-MOP ligands [16]. A phosphine ligand developed by Mino afforded 89% yields and 80% enatioselectivities with narrow substrate scope [17]. Other ligands such as phosphoramidite-thioether [18], phosphoramidite-terminal olefin [19], phosphine olefin [20,21], and helicenylphosphine ligands [22] were also reported as efficient catalysts.

The P,N-ligands are an important class of ligands and have successfully been applied in a large scope of reactions because the steric and electronic characters of ligands can be finely tuned [23,24]. However, for the asymmetric allylic alkylation of indole, P,N ligands were not as effective as other ligands mentioned above. The reaction only afforded < 10% ee using *^i^Pr*-phosphine-oxazoline (*^i^Pr*-PHOX) as the ligand [15]. Hence, design of highly active and selective P/N ligands is still desirable. 

**Scheme 1:**
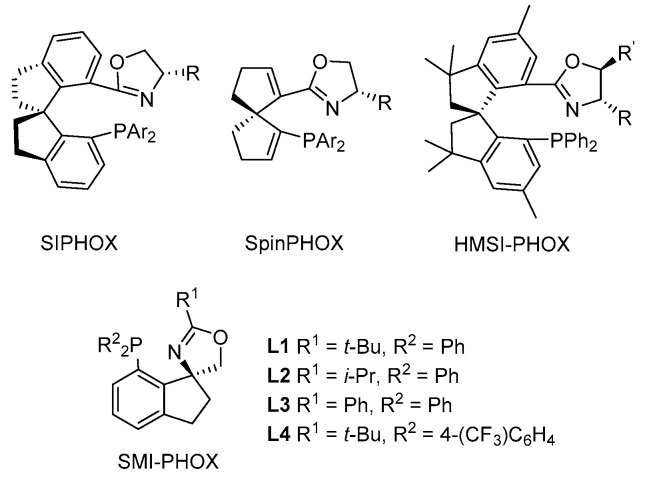
Spiro-backbone-based phosphine-oxazoline ligands.

Spiro phosphine-oxazoline ligands exhibit impressive catalytic performance in many cases by taking advantage of the chelating units of PHOX (phosphine-oxazoline ligands) and the spiro backbone. So far, there are four kinds of spiro phosphine-oxazoline ligands reported, including SIPHOX (spirobiindane-based phosphine-oxzoline ligands) by Zhou [25,26,27,28,29,30], SpinPHOX (spiro[4.4]-1,6-nonadiene-based phosphine-oxazoline ligands) by Ding [31,32,33,34], HMSI-PHOX (hexamethyl-1,1’-spirobiindane phosphine-oxazoline ligands) by Lin [35] and SMI-PHOX (an abbreviation for spiro mono-indane-based phosphine-oxazoline ligands **L1**–**L4** in Scheme 1) by us [36,37]. Compared with the other three ligands, SMI-PHOX ligands possess potentially distinct features: (i) first spiro indane-based phosphine-oxazoline ligand with non-*C_2_*-symmetric skeleton in asymmetric metal catalysis; (ii) better stability and higher rigidity; (iii) only one chiral center avoiding the complex stereochemistry; (iv) modularity by changing easily accessible carboxylic acid and ClPR^2^_2_ [36]. Because of the aforementioned properties, SMI-PHOX ligands have demonstrated excellent catalytic performance in several asymmetic reactions [36,37]. Therefore, we envisaged that they would be efficient chiral ligands for an indole C3-allylic alkylation reaction. As a result, high catalytic activities and enantioselectivities were obtained when these spiro phosphine-oxazoline ligands were used. We here disclose a detailed account of the Pd-catalyzed asymmetric allylic alkylation of indoles using a series of highly rigid spiro phosphine-oxazolines as chiral ligands.

## 2. Results and Discussion

The chiral phosphine-oxazoline ligands **L1**–**L4** were synthesized according to the literature [36]. We investigated the ability of four representative spiro phosphine-oxazoline ligands **L1**–**L4** for palladium-catalyzed asymmetric allylic alkylation of indole. The reaction of indole (**1a**) with 1,3-diphenyl-2-propenyl acetate (**2a**) as the model reaction was examined in the presence of [Pd(C_3_H_5_)Cl]_2_ (2.0 mol%) and ligand (4.0 mol%, Pd/ligand = 1/1) in toluene at room temperature using Cs_2_CO_3_ (2 equivalent) as the base. The results were summarized in Table 1. The reactions gave the corresponding product **3a** in high yields (87%–95%) and enantioselectivities (87–96% ee) with the use of ligands **L1**, **L2** and **L4** (entry 1, 2, 4). However, yield and enantioselectivity slightly decreased in the presence of ligand **L3** with a phenyl substitution on the oxazoline ring (entry 3). Taking into account the catalytic activity and enantioselectivity, ligand **L1** was chosen as the optimal ligand for further intensive study.

The reaction was investigated under various conditions using the ligand **L1**. The results are summarized in Table 2. It was shown that solvents have a great influence on the reaction activity and enantioselectivity (entries 1–8). High yield (95%) and excellent enantioselectivity (96% ee) were obtained with toluene as the solvent (entry 1). When the reaction was carried out in xylenes and mesitylene, the enantioselectivities slightly decreased (entries 2 and 3). With dichloromethane and ethyl acetate as solvents, the entioselectivities decreased to around 80% ee (entries 4 and 5). Using acetonitrile, tetrahydrofuran or *N,N*-dimethylformamide as the solvents, low yields and entioselectivities were obtained (entries 6–8). Toluene was proved to be the best choice. We next changed the base for the reaction in toluene besides of Cs_2_CO_3_ (entries 9 and 10). Using K_2_CO_3_ as the base, product **3a** was obtained with a slight decrease in enantioselectivity (89% ee) (entry 9). But the yield of **3a** decreased to 65%. With Na_2_CO_3_, the enantioselectivity of the reaction dramatically decreased to 15% ee (entry 10). Subsequent optimization of temperature indicated that the reaction afforded lower yield of **3a** at 0 °C and lower enantioselectivity at 40 °C than that at room temperature (entries 11 and 12). Compared to the results shown in entry 1, a slight decrease of yields and enantioselectivities was observed by changing the ratio of indole to 1,3-diphenyl-2-propenyl acetate from 1:1.2 to 1.2:1 (entries 13 and 14). Accordingly, the optimal reaction conditions were established as follows: 2 mol% [Pd(C_3_H_5_)Cl]_2_, 4 mol% of ligand **L1**, 2.0 equivalents of Cs_2_CO_3_ in toluene at room temperature with the molar ratio of **1a** to **2a** being 1:1.2.

With the optimized conditions in hand, we examined the substrate scope with various indoles **1a**–**1o** and allylic acetates **2a**–2**e**. As shown in Table 3, the reaction exhibited high tolerance of various substituted indoles. All reactions gave high yields and enantioselectivities either with substituents in the C2 position or in the phenyl ring. With 2-substituted indoles **1b**–**1c**, the reactions proceeded smoothly to obtain the desired products **3b** and **3c** with 85–89% yields and 86–96% ee. C4, C5, C6 and C7-substituted indoles **1d**–**1o** underwent the reactions smoothly to give the corresponding products **3d**–**3o** in consistently high yields (86%–98%) and enantioselectivities (83–98% ee) with either electron-donating groups (Me, MeO, BnO) or electron-withdrawing (Cl, Br) groups in the phenyl ring. The electronic properties of the substituents in the phenyl ring of the indoles have no obvious effect on the reaction. As for the 1,3-diarylallyl acetate components, the substrates bearing either electron-donating (OMe) or electron-withdrawing (NO_2_) substituents at the *para* position of the benzene ring underwent the reaction smoothly to give the corresponding products in good yield with excellent ee values (Table 3, **3p**–**3s**). To summarize Table 3, the ligand **L1** exhibits superior catalytic performance for allylic alkylation reaction of 1,3-diaryl-2-propenyl acetate and indoles.

Encouraged by the excellent results achieved in the asymmetric allylic alkylation of indole, we successfully extended the current catalytic system to Pd-catalyzed asymmetric allylic etherification [38,39,40,41] by directly utilizing relatively hard aliphatic alcohols as nucleophiles (Table 4). We were pleased to find that the protocol is applicable to aromatic rings of benzylicalcohols with different electronic and steric natures. For example, a wide variety of benzylic alcohols bearing electron-donating (MeO) or electron-withdrawing (Br) groups at *ortho*-, *meta*-, or *para*-positions were well tolerated, and afforded the corresponding products **5a**–**5e** in consistently high yields and enantioselectivities with dichloromethane (DCM) as the solvent (Table S1). When the aryl group was changed to naphthyl and aromatic heterocycle, good yields and ee values were also observed (**5f** and **5g**). Notably, spiro phosphine-oxazoline ligand **L1** also worked efficiency in the asymmetric allylic etherification of simple aliphatic alcohols, including primary and secondary alcohols, which led to the formation of the desired products in excellent enantioselectivities (**5h**–**5j**, 93–99% ee). 

## 3. Materials and Methods 

### 3.1. General Information 

All solvents were purified and dried according to standard methods prior to use. All air and moisture sensitive manipulations were carried out with standard Schlenk techniques under argon. Commercially available reagents were used without further purification. Melting points were recorded on a RY-1 microscopic melting apparatus and uncorrected. ^1^H- and ^13^C-nuclear magnetic resonance (NMR) spectra were recorded on a Bruker Avance 500 spectrometer (Bruker, Rheinstetten, Germany). Chemical shifts were reported in parts per million (δ) relative to tetramethylsilane (TMS). High resolution mass spectrometry (HRMS) were performed on an Ultima Global spectrometer (Waters, Milford, MA, USA) with an electrospray ionization (ESI) source. HPLC was performed on a Shimadzu LC-20 Liquid Chromatograph ( Shimadzu, Kyoto, Japan) using chiralcel OD-H, AD-H and OJ-H columns. Ligands **L1**–**L4** were prepared according to the reported procedure [36].

### 3.2. General Procedure for the Pd-Catalyzed Asymmetric Allylic Alkylation of Indoles

Ligand **L1** (5.0 mg, 4 mol%) and [Pd(C_3_H_5_)Cl]_2_ (2.2 mg, 2 mol%) were dissolved in toluene (1.0 mL) in a Schlenk tube under Ar. After 0.5 h of stirring at room temperature, allylic acetate **2** (0.36 mmol) dissolved in toluene (0.5 mL) was added, followed by indole **1** (0.3 mmol), and Cs_2_CO_3_ (195 mg, 0.6 mmol). The mixture was stirred at room temperature for 12 h and then was diluted with CH_2_Cl_2_ and washed with saturated NH_4_Cl (aq). The organic layers were dried over MgSO_4_ and filtered, and the solvents were evaporated in vacuo. The residue was purified by flash column chromatography, eluting with petroleum ether and ethyl acetate to afford the corresponding product **3**.

*(S,E)-3-(1,3-diphenylallyl)-1H-indole* (**3a**) [15]: Yellow solid (m.p. 121–123 °C), 95% yield. ^1^H-NMR (500 MHz, CDCl_3_) δ 7.98 (brs, 1H), 7.45 (d, *J* = 8.0 Hz, 1H), 7.39–7.19 (m, 12H), 7.04 (t, 7.4 Hz, 1H), 6.91 (s, 1H), 6.76 (dd, *J* = 15.8, 7.4 Hz, 1H), 6.47 (d, *J* = 15.8 Hz, 1H), 5.14 (d, *J* = 7.4 Hz, 1H); ^13^C-NMR (125 MHz, CDCl_3_) δ 143.3, 137.4, 136.6, 132.5, 130.5, 128.4, 128.3, 127.1, 126.7, 126.3, 126.2, 122.5, 122.0, 119.8, 119.3, 118.6, 111.0, 46,1. 

*(S,E)-3-(1,3-diphenylallyl)-2-methyl-1H-indole* (**3b**) [15]: Yellow solid (m.p. 39–41 °C), 89% yield. ^1^H-NMR (500 MHz, CDCl_3_) δ 7.83 (brs, 1H), 7.40–7.36 (m, 5H), 7.31–7.28 (m, 5H), 7.23–7.21 (m, 2H), 7.12 (t, *J* = 7.3 Hz, 1H), 7.00 (t, *J* = 7.5 Hz, 1H), 6.88 (dd, *J* = 15.8, 7.2 Hz, 1H), 6.45 (d, *J* = 15.8 Hz, 1H), 5.17 (d, *J* = 7.2 Hz, 1H), 2.40 (s, 3H); ^13^C-NMR (125 MHz, CDCl_3_) δ 143.4, 137.5, 135.3, 132.1, 131.6, 130.5, 128.4, 128.3, 128.2, 127.9, 127.0, 126.2, 126.1, 120.9, 119.4, 119.2, 112.8, 110.2, 45.0, 12.4.

*(S,E)-3-(1,3-diphenylallyl)-2-phenyl-1H-indole* (**3c**) [15]: Yellow solid (m.p. 40–42 °C), 85% yield. ^1^H-NMR (500 MHz, CDCl_3_) δ 8.09 (brs, 1H), 7.53 (d, *J* = 7.4 Hz, 2H), 7.45–7.31 (m, 9H), 7.26–7.24 (m, 4H), 7.19–7.14 (m, 3H), 6.99 (t, *J* = 7.6 Hz, 1H), 6.90 (dd, *J* = 15.8, 7.3 Hz, 1H), 6.40 (d, *J* = 15.8 Hz, 1H), 5.27 (d, *J* = 8.4 Hz, 1H); ^13^C-NMR (125 MHz, CDCl_3_) δ 143.4, 137.4, 136.2, 135.5, 132.9, 132.2, 131.0, 128.7, 128.6, 128.5, 128.4, 128.2, 128.1, 128.0, 127.8, 127.0, 126.2, 126.0, 122.0, 121.1, 119.6, 113.8, 110.8, 45.1.

*(S,E)-3-(1,3-diphenylallyl)-4-methyl-1H-indole* (**3d**) [17]: White solid (m.p. 172–174 °C), 93% yield. ^1^H-NMR (500 MHz, CDCl_3_) δ 8.00 (brs, 1H), 7.35 (d, *J* = 7.4 Hz, 2H), 7.32–7.26 (m, 6H), 7.25–7.18 (m, 3H), 7.07 (t, *J* = 7.5 Hz, 1H), 6.86 (d, *J* = 2.2 Hz, 1H), 6.81 (d, *J* = 7.0 Hz, 1H), 6.75 (dd, *J* = 15.8, 6.6 Hz, 1H), 6.25 (d, *J* = 15.8 Hz, 1H), 5.46 (d, *J* = 6.4 Hz, 1H), 2.53 (s, 3H); ^13^C-NMR (125 MHz, CDCl_3_) δ 144.1, 137.6, 137.1, 134.0, 131.2, 130.8, 129.0, 128.6, 127.2, 126.4, 125.7, 123.6, 122.3, 121.4, 119.1, 109.1, 46.6, 20.5.

*(S,E)-3-(1,3-diphenylallyl)-4-methoxy-1H-indole* (**3e**): Yellow oil, 95% yield. ^1^H-NMR (500 MHz, CDCl_3_) δ 7.96 (brs, 1H), 7.36 (d, *J* = 7.6 Hz, 2H), 7.31–7.25 (m, 6H), 7.19–7.16 (m, 2H), 7.07 (t, *J* = 8.0 Hz, 1H), 6.96 (d, *J* = 8.2 Hz, 1H), 6.83 (d, *J* = 1.8 Hz, 1H), 6.77 (dd, *J* = 15.8, 7.2 Hz, 1H), 6.45 (d, *J* = 7.8 Hz, 1H), 6.35 (d, *J* = 15.8 Hz, 1H), 5.55 (d, *J* = 7.2 Hz, 1H), 3.73 (s, 3H); ^13^C-NMR (125 MHz, CDCl_3_) δ 154.8, 144.7, 138.1, 137.9, 134.0, 129.9, 128.5, 128.4, 128.0, 126.8, 126.2, 125.7, 122.9, 121.1, 119.3, 117.0, 104.3, 100.0, 55.0, 46.3. HRMS (ESI): calcd for C_24_H_21_NNaO [M + Na]^+^: 362.1515, found 362.1510.

*(S,E)-3-(1,3-diphenylallyl)-5-methyl-1H-indole* (**3f**) [15]: White solid (m.p. 113–114 °C), 96% yield. ^1^H-NMR (500 MHz, CDCl_3_) δ 7.90 (brs, 1H), 7.38–7.26 (m, 8H), 7.25–7.19 (m, 4H), 7.01 (d, 8.2 Hz, 1H), 6.86 (s, 1H), 6.73 (dd, *J* = 15.8, 7.3 Hz, 1H), 6.44 (d, *J* = 15.8 Hz, 1H), 5.11 (d, *J* = 7.2 Hz, 1H), 2.38 (s, 3H); ^13^C-NMR (125 MHz, CDCl_3_) δ 143.6, 137.7, 135.0, 132.8, 130.6, 128.6, 127.2, 126.4, 123.8, 122.9, 119.5, 118.2, 110.9, 46.1, 21.6.

*(S,E)-3-(1,3-diphenylallyl)-5-methoxy-1H-indole* (**3g**) [15]: Colorless oil, 98% yield. ^1^H-NMR (500 MHz, CDCl_3_) δ 7.90 (brs, 1H), 7.38–7.19 (m, 11H), 6.89–6.83 (m, 3H), 6.74 (dd, *J* = 15.8, 7.4 Hz, 1H), 6.47 (d, *J* = 15.8 Hz, 1H), 5.09 (d, *J* = 7.3 Hz, 1H), 3.72 (s, 3H); ^13^C-NMR (125 MHz, CDCl_3_) δ 154.0, 143.5, 137.7, 132.7, 132.0, 130.8, 128.7, 128.6, 127.4, 127.3, 126.6, 126.5, 123.6, 118.6, 112.4, 111.9, 102.0, 56.0, 46.4.

*(S,E)-5-(benzyloxy)-3-(1,3-diphenylallyl)-1H-indole* (**3h**) [15]: Colorless oil, 97% yield. ^1^H-NMR (500 MHz, CDCl_3_) δ 7.82 (brs, 1H), 7.37–7.17 (m, 16H), 6.92–6.88 (m, 2H), 6.82 (s, 1H), 6.70 (dd, *J* = 15.8, 7.4 Hz, 1H), 6.43 (d, *J* = 15.8 Hz, 1H), 5.04 (d, *J* = 7.3 Hz, 1H), 4.93 (s, 2H); ^13^C-NMR (125 MHz, CDCl_3_) δ 152.9, 143.3, 137.6, 137.5, 132.4, 131.9, 130.5, 128.5, 127.7, 127.6, 127.2, 126.4, 126.3, 123.5, 118.3, 112.9, 111.8, 103.4, 70.8, 46.2.

*(S,E)-5-chloro-3-(1,3-diphenylallyl)-1H-indole* (**3i**) [15]: Yellow oil, 95% yield. ^1^H-NMR (500 MHz, CDCl_3_) δ 8.01 (brs, 1H), 7.38–7.20 (m, 12H), 7.13–7.11 (m, 1H), 6.94 (s, 1H), 6.69 (dd, *J* = 15.8, 7.3 Hz, 1H), 6.43 (d, *J* = 15.8 Hz, 1H), 5.07 (d, *J* = 7.3 Hz, 1H); ^13^C-NMR (125 MHz, CDCl_3_) δ 143.0, 137.4, 135.0, 132.1, 130.9, 128.6, 128.5, 128.0, 127.3, 126.6, 126.4, 125.2, 124.0, 122.5, 119.3, 118.6, 112.2, 46.0.

*(S,E)-5-bromo-3-(1,3-diphenylallyl)-1H-indole* (**3j**) [15]: Colorless oil, 92% yield. ^1^H-NMR (500 MHz, CDCl_3_) δ 8.00 (brs, 1H), 7.53 (s, 1H), 7.36 (d, *J* = 7.6 Hz, 2H), 7.32–7.28 (m, 5H), 7.26–7.19 (m, 5H), 6.90 (d, *J* = 2.2 Hz, 1H), 6.69 (dd, *J* = 15.8, 7.3 Hz, 1H), 6.42 (d, *J* = 15.8 Hz, 1H), 5.05 (d, *J* = 7.3 Hz, 1H); ^13^C-NMR (125 MHz, CDCl_3_) δ 142.8, 137.2, 135.2, 132.0, 130.7, 128.4, 128.3, 127.2, 126.5, 126.3, 125.0, 123.8, 122.2, 118.4, 112.7, 112.5, 45.8.

*(S,E)-3-(1,3-diphenylallyl)-6-methyl-1H-indole* (**3k**) [20]: Yellow oil, 90% yield. ^1^H-NMR (500 MHz, CDCl_3_) δ 7.85 (brs, 1H), 7.37–7.17 (m, 11H), 7.14 (s, 1H), 6.86–6.83 (m, 2H), 6.74 (dd, *J* = 15.8, 7.4 Hz, 1H), 6.45 (d, *J* = 15.8 Hz, 1H), 5.09 (d, *J* = 7.4 Hz, 1H), 2.43 (s, 3H); ^13^C-NMR (125 MHz, CDCl_3_) δ 143.4, 137.5, 137.1, 132.6, 131.8, 130.4, 128.4, 128.3, 127.1, 126.3, 124.6, 121.9, 121.1, 119.5, 118.4, 111.0, 46.2, 21.6.

*(S,E)-6-chloro-3-(1,3-diphenylallyl)-1H-indole* (**3l**) [20]: Yellow oil, 94% yield. ^1^H-NMR (500 MHz, CDCl_3_) δ 8.01 (brs, 1H), 7.38–7.28 (m, 10H), 7.26–7.20 (m, 2H), 7.00 (dd, *J* = 8.5, 1.8 Hz, 1H), 6.91 (d, *J* = 2.0 Hz, 1H), 6.72 (dd, *J* = 15.8, 7.4 Hz, 1H), 6.44 (d, *J* = 15.8 Hz, 1H), 5.09 (d, *J* = 7.4 Hz, 1H); ^13^C-NMR (125 MHz, CDCl_3_) δ 143.0, 137.2, 137.0, 132.1, 130.8, 128.5, 128.4, 128.0, 127.3, 126.6, 126.3, 125.4, 123.3, 120.8, 120.2, 118.9, 111.1, 46.1.

*(S,E)-3-(1,3-diphenylallyl)-7-methyl-1H-indole* (**3m**) [19]: Yellow oil, 90% yield. ^1^H-NMR (500 MHz, CDCl_3_) δ 7.89 (brs, 1H), 7.41–7.30 (m, 9H), 7.28-7.22 (m, 2H), 7.03–6.98 (m, 2H), 6.92 (s, 1H), 6.78 (dd, *J* = 15.8, 7.3 Hz, 1H), 6.49 (d, *J* = 15.8 Hz, 1H), 5.16 (d, *J* = 7.2 Hz, 1H), 2.50 (s, 3H); ^13^C-NMR (125 MHz, CDCl_3_) δ 143.4, 137.5, 136.2, 132.5, 130.5, 128.4, 128.3, 127.1, 126.3, 122.6, 122.3, 120.2, 119.6, 119.1, 117.6, 46.2, 16.5.

*(S,E)-3-(1,3-diphenylallyl)-7-methoxy-1H-indole* (**3n**): Yellow oil, 86% yield. ^1^H-NMR (500 MHz, CDCl_3_) *δ* 8.21 (brs, 1H), 7.37–7.17 (m, 10H), 7.03 (d, *J* = 8.0 Hz, 1H), 6.95-6.88 (m, 2H), 6.74 (dd, *J* = 15.8, 7.4 Hz, 1H), 6.63 (d, *J* = 7.6 Hz, 1H), 6.44 (d, *J* = 15.8 Hz, 1H), 5.10 (d, *J* = 7.4 Hz, 1H), 3.94 (s, 3H); ^13^C-NMR (125 MHz, CDCl_3_) *δ* 146.1, 143.5, 137.5, 132.6, 130.5, 128.4, 128.2, 127.1, 122.2, 119.8, 119.1, 112.7, 101.9, 55.3, 46.3. HRMS (ESI): calcd for C_24_H_21_NNaO [M+Na]^+^: 362.1515, found 362.1516.

*(S,E)-7-chloro-3-(1,3-diphenylallyl)-1H-indole* (**3o**): Yellow solid (m.p. 81–82 °C), 90% yield. ^1^H-NMR (500 MHz, CDCl_3_) δ 8.23 (brs, 1H), 7.38–7.28 (m, 9H), 7.24–7.17 (m, 3H), 6.98–6.94 (m, 2H), 6.73 (dd, *J* = 15.8, 7.3 Hz, 1H), 6.45 (d, *J* = 15.8 Hz, 1H), 5.11 (d, *J* = 7.3 Hz, 1H); ^13^C-NMR (125 MHz, CDCl_3_) δ 143.0, 137.3, 133.9, 132.1, 130.9, 128.5, 128.4, 128.3, 127.3, 126.6, 126.4, 123.3, 121.5, 120.3, 119.9, 118.6, 116.6, 46.2. HRMS (ESI): calcd for C_23_H_18_ClNNa [M + Na]^+^: 366.1020, found 366.1018.

*(S,E)-3-(1,3-di-p-tolylallyl)-1H-indole* (**3p**) [19]: Yellow oil, 92% yield. ^1^H-NMR (500 MHz, CDCl_3_) δ 7.97 (brs, 1H), 7.46 (d, J = 8.0 Hz, 1H), 7.37 (d, *J* = 8.1 Hz, 1H), 7.28–7.02 (m, 10H), 6.91 (s, 1H), 6.70 (dd, *J* = 15.8, 7.4 Hz, 1H), 6.44 (d, *J* = 15.8 Hz, 1H), 5.09 (d, *J* = 7.2 Hz, 1H), 2.35 (s, 3H), 2.33 (s, 3H); ^13^C-NMR (125 MHz, CDCl_3_) δ 140.7, 137.0, 136.8, 136.0, 135.0, 132.0, 130.4, 129.4, 129.3, 128.6, 127.0, 126.4, 122.8, 122.2, 120.1, 119.6, 119.2, 111.3, 46.0, 21.3.

*(S,E)-3-(1,3-bis(4-methoxyphenyl)allyl)-1H-indole* (**3q**) [43]: Yellow oil, 85% yield. ^1^H-NMR (500 MHz, CDCl_3_) δ 7.98 (s, 1H), 7.43–7.28 (m, 4H), 7.24 (d, *J* = 8.2 Hz, 2H), 7.16 (t, *J* = 7.6 Hz, 1H), 7.02 (t, *J* = 7.6 Hz, 1H), 6.89 (s, 1H), 6.85–6.81 (m, 4H), 6.56 (dd, *J* = 15.8, 7.3 Hz, 1H), 6.36 (d, *J* = 15.8 Hz, 1H), 5.05 (d, *J* = 7.3 Hz, 1H), 3.78 (s, 6H); ^13^C-NMR (125 MHz, CDCl_3_) δ 158.9, 158.1, 136.7, 135.8, 130.8, 130.4, 129.7, 129.5, 127.5, 126.9, 122.6, 122.0, 120.0, 119.4, 119.2, 113.9, 113.8, 111.2, 55.3, 55.2, 45.4. 

*(S,E)-3-(1,3-bis(4-chlorophenyl)allyl)-1H-indole* (**3r**) [19]: Yellow oil, 91% yield. ^1^H-NMR (500 MHz, CDCl_3_) δ 8.05 (brs, 1H), 7.39 (d, *J* = 8.2 Hz, 2H), 7.30–7.19 (m, 9H), 7.06 (t, *J* = 7.5 Hz, 1H), 6.92 (s, 1H), 6.69 (dd, *J* = 15.8, 7.2 Hz, 1H), 6.38 (d, *J* = 15.8 Hz, 1H), 5.11 (d, *J* = 7.2 Hz, 1H); ^13^C-NMR (125 MHz, CDCl_3_) δ 141.8, 136.9, 135.9, 133.1, 132.8, 132.4, 130.0, 129.9, 128.9, 128.8, 127.7, 126.7, 122.8, 122.5, 119.9, 119.8, 118.2, 111.4, 45.7.

*(S,E)-3-(1,3-bis(4-nitrophenyl)allyl)-1H-indole* (**3s**): Yellow oil, 87% yield. ^1^H-NMR (500 MHz, CDCl_3_) δ 8.23–8.11 (m, 5H), 7.50–7.32 (m, 6H), 7.22 (t, *J* = 7.4 Hz, 1H), 7.06 (t, *J* = 7.5 Hz, 1H), 6.98 (s, 1H), 6.90 (dd, *J* = 15.8, 7.2 Hz, 1H), 6.5 (d, *J* = 15.8 Hz, 1H), 5.28 (d, *J* = 8.0 Hz, 1H); ^13^C-NMR (125 MHz, CDCl_3_) δ 150.2, 146.8, 143.4, 136.7, 135.7, 130.0, 129.4, 127.0, 126.2, 124.0, 123.9, 122.8, 122.7, 119.9, 119.2, 116.2, 111.6, 46.1. HRMS (ESI): calcd for C_23_H_17_N_3_NaO_4_ [M + Na]^+^: 422.1111, found 422.1110.

### 3.3. General Procedure for the N-Boc Protection of Alkylated Indoles 

To a solution of **3** (0.15 mmol) and DMAP (0.9 mg, 0.0075 mmol) in CH_2_Cl_2_ (3 mL) was added (Boc)_2_O (49 mg, 0.225 mmol), and the solution was stirred for 0.5 h at room temperature. The resulting mixture was evaporated under reduced pressure and purified by flash column chromatography (elution with *n*-hexane/EtOAc = 30:1) to afford the *N*-Boc-protected alkylated indole (**Boc-3**).

*(S,E)-tert-butyl 3-(1,3-diphenylallyl)-1H-indole-1-carboxylate* (**Boc-3a**) [15]: Colorless oil. 99% yield and 96% ee, determined by chiral HPLC analysis (Chiralcel OD-H hexane/isopropanol, 99:1 *v*/*v*, 0.4 mL/min, 25 °C, UV 254 nm), Retention times: t_r_ = 13.2 min for (*S*)-isomer (major), t_r_ = 16.0 min for (*R*)-isomer (minor). ^1^H-NMR (500 MHz, CDCl_3_) δ 8.12 (brs, 1H), 7.39–7.21 (m, 13H), 7.14 (t, *J* = 7.6 Hz, 1H), 6.72 (dd, *J* = 15.8, 7.4 Hz, 1H), 6.46 (d, *J* = 15.8 Hz, 1H), 5.06 (d, *J* = 7.4 Hz, 1H), 1.68 (s, 9H); ^13^C-NMR (125 MHz, CDCl_3_) δ 150.0, 142.1, 137.3, 135.8, 131.4, 131.3, 129.9, 128.6, 128.5, 128.4, 127.4, 126.8, 126.4, 124.4, 123.8, 123.1, 122.5, 120.2, 115.3, 83.7, 46.0, 28.3.

*(S,E)-tert-butyl 3-(1,3-diphenylallyl)-2-methyl-1H-indole-1-carboxylate* (**Boc-3b**) [15]: Colorless oil. 99% yield and 96% ee, determined by chiral HPLC analysis (Chiralcel OJ-H hexane/isopropanol, 99:1 *v*/*v*, 0.3 mL/min, 25 °C, UV 254 nm), Retention times: t_r_ = 37.2 min for (*R*)-isomer (minor), t_r_ = 50.1 min for (*S*)-isomer (major). ^1^H-NMR (500 MHz, CDCl_3_) δ 8.14 (d, *J* = 8.4 Hz, 1H), 7.39 (d, *J* = 7.5 Hz, 2H), 7.34–7.27 (m, 7H), 7.23–7.19 (m, 3H), 7.07 (t, *J* = 7.5 Hz, 1H), 6.83 (dd, *J* = 15.8, 7.4 Hz, 1H), 6.48 (d, *J* = 16.0 Hz, 1H), 5.20 (d, *J* = 7.4 Hz, 1H), 2.62 (s, 3H), 1.70 (s, 9H); ^13^C-NMR (125 MHz, CDCl_3_) δ 150.7, 142.3, 137.2, 136.0, 134.0, 131.5, 130.5, 128.9, 128.5, 128.3, 128.0, 127.2, 126.3, 123.1, 122.2, 119.5, 119.1, 115.3, 83.6, 44.5, 28.3, 14.3.

*(S,E)-tert-butyl 3-(1,3-diphenylallyl)-2-phenyl-1H-indole-1-carboxylate* (**Boc-3c**) [15]: White solid (m.p. 153–155 °C). 98% yield and 86% ee, determined by chiral HPLC analysis (Chiralcel AD-H hexane/isopropanol, 99:1 *v*/*v*, 0.8 mL/min, 25 °C, UV 254 nm), Retention times: t_r_ = 5.1 min for (*S*)-isomer (major), t_r_ = 13.4 min for (*R*)-isomer (minor). ^1^H-NMR (500 MHz, CDCl_3_) δ 8.29 (d, *J* = 8.6 Hz, 1H), 7.42–7.36 (m, 5H), 7.33–7.17 (m, 12H), 7.10 (t, *J* = 7.8 Hz, 1H), 6.77 (dd, *J* = 15.8, 7.6 Hz, 1H), 6.32 (d, *J* = 15.8 Hz, 1H), 4.85 (d, *J* = 7.6 Hz, 1H), 1.22 (s, 9H); ^13^C-NMR (125 MHz, CDCl_3_) δ 150.4, 142.8, 137.6, 137.2, 136.9, 134.5, 131.9, 130.9, 130.3, 128.7, 128.5, 128.4, 128.2, 128.1, 127.5, 126.6, 124.5, 122.8, 121.5, 121.2, 115.6, 83.3, 45.1, 27.8.

*(S,E)-tert-butyl 3-(1,3-diphenylallyl)-4-methyl-1H-indole-1-carboxylate* (**Boc-3d**) [18]: White solid (m.p. 137–139 °C). 98% yield and 84% ee, determined by chiral HPLC analysis (Chiralcel OD-H hexane/isopropanol, 99:1 *v*/*v*, 0.7 mL/min, 25 °C, UV 254 nm), Retention times: t_r_ = 7.1 min for (*S*)-isomer (major), t_r_ = 8.5 min for (*R*)-isomer (minor). ^1^H-NMR (500 MHz, CDCl_3_) δ 8.07 (brs, 1H), 7.38 (d, *J* = 7.4 Hz, 2H), 7.35–7.25 (m, 8H), 7.24–7.19 (m, 2H), 6.94 (d, *J* = 7.3 Hz, 1H), 6.73 (dd, *J* = 15.8, 6.6 Hz, 1H), 6.31 (d, *J* = 15.8 Hz, 1H), 5.37 (d, *J* = 6.6 Hz, 1H), 2.50 (s, 3H), 1.68 (s, 9H); ^13^C-NMR (125 MHz, CDCl_3_) δ 149.9, 142.8, 137.4, 136.4, 132.7, 131.3, 131.2, 128.9, 128.6, 127.4, 126.7, 126.5, 125.0, 124.9, 124.4, 123.5, 113.2, 83.7, 46.6, 28.3, 27.5, 20.4.

*(S,E)-tert-butyl 3-(1,3-diphenylallyl)-4-methoxy-1H-indole-1-carboxylate* (**Boc-3e**): Colorless oil. 99% yield and 92% ee, determined by chiral HPLC analysis (Chiralcel OD-H hexane/isopropanol, 99:1 *v*/*v*, 0.7 mL/min, 25 °C, UV 254 nm), Retention times: t_r_ = 7.8 min for (*S*)-isomer (major), t_r_ = 11.6 min for (*R*)-isomer (minor). ^1^H-NMR (500 MHz, CDCl_3_) δ 7.73 (brs, 1H), 7.37 (d, *J* = 7.5 Hz, 2H), 7.30–7.27 (m, 7H), 7.20 (t, *J* = 7.8 Hz, 3H), 6.73 (dd, *J* = 15.8, 7.4 Hz, 1H), 6.60 (d, *J* = 8.0 Hz, 1H), 6.37 (d, *J* = 15.8 Hz, 1H), 5.46 (d, *J* = 7.4 Hz, 1H), 3.70 (s, 3H), 1.66 (s, 9H); ^13^C-NMR (125 MHz, CDCl_3_) δ 154.3, 150.0, 143.5, 137.7, 137.3, 132.9, 130.5, 128.5, 128.2, 127.1, 126.4, 126.1, 125.3, 123.4, 122.6, 119.6, 108.3, 103.8, 93.7, 55.2, 46.5, 28.3. HRMS (ESI): calcd for C_29_H_29_NNaO_3_ [M + Na]^+^: 462.2040, found 462.2033.

*(S,E)-tert-butyl 3-(1,3-diphenylallyl)-5-methyl-1H-indole-1-carboxylate* (**Boc-3f**) [15]: White solid (m.p. 151–152 °C). 98% yield and 95% ee, determined by chiral HPLC analysis (Chiralcel OD-H hexane/isopropanol, 99:1 *v*/*v*, 0.7 mL/min, 25 °C, ultraviolet (UV) 254 nm), Retention times: t_r_ = 7.5 min for (*S*)-isomer (major), t_r_ = 8.9 min for (*R*)-isomer (minor). ^1^H-NMR (500 MHz, CDCl_3_) δ 7.96 (brs, 1H), 7.37–7.27 (m, 9H), 7.25–7.19 (m, 2H), 7.12–7.09 (m, 2H), 6.68 (dd, *J* = 15.8, 7.3 Hz, 1H), 6.42 (d, *J* = 15.8 Hz, 1H), 5.01 (d, *J* = 7.3 Hz, 1H), 2.35 (s, 3H), 1.65 (s, 9H); ^13^C-NMR (125 MHz, CDCl_3_) δ 150.0, 142.2, 137.3, 134.0, 131.9, 131.4, 131.3, 130.1, 128.6, 128.5, 127.4, 126.7, 126.4, 125.8, 123.9, 122.8, 119.9, 114.9, 83.5, 45.9, 28.3, 21.5.

*(S,E)-tert-butyl 3-(1,3-diphenylallyl)-5-methoxy-1H-indole-1-carboxylate* (**Boc-3g**) [15]: Yellow oil. 98% yield and 98% ee, determined by chiral HPLC analysis (Chiralcel OD-H hexane/isopropanol, 99:1 *v*/*v*, 0.7 mL/min, 25 °C, UV 254 nm), Retention times: t_r_ = 8.0 min for (*S*)-isomer (major), t_r_ = 10.2 min for (*R*)-isomer (minor). ^1^H-NMR (500 MHz, CDCl_3_) δ 7.99 (brs, 1H), 7.39–7.22 (m, 11H), 6.91 (dd, *J* = 9.0, 2.4 Hz, 1H), 6.78 (d, *J* = 2.3 Hz, 1H), 6.69 (dd, *J* = 15.8, 7.4 Hz, 1H), 6.46 (d, *J* = 15.8 Hz, 1H), 5.00 (d, *J* = 7.3 Hz, 1H), 3.73 (s, 3H), 1.67 (s, 9H); ^13^C-NMR (125 MHz, CDCl_3_) δ 155.8, 155.0, 142.2, 137.4, 131.5, 131.3, 130.9, 128.8, 128.7, 128.6, 127.6, 126.9, 126.6, 124.7, 123.0, 116.1, 112.9, 103.3, 83.7, 55.8, 46.2, 28.4, 27.6.

*(S,E)-tert-butyl 5-(benzyloxy)-3-(1,3-diphenylallyl)-1H-indole-1-carboxylate* (**Boc-3h**) [15]: Colorless oil. 99% yield and 96% ee, determined by chiral HPLC analysis (Chiralcel OD-H hexane/isopropanol, 99:1 *v*/*v*, 0.35 mL/min, 25 °C, UV 254 nm), Retention times: t_r_ = 34.3 min for (*S*)-isomer (major), t_r_ = 38.3 min for (*R*)-isomer (minor). ^1^H-NMR (500 MHz, CDCl_3_) δ 8.00 (brs, 1H), 7.38–7.30 (m, 13H), 7.28–7.22 (m, 3H), 6.99 (dd, *J* = 9.0, 2.2 Hz, 1H), 6.86 (d, *J* = 2.2 Hz, 1H), 6.69 (dd, *J* = 15.8, 7.4 Hz, 1H), 6.45 (d, *J* = 15.8 Hz, 1H), 4.98 (d, *J* = 5.0 Hz, 3H), 1.67 (s, 9H); ^13^C-NMR (125 MHz, CDCl_3_) δ 154.9, 150.0, 142.2, 137.4, 131.5, 131.3, 130.9, 128.8, 128.7, 128.6, 128.0, 127.7, 127.6, 126.9, 126.6, 124.7, 123.0, 116.2, 113.9, 104.7, 83.7, 70.8, 46.2, 28.4.

*(S,E)-tert-butyl 5-chloro-3-(1,3-diphenylallyl)-1H-indole-1-carboxylate* (**Boc-3i**) [15]: White solid (m.p. 54–56 °C). 99% yield and 92% ee, determined by chiral HPLC analysis (Chiralcel OD-H hexane/isopropanol, 99:1 *v*/*v*, 0.7 mL/min, 25 °C, UV 254 nm), Retention times: t_r_ = 8.2 min for (*S*)-isomer (major), t_r_ = 9.0 min for (*R*)-isomer (minor). ^1^H-NMR (500 MHz, CDCl_3_) δ 8.02 (brs, 1H), 7.37–7.27 (m, 10H), 7.24–7.20 (m, 2H), 6.64 (dd, *J* = 15.8, 7.3 Hz, 1H), 6.42 (d, *J* = 15.8 Hz, 1H), 4.98 (d, *J* = 7.3 Hz, 1H), 1.65 (s, 9H); ^13^C-NMR (125 MHz, CDCl_3_) δ 149.6, 141.7, 137.1, 134.2, 131.6, 131.1, 130.8, 128.7, 128.6, 128.4, 128.2, 127.5, 127.0, 126.4, 125.1, 124.6, 122.5, 119.6, 116.4, 84.2, 45.8, 28.2.

*(S,E)-tert-butyl 5-bromo-3-(1,3-diphenylallyl)-1H-indole-1-carboxylate* (**Boc-3j**) [15]: White solid (m.p. 72–73 °C). 96% yield and 93% ee, determined by chiral HPLC analysis (Chiralcel OD-H hexane/isopropanol, 99:1 *v*/*v*, 0.7 mL/min, 25 °C, UV 254 nm), Retention times: t_r_ = 8.2 min for (*S*)-isomer (major), t_r_ = 8.8 min for (*R*)-isomer (minor). ^1^H-NMR (500 MHz, CDCl_3_) δ 7.99 (brs, 1H), 7.46 (s, 1H), 7.38–7.36 (m, 4H), 7.33–7.27 (m, 6H), 7.26–7.21 (m, 2H), 6.67 (dd, *J* = 15.8, 7.3 Hz, 1H), 6.43 (d, *J* = 15.8 Hz, 1H), 4.99 (d, *J* = 7.3 Hz, 1H), 1.65 (s, 9H); ^13^C-NMR (125 MHz, CDCl_3_) δ 149.4, 141.5, 137.0, 134.5, 131.5, 131.4, 130.7, 128.6, 128.4, 128.2, 127.4, 127.2, 126.8, 126.3, 124.8, 122.5, 122.3, 116.6, 115.8, 84.1, 45.6, 28.1.

*(S,E)-tert-butyl 3-(1,3-diphenylallyl)-6-methyl-1H-indole-1-carboxylate* (**Boc-3k**): White solid (m.p. 121–123 °C). 99% yield and 93% ee, determined by chiral HPLC analysis (Chiralcel OD-H hexane/isopropanol, 99:1 *v*/*v*, 0.7 mL/min, 25 °C, UV 254 nm), Retention times: t_r_ = 7.2 min for (*S*)-isomer (major), t_r_ = 8.2 min for (*R*)-isomer (minor). ^1^H-NMR (500 MHz, CDCl_3_) δ 7.97 (brs, 1H), 7.37–7.18 (m, 12H), 6.96 (d, *J* = 8.0 Hz, 1H), 6.69 (dd, *J* = 15.8, 7.4 Hz, 1H), 6.44 (d, *J* = 15.8 Hz, 1H), 5.01 (d, *J* = 7.4 Hz, 1H), 2.44 (s, 3H), 1.65 (s, 9H); ^13^C-NMR (125 MHz, CDCl_3_) δ 150.0, 142.2, 137.3, 136.3, 134.4, 131.3, 131.2, 128.6, 128.5, 128.4, 127.6, 127.4, 126.7, 126.4, 123.9, 123.1, 123.0, 119.7, 115.6, 83.5, 46.0, 28.2, 22.0. HRMS (ESI): calcd for C_29_H_29_NNaO_2_ [M + Na]^+^: 446.2091, found 446.2093.

*(S,E)-tert-butyl 6-chloro-3-(1,3-diphenylallyl)-1H-indole-1-carboxylate* (**Boc-3l**): Colorless oil. 98% yield and 93% ee, determined by chiral HPLC analysis (Chiralcel OD-H hexane/isopropanol, 99:1 *v*/*v*, 0.7 mL/min, 25 °C, UV 254 nm), Retention times: t_r_ = 8.5 min for (*S*)-isomer (major), t_r_ = 9.2 min for (*R*)-isomer (minor). ^1^H-NMR (500 MHz, CDCl_3_) δ 8.14 (brs, 1H), 7.36–7.19 (m, 12H), 7.10 (dd, *J* = 8.3, 1.7 Hz, 1H), 6.67 (dd, *J* = 15.8, 7.4 Hz, 1H), 6.43 (d, *J* = 15.8 Hz, 1H), 5.00 (d, *J* = 7.3 Hz, 1H), 1.66 (s, 9H); ^13^C-NMR (125 MHz, CDCl_3_) δ 171.2, 149.5, 141.8, 137.1, 136.2, 131.5, 130.9, 130.4, 128.7, 128.6, 128.4, 127.5, 126.9, 126.4, 124.3, 123.0, 122.9, 120.9, 115.6, 84.3, 60.4, 45.9, 28.2, 21.1, 14.2. HRMS (ESI): calcd for C_28_H_26_ClNNaO_2_ [M + Na]^+^: 466.1544, found 466.1547.

*(S,E)-tert-butyl 3-(1,3-diphenylallyl)-7-methyl-1H-indole-1-carboxylate* (**Boc-3m**) [19]: White solid (m.p. 123–124 °C). 99% yield and 83% ee, determined by chiral HPLC analysis (Chiralcel OD-H hexane/isopropanol, 99:1 *v*/*v*, 0.7 mL/min, 25 °C, UV 254 nm), Retention times: t_r_ = 7.6 min for (*S*)-isomer (major), t_r_ = 8.9 min for (*R*)-isomer (minor). ^1^H-NMR (500 MHz, CDCl_3_) δ 7.39–7.28 (m, 9H), 7.26–7.18 (m, 3H), 7.09–7.04 (m, 2H), 6.71 (dd, *J* = 15.8, 7.4 Hz, 1H), 6.45 (d, *J* = 15.8 Hz, 1H), 5.03 (d, *J* = 7.4 Hz, 1H), 2.64 (s, 3H), 1.63 (s, 9H); ^13^C-NMR (125 MHz, CDCl_3_) δ 149.6, 142.0, 137.2, 135.4, 131.2, 131.1, 131.0, 128.4, 128.3, 127.6, 127.2, 126.5, 126.3, 125.9, 125.3, 122.8, 122.4, 117.6, 83.2, 45.8, 28.0, 22.1.

*(S,E)-tert-butyl 3-(1,3-diphenylallyl)-7-methoxy-1H-indole-1-carboxylate* (**Boc-3n**): Colorless oil. 95% yield and 84% ee, determined by chiral HPLC analysis (Chiralcel OD-H hexane/isopropanol, 99:1 *v*/*v*, 0.7 mL/min, 25 °C, UV 254 nm), Retention times: t_r_ = 21.4 min for (*S*)-isomer (major), t_r_ = 49.0 min for (*R*)-isomer (minor). ^1^H-NMR (500 MHz, CDCl_3_) δ 7.37 (d, *J* = 7.5 Hz, 2H), 7.32–7.26 (m, 7H), 7.25–7.21 (m, 2H), 7.07 (t, *J* = 7.9 Hz, 1H), 6.96 (d, *J* = 7.8 Hz, 1H), 6.80 (d, *J* = 7.9 Hz, 1H), 6.69 (dd, *J* = 15.8, 7.4 Hz, 1H), 6.43 (d, *J* = 15.8 Hz, 1H), 5.01 (d, *J* = 7.4 Hz, 1H), 3.93 (s, 3H), 1.62 (s, 9H); ^13^C-NMR (125 MHz, CDCl_3_) δ 150.0, 148.4, 142.2, 137.3, 132.8, 131.4, 131.3, 128.6, 127.4, 126.7, 126.5, 126.4, 125.2, 123.5, 122.4, 112.8, 106.9, 83.3, 55.8, 46.0, 28.1. HRMS (ESI): calcd for C_29_H_29_NNaO_3_ [M+Na]^+^: 462.2040, found 462.2043.

*(S,E)-tert-butyl 7-chloro-3-(1,3-diphenylallyl)-1H-indole-1-carboxylate* (**Boc-3o**): Colorless oil. 99% yield and 94% ee, determined by chiral HPLC analysis (Chiralcel OD-H hexane/isopropanol, 99:1 *v*/*v*, 0.7 mL/min, 25 °C, UV 254 nm), Retention times: t_r_ = 11.6 min for (*S*)-isomer (major), t_r_ = 17.3 min for (*R*)-isomer (minor). ^1^H-NMR (500 MHz, CDCl_3_) δ 7.36–7.27 (m, 10H), 7.26–7.21 (m, 3H), 7.06 (t, *J* = 7.8 Hz, 1H), 6.67 (dd, *J* = 15.8, 7.4 Hz, 1H), 6.43 (d, *J* = 15.8 Hz, 1H), 5.00 (d, *J* = 7.4 Hz, 1H), 1.63 (s, 9H); ^13^C-NMR (125 MHz, CDCl_3_) δ 149.1, 141.7, 137.0, 133.2, 132.7, 131.5, 130.8, 128.6, 128.5, 128.4, 127.4, 127.3, 126.8, 126.5, 126.4, 123.4, 122.4, 120.4, 118.7, 84.5, 45.8, 27.9. HRMS (ESI): calcd for C_28_H_26_ClNNaO_2_ [M + Na]^+^: 466.1544, found 466.1539.

*(S,E)-tert-butyl 3-(1,3-di-p-tolylallyl)-1H-indole-1-carboxylate* (**Boc-3p**) [19]: Colorless oil. 99% yield and 94% ee, determined by chiral HPLC analysis (Chiralcel AD-H hexane/isopropanol, 99:1 *v*/*v*, 0.5 mL/min, 25 °C, UV 254 nm), Retention times: t_r_ = 12.0 min for (*S*)-isomer (major), t_r_ = 14.1 min for (*R*)-isomer (minor). ^1^H-NMR (500 MHz, CDCl_3_) δ 8.13 (brs, 1H), 7.38 (d, *J* = 8.2 Hz, 2H), 7.31–7.27 (m, 3H), 7.24–7.22 (m, 2H), 7.16–7.11 (m, 5H), 6.67 (dd, *J* = 15.8, 7.4 Hz, 1H), 6.43 (d, *J* = 15.8 Hz, 1H), 5.01 (d, *J* = 7.7 Hz, 1H), 2.35 (s, 3H), 2.34 (s, 3H), 1.69 (s, 9H); ^13^C-NMR (125 MHz, CDCl_3_) δ 150.1, 139.4, 137.2, 136.3, 136.0, 134.7, 131.1, 130.6, 130.1, 129.4, 129.3, 128.5, 126.4, 124.5, 123.9, 123.5, 122.6, 120.4, 115.4, 83.8, 45.7, 28.4, 21.3.

*(S,E)-tert-butyl 3-(1,3-bis(4-methoxyphenyl)allyl)-1H-indole-1-carboxylate* (**Boc-3q**): Yellow oil. 85% yield and 90% ee, determined by chiral HPLC analysis (Chiralcel OD-H hexane/isopropanol, 99:1 *v*/*v*, 0.7 mL/min, 25 °C, UV 254 nm), Retention times: t_r_ = 17.5 min for (*S*)-isomer (major), t_r_ = 19.3 min for (*R*)-isomer (minor). ^1^H-NMR (500 MHz, CDCl_3_) δ 8.08 (s, 1H), 7.33–7.74 (m, 5H), 7.22 (d, *J* = 8.6 Hz, 2H), 7.11 (t, *J* = 7.5 Hz, 1H), 6.82 (t, *J* = 9.2 Hz, 4H), 6.51 (dd, *J* = 15.8, 7.2 Hz, 1H), 6.34 (d, *J* = 15.8 Hz, 1H), 4.95 (d, *J* = 7.2 Hz, 1H), 3.78 (s, 6H), 1.65 (s, 9H); ^13^C-NMR (125 MHz, CDCl_3_) δ 159.1, 158.3, 150.0, 134.4, 130.4, 130.1, 130.0, 129.4, 127.5, 124.3, 123.7, 123.6, 122.4, 120.2, 115.3, 113.9, 83.7, 55.3, 55.2, 45.1, 28.3. HRMS (ESI): calcd for C_30_H_31_NNaO_4_ [M + Na]^+^: 492.2145, found 492.2150.

*(S,E)-tert-butyl 3-(1,3-bis(4-chlorophenyl)allyl)-1H-indole-1-carboxylate* (**Boc-3r**) [19]: White solid (m.p. 65–67 °C). 99% yield and 95% ee, determined by chiral HPLC analysis (Chiralcel OD-H hexane/isopropanol, 99:1 *v*/*v*, 0.7 mL/min, 25 °C, UV 254 nm), Retention times: t_r_ = 8.6 min for (*S*)-isomer (major), t_r_ = 11.7 min for (*R*)-isomer (minor). ^1^H-NMR (500 MHz, CDCl_3_) δ 8.11 (brs, 1H), 7.36 (s, 1H), 7.32–7.23 (m, 10H), 7.14 (t, *J* = 7.5 Hz, 1H), 6.64 (dd, *J* = 15.8, 7.2 Hz, 1H), 6.37 (d, *J* = 15.8 Hz, 1H), 5.01 (d, *J* = 7.2 Hz, 1H), 1.68 (s, 9H); ^13^C-NMR (125 MHz, CDCl_3_) δ 150.0, 140.5, 136.0, 135.6, 133.3, 132.8, 131.5, 130.7, 129.9, 129.7, 129.0, 128.9, 127.8, 124.7, 124.0, 122.7, 122.4, 120.1, 115.5, 84.1, 45.4, 28.4.

*(S,E)-tert-butyl 3-(1,3-bis(4-nitrophenyl)allyl)-1H-indole-1-carboxylate* (**Boc-3s**): Yellow oil. 87% yield and 97% ee, determined by chiral HPLC analysis (Chiralcel AD-H hexane/isopropanol, 93:7 *v*/*v*, 1.0 mL/min, 25 °C, UV 254 nm), Retention times: t_r_ = 43.5 min for (*R*)-isomer (minor), t_r_ = 54.4 min for (*S*)-isomer (major). ^1^H-NMR (500 MHz, CDCl_3_) δ 8.21–8.12 (m, 5H), 7.50 (d, *J* = 7.2 Hz, 4H), 7.42 (s, 1H), 7.33 (t, *J* = 7.6 Hz, 1H), 7.24–7.14 (m, 2H), 6.85 (dd, *J* = 15.8, 7.2 Hz, 1H), 6.51 (d, *J* = 15.8 Hz, 1H), 5.21 (d, *J* = 7.2 Hz, 1H), 1.68 (s, 9H); ^13^C-NMR (125 MHz, CDCl_3_) δ 148.7, 147.1, 142.9, 134.3, 130.7, 129.3, 129.0, 127.0, 124.9, 124.1, 122.8, 120.6, 119.5, 115.6, 84.3, 45.8, 28.2. HRMS (ESI): calcd for C_28_H_25_N_3_NaO_6_ [M + Na]^+^: 522.1636, found 522.1632.

### 3.4. General Procedure for the Pd-Catalyzed Asymmetric Allylic Etherification 

Ligand **L1** (5.0 mg, 4 mol%) and [Pd(C_3_H_5_)Cl]_2_ (2.2 mg, 2 mol%) were dissolved in DCM (1.0 mL) in a Schlenk tube under Ar. After 0.5 h of stirring at room temperature, allylic acetate **2a** (0.3 mmol) dissolved in DCM (0.5 mL) was added, followed by alcohol **4** (0.9 mmol) dissolved in DCM (0.5 mL) and Cs_2_CO_3_ (195 mg, 0.9 mmol). The mixture was stirred at 0 °C for 5 h and then was diluted with CH_2_Cl_2_ and washed with saturated NH_4_Cl (aq). The organic layers were dried over MgSO_4_ and filtered, and the solvents were evaporated in vacuo. The residue was purified by flash column chromatography, eluting with petroleum ether and ethyl acetate to afford the corresponding product **5**.

*(S,E)-(3-(benzyloxy)prop-1-ene-1,3-diyl)dibenzene* (**5a**) [44]: Colorless oil. 99% yield and 98% ee, determined by chiral HPLC analysis (Chiralcel OD-H hexane/isopropanol, 99:1 *v*/*v*, 0.5 mL/min, 25 °C, UV 254 nm), Retention times: t_r_ = 12.2 min for (*S*)-isomer (major), t_r_ = 13.4 min for (*R*)-isomer (minor). ^1^H-NMR (500 MHz, CDCl_3_) δ 7.46–7.30 (m, 14H), 7.27–7.23 (m, 1H), 6.66 (d, *J* = 15.9 Hz, 1H), 6.38 (dd, *J* = 15.9, 7.0 Hz, 1H), 5.04 (d, *J* = 7.0 Hz, 1H), 4.62-4.57 (m, 2H); ^13^C-NMR (125 MHz, CDCl_3_) δ 141.0, 138.2, 136.5, 131.4, 130.2, 129.6, 128.4, 128.3, 127.6, 127.4, 126.9, 126.5, 81.5, 70.0.

*(S,E)-(3-((4-methoxybenzyl)oxy)prop-1-ene-1,3-diyl)dibenzene* (**5b**) [44]: Colorless oil. 97% yield and 97% ee, determined by chiral HPLC analysis (Chiralcel OD-H hexane/isopropanol, 99:1 *v*/*v*, 0.5 mL/min, 25 °C, UV 254 nm), Retention times: t_r_ = 18.0 min for (*S*)-isomer (major), t_r_ = 23.8 min for (*R*)-isomer (minor). ^1^H-NMR (500 MHz, CDCl_3_) δ 7.44 (d, *J* = 7.6 Hz, 2H), 7.38 (t, *J* = 7.6 Hz, 4H), 7.32–7.29 (m, 5H), 7.23 (t, *J* = 7.2 Hz, 1H), 6.91 (d, *J* = 8.5 Hz, 2H), 6.63 (d, *J* = 15.9 Hz, 1H), 6.36 (dd, *J* = 15.9, 7.0 Hz, 1H), 5.01 (d, *J* = 7.0 Hz, 1H), 4.51 (s, 2H), 3.82 (s, 3H); ^13^C-NMR (125 MHz, CDCl_3_) δ 159.1, 141.2, 136.6, 131.4, 130.4, 130.3, 129.3, 128.5, 127.6, 126.9, 126.5, 113.7, 81.2, 69.7, 55.2.

*(S,E)-(3-((4-bromobenzyl)oxy)prop-1-ene-1,3-diyl)dibenzene* (**5c**) [44]: Colorless oil. 93% yield and 91% ee, determined by chiral HPLC analysis (Chiralcel OD-H hexane/isopropanol, 95:5 *v*/*v*, 0.5 mL/min, 25 °C, UV 254 nm), Retention times: t_r_ = 10.2 min for (*S*)-isomer (major), t_r_ = 11.2 min for (*R*)-isomer (minor). ^1^H-NMR (500 MHz, CDCl_3_) δ 7.47 (d, *J* = 8.3 Hz, 2H), 7.42-7.36 (m, 6H), 7.31–7.27 (m, 3H), 7.25–7.23 (m, 3H), 6.61 (d, *J* = 15.9 Hz, 1H), 6.32 (dd, *J* = 15.9, 7.1 Hz, 1H), 4.97 (d, *J* = 7.1 Hz, 1H), 4.55–4.48 (m, 2H); ^13^C-NMR (125 MHz, CDCl_3_) δ 140.9, 137.5, 136.5, 131.8, 131.5, 130.1, 129.4, 128.7, 128.6, 127.9, 127.0, 126.7, 121.4, 81.9, 69.4.

*(S,E)-(3-((3-bromobenzyl)oxy)prop-1-ene-1,3-diyl)dibenzene* (**5d**) [45]: Colorless oil. 95% yield and 94% ee, determined by chiral HPLC analysis (Chiralcel OD-H hexane/isopropanol, 99:1 *v*/*v*, 0.5 mL/min, 25 °C, UV 254 nm), Retention times: t_r_ = 14.3 min for (*S*)-isomer (major), t_r_ = 15.9 min for (*R*)-isomer (minor). ^1^H-NMR (500 MHz, CDCl_3_) δ 7.54 (s, 1H), 7.44–7.39 (m, 7H), 7.33–7.29 (m, 4H), 7.26–7.21 (m, 2H), 6.65 (d, *J* = 15.9 Hz, 1H), 6.36 (dd, *J* = 15.9, 7.1 Hz, 1H), 5.02 (d, *J* = 7.0 Hz, 1H), 4.58 (q, *J* = 12.2 Hz, 2H); ^13^C-NMR (125 MHz, CDCl_3_) δ 140.9, 136.5, 131.8, 130.6, 130.0, 128.6, 127.9, 127.0, 126.7, 126.1, 122.6, 82.1, 69.4.

*(S,E)-(3-((2-bromobenzyl)oxy)prop-1-ene-1,3-diyl)dibenzene* (**5e**) [46]: Colorless oil. 93% yield and 93% ee, determined by chiral HPLC analysis (Chiralcel OJ-H hexane/isopropanol, 99:1 *v*/*v*, 0.3 mL/min, 25 °C, UV 254 nm), Retention times: t_r_ = 73.1 min for (*S*)-isomer (major), t_r_ = 81.9 min for (*R*)-isomer (minor). ^1^H-NMR (500 MHz, CDCl_3_) δ 7.69 (d, *J* = 7.7 Hz, 1H), 7.64 (d, *J* = 7.9 Hz, 1H), 7.57–7.47 (m, 6H), 7.45–7.39 (m, 4H), 7.35–7.24 (m, 2H), 6.80 (d, *J* = 15.9 Hz, 1H), 6.47 (dd, *J* = 15.9, 7.0 Hz, 1H), 5.17 (d, *J* = 7.1 Hz, 1H), 4.76 (q, *J* = 13.1 Hz, 2H); ^13^C-NMR (125 MHz, CDCl_3_) δ 141.0, 137.9, 136.6, 132.9, 132.5, 131.8, 130.0, 129.2, 128.9, 128.6, 127.8, 127.4, 126.9, 126.7, 82.4, 69.8.

*(S,E)-2-(((1,3-diphenylallyl)oxy)methyl)naphthalene* (**5f**) [40]: Colorless oil. 97% yield and 94% ee, determined by chiral HPLC analysis (Chiralcel OD-H hexane/isopropanol, 98:2 *v*/*v*, 1.0 mL/min, 25 °C, UV 254 nm), Retention times: t_r_ = 7.3 min for (*S*)-isomer (major), t_r_ = 10.2 min for (*R*)-isomer (minor). ^1^H-NMR (500 MHz, CDCl_3_) δ 7.84–7.79 (m, 4H), 7.51–7.45 (m, 5H), 7.40–7.37 (m, 4H), 7.32–7.28 (m, 3H), 7.23 (t, *J* = 7.3 Hz, 1H), 6.66 (d, *J* = 15.9 Hz, 1H), 6.39 (dd, *J* = 15.9, 7.0 Hz, 1H), 5.06 (d, *J* = 7.0 Hz, 1H), 4.74 (s, 2H); ^13^C-NMR (125 MHz, CDCl_3_) δ 141.2, 136.7, 136.0, 133.4, 133.1, 131.7, 130.3, 128.6, 128.2, 128.0, 127.8, 127.7, 127.1, 126.7, 126.5, 126.1, 125.9, 125.8, 81.7, 70.3.

*(S,E)-2-(((1,3-diphenylallyl)oxy)methyl)pyridine* (**5g**) [44]: Colorless oil. 86% yield and 94% ee, determined by chiral HPLC analysis (Chiralcel OD-H hexane/isopropanol, 99:1 *v*/*v*, 0.8 mL/min, 25 °C, UV 254 nm), Retention times: t_r_ = 25.0 min for (*S*)-isomer (major), t_r_ = 36.8 min for (*R*)-isomer (minor). ^1^H-NMR (500 MHz, CDCl_3_) δ 8.56 (d, *J* = 4.6 Hz, 1H), 7.74–7.71 (m, 1H), 7.58 (d, *J* = 7.8 Hz, 1H), 7.49 (d, *J* = 7.4 Hz, 2H), 7.40 (t, *J* = 7.4 Hz, 4H), 7.32 (t, *J* = 7.6 Hz, 3H), 7.25 (t, *J* = 7.3 Hz, 1H), 7.21–7.19 (m, 1H), 6.72 (d, *J* = 15.8 Hz, 1H), 6.40 (dd, *J* = 15.8, 7.1 Hz, 1H), 5.11 (d, *J* = 7.1 Hz, 1H), 4.78 (dd, *J* = 34.2, 13.5 Hz, 2H); ^13^C-NMR (125 MHz, CDCl_3_) δ 158.7, 148.9, 140.7, 136.4, 131.7, 129.8, 128.4, 127.7, 126.8, 126.5, 122.1, 121.2, 82.5, 71.1.

*(S,E)-(3-ethoxyprop-1-ene-1,3-diyl)dibenzene* (**5h**) [44]: Colorless oil. 98% yield and 99% ee, determined by chiral HPLC analysis (Chiralcel OD-H hexane/isopropanol, 99:1 *v*/*v*, 0.5 mL/min, 25 °C, UV 254 nm), Retention times: t_r_ = 9.2 min for (*S*)-isomer (major), t_r_ = 10.4 min for (*R*)-isomer (minor). ^1^H-NMR (500 MHz, CDCl_3_) δ 7.42–7.35 (m, 6H), 7.31–7.27 (m, 3H), 7.22 (t, *J* = 7.3 Hz, 1H), 6.63 (d, *J* = 15.9 Hz, 1H), 6.34 (dd, *J* = 15.9, 7.1 Hz, 1H), 4.93 (d, *J* = 7.1 Hz, 1H), 3.61–3.56 (m, 1H), 3.52–3.48 (m, 1H), 1.27 (t, *J* = 7.0 Hz, 3H); ^13^C-NMR (125 MHz, CDCl_3_) δ 141.6, 136.7, 131.2, 130.7, 128.5, 127.7, 127.6, 126.9, 126.6, 82.6, 64.0, 15.4.

*(S,E)-(3-(but-3-en-1-yloxy)prop-1-ene-1,3-diyl)dibenzene* (**5i**) [44]: Colorless oil. 97% yield and 95% ee, determined by chiral HPLC analysis (Chiralcel OD-H hexane/isopropanol, 99.2:0.8 *v*/*v*, 0.4 mL/min, 25 °C, UV 254 nm), Retention times: t_r_ = 10.3 min for (*S*)-isomer (major), t_r_ = 12.1 min for (*R*)-isomer (minor). ^1^H-NMR (500 MHz, CDCl_3_) δ 7.40–7.34 (m, 6H), 7.30–7.26 (m, 3H), 7.22 (t, *J* = 7.4 Hz, 1H), 6.62 (d, *J* = 15.9 Hz, 1H), 6.31 (dd, *J* = 15.9, 7.0 Hz, 1H), 5.90–5.81 (m, 1H), 5.11–5.02 (m, 2H), 4.92 (d, *J* = 7.0 Hz, 1H), 3.60–3.56 (m, 1H), 3.50–3.46 (m, 1H), 2.43 (q, *J* = 6.8 Hz, 2H); ^13^C-NMR (125 MHz, CDCl_3_) δ 141.4, 136.6, 135.3, 131.3, 130.5, 128.5, 127.7, 127.6, 126.8, 126.6, 116.3, 82.7, 68.0, 34.4.

*(S,E)-2-((1,3-diphenylallyl)oxy)-2,3-dihydro-1H-indene* (**5j**) [41]: Colorless oil. 96% yield and 93% ee, determined by chiral HPLC analysis (Chiralcel OD-H hexane/isopropanol, 99:1 *v*/*v*, 0.5 mL/min, 25 °C, UV 254 nm), Retention times: t_r_ = 13.7 min for (*S*)-isomer (major), t_r_ = 14.8 min for (*R*)-isomer (minor). ^1^H-NMR (500 MHz, CDCl_3_) δ 7.45–7.36 (m, 6H), 7.32–7.30 (m, 3H), 7.26–7.15 (m, 5H), 6.65 (d, *J* = 15.9 Hz, 1H), 6.37 (dd, *J* = 15.9, 7.0 Hz, 1H), 5.12 (d, *J* = 6.9 Hz, 1H), 4.55–4.51 (m, 1H), 3.25–3.02 (m, 4H); ^13^C-NMR (125 MHz, CDCl_3_) δ 141.4, 140.8, 136.5, 131.1, 130.6, 128.4, 127.5, 126.8, 126.5, 126.3, 124.5, 80.9, 77.9, 39.5. 

## 4. Conclusions

SMI-PHOX ligand exhibited good catalytic performance in Pd-catalyzed asymmetric allylic alkylation reactions of indoles with 1,3-diaryl-2-propenyl acetate. The reactions afforded the corresponding products with high yields (up to 98%) and enantioselectivities (up to 98% ee) for a broad scope of indole derivatives. SMI-PHOX ligand was determined to be the most efficient P,N-ligand for this reaction. Furthermore, SMI-PHOX ligand was also efficient for Pd-catalyzed asymmetric allylic etherification with hard aliphatic alcohols as nucleophiles. Up to 99% yield and 99% ee were obtained. Further applications of these ligands in other asymmetric transformations are currently under development in our laboratory.

## Figures and Tables

**Table 1:**
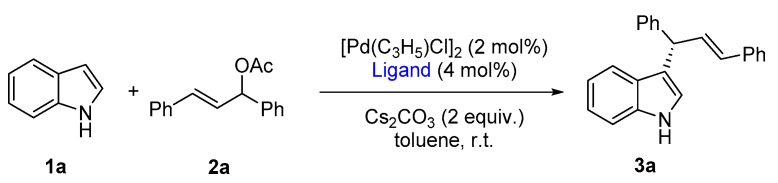
Evaluation of ligands for Pd-catalyzed allylic alkylation of indoles. ^a^

Entry	Ligand	T (°C)	Time (h)	Yield ^b^ (%)	ee ^c,d^ (%)
1	**L1**	25	12	95	96
2	**L2**	25	12	87	91
3	**L3**	25	24	70	80
4	**L4**	25	12	94	87

^a^ Unless otherwise noted, reactions were performed with **1a** (0.3 mmol), **2a** (0.36 mmol), [Pd(C_3_H_5_)Cl]_2_ (2 mol%), Ligand (4 mol%), Cs_2_CO_3_ (0.6 mmol) in toluene (1.5 mL) at room temperature (r.t.). ^b^ Isolated yield. ^c^ The ee values were determined by chiral high-performance liquid chromatography (HPLC) with the *N*-Boc-protected derivative of **3a**. ^d^ The absolute configurations were assigned as *S* via comparison of specific rotations with the literature data [20].

**Table 2:**
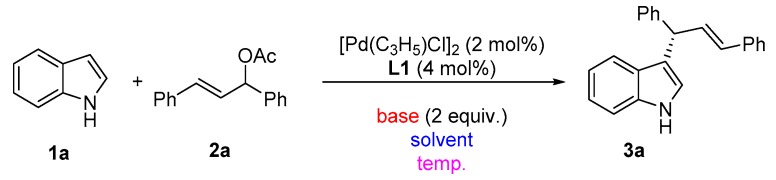
Optimization of reaction conditions for Pd-catalyzed allylic alkylation of indoles. ^a^

Entry	Solvent	Base	Time (h)	T (°C)	Yield ^b^ (%)	ee ^c,d^ (%)
1	toluene	Cs_2_CO_3_	12	25	95	96
2	xylenes	Cs_2_CO_3_	12	25	86	94
3	mesitylene	Cs_2_CO_3_	12	25	84	90
4	CH_2_Cl_2_	Cs_2_CO_3_	12	25	76	83
5	EtOAc	Cs_2_CO_3_	12	25	74	84
6	CH_3_CN	Cs_2_CO_3_	24	25	47	19
7	THF	Cs_2_CO_3_	24	25	49	3
8	DMF	Cs_2_CO_3_	48	25	N.R. ^e^	N.D. ^f^
9	toluene	K_2_CO_3_	12	25	65	89
10	toluene	Na_2_CO_3_	12	25	76	15
11	toluene	Cs_2_CO_3_	12	0	70	90
12	toluene	Cs_2_CO_3_	12	40	84	79
13 ^g^	toluene	Cs_2_CO_3_	12	25	91	94
14 ^h^	toluene	Cs_2_CO_3_	12	25	90	95

^a^ Unless otherwise noted, reactions were performed with **1a** (0.3 mmol), **2a** (0.36 mmol), [Pd(C_3_H_5_)Cl]_2_ (2 mol%), **L1** (4 mol%), base (0.6 mmol) in solvent (1.5 mL) at a certain temperature. ^b^ Isolated yield. ^c^ The ee values were determined by chiral HPLC with the *N*-Boc-protected derivative of **3a**. ^d^ The absolute configurations were assigned as S via comparison of specific rotations with the literature data [20]. ^e^ No reaction. ^f^ Not detected. ^g^ The molar ratio of **1a** to **2a** was 1:1. ^h^ The molar ratio of **1a** to **1b** was 1.2:1.

**Table 3:**
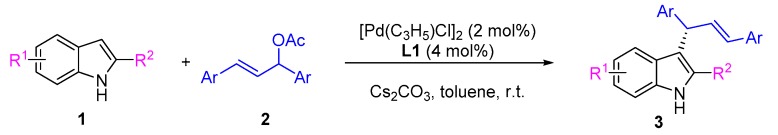
Substrate scope of asymmetric allylic alkylation of indoles. ^a^

Entry	R^1^	R^2^	Ar	Product	Yield ^b^ (%)	Ee ^c,d^ (%)
1	H	H	Ph	**3a**	95	96
2	H	Me	Ph	**3b**	89	96
3	H	Ph	Ph	**3c**	85	86
4	4-Me	H	Ph	**3d**	93	84
5	4-MeO	H	Ph	**3e**	95	92
6	5-Me	H	Ph	**3f**	96	95
7	5-MeO	H	Ph	**3g**	98	98
8	5-BnO	H	Ph	**3h**	97	96
9	5-Cl	H	Ph	3i	95	92
10	5-Br	H	Ph	**3j**	92	93
11	6-Me	H	Ph	3k	90	93
12	6-Cl	H	Ph	**3l**	94	93
13	7-Me	H	Ph	3m	90	83
14	7-MeO	H	Ph	**3n**	86	84
15	7-Cl	H	Ph	**3o**	90	94
16	H	H	4-MePh	**3p**	92	94
17	H	H	4-MeOPh	**3q**	85	90
18	H	H	4-ClPh	**3r**	91	95
19	H	H	4-NO_2_Ph	**3s**	87	97

^a^ Unless otherwise noted, reactions were performed with **1** (0.3 mmol), **2** (0.36 mmol), [Pd(C_3_H_5_)Cl]_2_ (2 mol%), **L1** (4 mol%), Cs_2_CO_3_ (0.6 mmol) in toluene (1.5 mL) at room temperature. ^b^ Isolated yield. ^c^ The ee values were determined by chiral HPLC with the *N*-Boc-protected derivative of **3**. ^d^ The absolute configurations were assigned as S via comparison of specific rotations with the literature data [20].

**Table 4:**
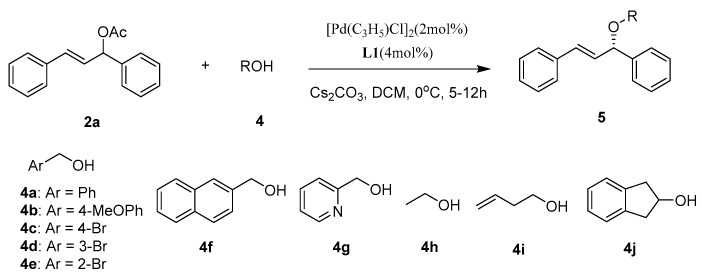
Substrate scope of asymmetric allylic etherification. ^a^

Entry	4	Product	Yield ^b^ (%)	ee ^c,d^ (%)
1	**4a**	**5a**	99	98
2	**4b**	**5b**	97	97
3	**4c**	**5c**	93	91
4	**4d**	**5d**	95	94
5	**4e**	**5e**	93	93
6	**4f**	**5f**	97	94
7	**4g**	**5g**	86	94
8	**4h**	**5h**	98	99
9	**4i**	**5i**	97	95
10	**4j**	**5j**	96	93

^a^ Unless otherwise noted, reactions were performed with **2a** (0.3 mmol), **4** (0.9 mmol), [Pd(C_3_H_5_)Cl]_2_ (2 mol%), **L1** (4 mol%), Cs_2_CO_3_ (0.9 mmol) in DCM (2 mL) at 0^o^C. ^b^ Isolated yield. ^c^ Determined by HPLC using a chiral column. ^d^ The absolute configurations were assigned as S via comparison of specific rotations with the literature data [42].

## Supplementary Materials

The supplementary materials including NMR spectra and HPLC traces of the compounds are available online.

## References

[1] Scholz U., Winterfeldt E. (2000). Biomimetic synthesis of alkaloids. Nat. Prod. Rep..

[2] O’Connor S.E., Maresh J.J. (2006). Chemistry and biology of monoterpene indole alkaloid biosynthesis. Nat. Prod. Rep..

[3] Kochanowska-Karamyan A.J., Hamann M.T. (2010). Marin indole alkaloids: Potential new drug leads for the control of depression and anxiety. Chem. Rev..

[4] Rios I.G., Rosas-Hernandez A., Martin E. (2011). Recent advances in the application of chiral phosphine ligands in Pd-catalysed asymmetric allylic alkylation. Molecules.

[5] Bariwal J., Voskressensky L.G., Van der Eycken E.V. (2018). Recent advances in spirocyclization of indole derivatives. Chem. Soc. Rev..

[6] Dalpozzo R. (2015). Strategies for the asymmetric functionalization of indoles: An update. Chem. Soc. Rev..

[7] Jia Y.X., Xie J.H., Duan H.F., Wang L.X., Zhou Q.L. (2006). Asymmetric Friedel-Crafts addition of indoles to *N*-sulfonyl aldimines: A simple approach to optically active 3-indolyl-methanamine derivatives. Org. Lett..

[8] Yamazaki S., Iwata Y. (2006). Catalytic enantioselective Friedel-Crafts/Michael addition reactions of indoles to ethenetricarboxylates. J. Org. Chem..

[9] Yadav J.S., Reddy B.V.S., Praneeth K. (2008). FeCl_3_-catalyzed alkylation of indoles with 1,3-dicarbonyl compounds: An expedient synthesis of 3-substitued indoles. Tetrahedron Lett..

[10] Srivastava A., Yadav A., Samanta S. (2015). Biopolymeric alginic acid: An efficient recyclable green catalyst for the Friedel-Crafts reaction of indoles with isoquinoline-1,3,4-triones in water. Tetrahedron Lett..

[11] Kirsch J.K., Manske J.L., Wolfe J.P. (2018). Pd-catalyzed alkene carboheteroarylation reactions for the synthesis of 3-cyclopentylindole derivatives. J. Org. Chem..

[12] Panda S., Ready J.M. (2018). Tandem allylation/1,2-boronate rearrangement for the asymmetric synthesis of indolines with adjacent quaternary stereocenters. J. Am. Chem. Soc..

[13] Pritchett B.P., Stoltz B.M. (2018). Enantioselective palladium-catalyzed allylic alkylation reactions in the synthesis of *Aspidosperma* and structurally related monoterpene indole alkaloids. Nat. Prod. Rep..

[14] Chen J.B., Jia Y.X. (2017). Recent progress in transition-metal-catalyzed enantioselective indole functionalization. Org. Biomol. Chem..

[15] Cheung H.Y., Yu W.Y., Lam F.L., Au-Yeung T.T.L., Zhou X.Y., Chan T.H., Chan A.S.C. (2007). Enantioselective Pd-catalyzed allylic alkylation of indoles by a new class of chiral ferrocenyl P/S ligands. Org. Lett..

[16] Hossi T., Sasaki K., Sato S., Ishii Y., Suzuki T., Hagiwara H. (2011). Highly enantioselective Pd-catalyzed allylic alkylation of indoles using sulfur-MOP ligand. Org. Lett..

[17] Mino T., Ishikawa M., Nishikawa K., Wakui K., Sakamoto M. (2013). Palladium-catalyzed asymmetric allylic alkylation of indoles by C–N bond axially chiral phosphine ligands. Tetrahedron Asymmetry.

[18] Feng B., Pu X.Y., Liu Z.C., Xiao W.J., Chen J.R. (2016). Highly enantioselective Pd-catalyzed indole allylic alkylation using binaphthyl-based phosphoramidite-thioether ligands. Org. Chem. Front..

[19] Liu Z.Q., Cao Z.P., Du H.F. (2011). Highly effective chiral phosphorus amidite–olefin ligands for palladium-catalyzed asymmetric allylic substitutions. Org. Biomol. Chem..

[20] Cao Z.P., Liu Y.L., Liu Z.Q., Feng X.Q., Zhuang M.Y., Du H.F. (2011). Pd-catalyzed asymmetric allylic alkylation of indoles and pyrroles by chiral alkene-phosphine ligands. Org. Lett..

[21] Mino T., Nishikawa K., Asano M., Shima Y., Ebisawa T., Yoshida T., Yoshida Y., Sakamoto M. (2016). Chiral *N*-1-adamantyl-*N*-*trans*-cinnamylaniline type ligands: synthesis and application to palladium-catalyzed asymmetric allylic alkylation of indoles. Org. Biomol. Chem..

[22] Yamamoto K., Shimizu T., Igawa K., Tomooka K., Hirai G., Suemune H., Usui K. (2016). Rational design and synthesis of [5]helicene-derived phosphine ligands and their application in Pd-catalyzed asymmetric reactions. Sci. Rep..

[23] Zhang Z.F., Butt N.A., Zhang W.B. (2016). Asymmetric hydrogenation of nonaromatic cyclic substrates. Chem. Rev..

[24] Pellissier H. (2016). Enantioselective silver-catalyzed transformations. Chem. Rev..

[25] Zhu S.F., Xie J.B., Zhang Y.Z., Li S., Zhou Q.L. (2006). Well-defined chiral spiro iridium/phosphine-oxazoline cationic complexes for highly enantioselective hydrogenation of imines at ambient pressure. J. Am. Chem. Soc..

[26] Li S., Zhu S.F., Zhang C.M., Song S., Zhou Q.L. (2008). Iridium-catalyzed enantioselective hydrogenation of α,β-unsaturated carboxylic acids. J. Am. Chem. Soc..

[27] Li S., Zhu S.F., Xie J.H., Song S., Zhang C.M., Zhou Q.L. (2010). Enantioselective hydrogenation of α-aryloxy and α-alkoxy α,β-unsaturated carboxylic acids catalyzed by chiral spiro iridium/phosphino-oxazoline complexes. J. Am. Chem. Soc..

[28] Song S., Zhu S.F., Yang S., Li S., Zhou Q.L. (2012). Enantioselective iridium-catalyzed hydrogenation of β,γ-unsaturated carboxylic acids: an efficient approach to chiral 4-alkyl-4-aryl butanoic acids. Angew. Chem. Int. Ed..

[29] Song S., Zhu S.F., Pu L.Y., Zhou Q.L. (2013). Iridium-catalyzed enantioselective hydrogenation of unsaturated heterocyclic acids. Angew. Chem. Int. Ed..

[30] Song S., Zhu S.F., Yu Y.B., Zhou Q.L. (2013). Carboxy-directed asymmetric hydrogenation of 1,1-diarylethenes and 1,1-dialkylethenes. Angew. Chem. Int. Ed..

[31] Han Z.B., Wang Z., Zhang X.M., Ding K.L. (2009). Spiro[4,4]-1,6-nonadiene-based phosphine–oxazoline ligands for iridium-catalyzed enantioselective hydrogenation of ketimines. Angew. Chem. Int. Ed..

[32] Zhang Y., Han Z.B., Li F.Y., Ding K.L., Zhang A. (2010). Highly enantioselective hydrogenation of α-aryl-β-substituted acrylic acids catalyzed by Ir-SpinPHOX. Chem. Commun..

[33] Shang J., Han Z.B., Li Y., Wang Z., Ding K.L. (2012). Highly enantioselective asymmetric hydrogenation of (*E*)-β,β-disubstituted α,β-unsaturated Weinreb amides catalyzed by Ir(I) complexes of SpinPhox ligands. Chem. Commun..

[34] Wang X.M., Han Z.B., Wang Z., Ding K.L. (2012). Catalytic asymmetric synthesis of aromatic spiroketals by SpinPhox/iridium(I)-catalyzed hydrogenation and spiroketalization of α,α′-bis(2-hydroxyarylidene) ketones. Angew. Chem. Int. Ed..

[35] Sun W.Y., Gu H.R., Lin X.F. (2018). Synthesis and application of hexamethyl-1,1′-spirobiindane-based phosphine-oxazoline ligands in Ni-catalyzed asymmetric arylation of cyclic aldimines. J. Org. Chem..

[36] Qiu Z.X., Sun R., Teng D.W. (2018). Synthesis of highly rigid phosphine-oxazoline ligands for palladium-catalyzed asymmetric allylic alkylation. Org. Biomol. Chem..

[37] Gao Y.F., Qiu Z.X., Sun R., Teng D.W. (2018). New spiro phophinooxazolines for palladium-catalyzed asymmetric allylic amination. Tetrahedron Lett..

[38] Lam F.L., Au-Yeung T.T.L., Kwong F.Y., Zhou Z.Y., Wong K.Y., Chan A.S.C. (2008). Palladium-(*S*,_p_R)-ferroNPS-catalyzed asymmetric allylic etherification: electronic effect of nonconjugated substituents on benzylic alcohols on enantioselectivity. Angew. Chem. Int. Ed..

[39] Ye F., Zheng Z.J., Li L., Yang K.F., Xia C.G., Xu L.W. (2013). Development of a novel multifunctional N,P ligand for highly enantioselective palladium-catalyzed asymmetric allylic etherification of alcohols and silanols. Chem. Eur. J..

[40] Feng B., Cheng H.G., Chen J.R., Deng Q.H., Lu L.Q., Xiao W.J. (2014). Palladium/sulfoxide-phosphine-catalyzed highly enantioselective allylic etherification and amination. Chem. Commun..

[41] Khan A., Khan S., Khan I., Zhao C., Mao Y.X., Chen Y., Zhang Y.J. (2017). Enantioselective construction of tertiary C-O bond via allylic substitution of vinylethylene carbonates with water and alcohols. J. Am. Chem. Soc..

[42] Magre M., Biosca M., Norrby P.O., Pàmies O., Diéguez M. (2015). Theoretical and experimental optimization of a new amino phosphite ligand library for asymmetric palladium-catalyzed allylic substitution. ChemCatChem.

[43] Fan G.P., Liu Z., Wang G.W. (2013). Efficient ZnBr_2_-catalyzed reactions of allylic alcohols with indoles, sulfamides and anilines under high-speed vibration miling conditions. Green Chem..

[44] Liu Q.L., Chen W.F., Jiang Q.Y., Bai X.F., Li Z.F., Xu Z., Xu L.W. (2016). A d-camphor-based Schiff base as a highly efficient N,P ligands for enantioselective palladium-catalyzed allylic substitutions. ChemCatChem.

[45] Xing J.W., Cao P., Liao J. (2012). Chiral SO/P hybrid ligands: An enantioseletive switch in palladium-catalyzed asymmetric allylic etherifications. Tetrahedron: Asymmetry.

[46] Du L., Cao P., Liao J. (2013). Bifunctional ligand promoted Pd-catalyzed asymmetric allylic etherification/amination. Acta Chim. Sinica.

