# Supplementary material for: Spiro Indane-Based Phosphine-Oxazolines as Highly Efficient P,N Ligands for Enantioselective Pd-Catalyzed Allylic Alkylation of Indoles and Allylic Etherification
Source: Molecules. 2019 Apr 21;24(8):1575. doi: 10.3390/molecules24081575 (PMC6515030; doi:10.3390/molecules24081575)
Supplement: Supplementary file 1 [file molecules-24-01575-s001.pdf]

Supplementary Materials: Spiro indane-based phosphine-oxazolines as highly efficient P,N ligands for enantioselective Pd-catalyzed allylic alkylation of indoles and allylic etherification

Zhongxuan Qiu, Rui Sun, Kun Yang and Dawei Teng *

Table of Contents

1. **Table S1** Solvent screening for Pd-catalyzed allylic etherification S2

2. NMR spectra of compounds S3-S98

3. HPLC traces of compounds S99-S127

Table S1 Solvent screening for Pd-catalyzed allylic etherification [a].

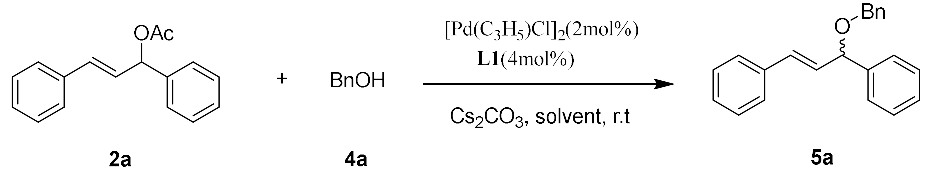

Entry	Solvent	Time (h)	Yield[b] (%)	ee[c] (%)
1	toluene	2	95	93
2	THF	12	92	93
3	DCM	2	99	96
4	EtOAc	12	76	85
5	CH_3CN	24	36	52

[a] Reaction conditions: **2a**(0.3 mmol), **4a** (0.9 mmol), $[Pd(C_3H_5)Cl]_2$ (2 mol%), **L1** (4 mol%), Cs_2CO_3 (0.9 mmol) in a certain solvent (2 mL) at room temperature. [b] Isolated yield. [c] Determined by HPLC using a chiral OD-H column.

Copies of NMR spectra

(*S*,*E*)-3-(1,3-diphenylallyl)-1H-indole **(3a)**

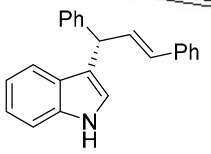

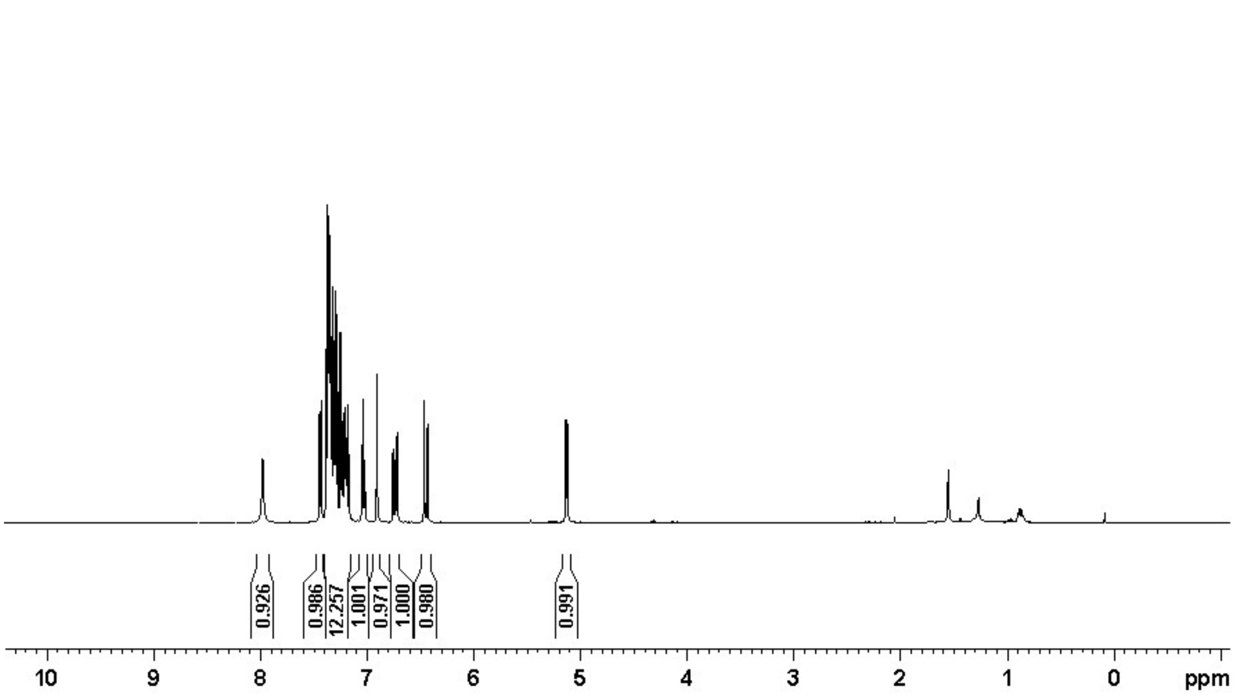


```
NAME          QZX-4-39-4
EXPNO                  1
PROCNO                 1
Date_           20180319
Time               16.05
INSTRUM            spect
PROBHD   5 mm PABBO BB-
PULPROG             zg30
TD                 16384
SOLVENT            CDCl3
NS                     8
DS                     0
SWH            10000.000 Hz
FIDRES          0.610352 Hz
AQ             0.8193000 sec
RG                   322
DW                50.000 usec
DE                  8.00 usec
TE                 293.3 K
D1            2.00000000 sec
TD0                    1

======== CHANNEL f1 ========
NUC1                  1H
P1                 13.00 usec
PL1                 2.00 dB
SFO1         500.0335000 MHz
SI                 16384
SF           500.0300132 MHz
WDW                   EM
SSB                    0
LB                  0.30 Hz
GB                     0
PC                  1.00
```

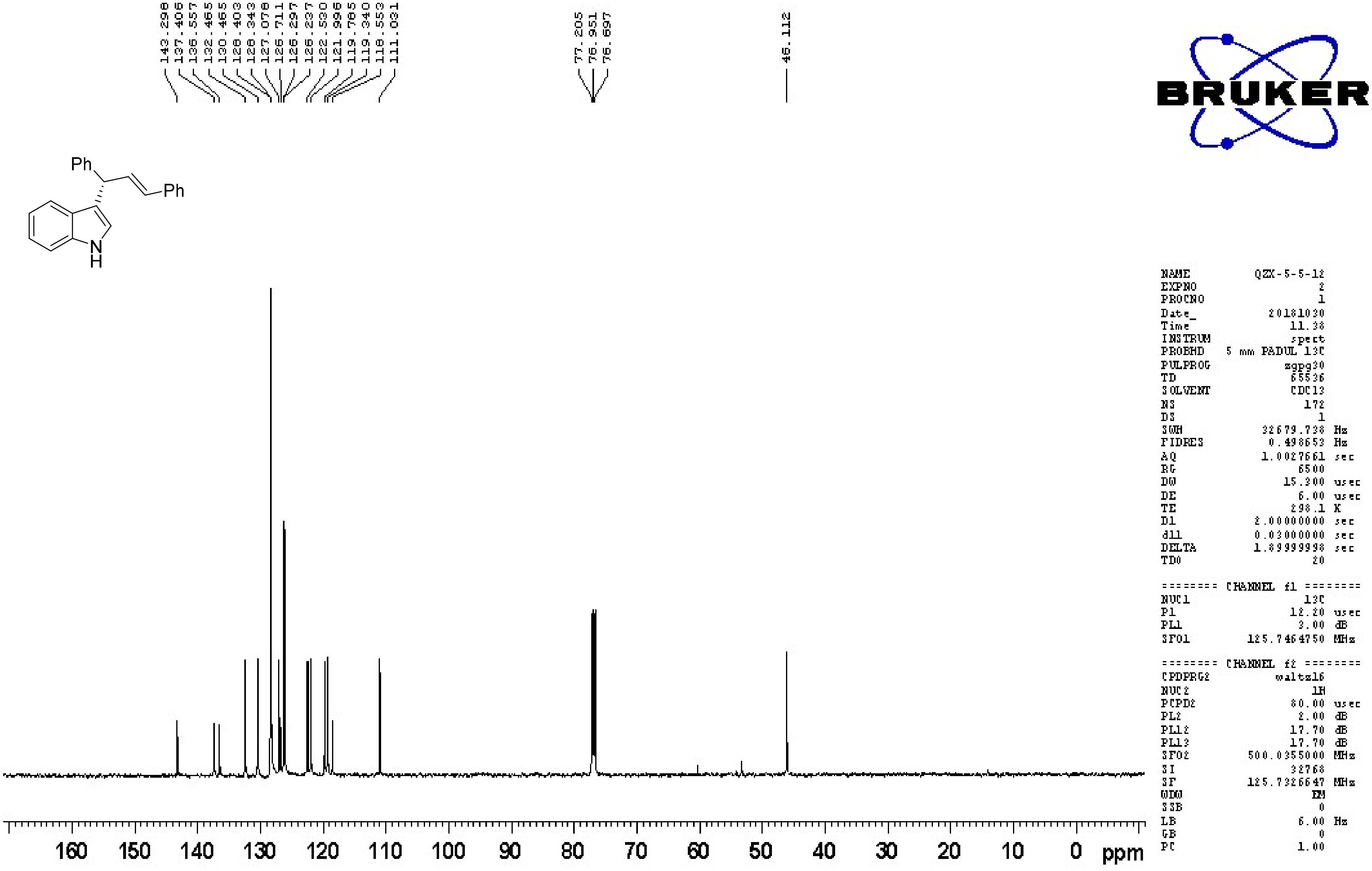
Ph
Ph
N
H
143.298
137.406
136.557
132.465
130.465
128.403
128.343
127.078
126.711
126.297
126.237
122.530
121.996
119.765
119.340
118.553
111.031
77.205
76.951
76.697
46.112
BRUKER
160 150 140 130 120 110 100 90 80 70 60 50 40 30 20 10 0 ppm
NAME QZX-5-5-12
EXPNO 2
PROCNO 1
Date_ 20181030
Time 11.38
INSTRUM spect
PROBHD 5 mm PADUL 13C
PULPROG zgpg30
TD 65536
SOLVENT CDCl3
NS 172
DS 1
SWH 32679.738 Hz
FIDRES 0.498653 Hz
AQ 1.0027661 sec
RG 6500
DW 15.300 usec
DE 6.00 usec
TE 298.1 K
D1 2.00000000 sec
d11 0.03000000 sec
DELTA 1.89999998 sec
TD0 20
======== CHANNEL f1 ========
NUC1 13C
P1 12.20 usec
PL1 3.00 dB
SFO1 125.7464750 MHz
======== CHANNEL f2 ========
CPDPRG2 waltz16
NUC2 1H
PCPD2 80.00 usec
PL2 2.00 dB
PL12 17.70 dB
PL13 17.70 dB
SFO2 500.0355000 MHz
SI 32768
SF 125.7326647 MHz
WDW EM
SSB 0
LB 6.00 Hz
GB 0
PC 1.00

(*S*,*E*)-3-(1,3-diphenylallyl)-2-methyl-1H-indole **(3b)**

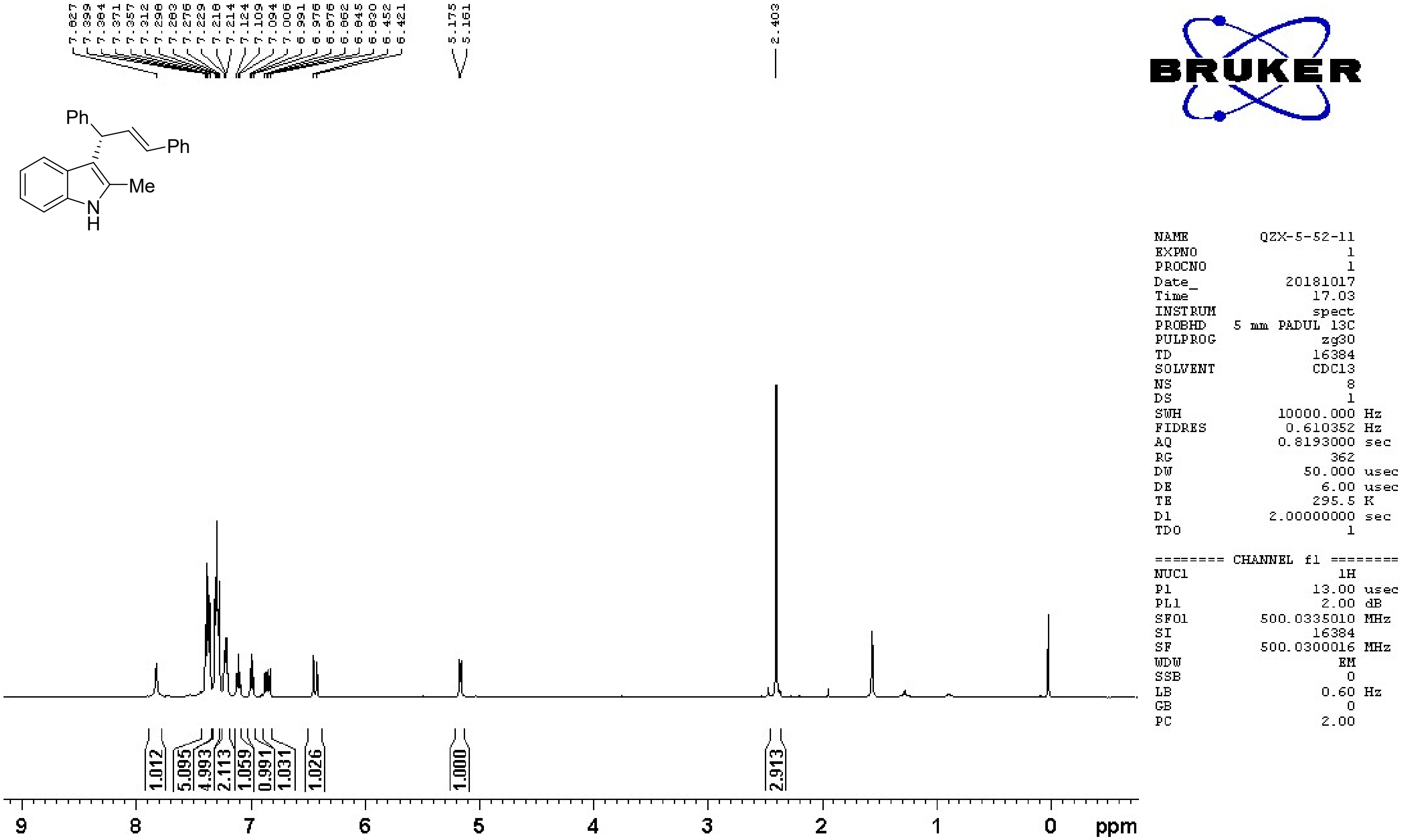

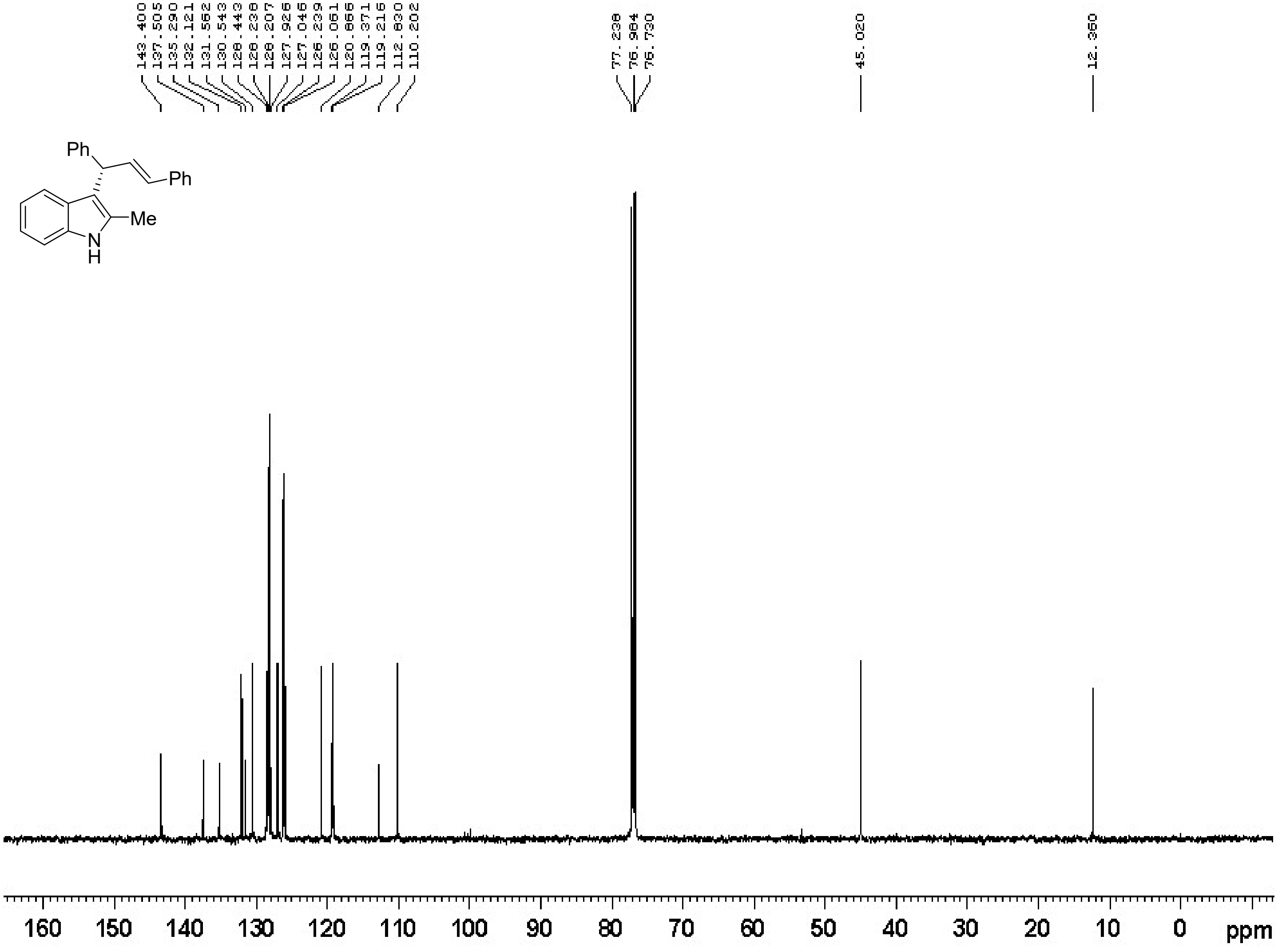

NAME	qzx-5-52-12	
EXPNO	2	
PROCNO	1	
Date_	20181017	
Time	17.32	
INSTRUM	spect	
PROBHD	5 mm PADUL 13C	
PULPROG	zgpg30	
TD	65536	
SOLVENT	CDCl3	
NS	369	
DS	4	
SWH	30030.029	Hz
FIDRES	0.458222	Hz
AQ	1.0912410	sec
RG	128	
DW	16.650	usec
DE	8.00	usec
TE	297.1	K
D1	2.00000000	sec
d11	0.03000000	sec
DELTA	1.89999998	sec
TD0	1	

======== CHANNEL f1 ========

NUC1	13C	
P1	12.20	usec
PL1	4.00	dB
SFO1	125.7452168	MHz

======== CHANNEL f2 ========

CPDPRG2	waltz16	
NUC2	1H	
PCPD2	80.00	usec
PL2	2.00	dB
PL12	18.00	dB
PL13	18.00	dB
SFO2	500.0320001	MHz
SI	32768	
SF	125.7326536	MHz
WDW	EM	
SSB	0	
LB	3.00	Hz
GB	0	
PC	1.40	

(*S*,*E*)-3-(1,3-diphenylallyl)-2-phenyl-1H-indole **(3c)**

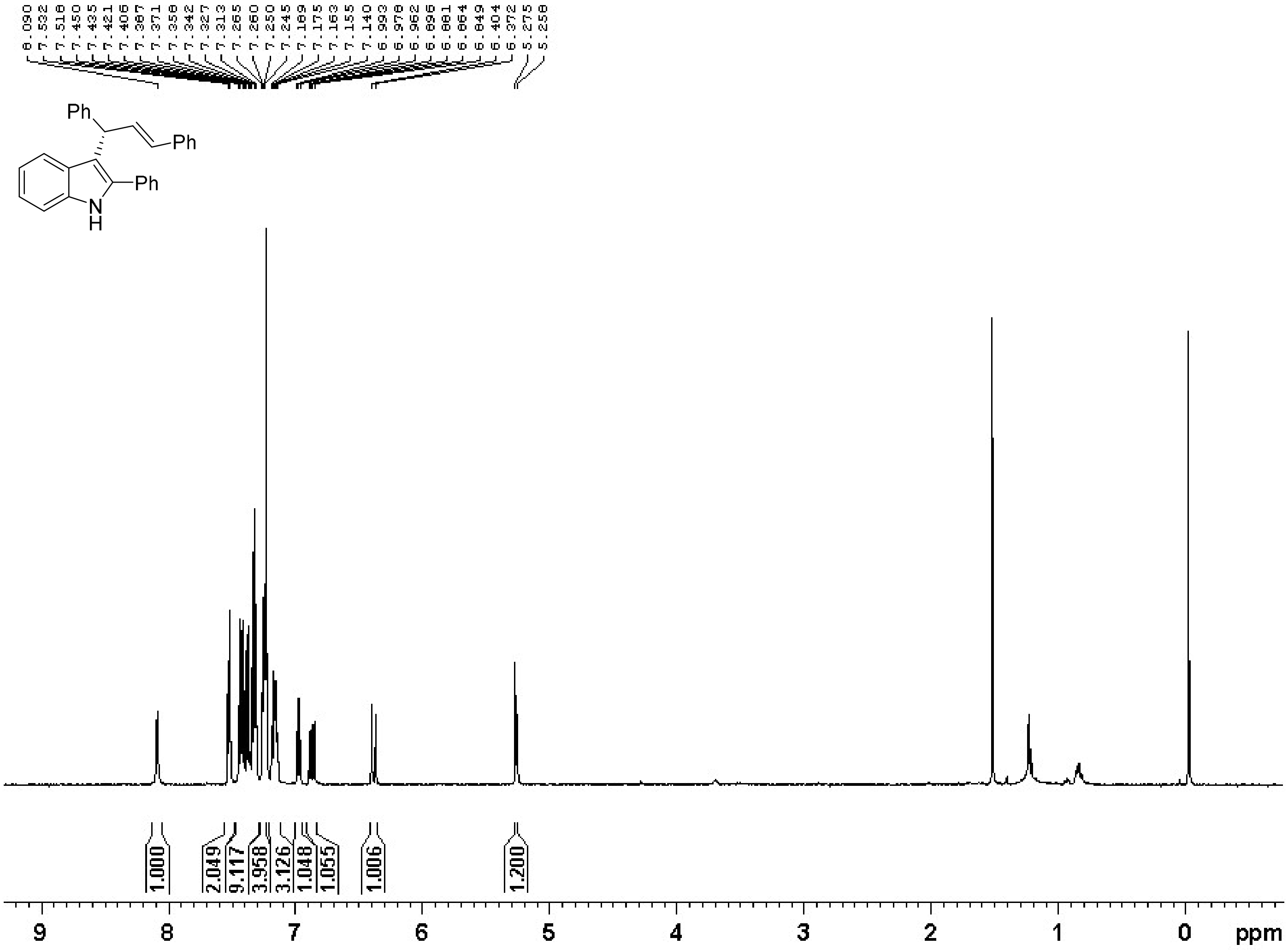

NAME	QZX-5-44-11-1015
EXPNO	1
PROCNO	1
Date_	20181015
Time	16.40
INSTRUM	spect
PROBHD	5 mm PADUL 13C
PULPROG	zg30
TD	16384
SOLVENT	CDCl3
NS	8
DS	0
SWH	10000.000 Hz
FIDRES	0.610352 Hz
AQ	0.8193000 sec
RG	724
DW	50.000 usec
DE	6.00 usec
TE	295.7 K
D1	2.00000000 sec
TD0	1

======== CHANNEL f1 ========	
NUC1	1H
P1	13.00 usec
PL1	2.00 dB
SFO1	500.0335010 MHz
SI	16384
SF	500.0300226 MHz
WDW	EM
SSB	0
LB	0.30 Hz
GB	0
PC	1.00

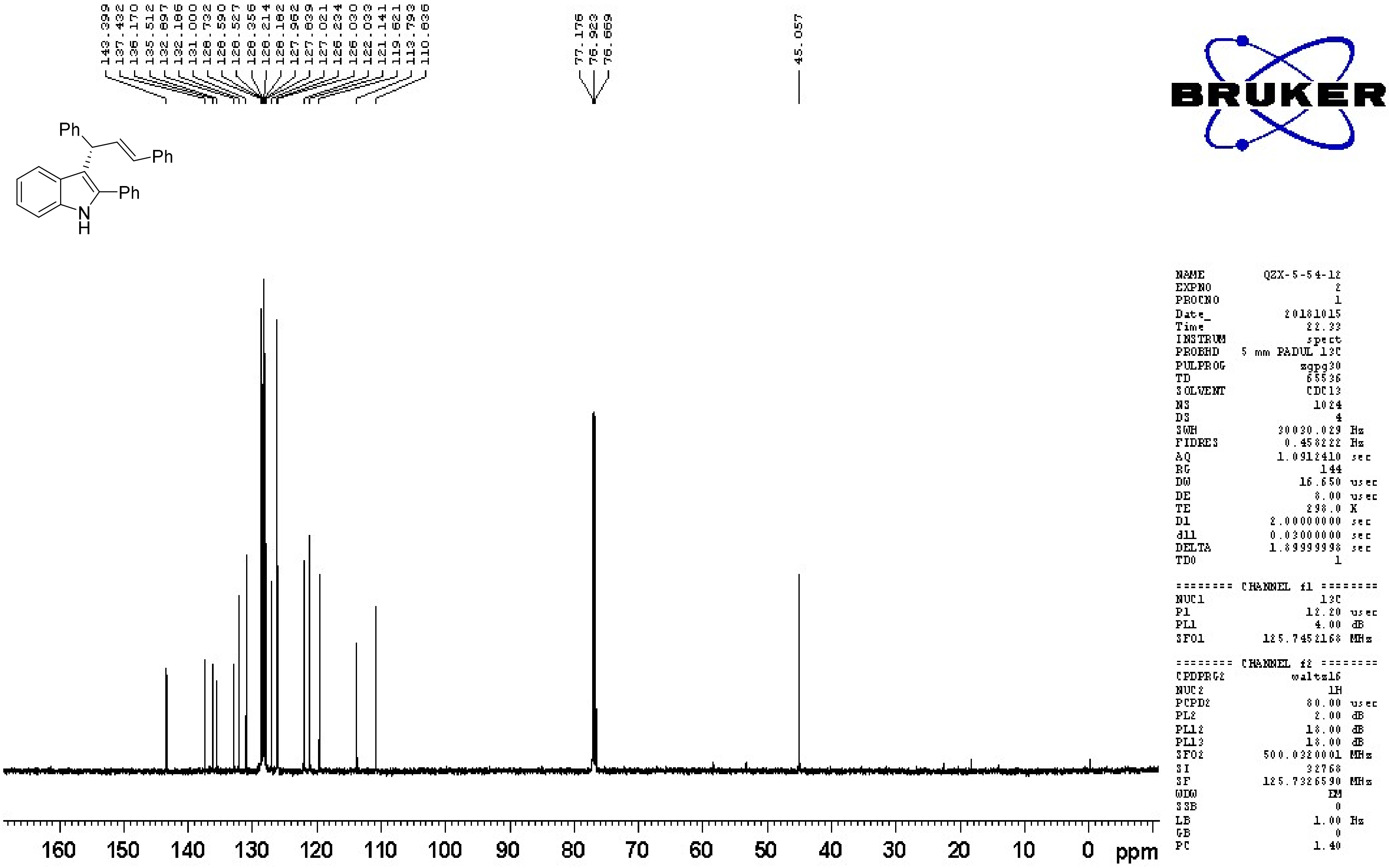
143.399
137.432
136.170
135.512
132.897
132.186
131.000
128.732
128.590
128.527
128.356
128.214
128.182
127.962
127.639
127.021
126.234
126.030
122.033
121.141
119.621
113.793
110.836
77.176
76.923
76.669
45.057
Ph
Ph
Ph
N
H
BRUKER
NAME QZX-5-54-12
EXPNO 2
PROCNO 1
Date_ 20181015
Time 22.33
INSTRUM spect
PROBHD 5 mm PADUL 13C
PULPROG zgpg30
TD 65536
SOLVENT CDCl3
NS 1024
DS 4
SWH 30030.029 Hz
FIDRES 0.458222 Hz
AQ 1.0912410 sec
RG 144
DW 16.650 usec
DE 8.00 usec
TE 298.0 K
D1 2.00000000 sec
d11 0.03000000 sec
DELTA 1.89999998 sec
TD0 1
======== CHANNEL f1 ========
NUC1 13C
P1 12.20 usec
PL1 4.00 dB
SFO1 125.7452168 MHz
======== CHANNEL f2 ========
CPDPRG2 waltz16
NUC2 1H
PCPD2 80.00 usec
PL2 2.00 dB
PL12 18.00 dB
PL13 18.00 dB
SFO2 500.0320001 MHz
SI 32768
SF 125.7326590 MHz
WDW EM
SSB 0
LB 1.00 Hz
GB 0
PC 1.40
160 150 140 130 120 110 100 90 80 70 60 50 40 30 20 10 0 ppm

(*S*,*E*)-3-(1,3-diphenylallyl)-4-methyl-1H-indole **(3d)**

QZX-5-33-11 1H 1D 2018 09 18

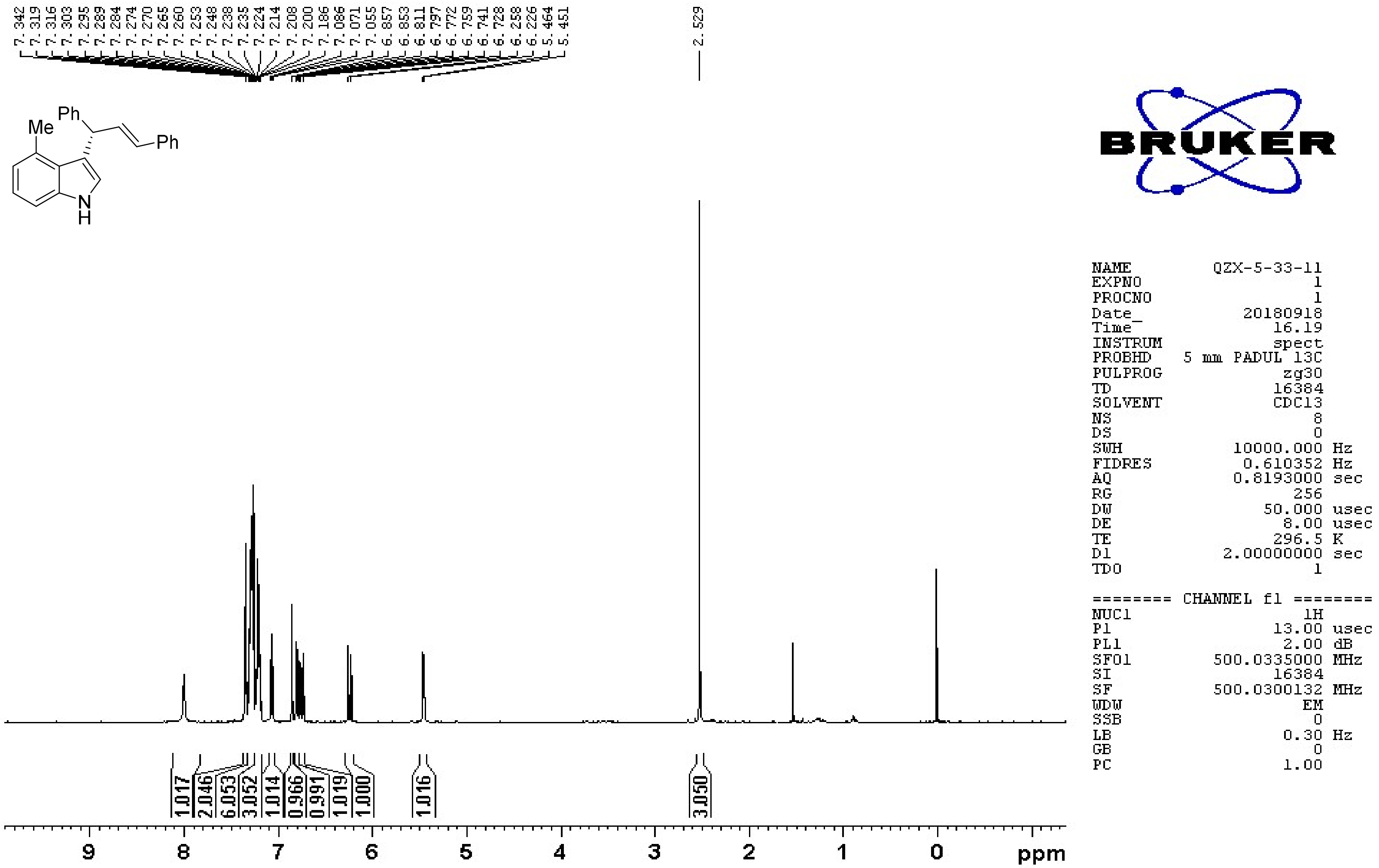

QZX-5-33-12 13C 2018 09 18

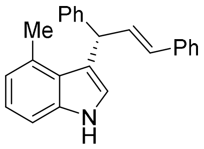

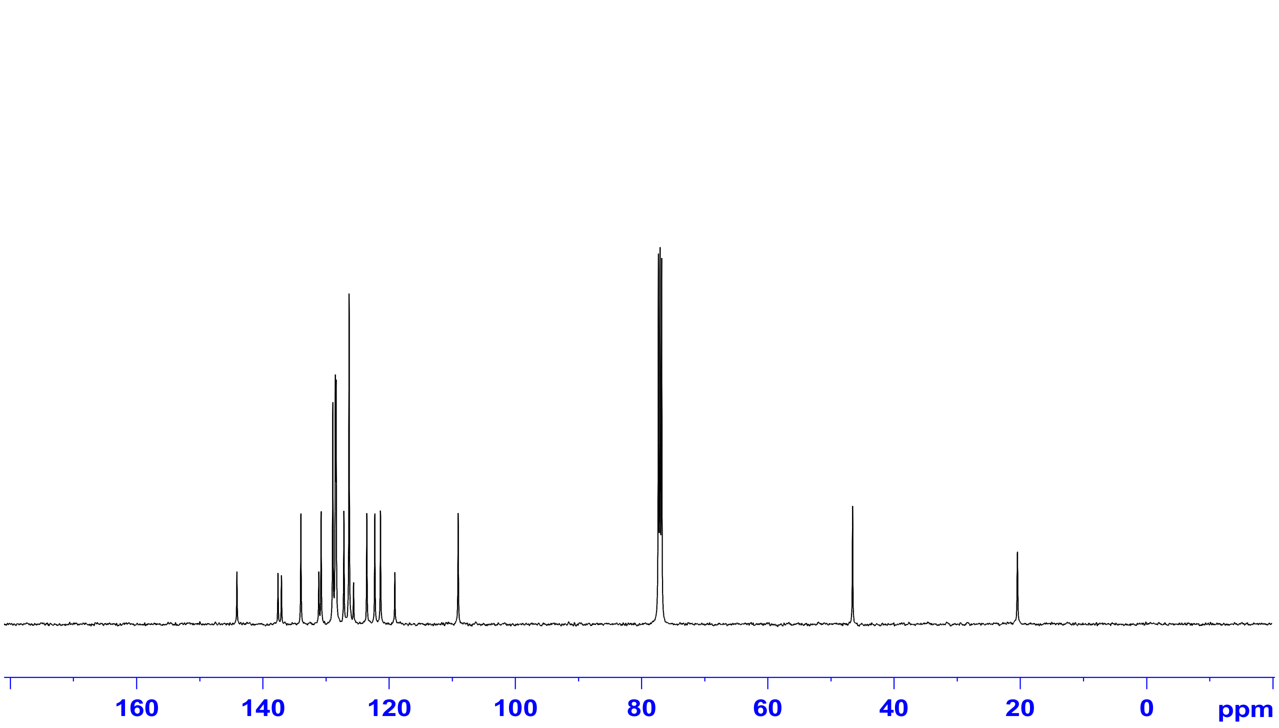

NAME	QZX-5-33-12	
EXPNO	2	
PROCNO	1	
Date_	20180918	
Time	17.46	
INSTRUM	spect	
PROBHD	5 mm PADUL 13C	
PULPROG	zgpg30	
TD	65536	
SOLVENT	CDCl3	
NS	1118	
DS	1	
SWH	32679.738	Hz
FIDRES	0.498653	Hz
AQ	1.0027661	sec
RG	8200	
DW	15.300	usec
DE	6.00	usec
TE	298.8	K
D1	2.00000000	sec
d11	0.03000000	sec
DELTA	1.89999998	sec
TD0	20	
======== CHANNEL f1 ========		
NUC1	13C	
P1	12.20	usec
PL1	3.00	dB
SFO1	125.7464750	MHz
======== CHANNEL f2 ========		
CPDPRG2	waltz16	
NUC2	1H	
PCPD2	80.00	usec
PL2	2.00	dB
PL12	17.70	dB
PL13	17.70	dB
SFO2	500.0355000	MHz
SI	32768	
SF	125.7326392	MHz
WDW	EM	
SSB	0	
LB	10.00	Hz
GB	0	
PC	1.00	

(*S*,*E*)-3-(1,3-diphenylallyl)-4-methoxy-1H-indole **(3e)**

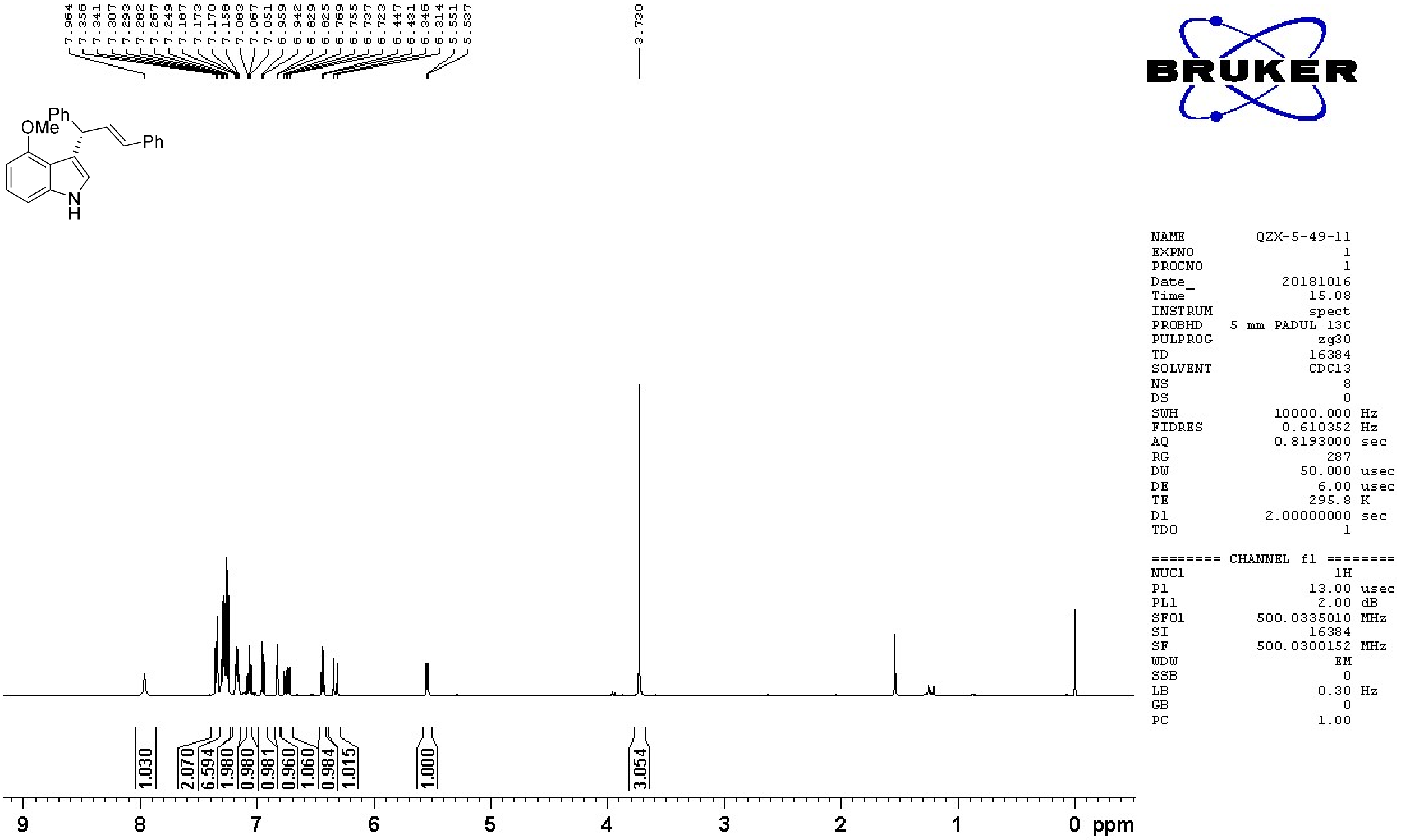

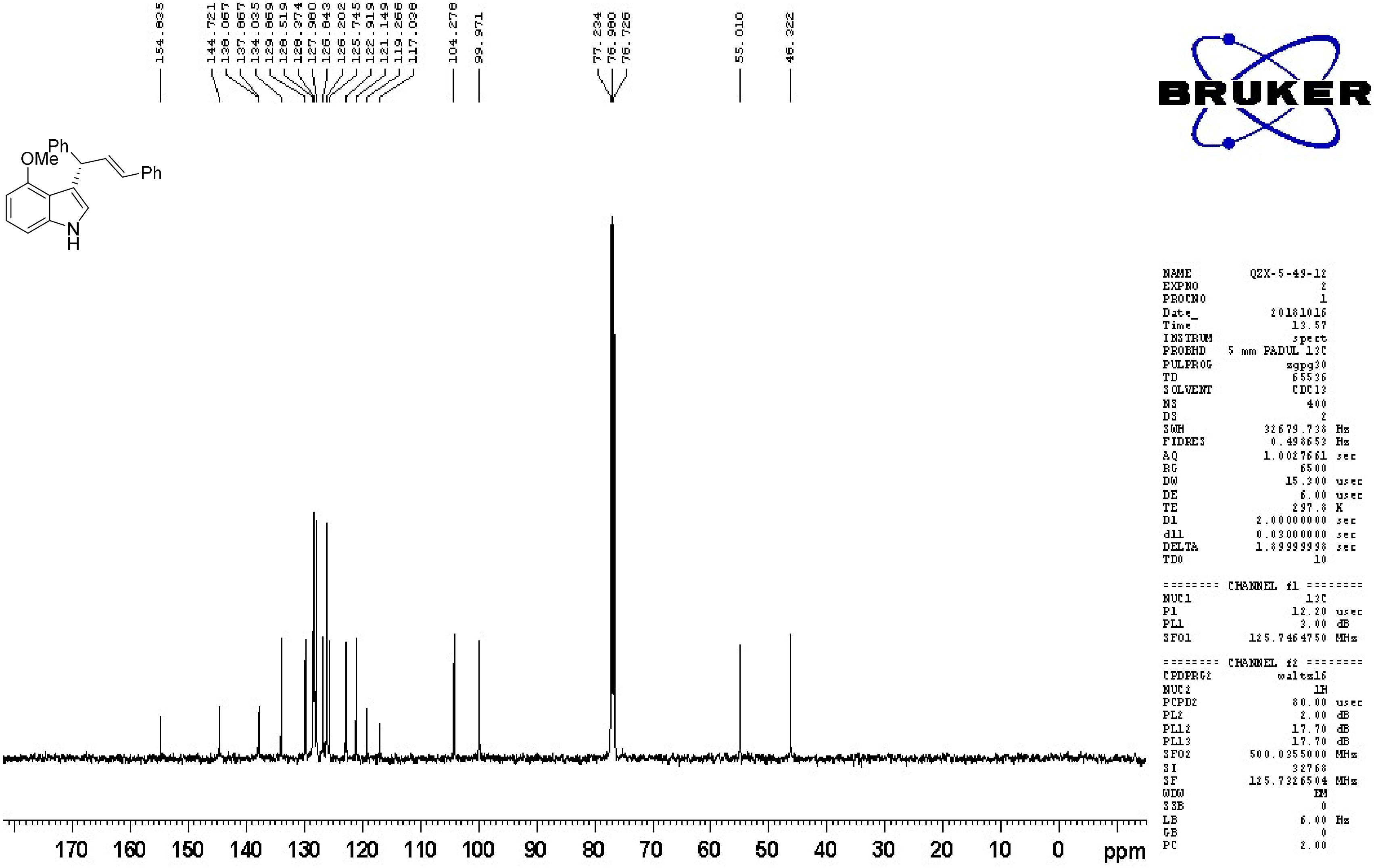
154.835
144.721
138.067
137.867
134.035
129.869
128.519
128.374
127.980
126.843
126.202
125.745
122.919
121.149
119.266
117.038
104.278
99.971
77.234
76.980
76.726
55.010
46.322
NAME QZX-5-49-12
EXPNO 2
PROCNO 1
Date_ 20181016
Time 13.57
INSTRUM spect
PROBHD 5 mm PADUL 13C
PULPROG zgpg30
TD 65536
SOLVENT CDCl3
NS 400
DS 2
SWH 32679.738 Hz
FIDRES 0.498653 Hz
AQ 1.0027661 sec
RG 6500
DW 15.300 usec
DE 6.00 usec
TE 297.8 K
D1 2.00000000 sec
d11 0.03000000 sec
DELTA 1.89999998 sec
TD0 10
======== CHANNEL f1 ========
NUC1 13C
P1 12.20 usec
PL1 3.00 dB
SFO1 125.7464750 MHz
======== CHANNEL f2 ========
CPDPRG2 waltz16
NUC2 1H
PCPD2 80.00 usec
PL2 2.00 dB
PL12 17.70 dB
PL13 17.70 dB
SFO2 500.0355000 MHz
SI 32768
SF 125.7326504 MHz
WDW EM
SSB 0
LB 6.00 Hz
GB 0
PC 2.00
170 160 150 140 130 120 110 100 90 80 70 60 50 40 30 20 10 0 ppm

(*S*,*E*)-3-(1,3-diphenylallyl)-5-methyl-1H-indole **(3f)**

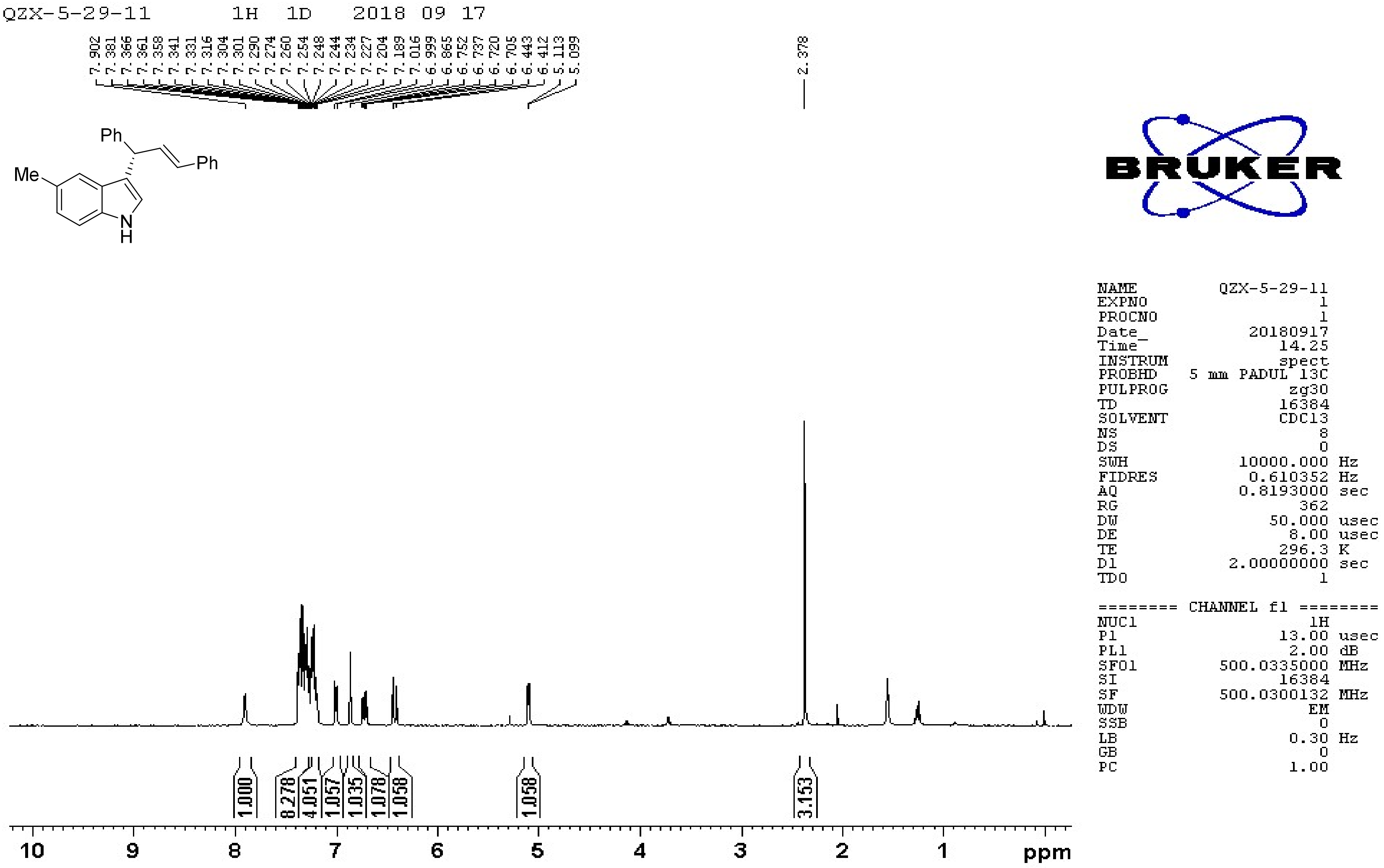

QZX-5-29-12 13C 2018 09 17

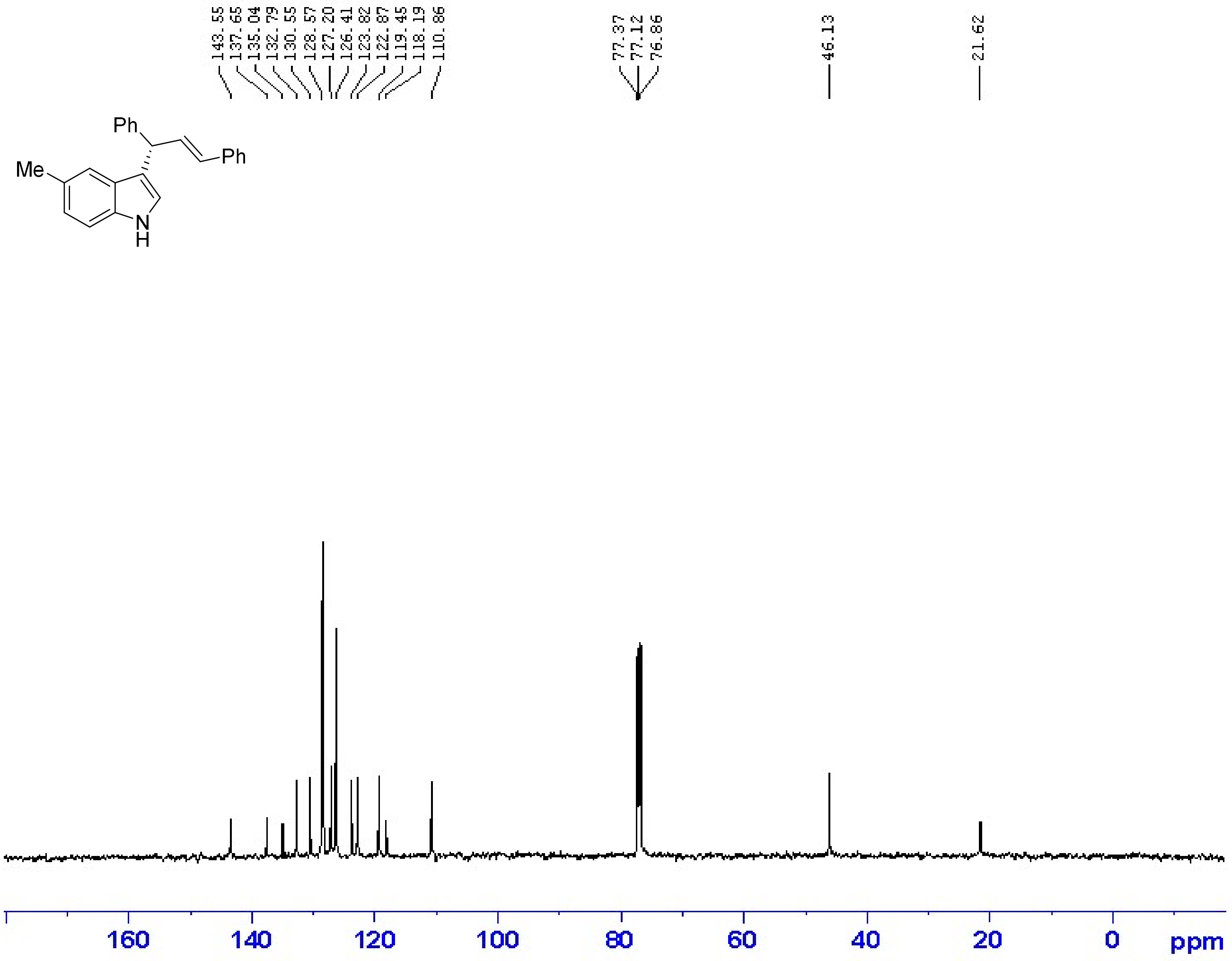

BRUKER

NAME	QZX-5-29-12
EXPNO	2
PROCNO	1
Date_	20180917
Time	17.30
INSTRUM	spect
PROBHD	5 mm PADUL 13C
PULPROG	zgpg30
TD	65536
SOLVENT	CDCl3
NS	106
DS	1
SWH	32679.738 Hz
FIDRES	0.498653 Hz
AQ	1.0027661 sec
RG	3250
DW	15.300 usec
DE	6.00 usec
TE	298.5 K
D1	2.00000000 sec
d11	0.03000000 sec
DELTA	1.89999998 sec
TD0	20
======== CHANNEL f1 ========	
NUC1	13C
P1	12.20 usec
PL1	3.00 dB
SFO1	125.7464750 MHz
======== CHANNEL f2 ========	
CPDPRG2	waltz16
NUC2	1H
PCPD2	80.00 usec
PL2	2.00 dB
PL12	17.70 dB
PL13	17.70 dB
SFO2	500.0355000 MHz
SI	32768
SF	125.7326392 MHz
WDW	EM
SSB	0
LB	10.00 Hz
GB	0
PC	1.00

(*S*,*E*)-3-(1,3-diphenylallyl)-5-methoxy-1H-indole **(3g)**

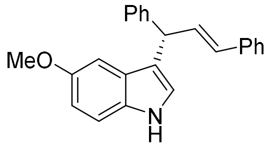

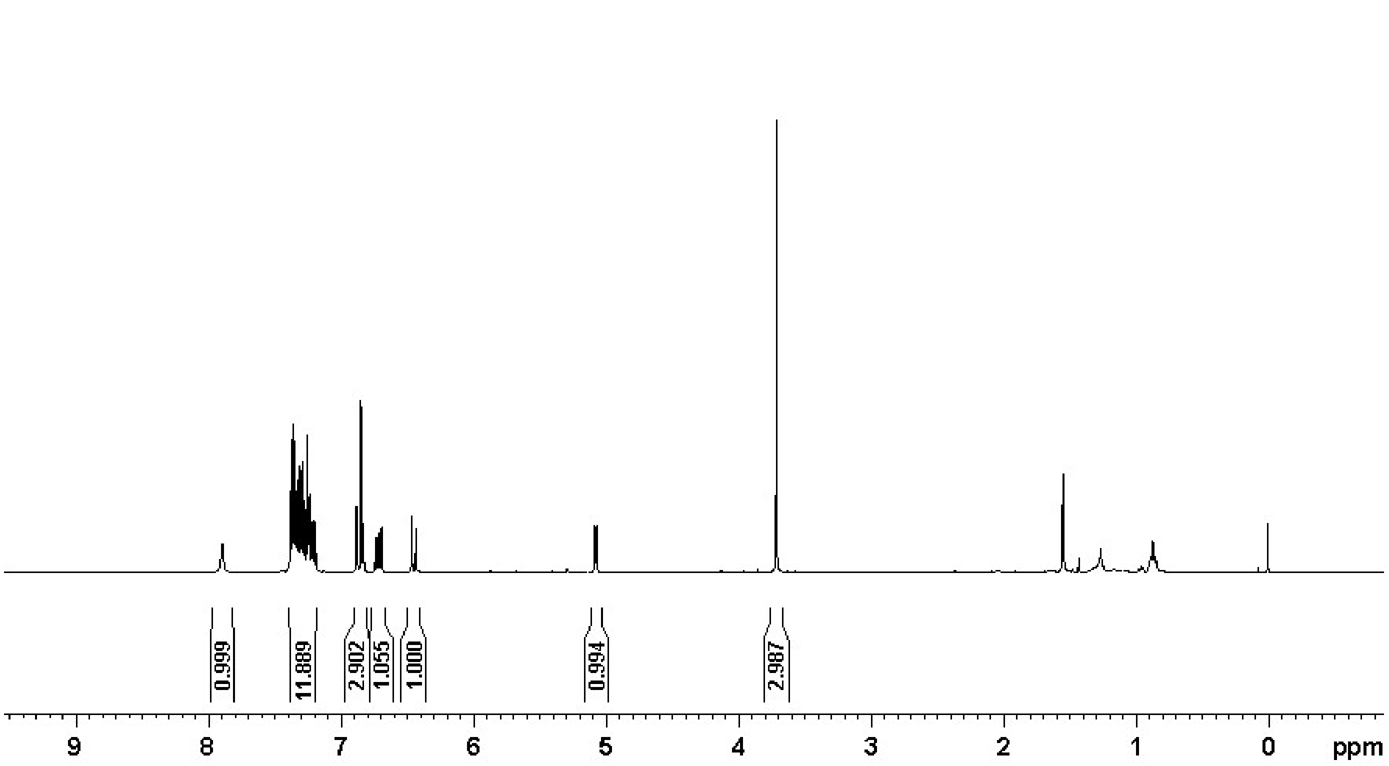

NAME QZX-5-36-11(1030)
EXPNO 1
PROCNO 1
Date_ 20181030
Time 11.27
INSTRUM spect
PROBHD 5 mm PADUL 13C
PULPROG zg30
TD 16384
SOLVENT CDCl3
NS 8
DS 1
SWH 10000.000 Hz
FIDRES 0.610352 Hz
AQ 0.8193000 sec
RG 322
DW 50.000 usec
DE 8.00 usec
TE 298.2 K
D1 2.00000000 sec
TD0 1

======== CHANNEL f1 ========
NUC1 1H
P1 13.00 usec
PL1 2.00 dB
SFO1 500.0335000 MHz
SI 16384
SF 500.0300121 MHz
WDW EM
SSB 0
LB 0.30 Hz
GB 0
PC 1.00

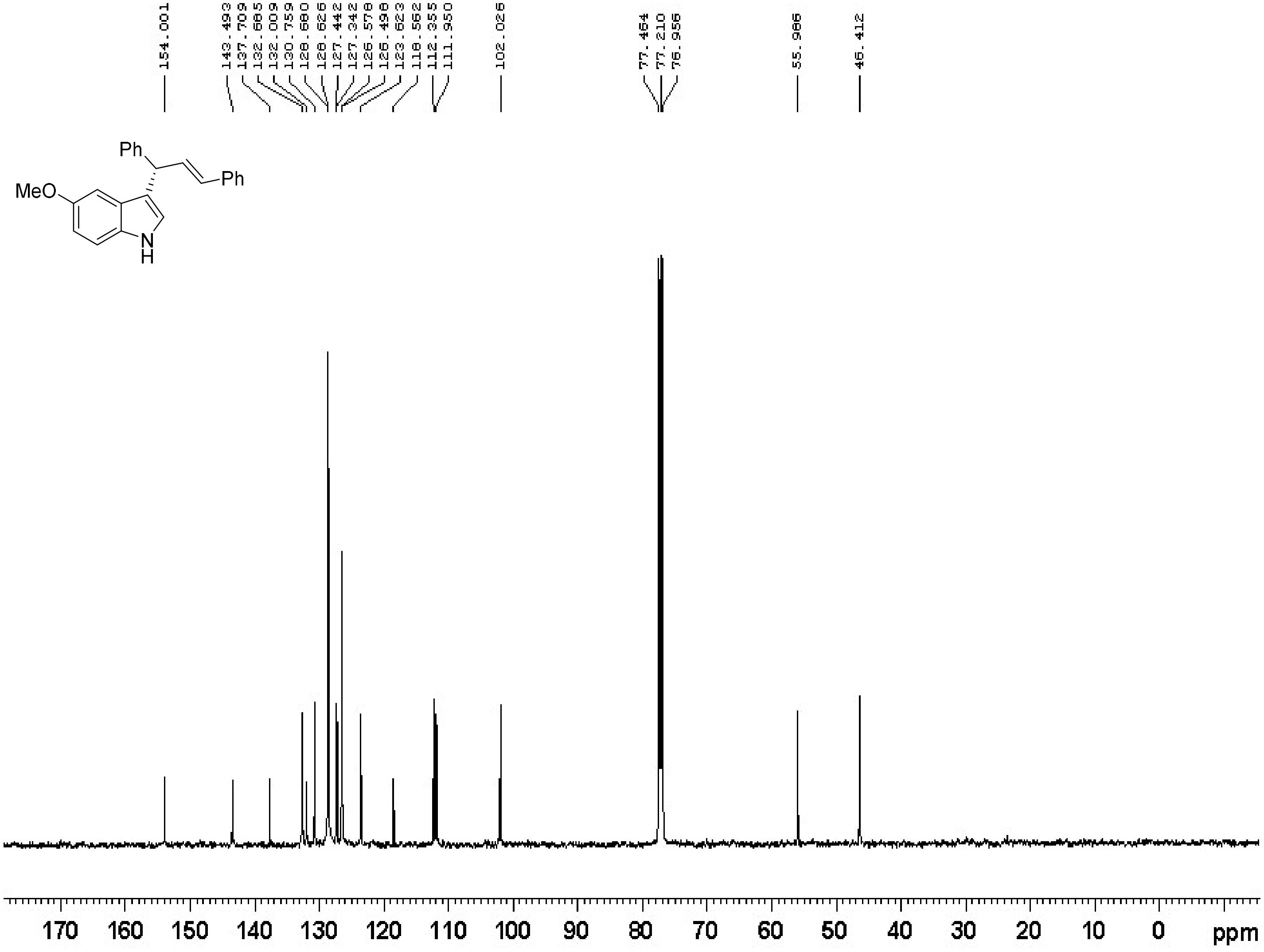

NAME	QZX-5-36-12	
EXPNO	2	
PROCNO	1	
Date_	20181030	
Time	12.21	
INSTRUM	spect	
PROBHD	5 mm PADUL 13C	
PULPROG	zgpg30	
TD	65536	
SOLVENT	CDCl3	
NS	744	
DS	1	
SWH	32679.738	Hz
FIDRES	0.498653	Hz
AQ	1.0027661	sec
RG	6500	
DW	15.300	usec
DE	6.00	usec
TE	298.1	K
D1	2.00000000	sec
d11	0.03000000	sec
DELTA	1.89999998	sec
TD0	20	
======== CHANNEL f1 ========		
NUC1	13C	
P1	12.20	usec
PL1	3.00	dB
SFO1	125.7464750	MHz
======== CHANNEL f2 ========		
CPDPRG2	waltz16	
NUC2	1H	
PCPD2	80.00	usec
PL2	2.00	dB
PL12	17.70	dB
PL13	17.70	dB
SFO2	500.0355000	MHz
SI	32768	
SF	125.7326225	MHz
WDW	EM	
SSB	0	
LB	6.00	Hz
GB	0	
PC	1.00	

(*S,E*)-5-(benzyloxy)-3-(1,3-diphenylallyl)-1H-indole **(3h)**

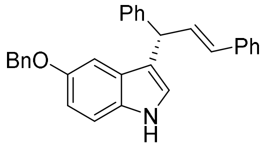

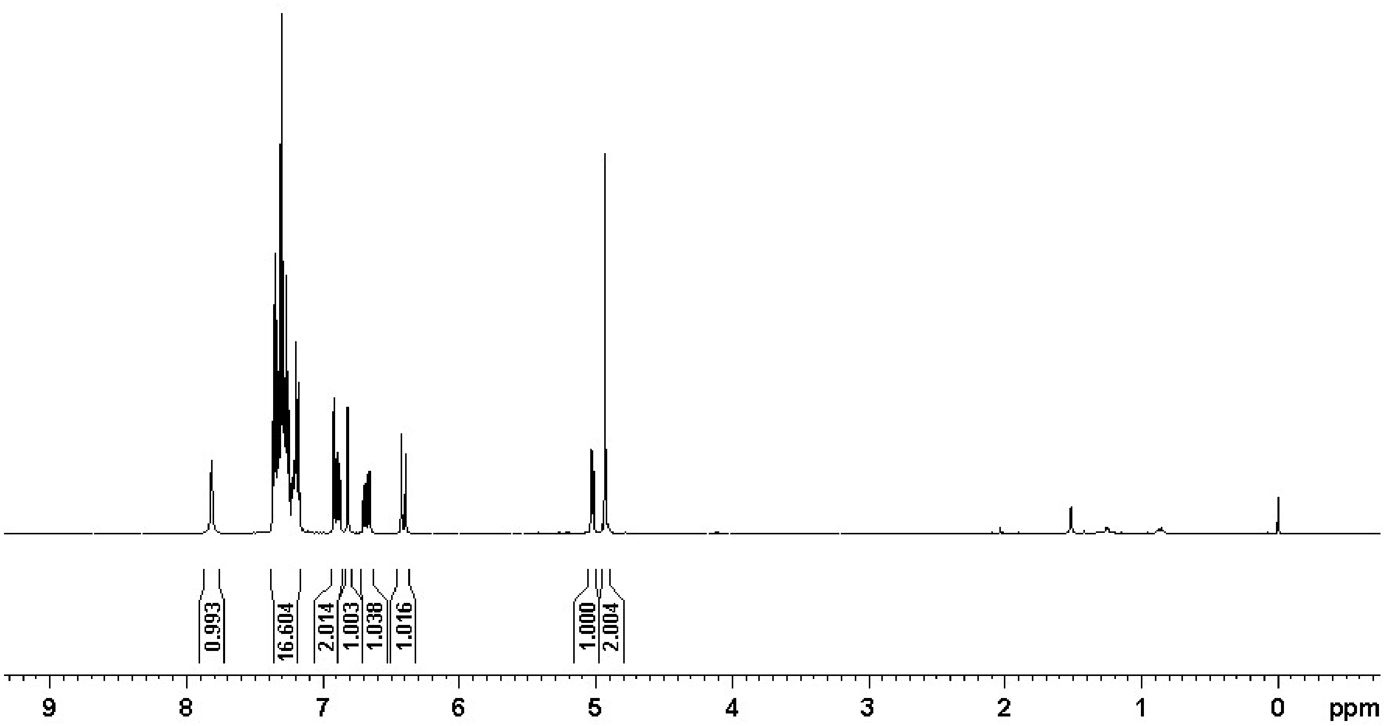


```
NAME         QZX-5-46-11
EXPNO                  1
PROCNO                 1
Date_           20181018
Time               10.19
INSTRUM            spect
PROBHD   5 mm PADUL 13C
PULPROG             zg30
TD                 16384
SOLVENT            CDCl3
NS                     8
DS                     0
SWH            10000.000 Hz
FIDRES          0.610352 Hz
AQ             0.8193000 sec
RG                   114
DW                50.000 usec
DE                  6.00 usec
TE                 293.6 K
D1            2.00000000 sec
TD0                    1

======== CHANNEL f1 ========
NUC1                  1H
P1                 13.00 usec
PL1                 2.00 dB
SFO1         500.0335010 MHz
SI                 16384
SF           500.0300368 MHz
WDW                   EM
SSB                    0
LB                  0.30 Hz
GB                     0
PC                  1.00
```

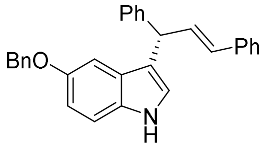

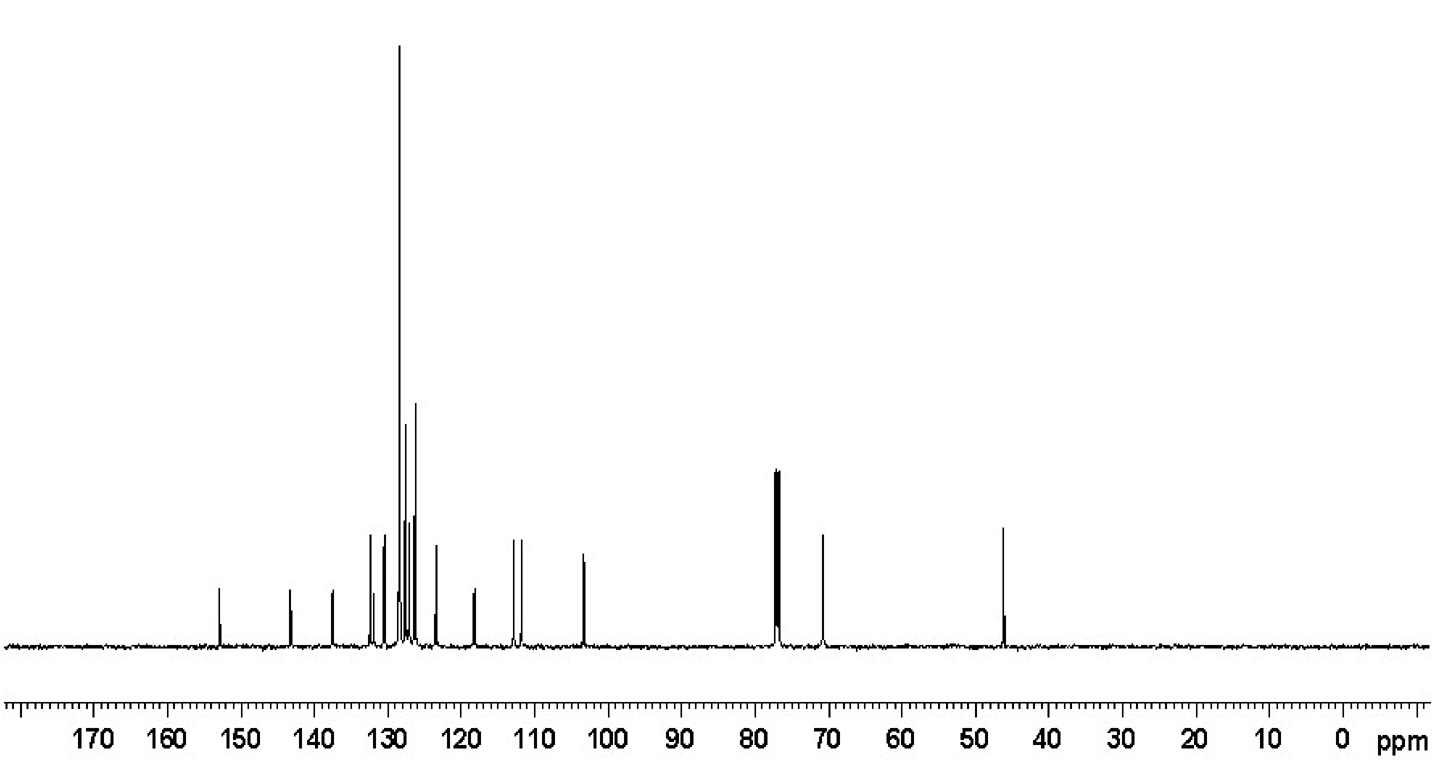

NAME	QZX-5-46-12	
EXPNO	2	
PROCNO	1	
Date_	20181018	
Time	11.03	
INSTRUM	spect	
PROBHD	5 mm PADUL 13C	
PULPROG	zgpg30	
TD	65536	
SOLVENT	CDCl3	
NS	110	
DS	2	
SWH	32679.738	Hz
FIDRES	0.498653	Hz
AQ	1.0027661	sec
RG	8200	
DW	15.300	usec
DE	6.00	usec
TE	295.4	K
D1	2.00000000	sec
d11	0.03000000	sec
DELTA	1.89999998	sec
TD0	10	

======== CHANNEL f1 ========

NUC1	13C	
P1	12.20	usec
PL1	3.00	dB
SFO1	125.7464750	MHz

======== CHANNEL f2 ========

CPDPRG2	waltz16	
NUC2	1H	
PCPD2	80.00	usec
PL2	2.00	dB
PL12	17.70	dB
PL13	17.70	dB
SFO2	500.0355000	MHz
SI	32768	
SF	125.7326556	MHz
WDW	EM	
SSB	0	
LB	6.00	Hz
GB	0	
PC	2.00	

(*S*,*E*)-5-chloro-3-(1,3-diphenylallyl)-1H-indole **(3i)**

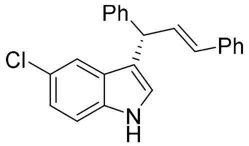

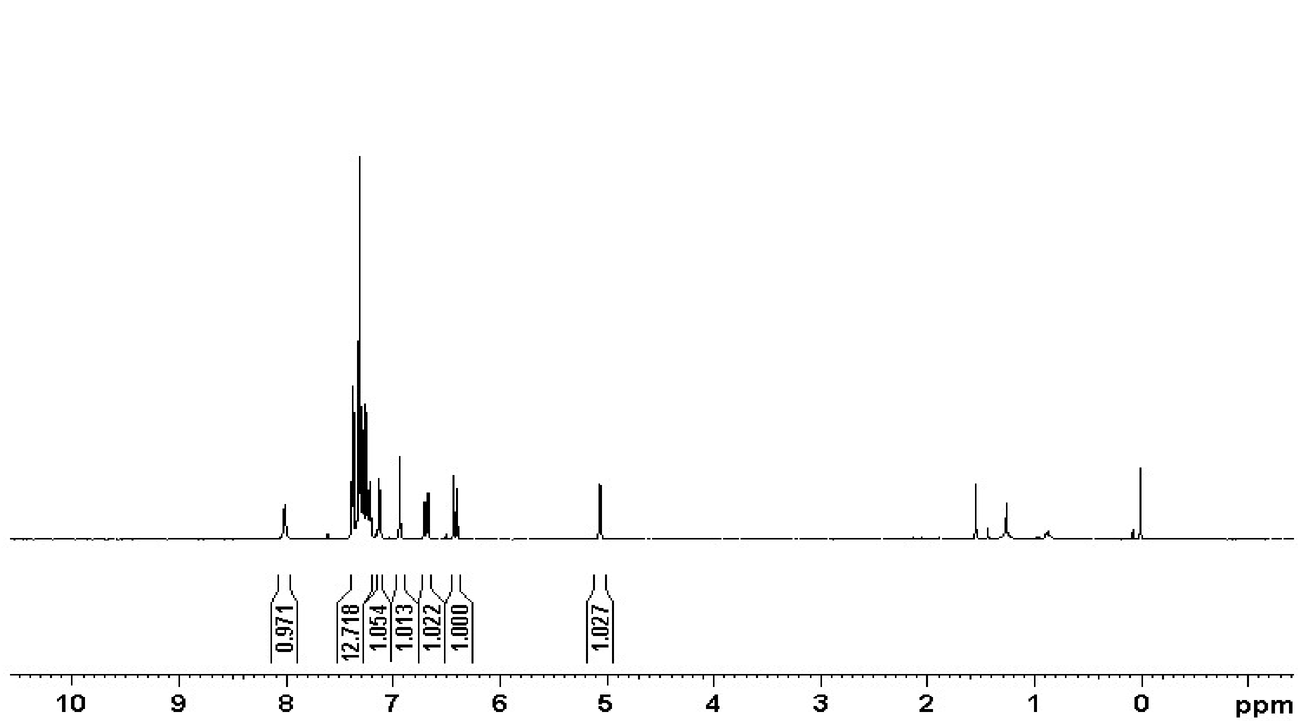


```
NAME          QZX-5-28-11
EXPNO                   1
PROCNO                  1
Date_            20180917
Time                16.15
INSTRUM             spect
PROBHD    5 mm PADUL 13C
PULPROG              zg30
TD                  16384
SOLVENT             CDCl3
NS                      8
DS                      0
SWH             10000.000 Hz
FIDRES           0.610352 Hz
AQ              0.8193000 sec
RG                    228
DW                 50.000 usec
DE                   8.00 usec
TE                  296.6 K
D1             2.00000000 sec
TD0                     1

======== CHANNEL f1 ========
NUC1                   1H
P1                  13.00 usec
PL1                  2.00 dB
SFO1          500.0335000 MHz
SI                  16384
SF            500.0300132 MHz
WDW                    EM
SSB                     0
LB                   0.30 Hz
GB                      0
PC                   1.00
```

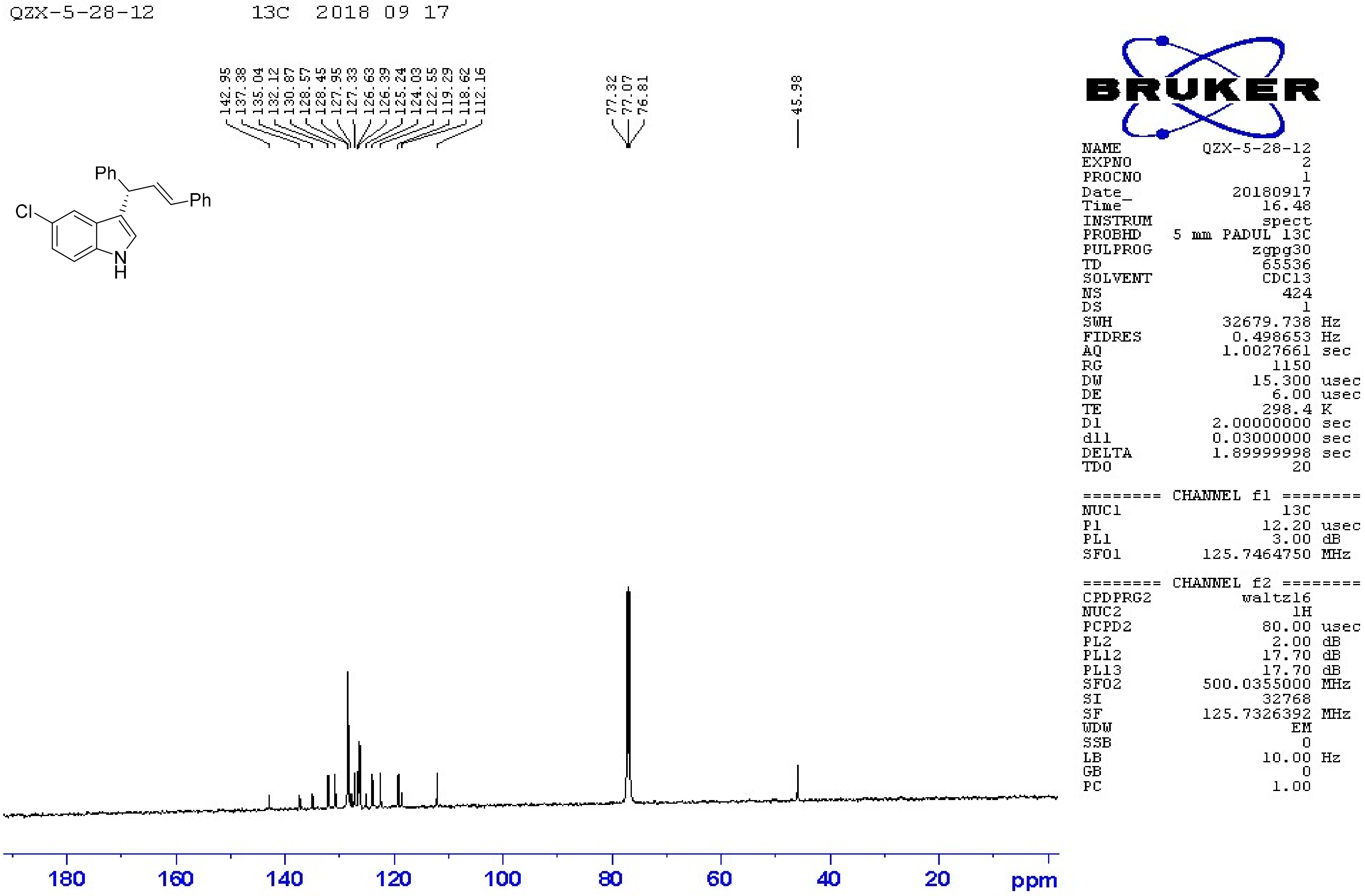
QZX-5-28-12 13C 2018 09 17
142.95
137.38
135.04
132.12
130.87
128.57
128.45
127.95
127.33
126.63
126.39
125.24
124.03
122.55
119.29
118.62
112.16
77.32
77.07
76.81
45.98
Ph
Ph
Cl
N
H
BRUKER
NAME QZX-5-28-12
EXPNO 2
PROCNO 1
Date_ 20180917
Time 16.48
INSTRUM spect
PROBHD 5 mm PADUL 13C
PULPROG zgpg30
TD 65536
SOLVENT CDCl3
NS 424
DS 1
SWH 32679.738 Hz
FIDRES 0.498653 Hz
AQ 1.0027661 sec
RG 1150
DW 15.300 usec
DE 6.00 usec
TE 298.4 K
D1 2.00000000 sec
d11 0.03000000 sec
DELTA 1.89999998 sec
TD0 20
======== CHANNEL f1 ========
NUC1 13C
P1 12.20 usec
PL1 3.00 dB
SFO1 125.7464750 MHz
======== CHANNEL f2 ========
CPDPRG2 waltz16
NUC2 1H
PCPD2 80.00 usec
PL2 2.00 dB
PL12 17.70 dB
PL13 17.70 dB
SFO2 500.0355000 MHz
SI 32768
SF 125.7326392 MHz
WDW EM
SSB 0
LB 10.00 Hz
GB 0
PC 1.00
180
160
140
120
100
80
60
40
20
ppm

(*S*,*E*)-5-bromo-3-(1,3-diphenylallyl)-1H-indole **(3j)**

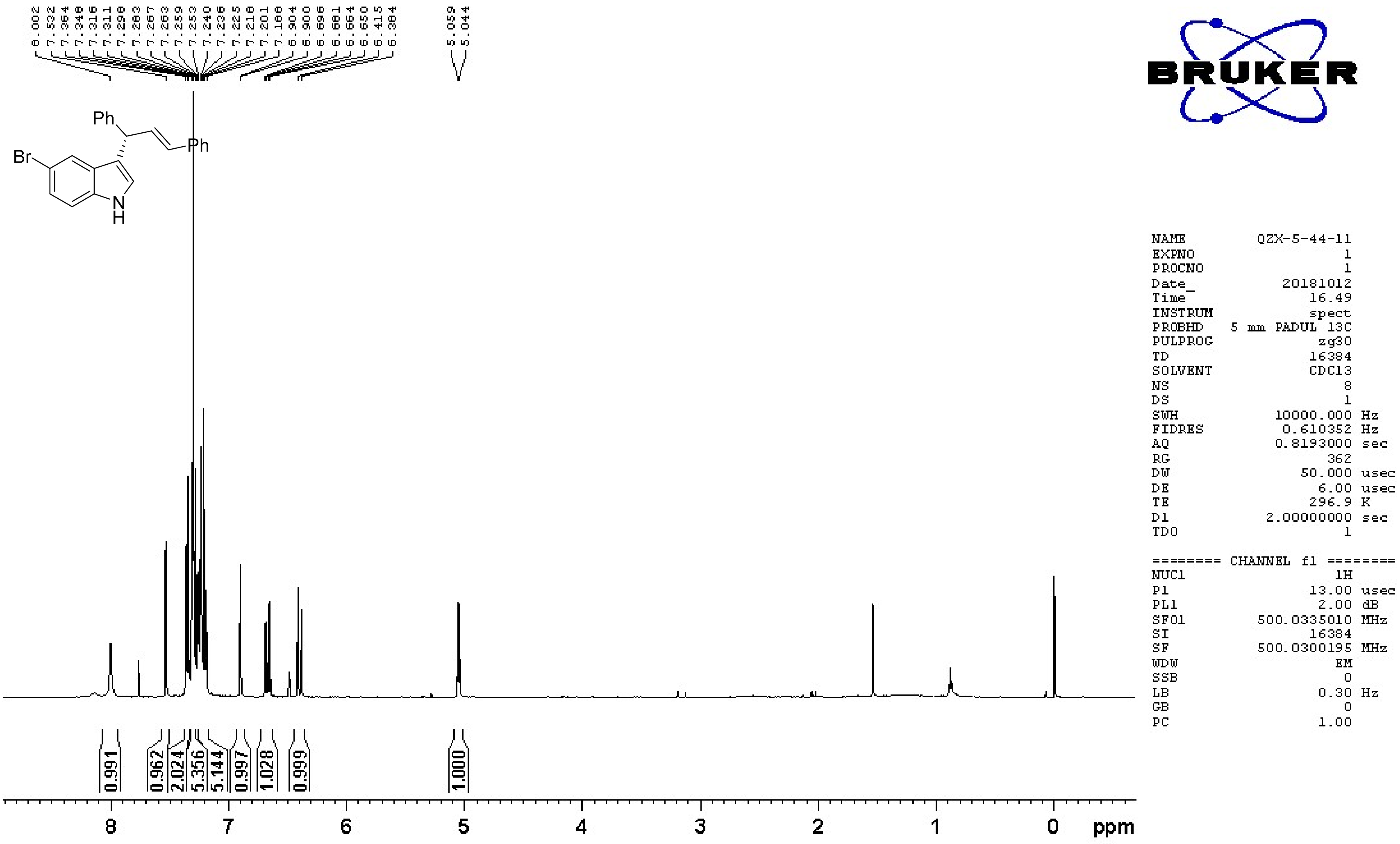

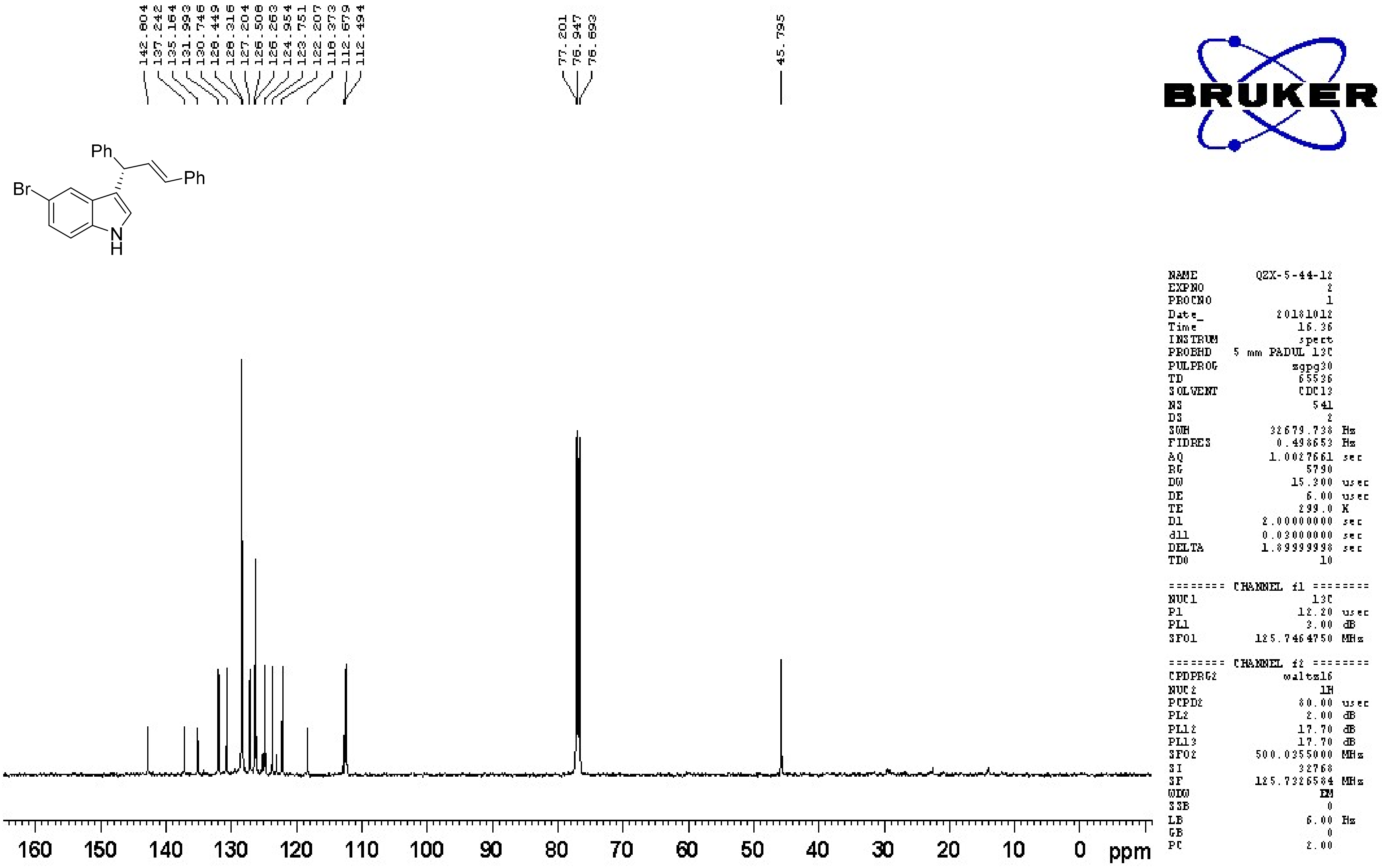

142.604
137.242
135.164
131.993
130.746
128.449
128.316
127.204
126.508
126.263
124.954
123.751
122.207
118.373
112.679
112.494
77.201
76.947
76.693
45.795
Ph
Ph
Br
N
H
BRUKER
NAME QZX-5-44-12
EXPNO 2
PROCNO 1
Date_ 20181012
Time 16.36
INSTRUM spect
PROBHD 5 mm PADUL 13C
PULPROG zgpg30
TD 65536
SOLVENT CDCl3
NS 541
DS 2
SWH 32679.738 Hz
FIDRES 0.498653 Hz
AQ 1.0027661 sec
RG 5790
DW 15.300 usec
DE 6.00 usec
TE 299.0 K
D1 2.00000000 sec
d11 0.03000000 sec
DELTA 1.89999998 sec
TD0 10
======== CHANNEL f1 ========
NUC1 13C
P1 12.20 usec
PL1 3.00 dB
SFO1 125.7464750 MHz
======== CHANNEL f2 ========
CPDPRG2 waltz16
NUC2 1H
PCPD2 80.00 usec
PL2 2.00 dB
PL12 17.70 dB
PL13 17.70 dB
SFO2 500.0355000 MHz
SI 32768
SF 125.7326584 MHz
WDW EM
SSB 0
LB 6.00 Hz
GB 0
PC 2.00
160 150 140 130 120 110 100 90 80 70 60 50 40 30 20 10 0 ppm

(*S*,*E*)-3-(1,3-diphenylallyl)-6-methyl-1H-indole **(3k)**

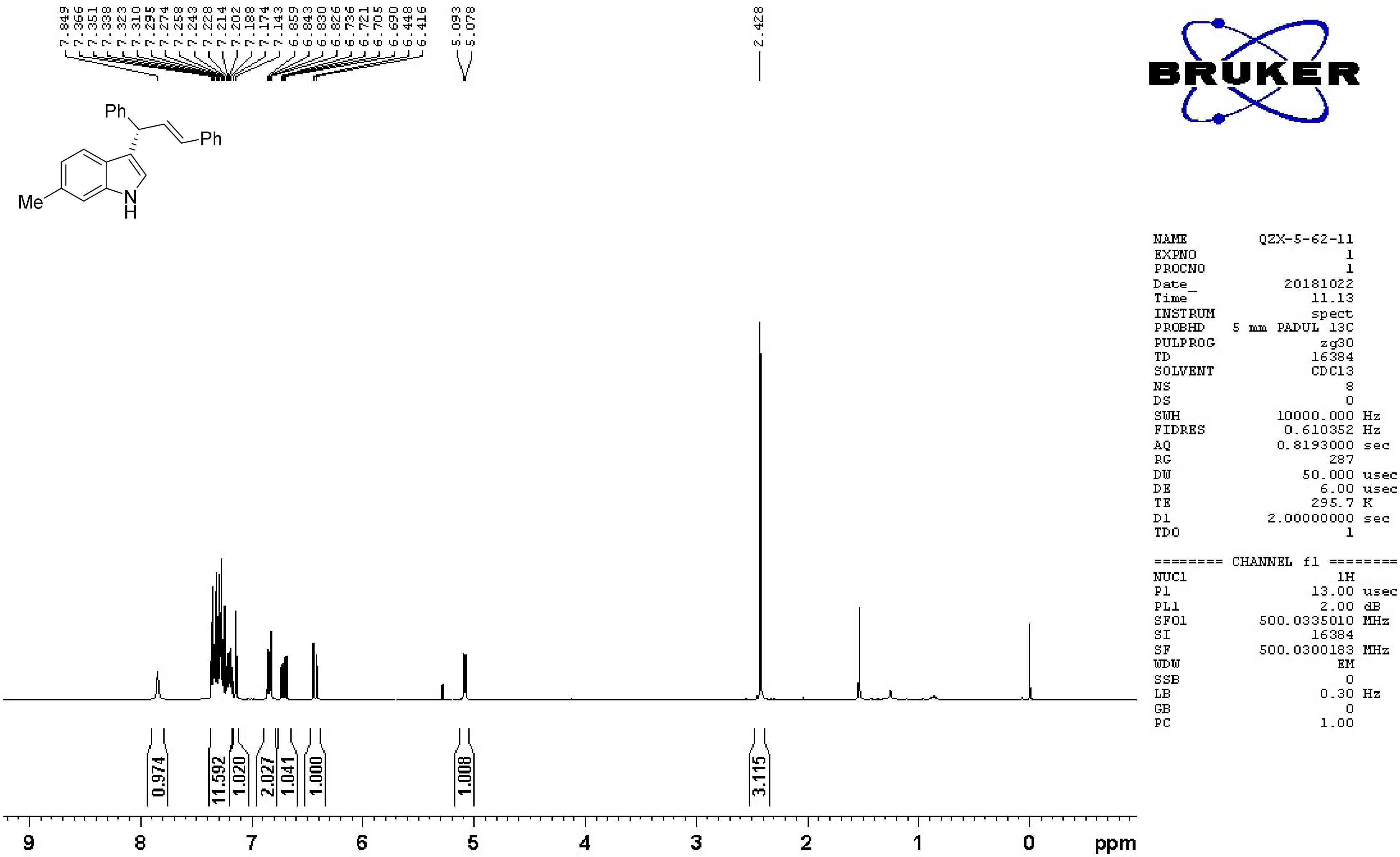

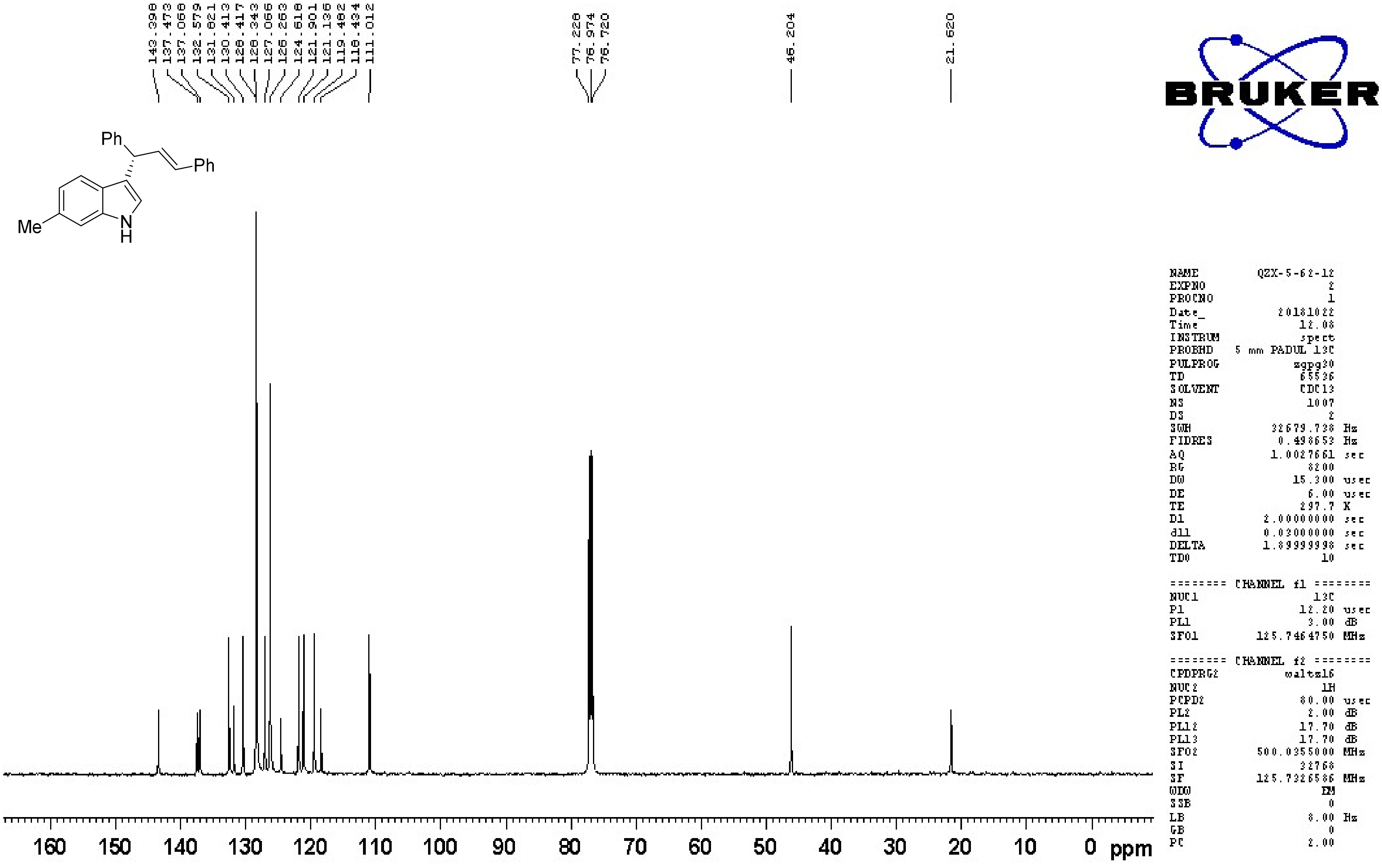
143.398
137.473
137.060
132.579
131.621
130.413
128.417
128.343
127.055
125.253
124.618
121.901
121.135
119.482
118.434
111.012
77.228
76.974
76.720
46.204
21.620
Ph
Ph
Me
N
H
BRUKER
NAME QZX-5-62-12
EXPNO 2
PROCNO 1
Date_ 20181022
Time 12.08
INSTRUM spect
PROBHD 5 mm PADUL 13C
PULPROG zgpg30
TD 65536
SOLVENT CDCl3
NS 1007
DS 2
SWH 32679.738 Hz
FIDRES 0.498653 Hz
AQ 1.0027661 sec
RG 8200
DW 15.300 usec
DE 6.00 usec
TE 297.7 K
D1 2.00000000 sec
d11 0.03000000 sec
DELTA 1.89999998 sec
TD0 10
======== CHANNEL f1 ========
NUC1 13C
P1 12.20 usec
PL1 3.00 dB
SFO1 125.7464750 MHz
======== CHANNEL f2 ========
CPDPRG2 waltz16
NUC2 1H
PCPD2 80.00 usec
PL2 2.00 dB
PL12 17.70 dB
PL13 17.70 dB
SFO2 500.0355000 MHz
SI 32768
SF 125.7326586 MHz
WDW EM
SSB 0
LB 8.00 Hz
GB 0
PC 2.00
160 150 140 130 120 110 100 90 80 70 60 50 40 30 20 10 0 ppm

(*S*,*E*)-6-chloro-3-(1,3-diphenylallyl)-1H-indole **(3l)**

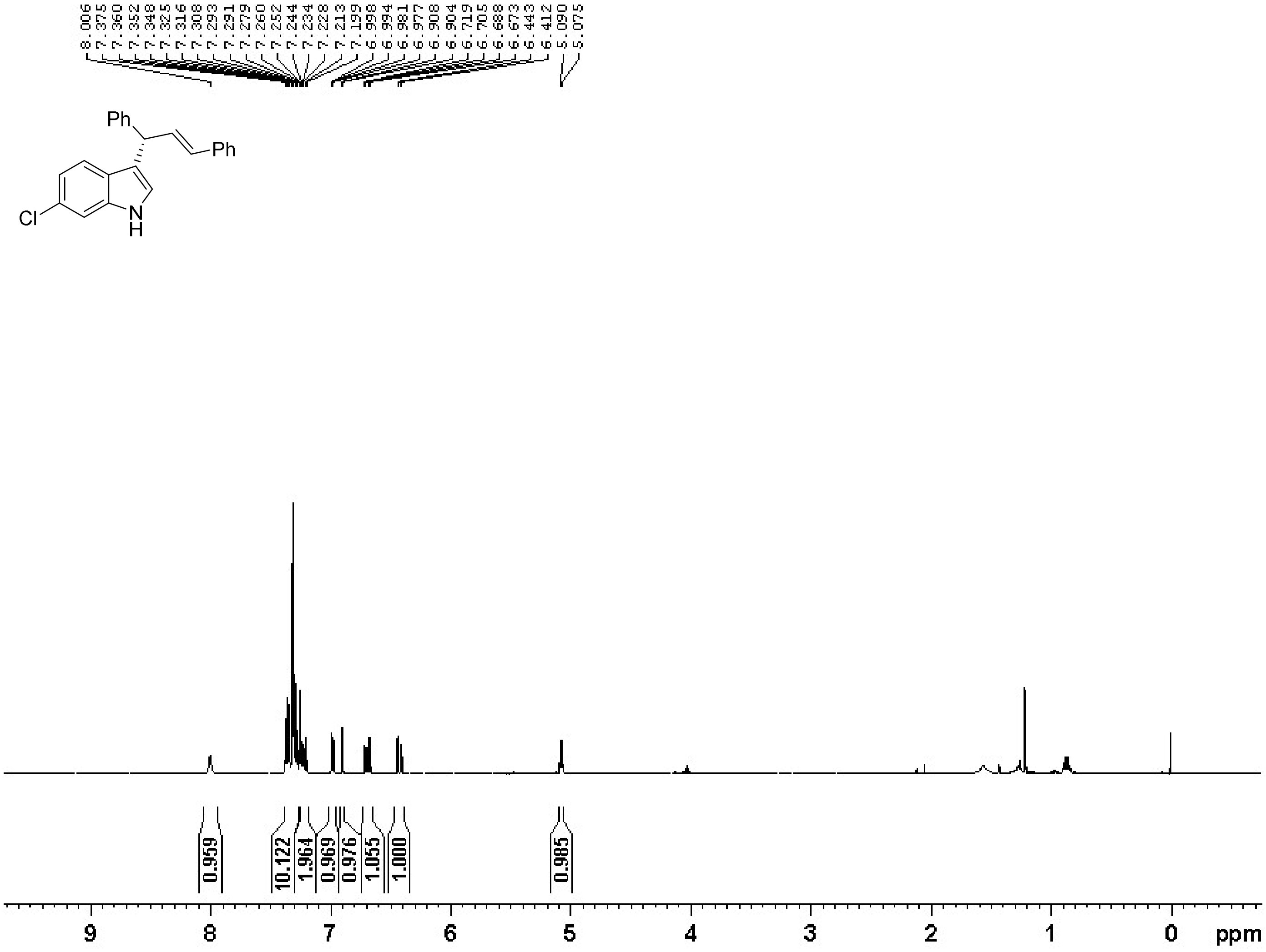


```
NAME         QZX-5-64-11
EXPNO                  1
PROCNO                 1
Date_           20181024
Time               11.49
INSTRUM            spect
PROBHD   5 mm PADUL 13C
PULPROG             zg30
TD                 16384
SOLVENT            CDCl3
NS                     8
DS                     0
SWH            10000.000 Hz
FIDRES          0.610352 Hz
AQ             0.8193000 sec
RG                   287
DW                50.000 usec
DE                  6.00 usec
TE                 295.7 K
D1            2.00000000 sec
TD0                    1

======== CHANNEL f1 ========
NUC1                  1H
P1                 13.00 usec
PL1                 2.00 dB
SFO1         500.0335010 MHz
SI                 16384
SF           500.0300101 MHz
WDW                   EM
SSB                    0
LB                  0.30 Hz
GB                     0
PC                  1.00
```

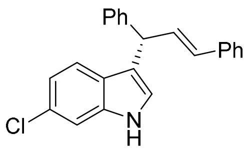

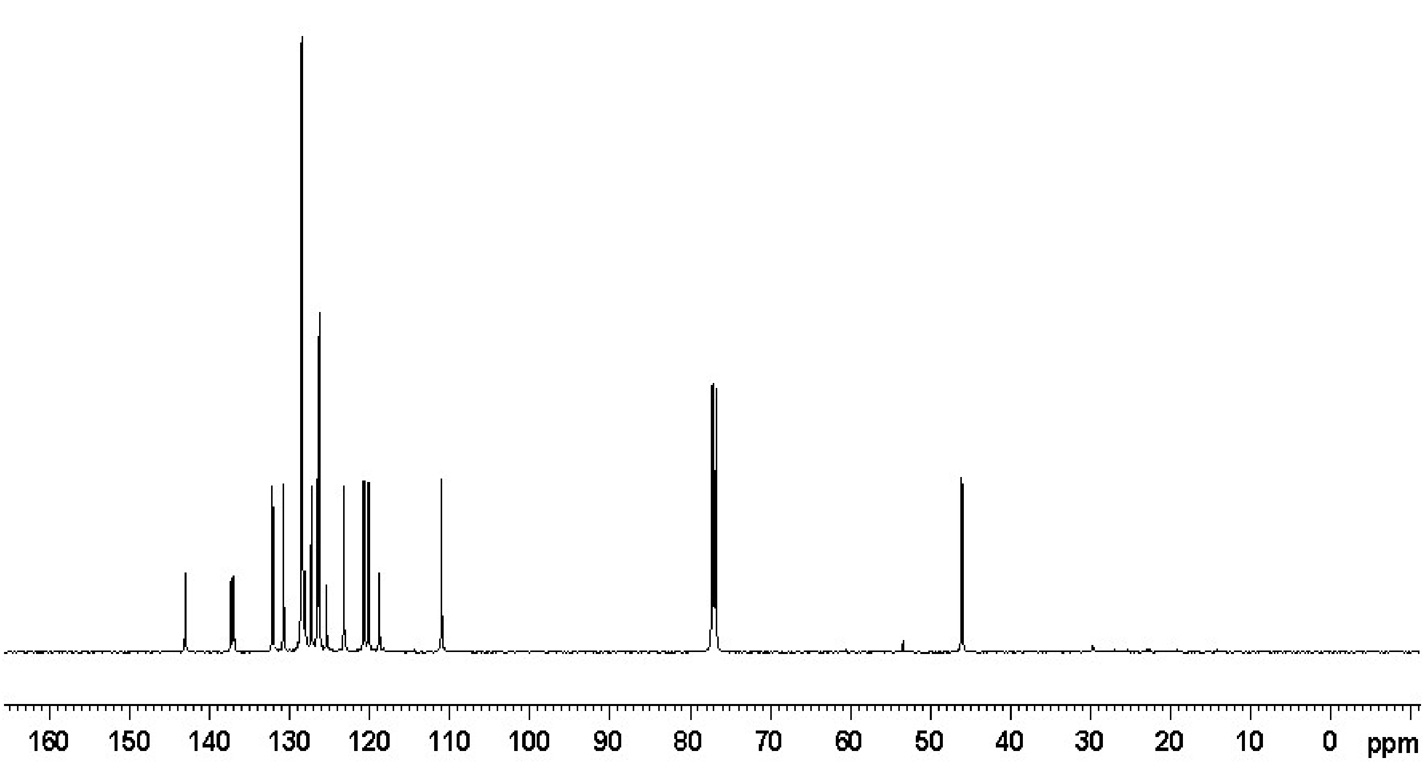

NAME	QZX-5-64-12	
EXPNO	2	
PROCNO	1	
Date_	20181024	
Time	12.55	
INSTRUM	spect	
PROBHD	5 mm PADUL 13C	
PULPROG	zgpg30	
TD	65536	
SOLVENT	CDCl3	
NS	2173	
DS	2	
SWH	32679.738	Hz
FIDRES	0.498653	Hz
AQ	1.0027661	sec
RG	8200	
DW	15.300	usec
DE	6.00	usec
TE	297.5	K
D1	2.00000000	sec
d11	0.03000000	sec
DELTA	1.89999998	sec
TD0	10	
======== CHANNEL f1 ========		
NUC1	13C	
P1	12.20	usec
PL1	3.00	dB
SFO1	125.7464750	MHz
======== CHANNEL f2 ========		
CPDPRG2	waltz16	
NUC2	1H	
PCPD2	80.00	usec
PL2	2.00	dB
PL12	17.70	dB
PL13	17.70	dB
SFO2	500.0355000	MHz
SI	32768	
SF	125.7326504	MHz
WDW	EM	
SSB	0	
LB	8.00	Hz
GB	0	
PC	2.00	

(*S*,*E*)-3-(1,3-diphenylallyl)-7-methyl-1H-indole **(3m)**

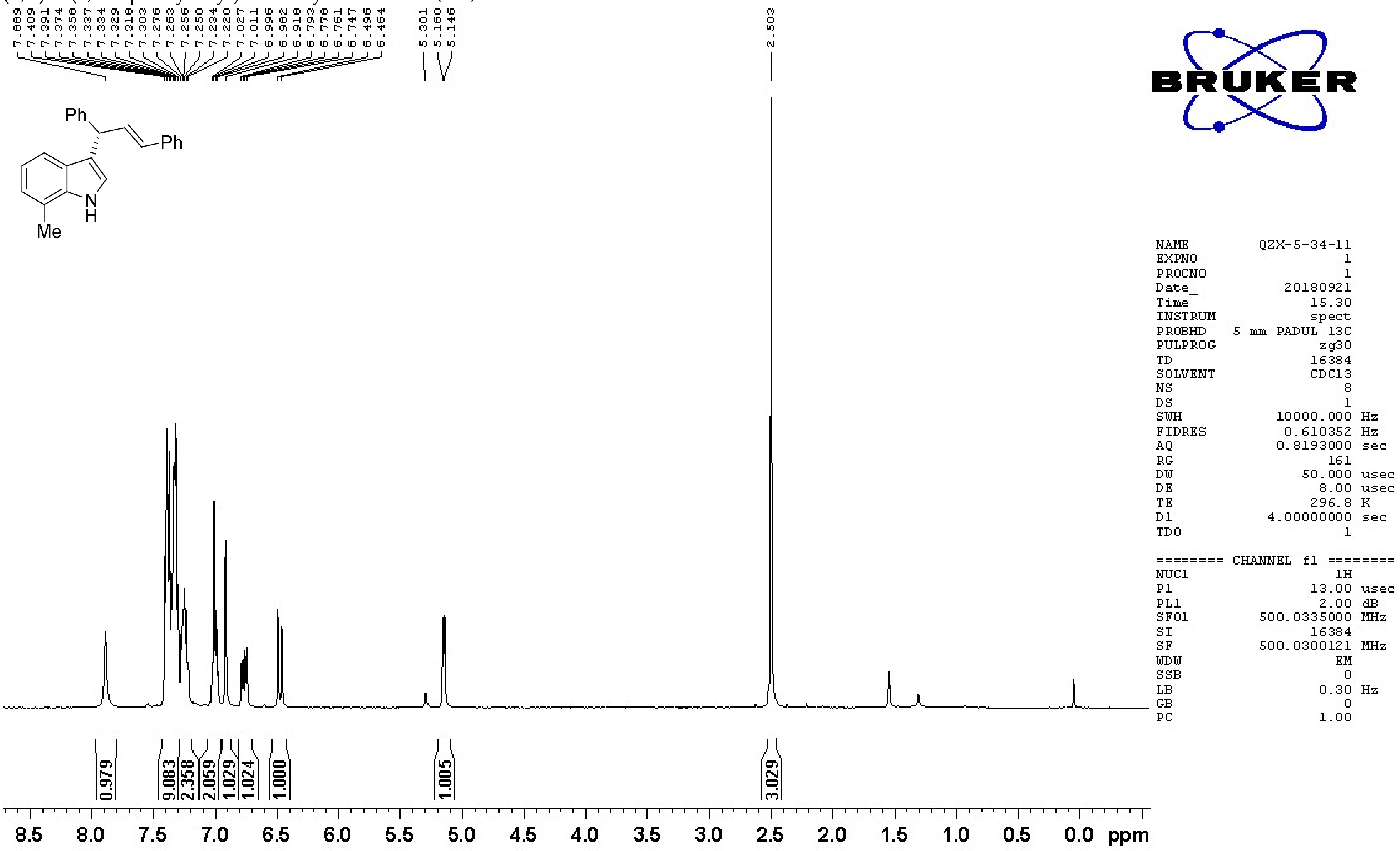

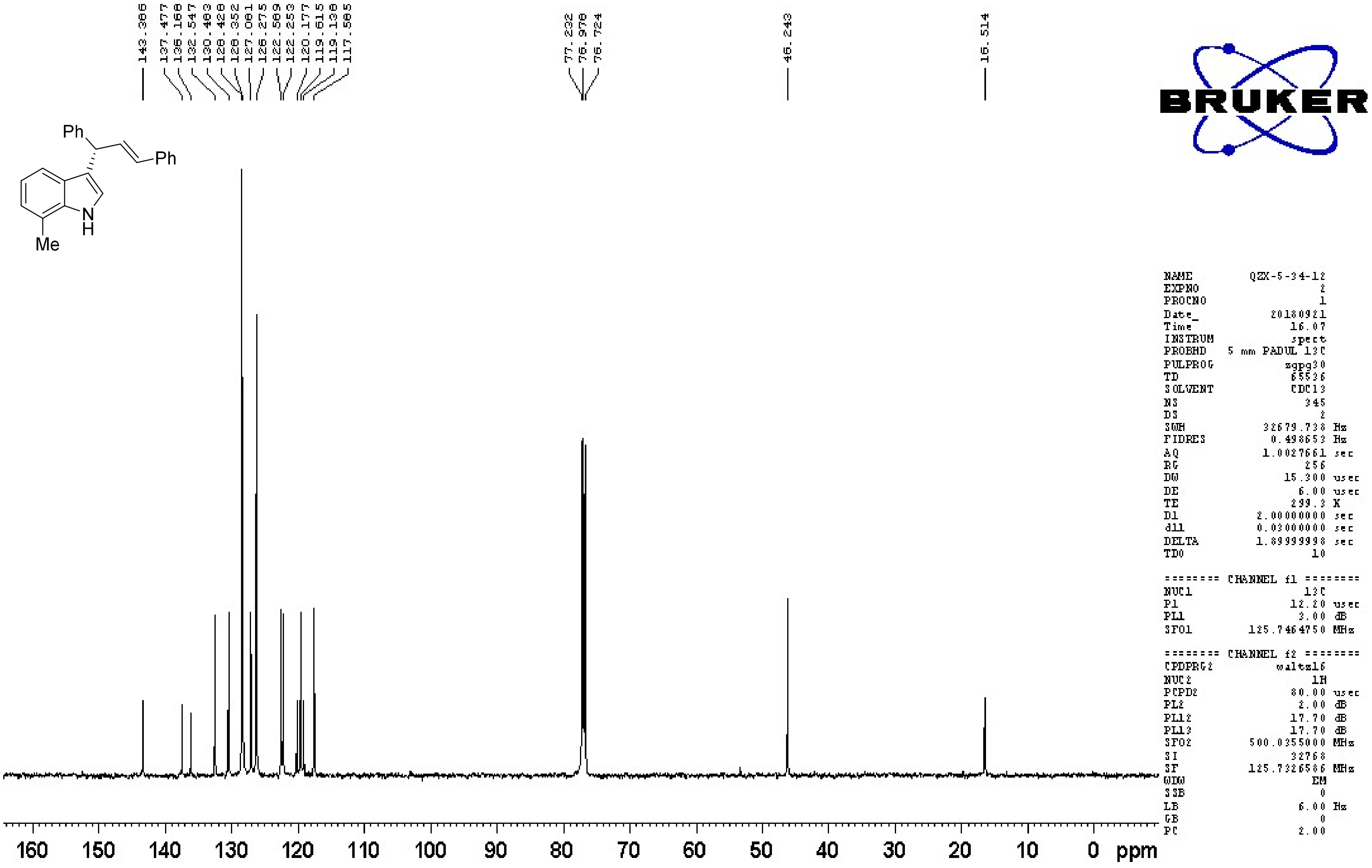
143.306
137.477
136.168
132.547
130.403
128.428
128.352
127.061
126.275
122.509
122.253
120.177
119.615
119.136
117.585
77.232
76.978
76.724
46.243
16.514
Ph
Ph
N
H
Me
BRUKER
NAME QZX-5-34-12
EXPNO 2
PROCNO 1
Date_ 20180921
Time 16.07
INSTRUM spect
PROBHD 5 mm PADUL 13C
PULPROG zgpg30
TD 65536
SOLVENT CDCl3
NS 345
DS 2
SWH 32679.738 Hz
FIDRES 0.498653 Hz
AQ 1.0027661 sec
RG 256
DW 15.300 usec
DE 6.00 usec
TE 299.3 K
D1 2.00000000 sec
d11 0.03000000 sec
DELTA 1.89999998 sec
TD0 10
======== CHANNEL f1 ========
NUC1 13C
P1 12.20 usec
PL1 3.00 dB
SFO1 125.7464750 MHz
======== CHANNEL f2 ========
CPDPRG2 waltz16
NUC2 1H
PCPD2 80.00 usec
PL2 2.00 dB
PL12 17.70 dB
PL13 17.70 dB
SFO2 500.0355000 MHz
SI 32768
SF 125.7326586 MHz
WDW EM
SSB 0
LB 6.00 Hz
GB 0
PC 2.00
160 150 140 130 120 110 100 90 80 70 60 50 40 30 20 10 0 ppm

(*S*,*E*)-3-(1,3-diphenylallyl)-7-methoxy-1H-indole **(3n)**

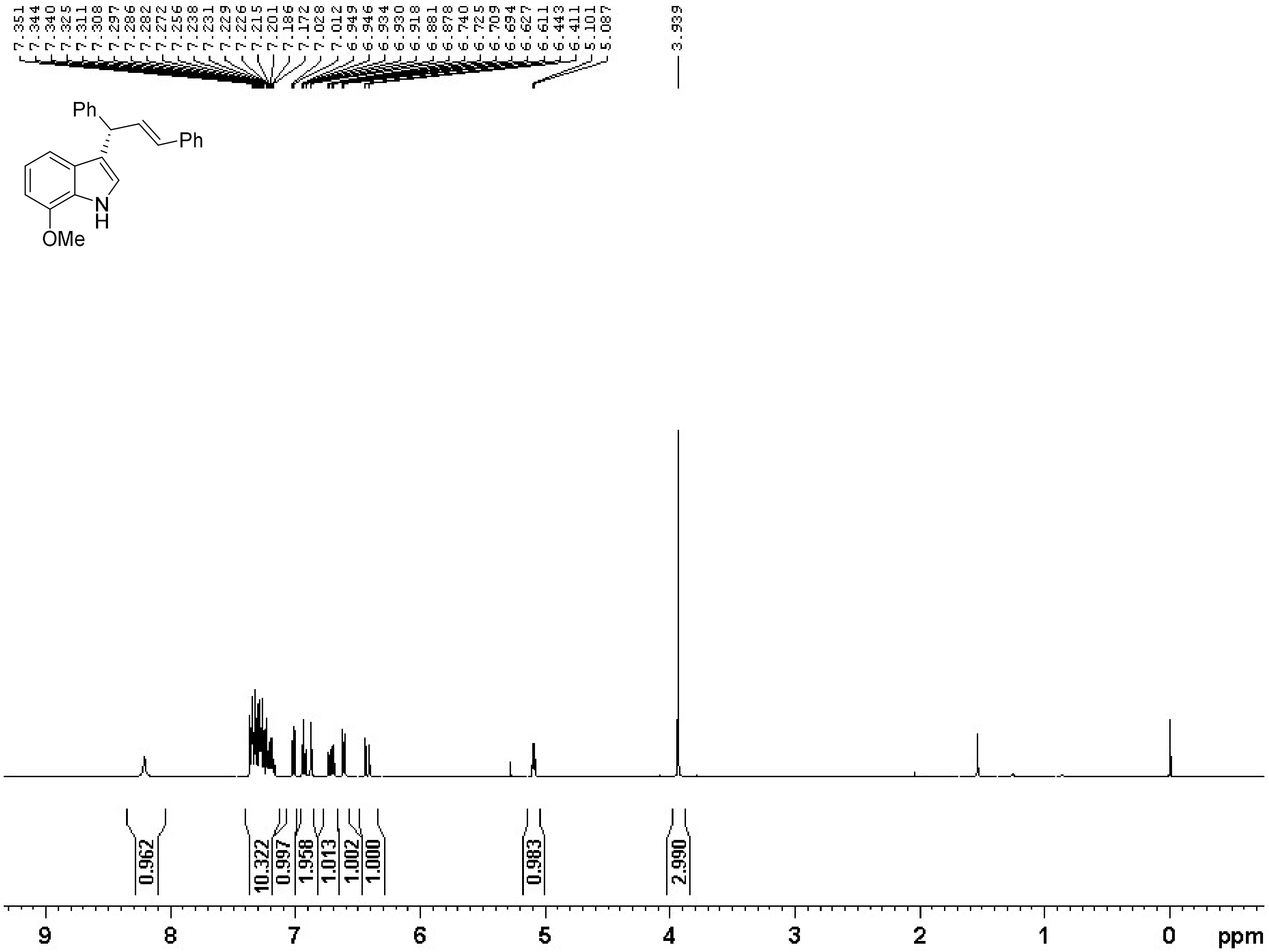

NAME	QZX-5-50-11
EXPNO	1
PROCNO	1
Date_	20181016
Time	16.40
INSTRUM	spect
PROBHD	5 mm PADUL 13C
PULPROG	zg30
TD	16384
SOLVENT	CDCl3
NS	8
DS	0
SWH	10000.000 Hz
FIDRES	0.610352 Hz
AQ	0.8193000 sec
RG	287
DW	50.000 usec
DE	6.00 usec
TE	295.8 K
D1	2.00000000 sec
TD0	1

======== CHANNEL f1 ========

NUC1	1H
P1	13.00 usec
PL1	2.00 dB
SFO1	500.0335010 MHz
SI	16384
SF	500.0300207 MHz
WDW	EM
SSB	0
LB	0.30 Hz
GB	0
PC	1.00

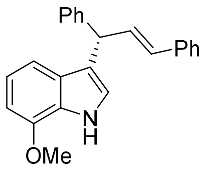

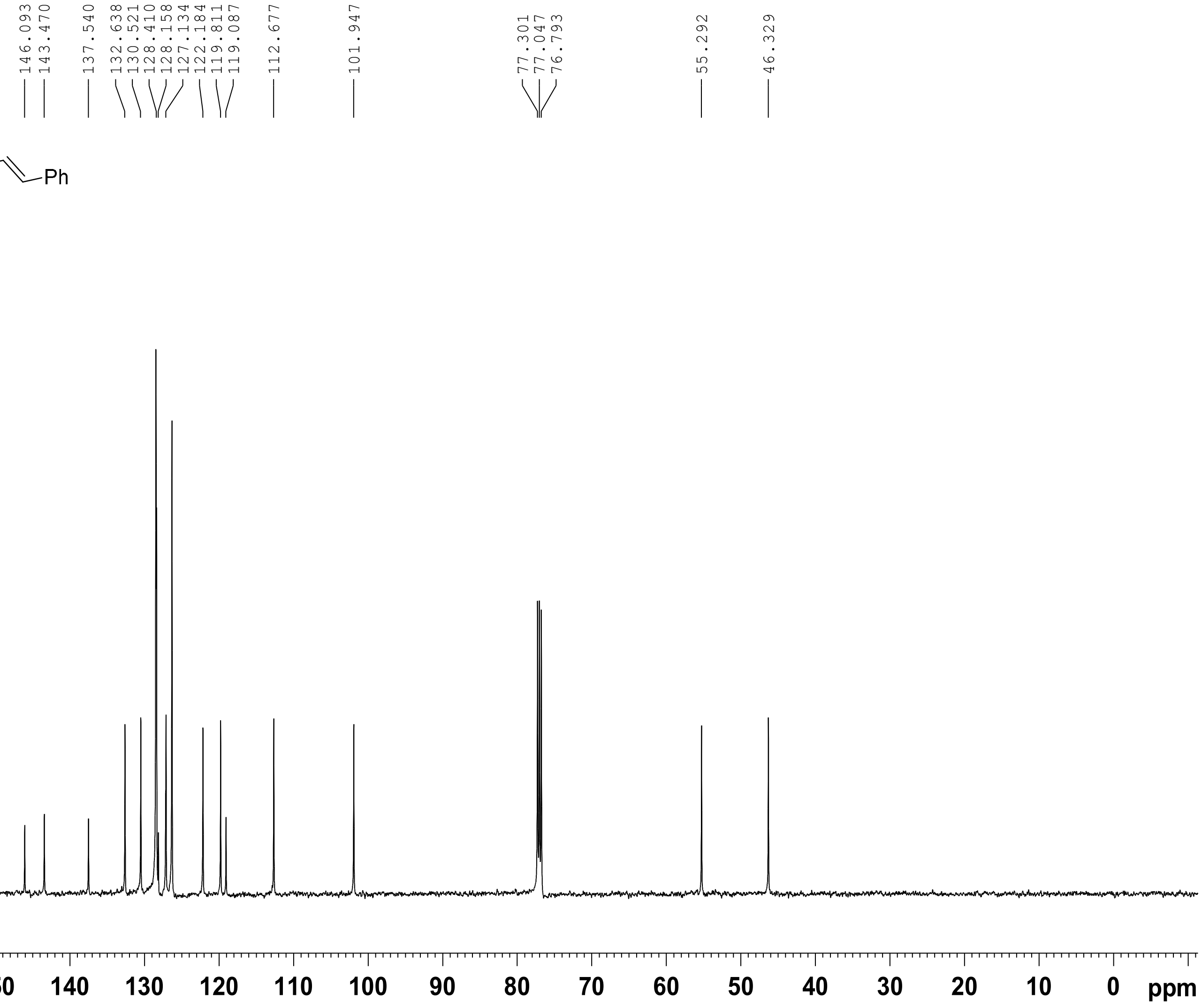

NAME	QZX-5-50-12
EXPNO	2
PROCNO	1
Date_	20181016
Time	16.55
INSTRUM	spect
PROBHD	5 mm PADUL 13C
PULPROG	zgpg30
TD	65536
SOLVENT	CDCl3
NS	241
DS	2
SWH	32679.738 Hz
FIDRES	0.498653 Hz
AQ	1.0027661 sec
RG	8200
DW	15.300 usec
DE	6.00 usec
TE	297.8 K
D1	2.00000000 sec
d11	0.03000000 sec
DELTA	1.89999998 sec
TD0	10

======== CHANNEL f1 ========

NUC1	13C
P1	12.20 usec
PL1	3.00 dB
SFO1	125.7464750 MHz

======== CHANNEL f2 ========

CPDPRG2	waltz16
NUC2	1H
PCPD2	80.00 usec
PL2	2.00 dB
PL12	17.70 dB
PL13	17.70 dB
SFO2	500.0355000 MHz
SI	32768
SF	125.7326504 MHz
WDW	EM
SSB	0
LB	6.00 Hz
GB	0
PC	2.00

(*S*,*E*)-7-chloro-3-(1,3-diphenylallyl)-1H-indole **(3o)**

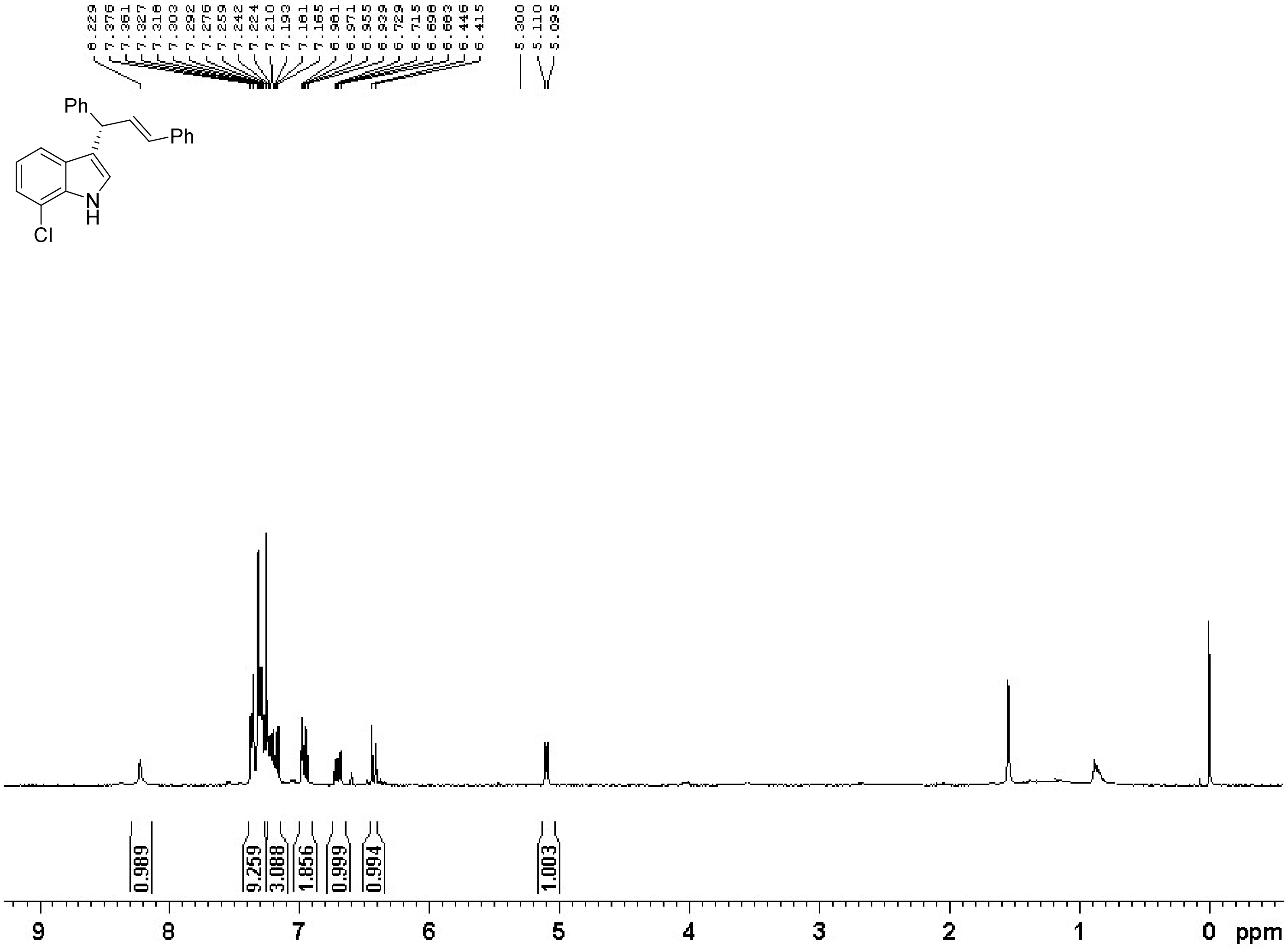

NAME	QZX-5-42-11
EXPNO	1
PROCNO	1
Date_	20181011
Time	15.58
INSTRUM	spect
PROBHD	5 mm PADUL 13C
PULPROG	zg30
TD	16384
SOLVENT	CDCl3
NS	8
DS	1
SWH	10000.000 Hz
FIDRES	0.610352 Hz
AQ	0.8193000 sec
RG	575
DW	50.000 usec
DE	6.00 usec
TE	296.2 K
D1	2.00000000 sec
TD0	1

======== CHANNEL f1 ========

NUC1	1H
P1	13.00 usec
PL1	2.00 dB
SFO1	500.0335010 MHz
SI	16384
SF	500.0300101 MHz
WDW	EM
SSB	0
LB	0.30 Hz
GB	0
PC	1.00

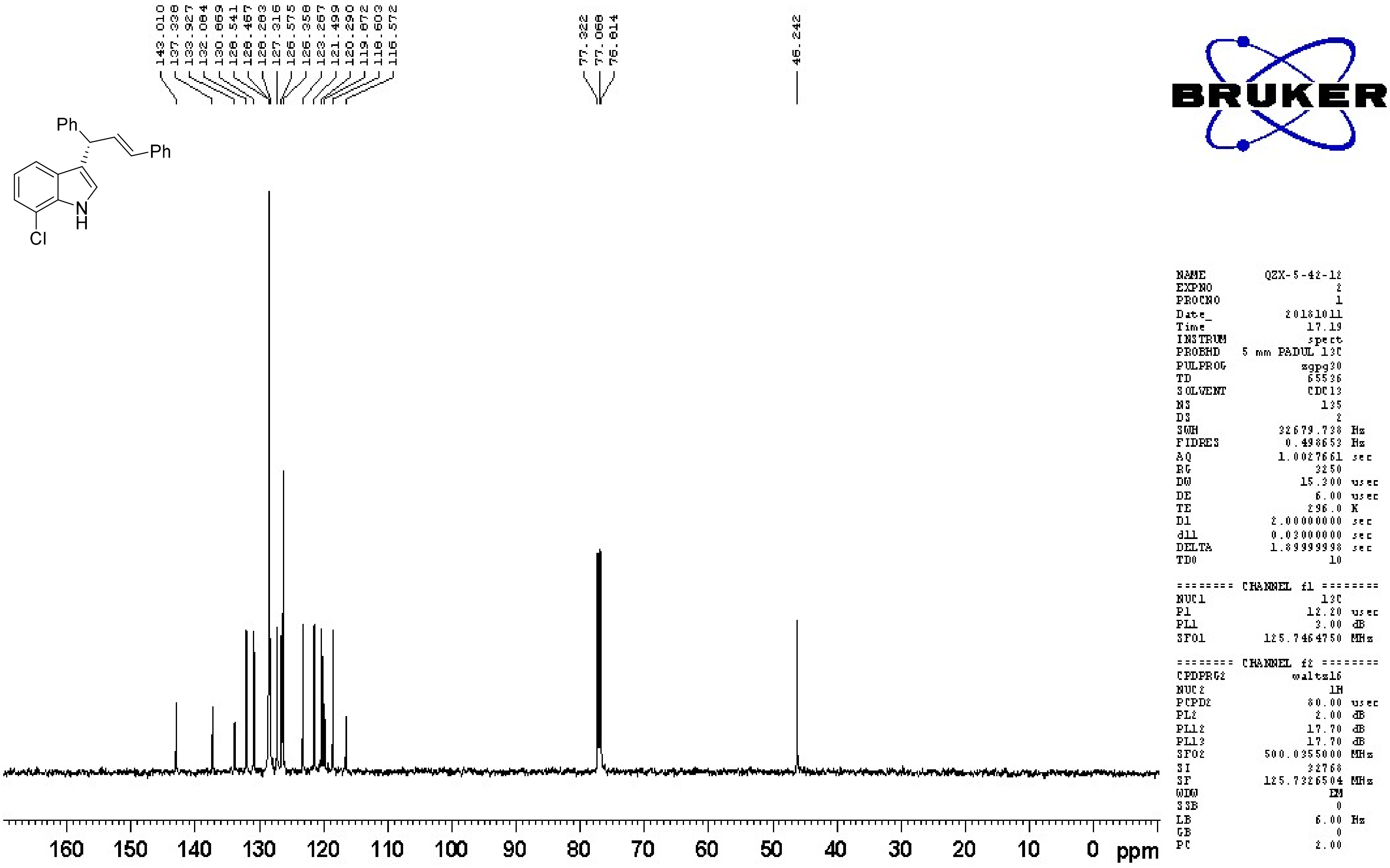
143.010
137.338
133.927
132.084
130.869
128.541
128.467
128.283
127.316
126.575
126.358
123.267
121.499
120.290
119.872
118.603
116.572
77.322
77.068
76.814
46.242
Ph
Ph
N
H
Cl
BRUKER
160 150 140 130 120 110 100 90 80 70 60 50 40 30 20 10 0 ppm
NAME QZX-5-42-12
EXPNO 2
PROCNO 1
Date_ 20181011
Time 17.19
INSTRUM spect
PROBHD 5 mm PADUL 13C
PULPROG zgpg30
TD 65536
SOLVENT CDCl3
NS 135
DS 2
SWH 32679.738 Hz
FIDRES 0.498653 Hz
AQ 1.0027661 sec
RG 3250
DW 15.300 usec
DE 6.00 usec
TE 296.0 K
D1 2.00000000 sec
d11 0.03000000 sec
DELTA 1.89999998 sec
TD0 10
======== CHANNEL f1 ========
NUC1 13C
P1 12.20 usec
PL1 3.00 dB
SFO1 125.7464750 MHz
======== CHANNEL f2 ========
CPDPRG2 waltz16
NUC2 1H
PCPD2 80.00 usec
PL2 2.00 dB
PL12 17.70 dB
PL13 17.70 dB
SFO2 500.0355000 MHz
SI 32768
SF 125.7326504 MHz
WDW EM
SSB 0
LB 6.00 Hz
GB 0
PC 2.00

(*S*,*E*)-3-(1,3-di-p-tolylallyl)-1H-indole **(3p)**

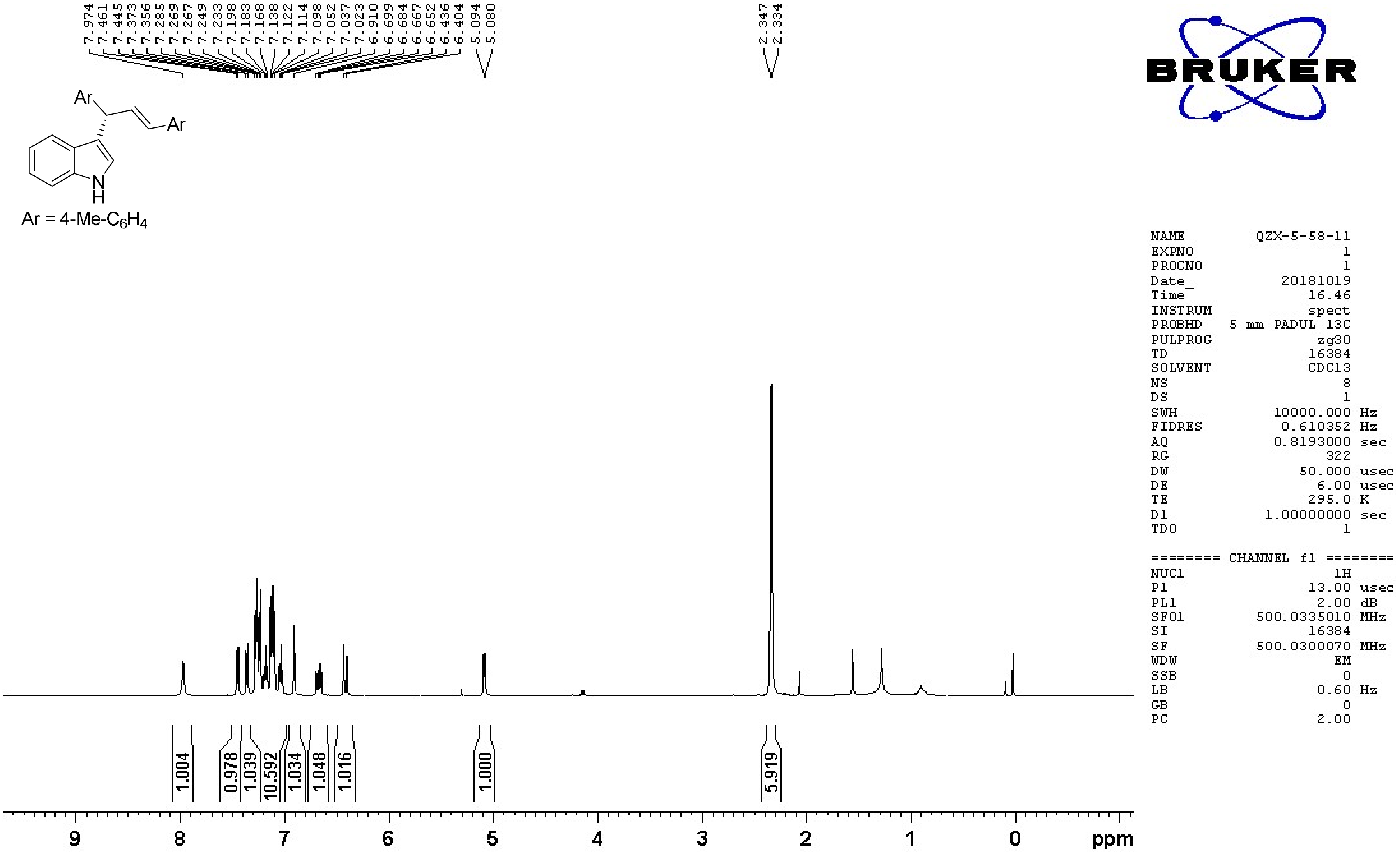

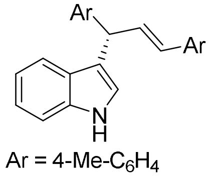
Ar
Ar
N
H
Ar = 4-Me-C6H4

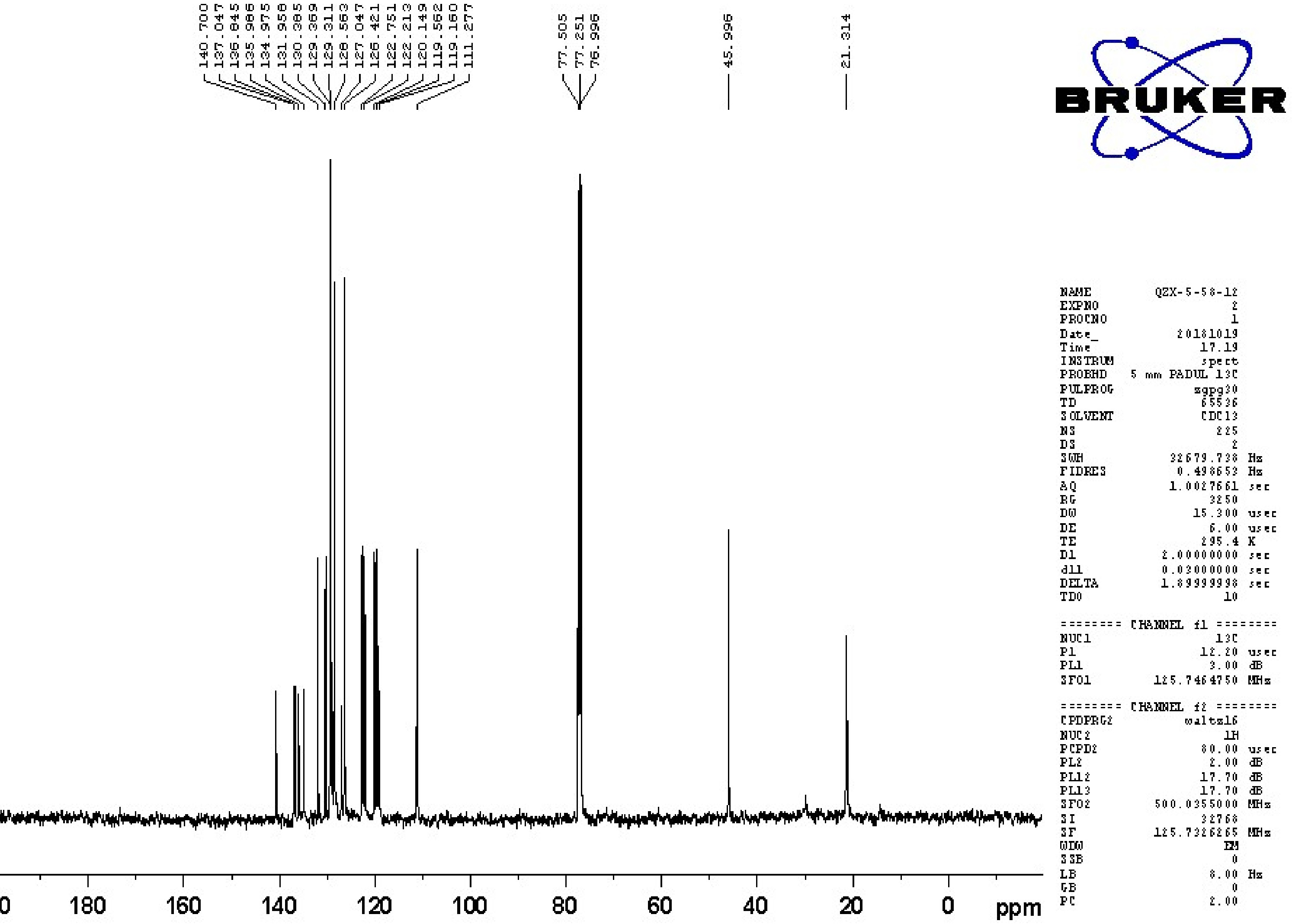

(*S,E*)-3-(1,3-bis(4-methoxyphenyl)allyl)-1H-indole **(3q)**

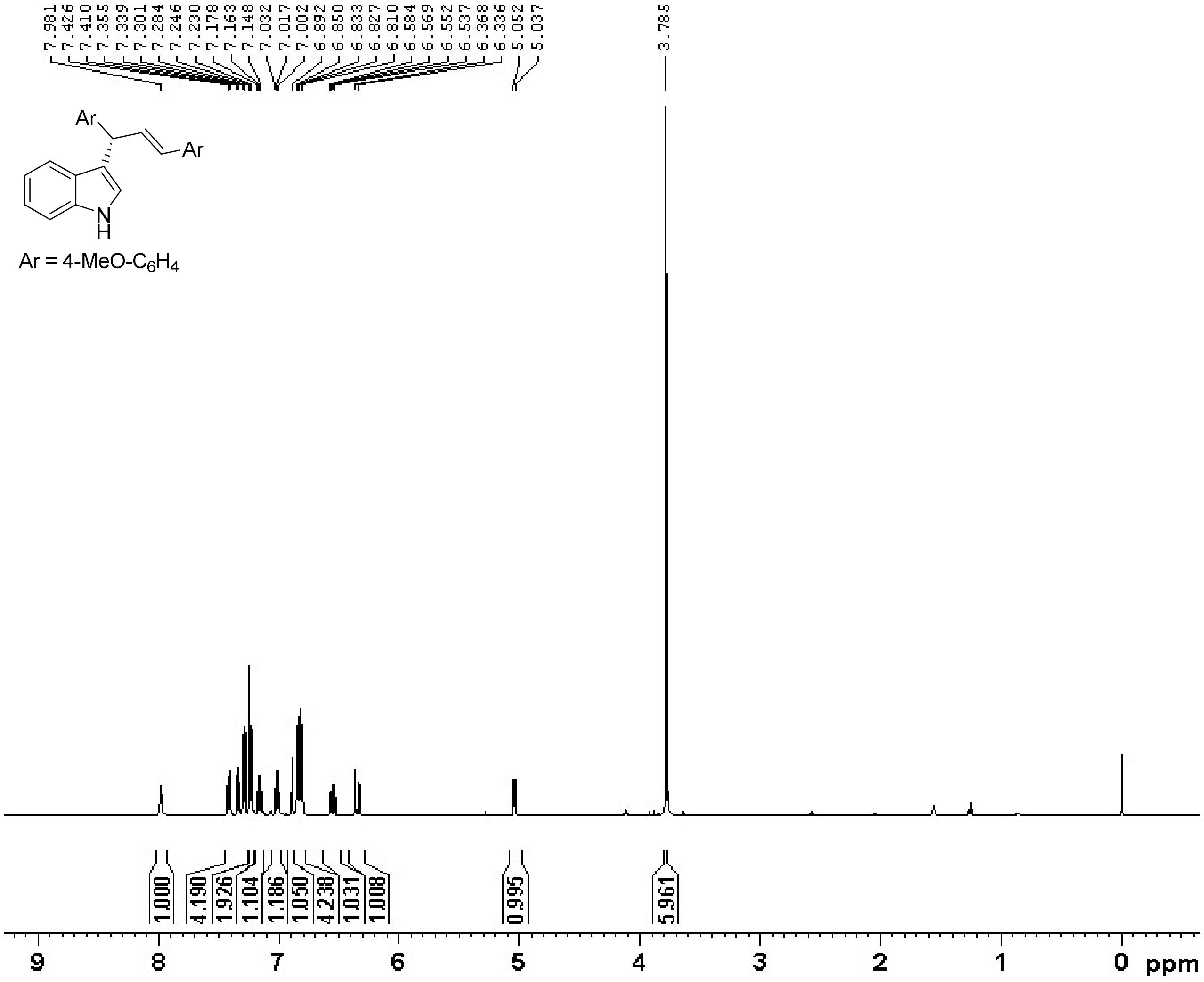

NAME	QZX-5-194-1
EXPNO	1
PROCNO	1
Date_	20190410
Time	9.19
INSTRUM	spect
PROBHD	5 mm PABBO BB-
PULPROG	zg30
TD	16384
SOLVENT	CDCl3
NS	8
DS	0
SWH	10000.000 Hz
FIDRES	0.610352 Hz
AQ	0.8193000 sec
RG	456
DW	50.000 usec
DE	8.00 usec
TE	292.3 K
D1	2.00000000 sec
TD0	1

======== CHANNEL f1 ========

NUC1	1H
P1	13.00 usec
PL1	2.00 dB
SFO1	500.0335000 MHz
SI	16384
SF	500.0300171 MHz
WDW	EM
SSB	0
LB	0.30 Hz
GB	0
PC	1.00

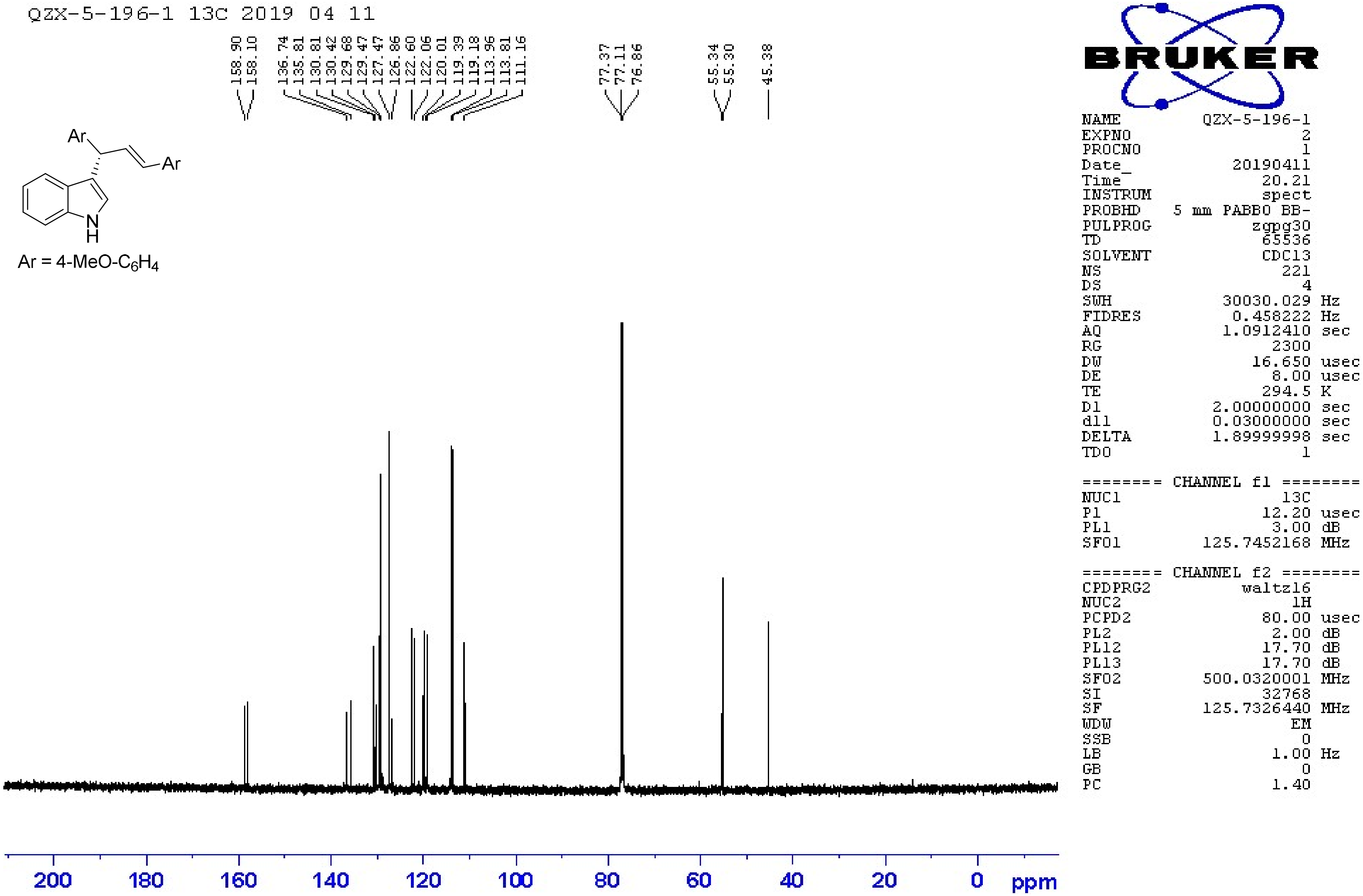
QZX-5-196-1 13C 2019 04 11
158.90
158.10
136.74
135.81
130.81
130.42
129.68
129.47
127.47
126.86
122.60
122.06
120.01
119.39
119.18
113.96
113.81
111.16
77.37
77.11
76.86
55.34
55.30
45.38
Ar
Ar
N
H
Ar = 4-MeO-C6H4
200
180
160
140
120
100
80
60
40
20
0
ppm
BRUKER
NAME QZX-5-196-1
EXPNO 2
PROCNO 1
Date_ 20190411
Time 20.21
INSTRUM spect
PROBHD 5 mm PABBO BB-
PULPROG zgpg30
TD 65536
SOLVENT CDCl3
NS 221
DS 4
SWH 30030.029 Hz
FIDRES 0.458222 Hz
AQ 1.0912410 sec
RG 2300
DW 16.650 usec
DE 8.00 usec
TE 294.5 K
D1 2.00000000 sec
d11 0.03000000 sec
DELTA 1.89999998 sec
TD0 1
======== CHANNEL f1 ========
NUC1 13C
P1 12.20 usec
PL1 3.00 dB
SFO1 125.7452168 MHz
======== CHANNEL f2 ========
CPDPRG2 waltz16
NUC2 1H
PCPD2 80.00 usec
PL2 2.00 dB
PL12 17.70 dB
PL13 17.70 dB
SFO2 500.0320001 MHz
SI 32768
SF 125.7326440 MHz
WDW EM
SSB 0
LB 1.00 Hz
GB 0
PC 1.40

(*S*,*E*)-3-(1,3-bis(4-chlorophenyl)allyl)-1H-indole **(3r)**

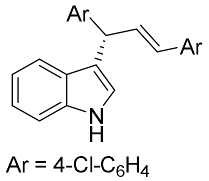

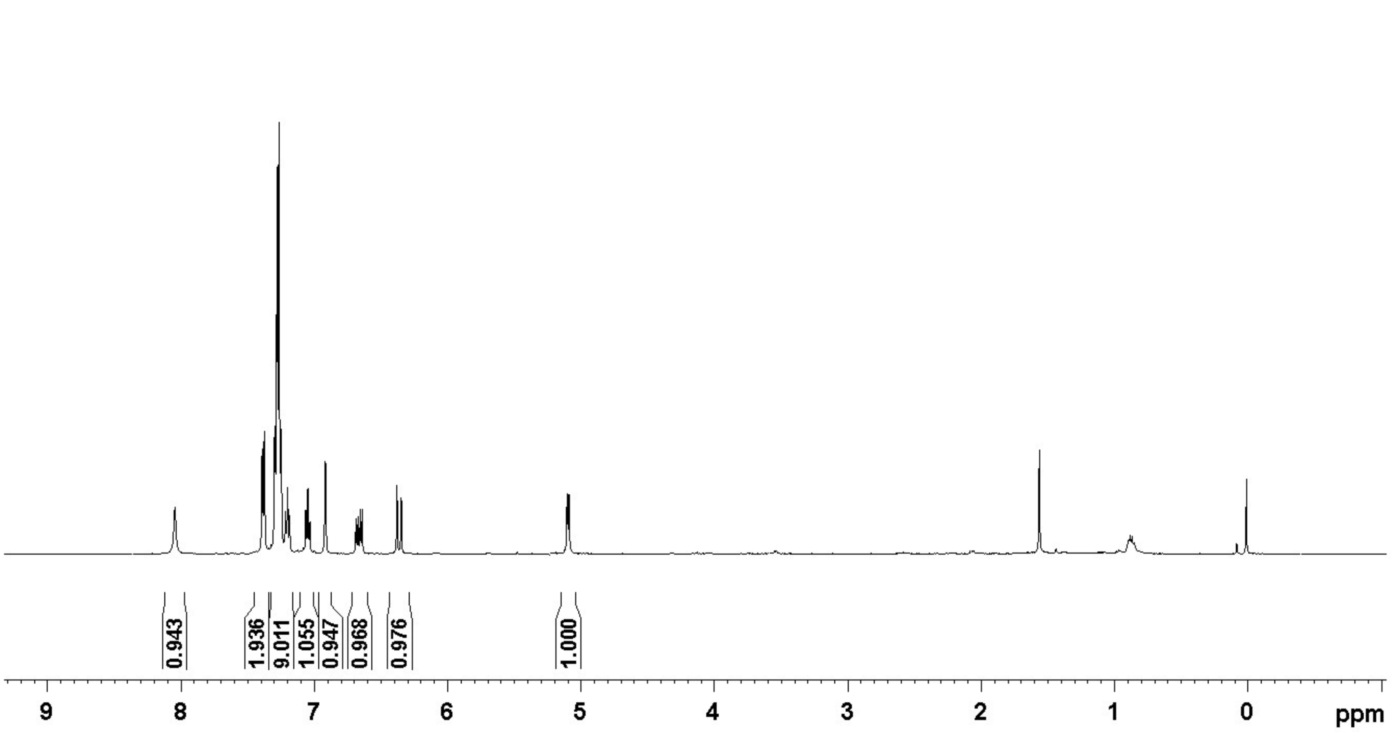

NAME	QZX-5-57-11
EXPNO	1
PROCNO	1
Date_	20181019
Time	16.44
INSTRUM	spect
PROBHD	5 mm PADUL 13C
PULPROG	zg30
TD	16384
SOLVENT	CDCl3
NS	8
DS	1
SWH	10000.000 Hz
FIDRES	0.610352 Hz
AQ	0.8193000 sec
RG	322
DW	50.000 usec
DE	6.00 usec
TE	295.0 K
D1	1.00000000 sec
TD0	1

======== CHANNEL f1 ========

NUC1	1H
P1	13.00 usec
PL1	2.00 dB
SFO1	500.0335010 MHz
SI	16384
SF	500.0300070 MHz
WDW	EM
SSB	0
LB	0.60 Hz
GB	0
PC	2.00

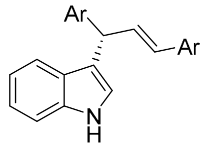

Ar = 4-Cl-C_6H_4

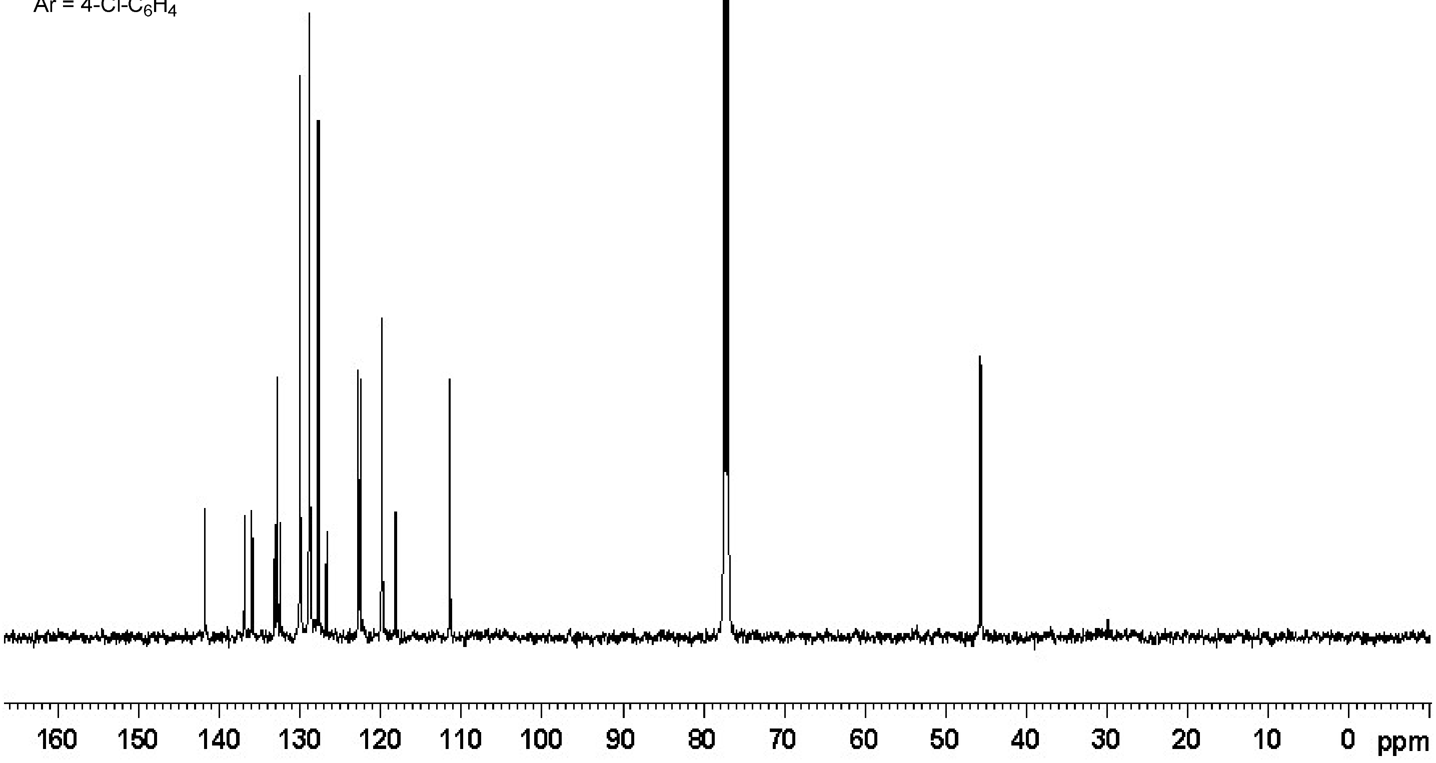

BRUKER

NAME	QZX-5-57-12	
EXPNO	2	
PROCNO	1	
Date_	20181019	
Time	17.47	
INSTRUM	spect	
PROBHD	5 mm PADUL 13C	
PULPROG	zgpg30	
TD	65536	
SOLVENT	CDCl3	
NS	372	
DS	2	
SWH	32679.738	Hz
FIDRES	0.498653	Hz
AQ	1.0027661	sec
RG	3250	
DW	15.300	usec
DE	6.00	usec
TE	295.1	K
D1	2.00000000	sec
d11	0.03000000	sec
DELTA	1.89999998	sec
TD0	10	
======== CHANNEL f1 ========		
NUC1	13C	
P1	12.20	usec
PL1	3.00	dB
SFO1	125.7464750	MHz
======== CHANNEL f2 ========		
CPDPRG2	waltz16	
NUC2	1H	
PCPD2	80.00	usec
PL2	2.00	dB
PL12	17.70	dB
PL13	17.70	dB
SFO2	500.0355000	MHz
SI	32768	
SF	125.7326195	MHz
WDW	EM	
SSB	0	
LB	6.00	Hz
GB	0	
PC	2.00	

(*S,E*)-3-(1,3-bis(4-nitrophenyl)allyl)-1H-indole **(3s)**

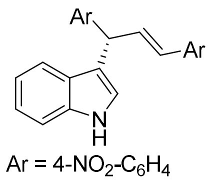

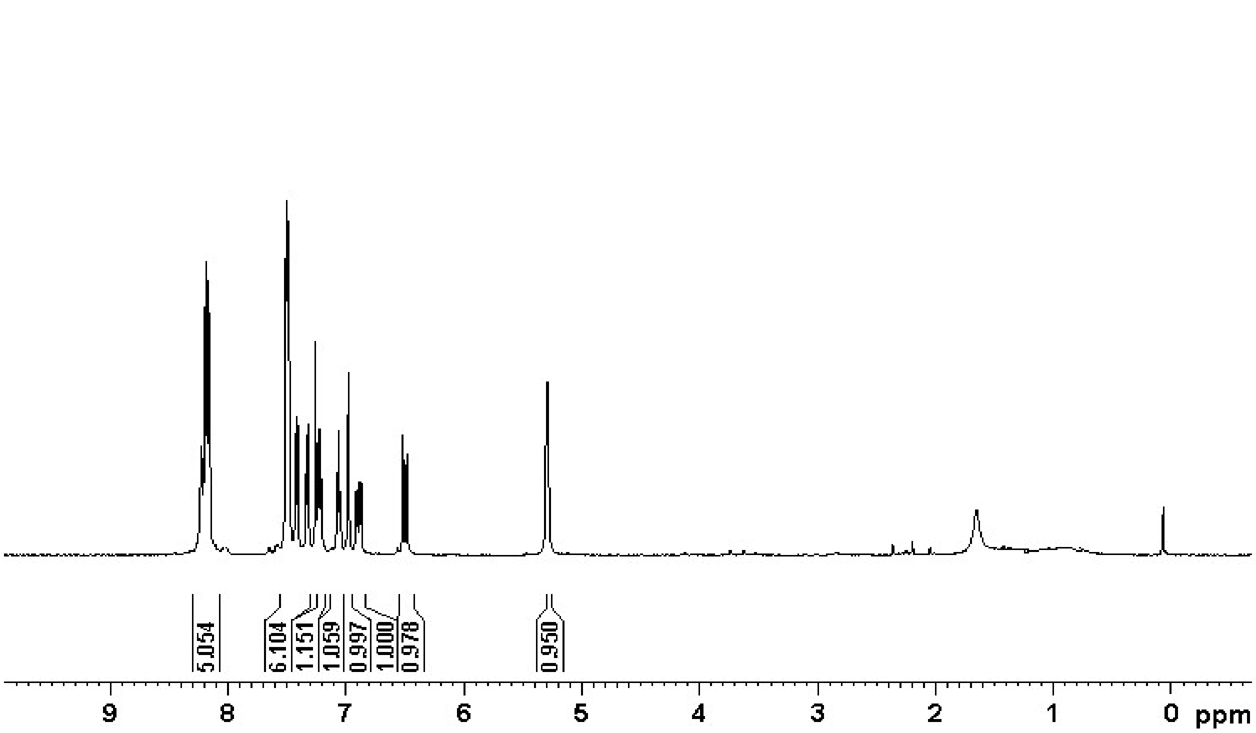

NAME	QZX-5-197-1	
EXPNO	1	
PROCNO	1	
Date_	20190412	
Time	10.22	
INSTRUM	spect	
PROBHD	5 mm PABBO BB-	
PULPROG	zg30	
TD	16384	
SOLVENT	CDCl3	
NS	8	
DS	0	
SWH	10000.000	Hz
FIDRES	0.610352	Hz
AQ	0.8193000	sec
RG	456	
DW	50.000	usec
DE	8.00	usec
TE	293.1	K
D1	2.00000000	sec
TD0	1	
======== CHANNEL f1 ========		
NUC1	1H	
P1	13.00	usec
PL1	2.00	dB
SFO1	500.0335000	MHz
SI	16384	
SF	500.0300101	MHz
WDW	EM	
SSB	0	
LB	0.30	Hz
GB	0	
PC	1.00	

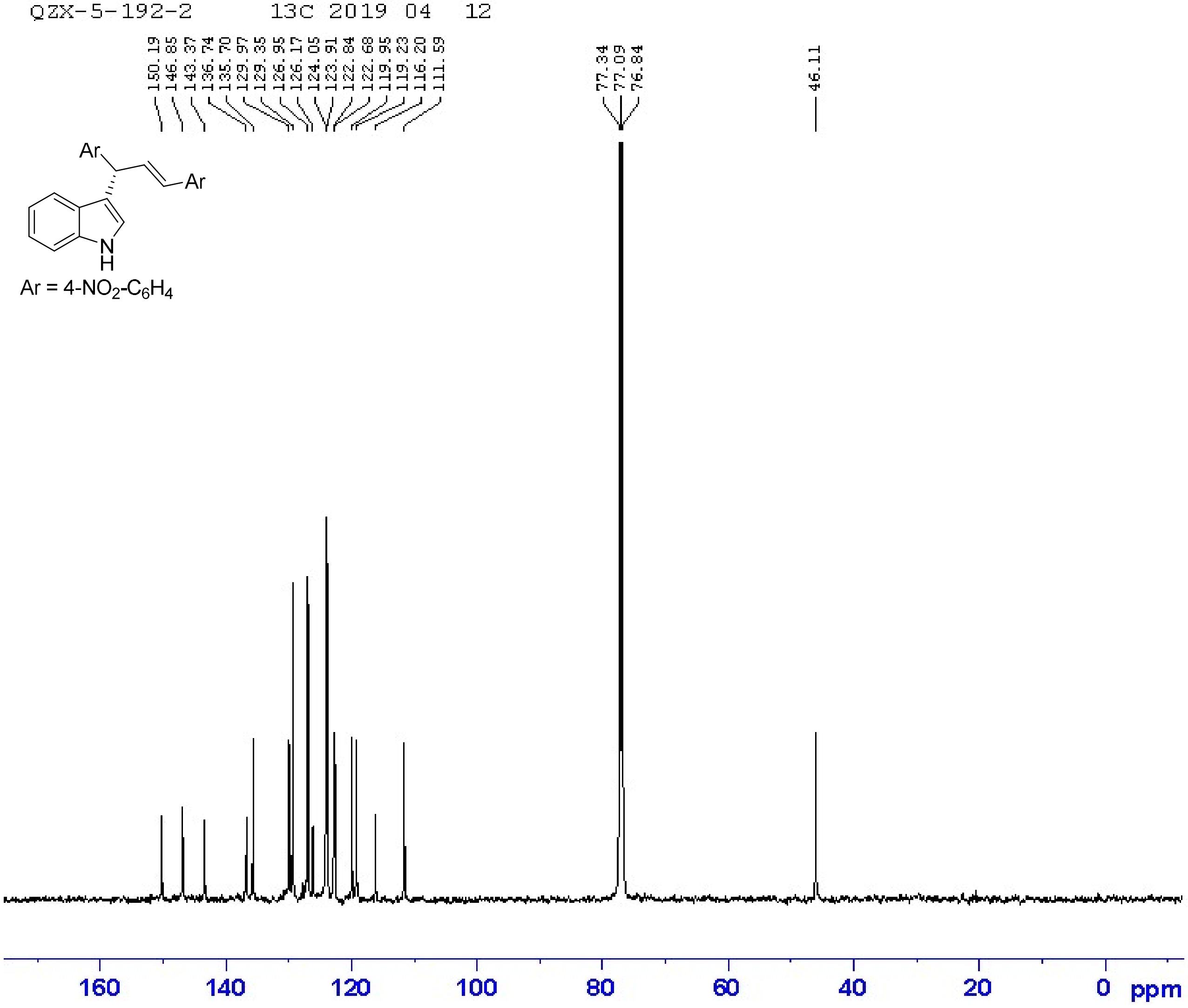

BRUKER

```
NAME          QZX-5-192-2
EXPNO                   2
PROCNO                  1
Date_            20190412
Time                15.41
INSTRUM             spect
PROBHD   5 mm PABBO BB-
PULPROG           zgpg30
TD                  65536
SOLVENT             CDCl3
NS                    671
DS                      0
SWH             30030.029 Hz
FIDRES           0.458222 Hz
AQ              1.0912410 sec
RG                   9200
DW                 16.650 usec
DE                   8.00 usec
TE                  294.8 K
D1             2.00000000 sec
d11            0.03000000 sec
DELTA          1.89999998 sec
TD0                     4

======== CHANNEL f1 ========
NUC1                  13C
P1                  12.20 usec
PL1                  3.00 dB
SFO1          125.7452170 MHz

======== CHANNEL f2 ========
CPDPRG2           waltz16
NUC2                   1H
PCPD2               80.00 usec
PL2                  2.00 dB
PL12                17.70 dB
PL13                17.70 dB
SFO2          500.0320000 MHz
SI                  32768
SF            125.7326440 MHz
WDW                    EM
SSB                     0
LB                   8.00 Hz
GB                      0
PC                   1.40
```

(*S,E*)-*tert*-butyl 3-(1,3-diphenylallyl)-1H-indole-1-carboxylate **(Boc-3a)**

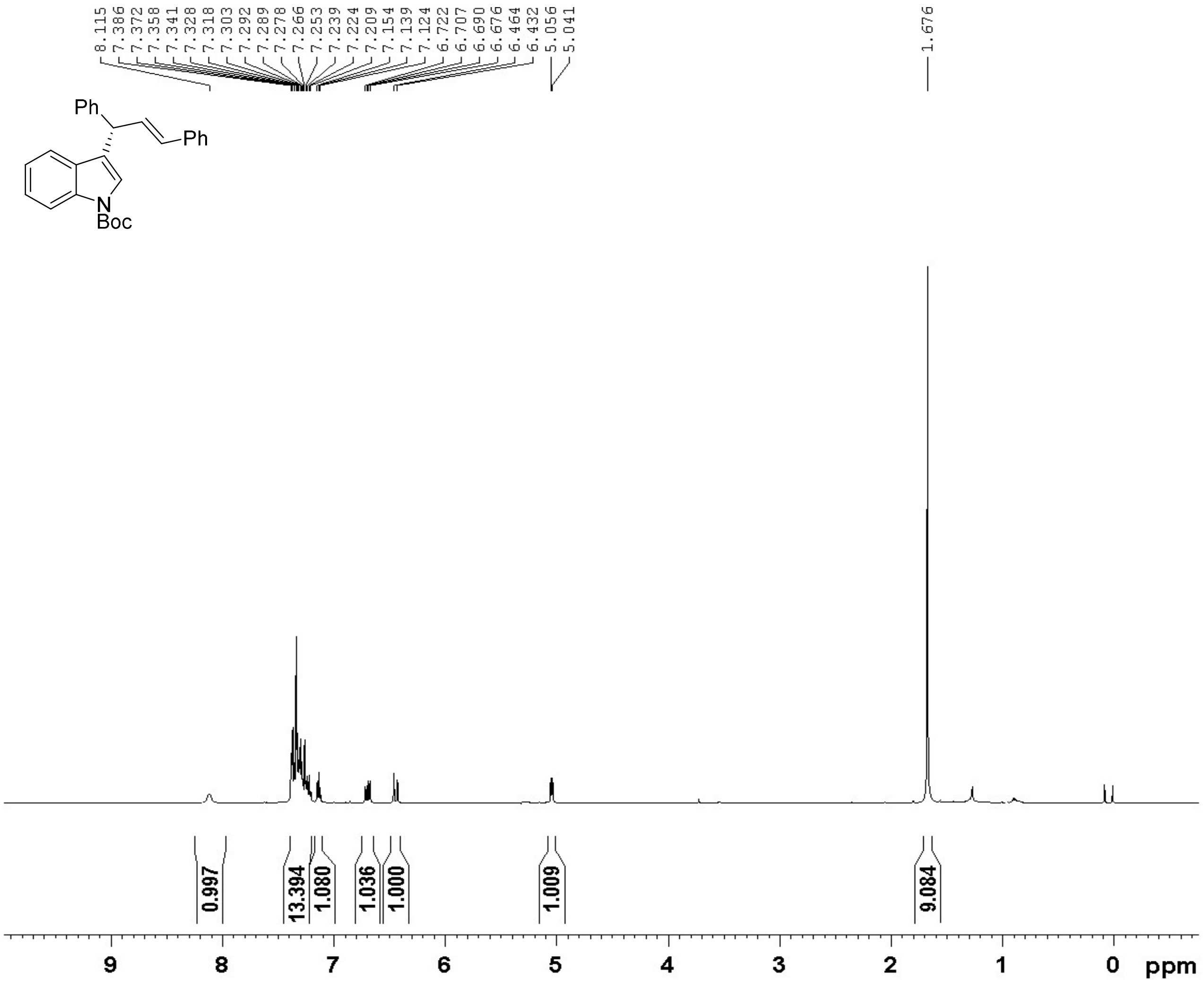

NAME	QZX-5-5-21
EXPNO	1
PROCNO	1
Date_	20181030
Time	16.37
INSTRUM	spect
PROBHD	5 mm PADUL 13C
PULPROG	zg30
TD	16384
SOLVENT	CDCl3
NS	8
DS	1
SWH	10000.000 Hz
FIDRES	0.610352 Hz
AQ	0.8193000 sec
RG	228
DW	50.000 usec
DE	6.00 usec
TE	298.2 K
D1	1.00000000 sec
TD0	1

======== CHANNEL f1 ========

NUC1	1H
P1	13.00 usec
PL1	2.00 dB
SFO1	500.0335010 MHz
SI	16384
SF	500.0300070 MHz
WDW	EM
SSB	0
LB	0.90 Hz
GB	0
PC	2.00

QZX-5-5   13C   2018 09 14

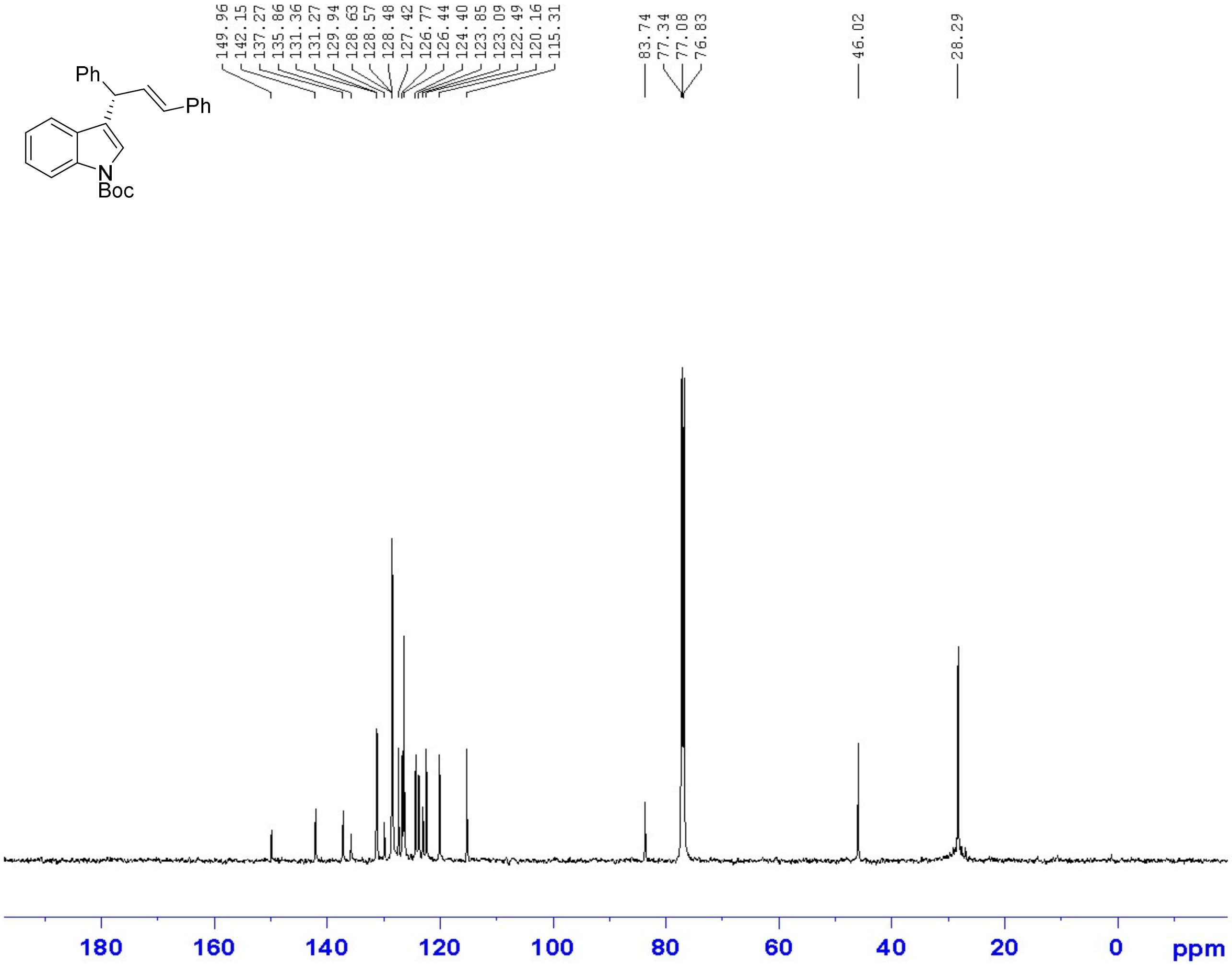

BRUKER

NAME	QZX-5-5
EXPNO	2
PROCNO	1
Date_	20180914
Time	15.53
INSTRUM	spect
PROBHD	5 mm PADUL 13C
PULPROG	zgpg30
TD	65536
SOLVENT	CDCl3
NS	449
DS	1
SWH	32679.738 Hz
FIDRES	0.498653 Hz
AQ	1.0027661 sec
RG	9200
DW	15.300 usec
DE	6.00 usec
TE	298.9 K
D1	2.00000000 sec
d11	0.03000000 sec
DELTA	1.89999998 sec
TD0	20

======== CHANNEL f1 ========

NUC1	13C
P1	12.20 usec
PL1	3.00 dB
SFO1	125.7464750 MHz

======== CHANNEL f2 ========

CPDPRG2	waltz16
NUC2	1H
PCPD2	80.00 usec
PL2	2.00 dB
PL12	17.70 dB
PL13	17.70 dB
SFO2	500.0355000 MHz
SI	32768
SF	125.7326392 MHz
WDW	EM
SSB	0
LB	10.00 Hz
GB	0
PC	1.00

(*S,E*)-*tert*-butyl 3-(1,3-diphenylallyl)-2-methyl-1H-indole-1-carboxylate **(Boc-3b)**

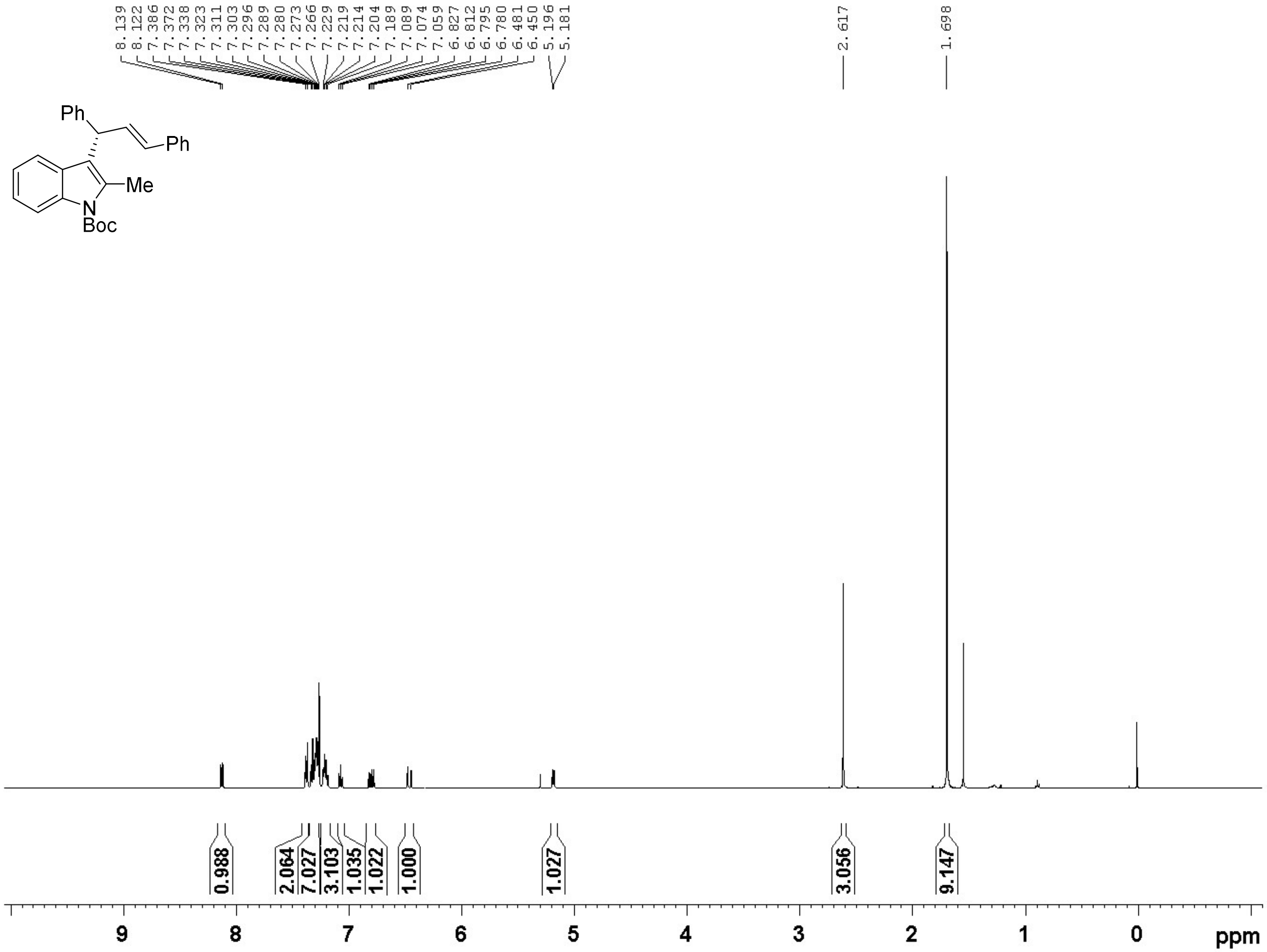

NAME	QZX-5-52-21
EXPNO	1
PROCNO	1
Date_	20181026
Time	14.32
INSTRUM	spect
PROBHD	5 mm PADUL 13C
PULPROG	zg30
TD	16384
SOLVENT	CDCl3
NS	8
DS	1
SWH	10000.000 Hz
FIDRES	0.610352 Hz
AQ	0.8193000 sec
RG	512
DW	50.000 usec
DE	6.00 usec
TE	298.1 K
D1	1.00000000 sec
TD0	1
======== CHANNEL f1 ========	
NUC1	1H
P1	13.00 usec
PL1	2.00 dB
SFO1	500.0335010 MHz
SI	16384
SF	500.0300070 MHz
WDW	EM
SSB	0
LB	0.30 Hz
GB	0
PC	2.00

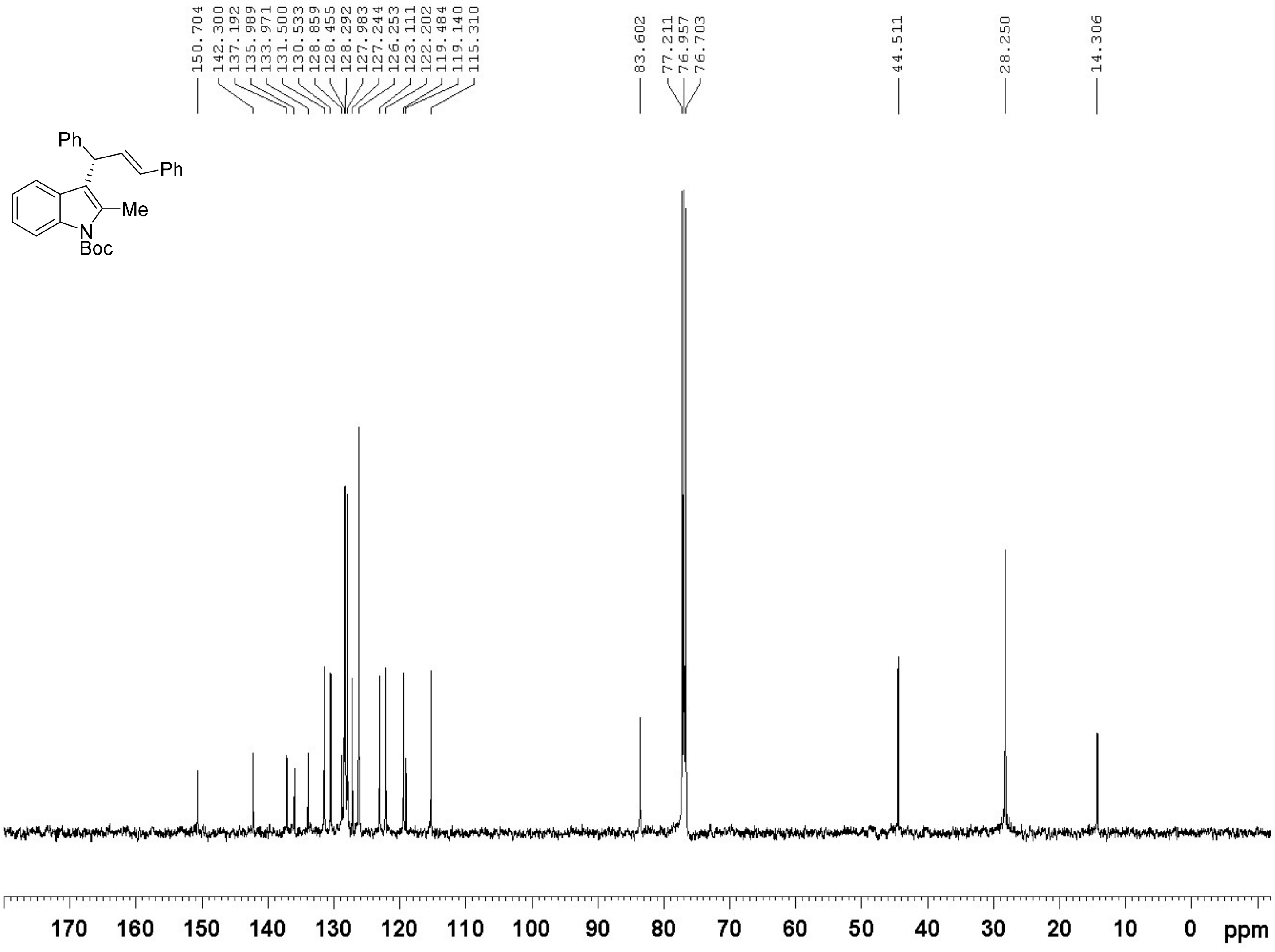


```
NAME          QZX-5-52-22
EXPNO                   2
PROCNO                  1
Date_            20181026
Time                17.31
INSTRUM             spect
PROBHD   5 mm PADUL 13C
PULPROG           zgpg30
TD                  65536
SOLVENT             CDCl3
NS                    257
DS                      1
SWH             32679.738 Hz
FIDRES           0.498653 Hz
AQ              1.0027661 sec
RG                   7290
DW                 15.300 usec
DE                   6.00 usec
TE                  297.0 K
D1             2.00000000 sec
d11            0.03000000 sec
DELTA          1.89999998 sec
TD0                    10

======== CHANNEL f1 ========
NUC1                  13C
P1                  12.20 usec
PL1                  3.00 dB
SFO1          125.7464750 MHz

======== CHANNEL f2 ========
CPDPRG2           waltz16
NUC2                   1H
PCPD2               80.00 usec
PL2                  2.00 dB
PL12                17.70 dB
PL13                17.70 dB
SFO2          500.0355000 MHz
SI                  32768
SF            125.7326548 MHz
WDW                    EM
SSB                     0
LB                   6.00 Hz
GB                      0
PC                   1.00
```

(*S,E*)-*tert*-butyl 3-(1,3-diphenylallyl)-2-phenyl-1H-indole-1-carboxylate **(Boc-3c)**

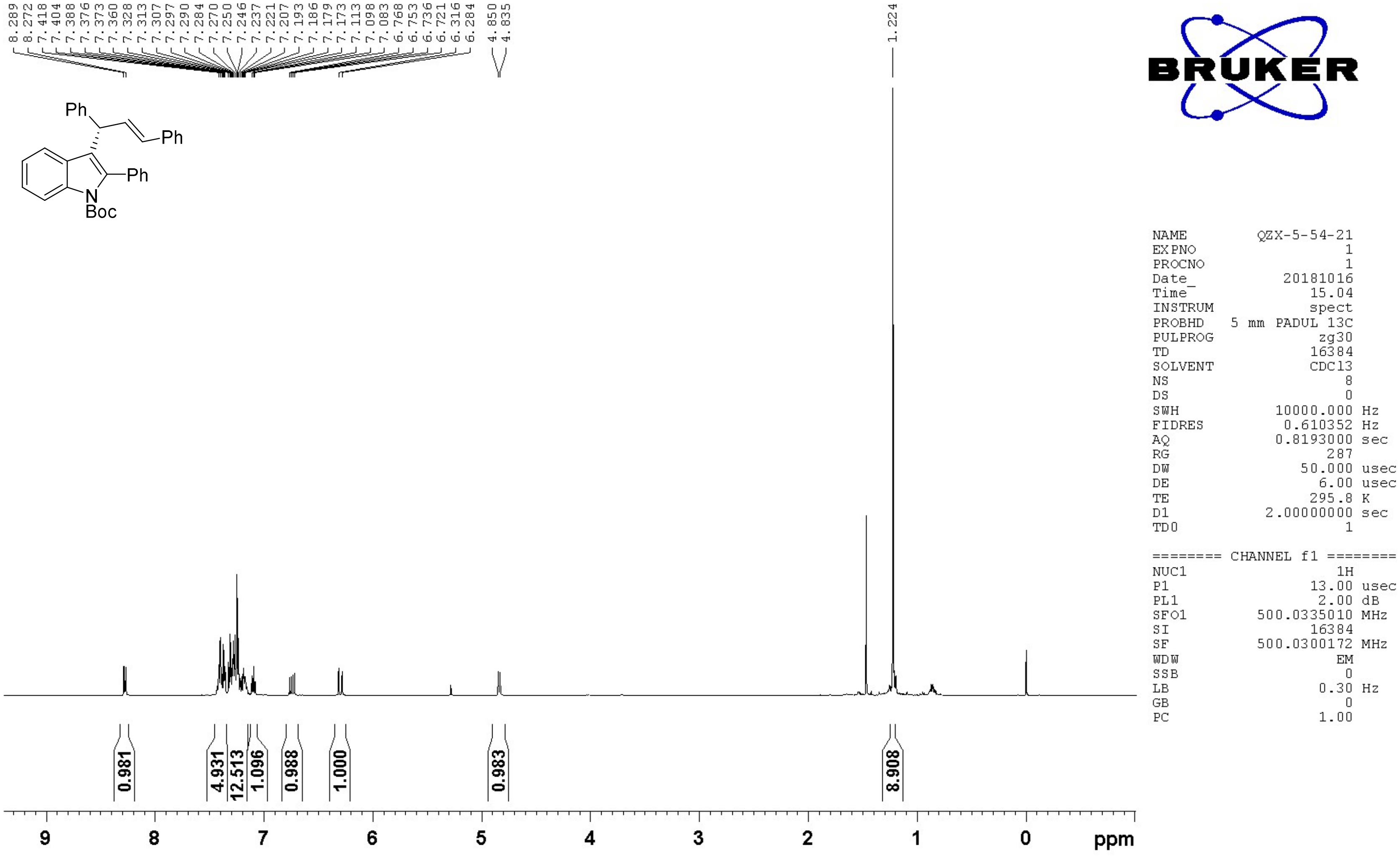

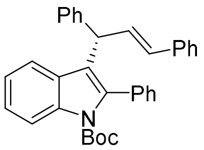

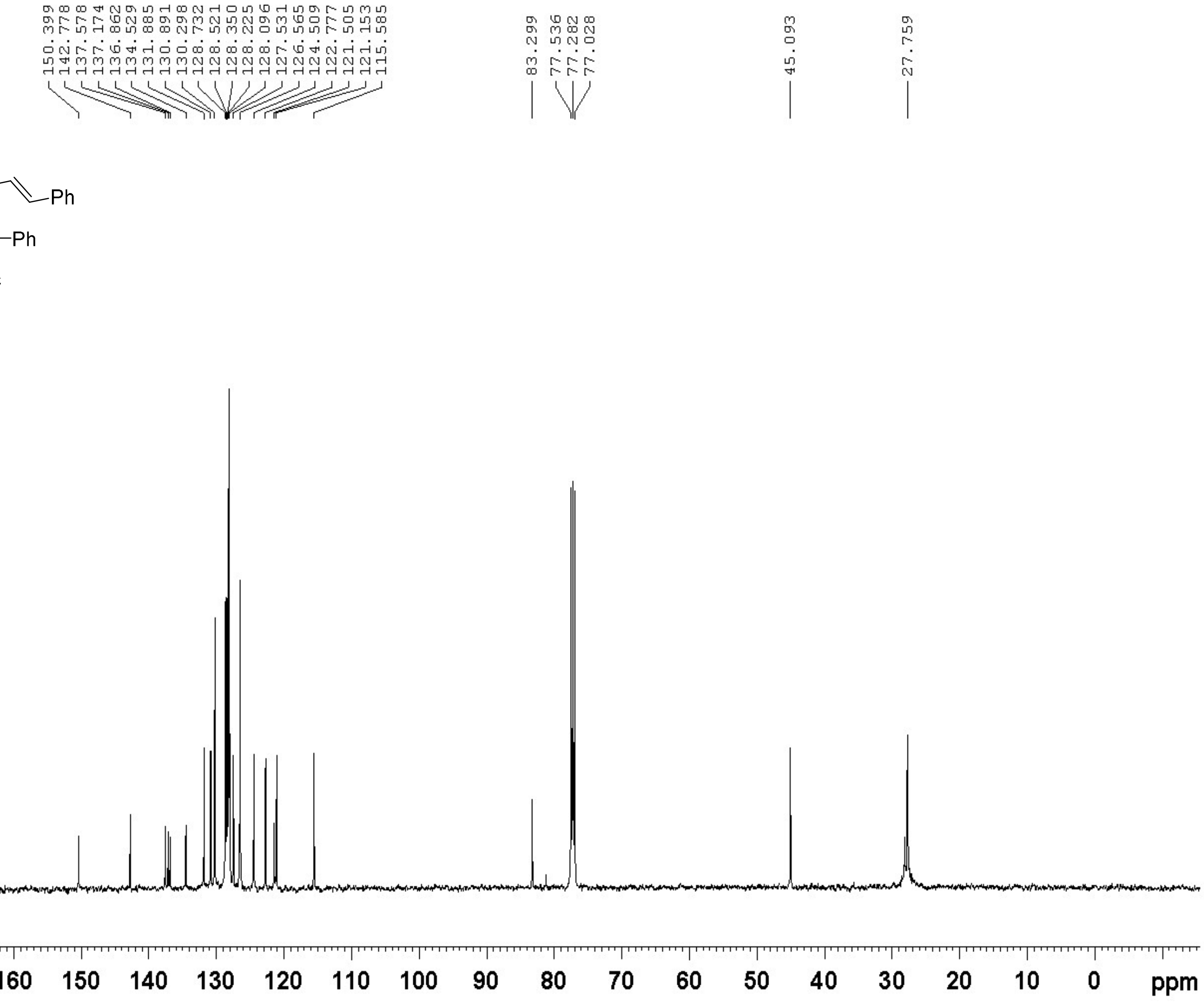

NAME	QZX-5-54-22
EXPNO	2
PROCNO	1
Date_	20181016
Time	19.59
INSTRUM	spect
PROBHD	5 mm PADUL 13C
PULPROG	zgpg30
TD	65536
SOLVENT	CDCl3
NS	455
DS	2
SWH	32679.738 Hz
FIDRES	0.498653 Hz
AQ	1.0027661 sec
RG	6500
DW	15.300 usec
DE	6.00 usec
TE	297.7 K
D1	2.00000000 sec
d11	0.03000000 sec
DELTA	1.89999998 sec
TD0	10
======== CHANNEL f1 ========	
NUC1	13C
P1	12.20 usec
PL1	3.00 dB
SFO1	125.7464750 MHz
======== CHANNEL f2 ========	
CPDPRG2	waltz16
NUC2	1H
PCPD2	80.00 usec
PL2	2.00 dB
PL12	17.70 dB
PL13	17.70 dB
SFO2	500.0355000 MHz
SI	32768
SF	125.7326147 MHz
WDW	EM
SSB	0
LB	6.00 Hz
GB	0
PC	2.00

(*S*,*E*)-*tert*-butyl 3-(1,3-diphenylallyl)-4-methyl-1H-indole-1-carboxylate **(Boc-3d)**

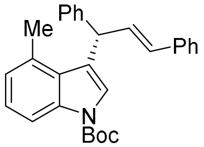

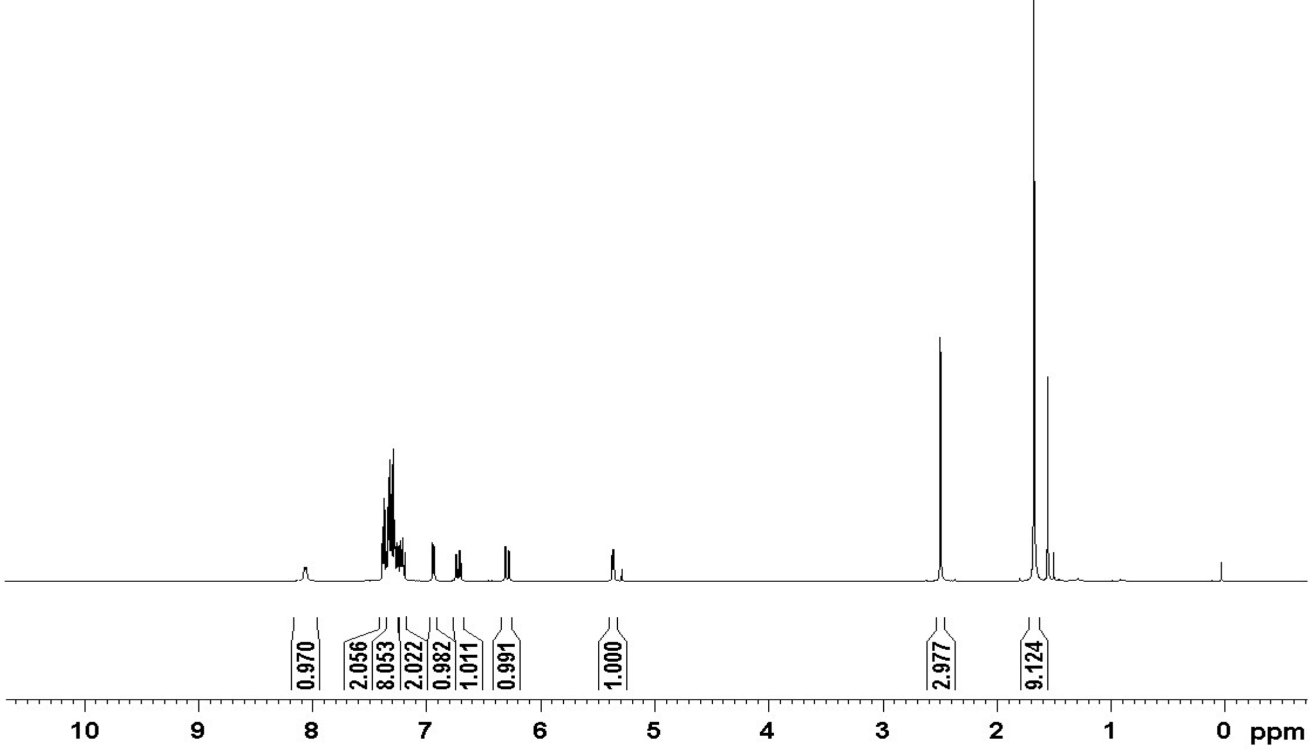

NAME	QZX-5-33-21
EXPNO	1
PROCNO	1
Date_	20180918
Time	16.23
INSTRUM	spect
PROBHD	5 mm PADUL 13C
PULPROG	zg30
TD	16384
SOLVENT	CDCl3
NS	8
DS	0
SWH	10000.000 Hz
FIDRES	0.610352 Hz
AQ	0.8193000 sec
RG	101
DW	50.000 usec
DE	8.00 usec
TE	296.5 K
D1	2.00000000 sec
TD0	1
======== CHANNEL f1 ========	
NUC1	1H
P1	13.00 usec
PL1	2.00 dB
SFO1	500.0335000 MHz
SI	16384
SF	500.0300132 MHz
WDW	EM
SSB	0
LB	0.30 Hz
GB	0
PC	1.00

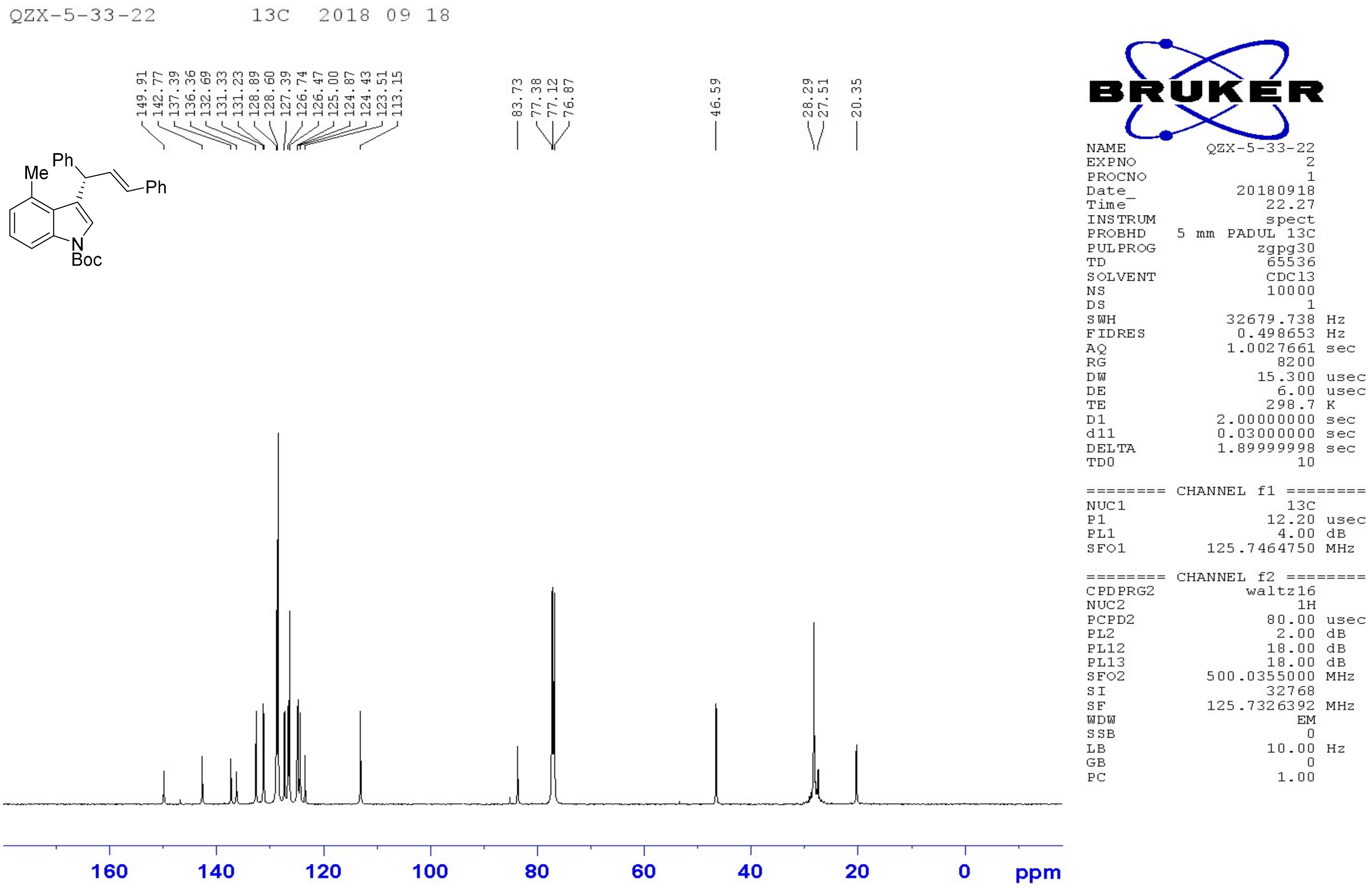
QZX-5-33-22 13C 2018 09 18
149.91
142.77
137.39
136.36
132.69
131.33
131.23
128.89
128.60
127.39
126.74
126.47
125.00
124.87
124.43
123.51
113.15
83.73
77.38
77.12
76.87
46.59
28.29
27.51
20.35
Me
Ph
Ph
N
Boc
BRUKER
NAME QZX-5-33-22
EXPNO 2
PROCNO 1
Date_ 20180918
Time 22.27
INSTRUM spect
PROBHD 5 mm PADUL 13C
PULPROG zgpg30
TD 65536
SOLVENT CDCl3
NS 10000
DS 1
SWH 32679.738 Hz
FIDRES 0.498653 Hz
AQ 1.0027661 sec
RG 8200
DW 15.300 usec
DE 6.00 usec
TE 298.7 K
D1 2.00000000 sec
d11 0.03000000 sec
DELTA 1.89999998 sec
TD0 10
======== CHANNEL f1 ========
NUC1 13C
P1 12.20 usec
PL1 4.00 dB
SFO1 125.7464750 MHz
======== CHANNEL f2 ========
CPDPRG2 waltz16
NUC2 1H
PCPD2 80.00 usec
PL2 2.00 dB
PL12 18.00 dB
PL13 18.00 dB
SFO2 500.0355000 MHz
SI 32768
SF 125.7326392 MHz
WDW EM
SSB 0
LB 10.00 Hz
GB 0
PC 1.00
160
140
120
100
80
60
40
20
0
ppm

(*S*,*E*)-*tert*-butyl 3-(1,3-diphenylallyl)-4-methoxy-1H-indole-1-carboxylate **(Boc-3e)**

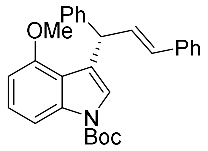

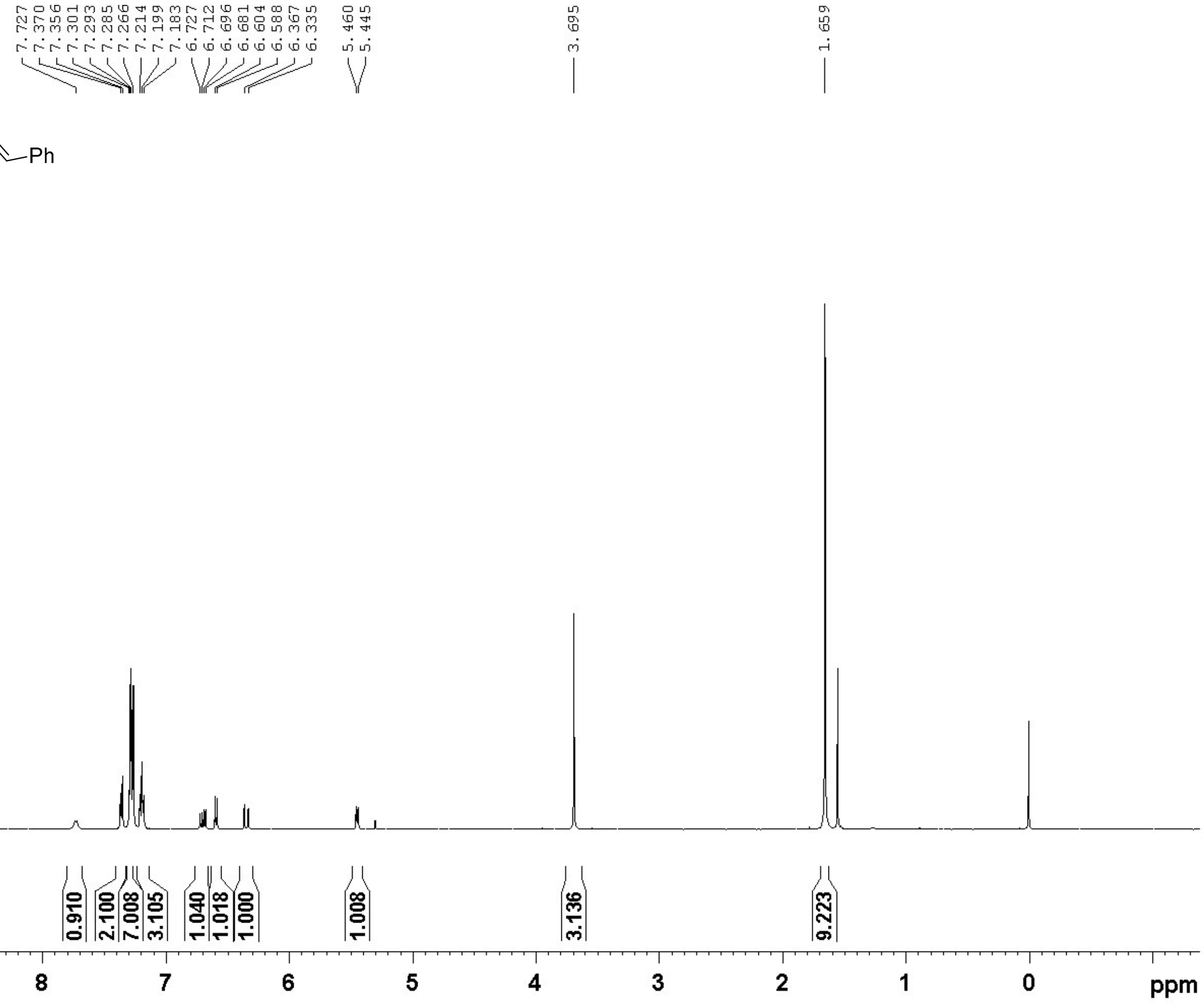


```
NAME          QZX-5-50-21
EXPNO                   1
PROCNO                  1
Date_            20181022
Time                14.21
INSTRUM             spect
PROBHD   5 mm PADUL 13C
PULPROG              zg30
TD                  16384
SOLVENT             CDCl3
NS                      8
DS                      1
SWH             10000.000 Hz
FIDRES           0.610352 Hz
AQ              0.8193000 sec
RG                    512
DW                 50.000 usec
DE                   6.00 usec
TE                  295.5 K
D1             1.00000000 sec
TD0                     1

======== CHANNEL f1 ========
NUC1                   1H
P1                  13.00 usec
PL1                  2.00 dB
SFO1          500.0335010 MHz
SI                  16384
SF            500.0300070 MHz
WDW                    EM
SSB                     0
LB                   0.60 Hz
GB                      0
PC                   2.00
```

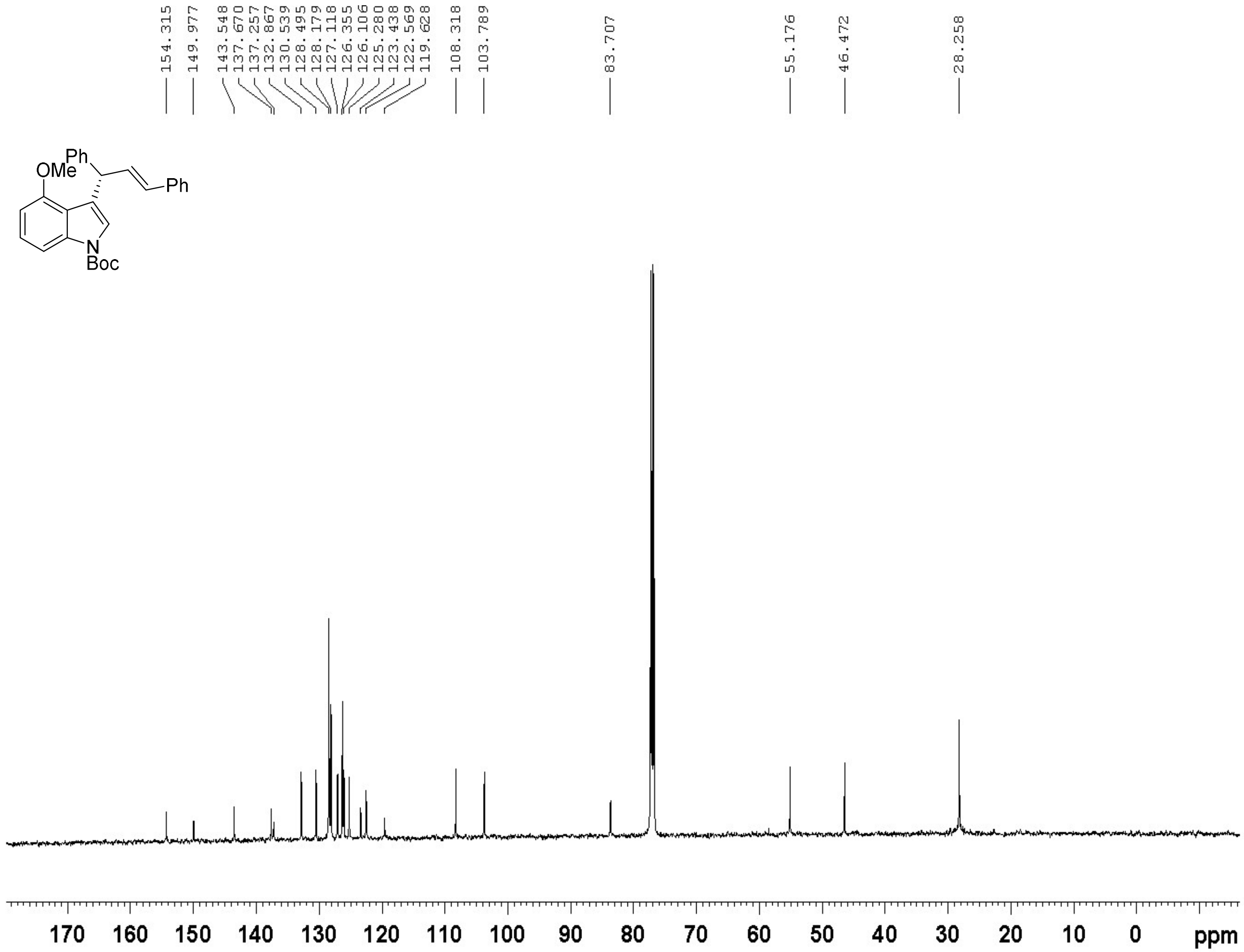

NAME	QZX-5-50-22	
EXPNO	21	
PROCNO	1	
Date_	20181023	
Time	16.45	
INSTRUM	spect	
PROBHD	5 mm PADUL 13C	
PULPROG	zgpg30	
TD	65536	
SOLVENT	CDCl3	
NS	519	
DS	1	
SWH	32679.738	Hz
FIDRES	0.498653	Hz
AQ	1.0027661	sec
RG	2050	
DW	15.300	usec
DE	6.00	usec
TE	297.5	K
D1	2.00000000	sec
d11	0.03000000	sec
DELTA	1.89999998	sec
TD0	20	
======== CHANNEL f1 ========		
NUC1	13C	
P1	12.20	usec
PL1	3.00	dB
SFO1	125.7464750	MHz
======== CHANNEL f2 ========		
CPDPRG2	waltz16	
NUC2	1H	
PCPD2	80.00	usec
PL2	2.00	dB
PL12	17.70	dB
PL13	17.70	dB
SFO2	500.0355000	MHz
SI	32768	
SF	125.7326392	MHz
WDW	EM	
SSB	0	
LB	6.00	Hz
GB	0	
PC	1.00	

(*S,E*)-*tert*-butyl 3-(1,3-diphenylallyl)-5-methyl-1H-indole-1-carboxylate **(Boc-3f)**

QZX-5-29-21 1H 1D 2018 09 17

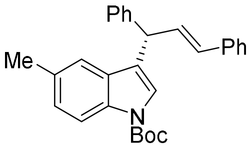

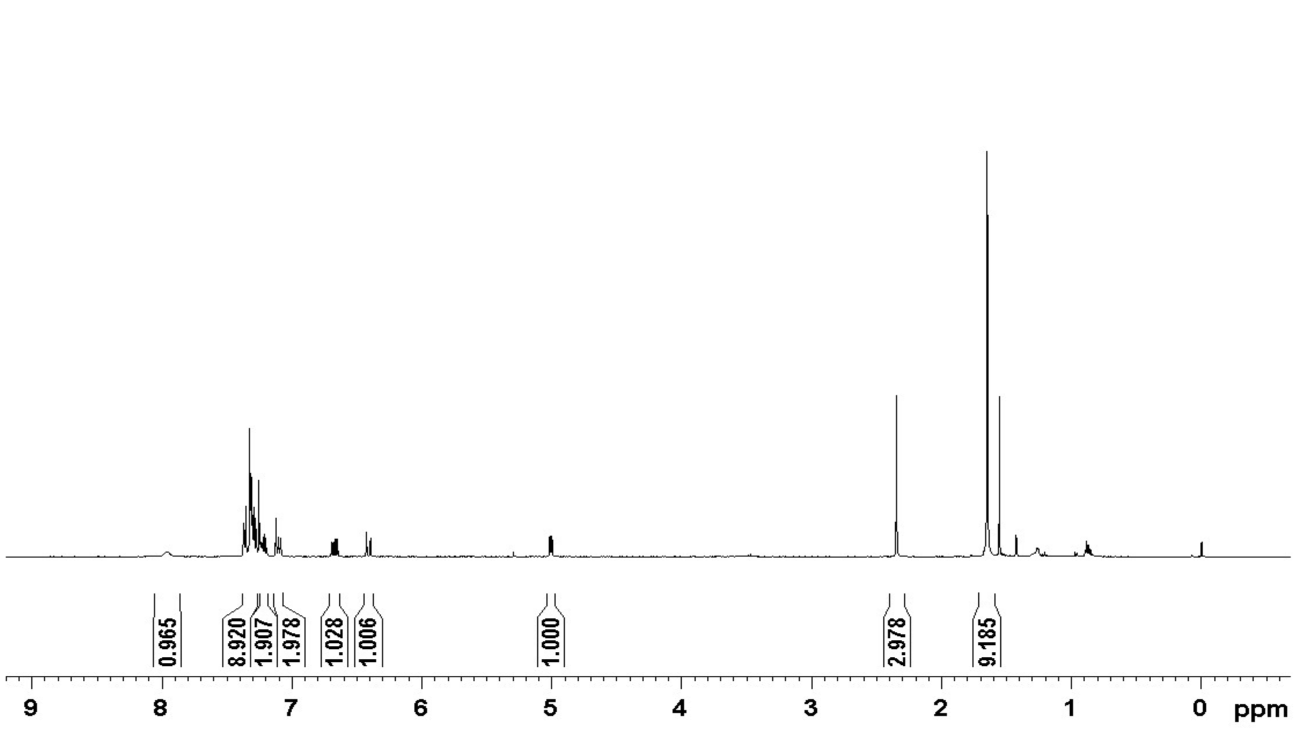

NAME	QZX-5-29-21
EXPNO	1
PROCNO	1
Date_	20180917
Time	14.21
INSTRUM	spect
PROBHD	5 mm PADUL 13C
PULPROG	zg30
TD	16384
SOLVENT	CDCl3
NS	8
DS	0
SWH	10000.000 Hz
FIDRES	0.610352 Hz
AQ	0.8193000 sec
RG	362
DW	50.000 usec
DE	8.00 usec
TE	296.3 K
D1	2.00000000 sec
TD0	1

======== CHANNEL f1 ========	
NUC1	1H
P1	13.00 usec
PL1	2.00 dB
SFO1	500.0335000 MHz
SI	16384
SF	500.0300132 MHz
WDW	EM
SSB	0
LB	0.30 Hz
GB	0
PC	1.00

QZX-5-29-22 13C 2018 09 17

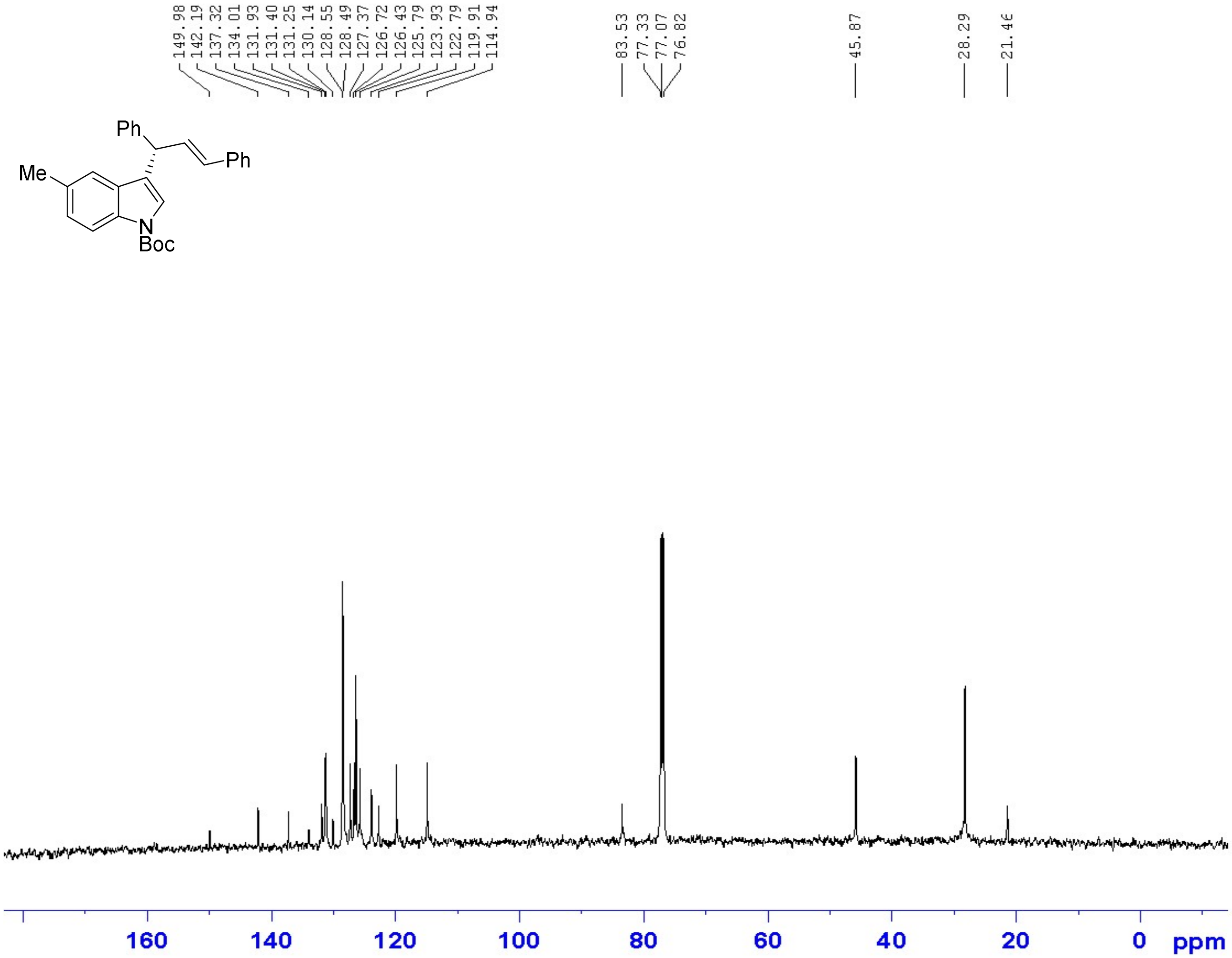

NAME QZX-5-29-22
EXPNO 2
PROCNO 1
Date_ 20180917
Time 17.48
INSTRUM spect
PROBHD 5 mm PADUL 13C
PULPROG zgpg30
TD 65536
SOLVENT CDCl3
NS 114
DS 1
SWH 32679.738 Hz
FIDRES 0.498653 Hz
AQ 1.0027661 sec
RG 3250
DW 15.300 usec
DE 6.00 usec
TE 298.3 K
D1 2.00000000 sec
d11 0.03000000 sec
DELTA 1.89999998 sec
TD0 20

======== CHANNEL f1 ========
NUC1 13C
P1 12.20 usec
PL1 3.00 dB
SFO1 125.7464750 MHz

======== CHANNEL f2 ========
CPDPRG2 waltz16
NUC2 1H
PCPD2 80.00 usec
PL2 2.00 dB
PL12 17.70 dB
PL13 17.70 dB
SFO2 500.0355000 MHz
SI 32768
SF 125.7326392 MHz
WDW EM
SSB 0
LB 10.00 Hz
GB 0
PC 1.00

(*S*,*E*)-*tert*-butyl 3-(1,3-diphenylallyl)-5-methoxy-1H-indole-1-carboxylate **(Boc-3g)**

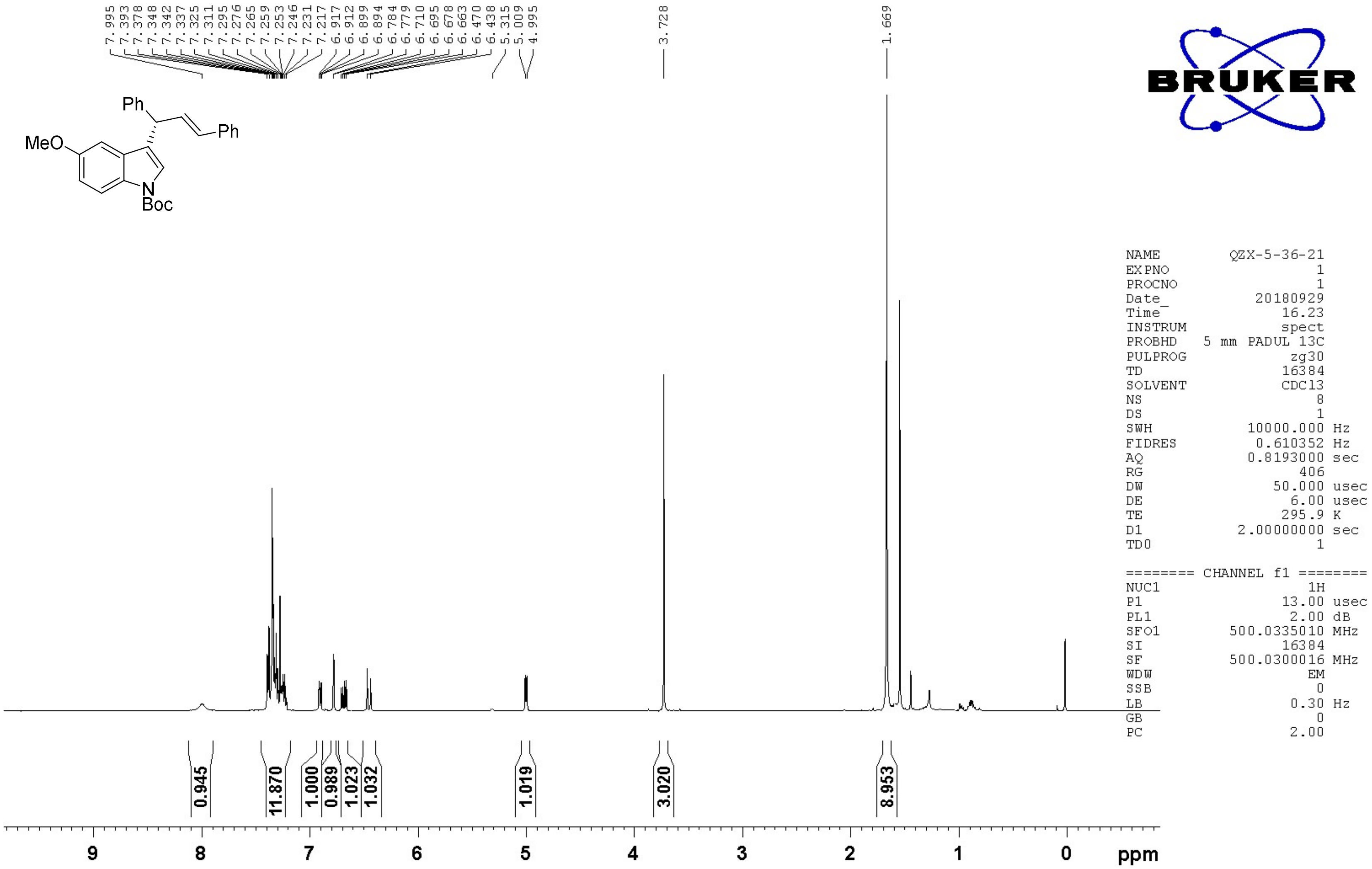

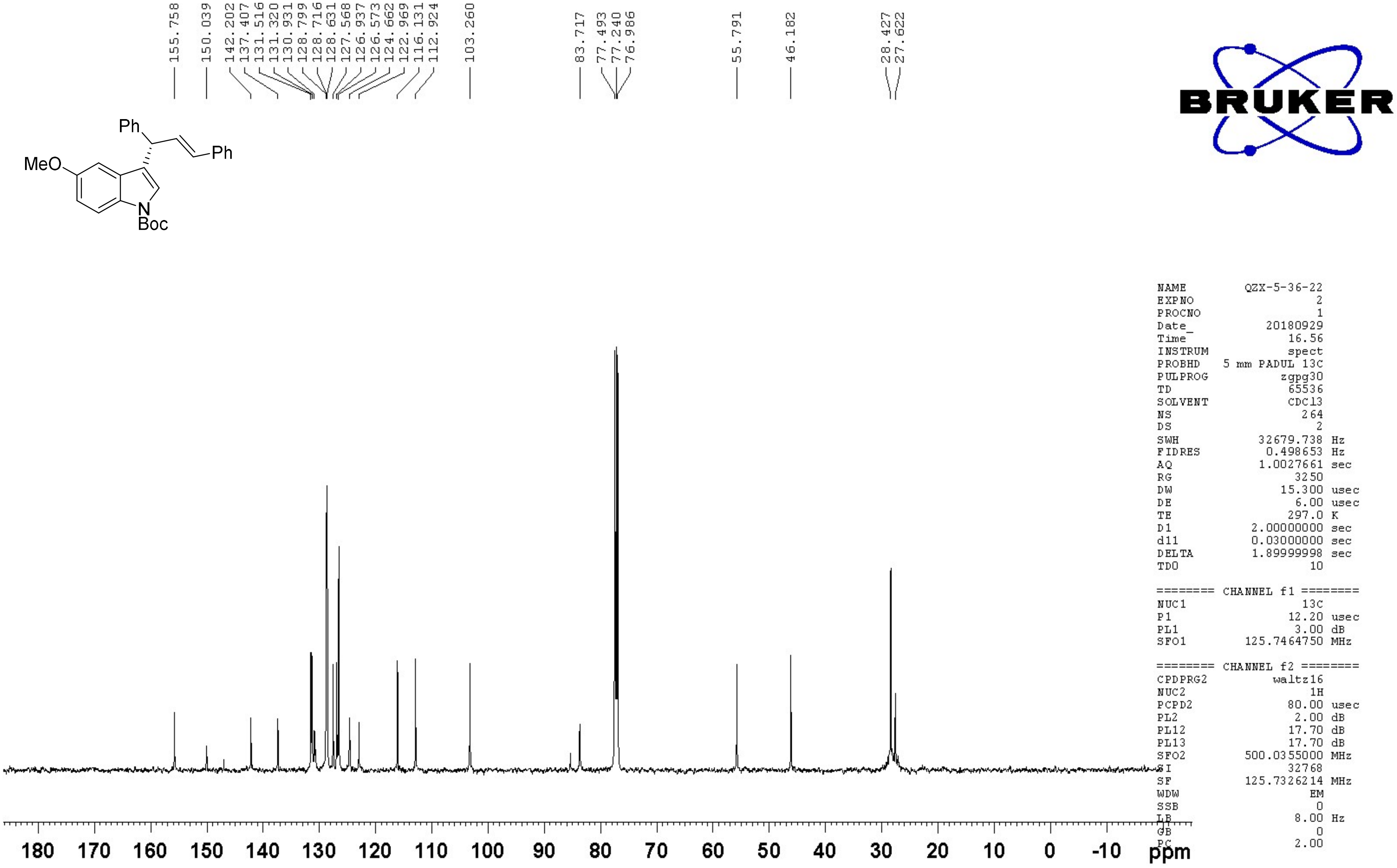
155.758
150.039
142.202
137.407
131.516
131.320
130.931
128.799
128.716
128.631
127.568
126.937
126.573
124.662
122.969
116.131
112.924
103.260
83.717
77.493
77.240
76.986
55.791
46.182
28.427
27.622
Ph
Ph
MeO
N
Boc
NAME QZX-5-36-22
EXPNO 2
PROCNO 1
Date_ 20180929
Time 16.56
INSTRUM spect
PROBHD 5 mm PADUL 13C
PULPROG zgpg30
TD 65536
SOLVENT CDCl3
NS 264
DS 2
SWH 32679.738 Hz
FIDRES 0.498653 Hz
AQ 1.0027661 sec
RG 3250
DW 15.300 usec
DE 6.00 usec
TE 297.0 K
D1 2.00000000 sec
d11 0.03000000 sec
DELTA 1.89999998 sec
TD0 10
======== CHANNEL f1 ========
NUC1 13C
P1 12.20 usec
PL1 3.00 dB
SFO1 125.7464750 MHz
======== CHANNEL f2 ========
CPDPRG2 waltz16
NUC2 1H
PCPD2 80.00 usec
PL2 2.00 dB
PL12 17.70 dB
PL13 17.70 dB
SFO2 500.0355000 MHz
SI 32768
SF 125.7326214 MHz
WDW EM
SSB 0
LB 8.00 Hz
GB 0
PC 2.00
180 170 160 150 140 130 120 110 100 90 80 70 60 50 40 30 20 10 0 -10 ppm

(*S,E*)-*tert*-butyl 5-(benzyloxy)-3-(1,3-diphenylallyl)-1H-indole-1-carboxylate **(Boc-3h)**

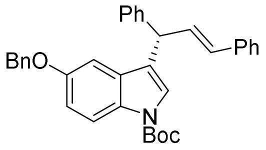

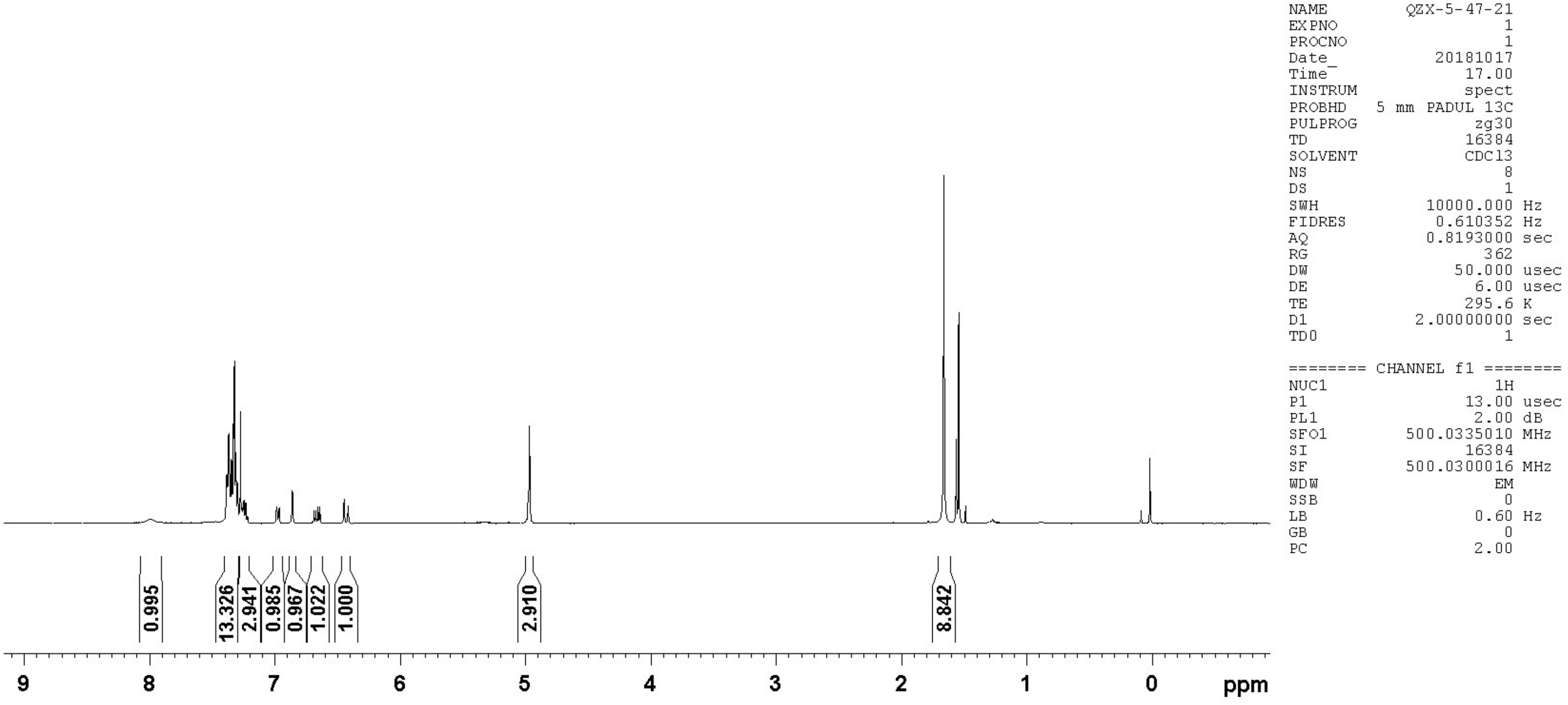

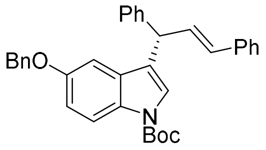

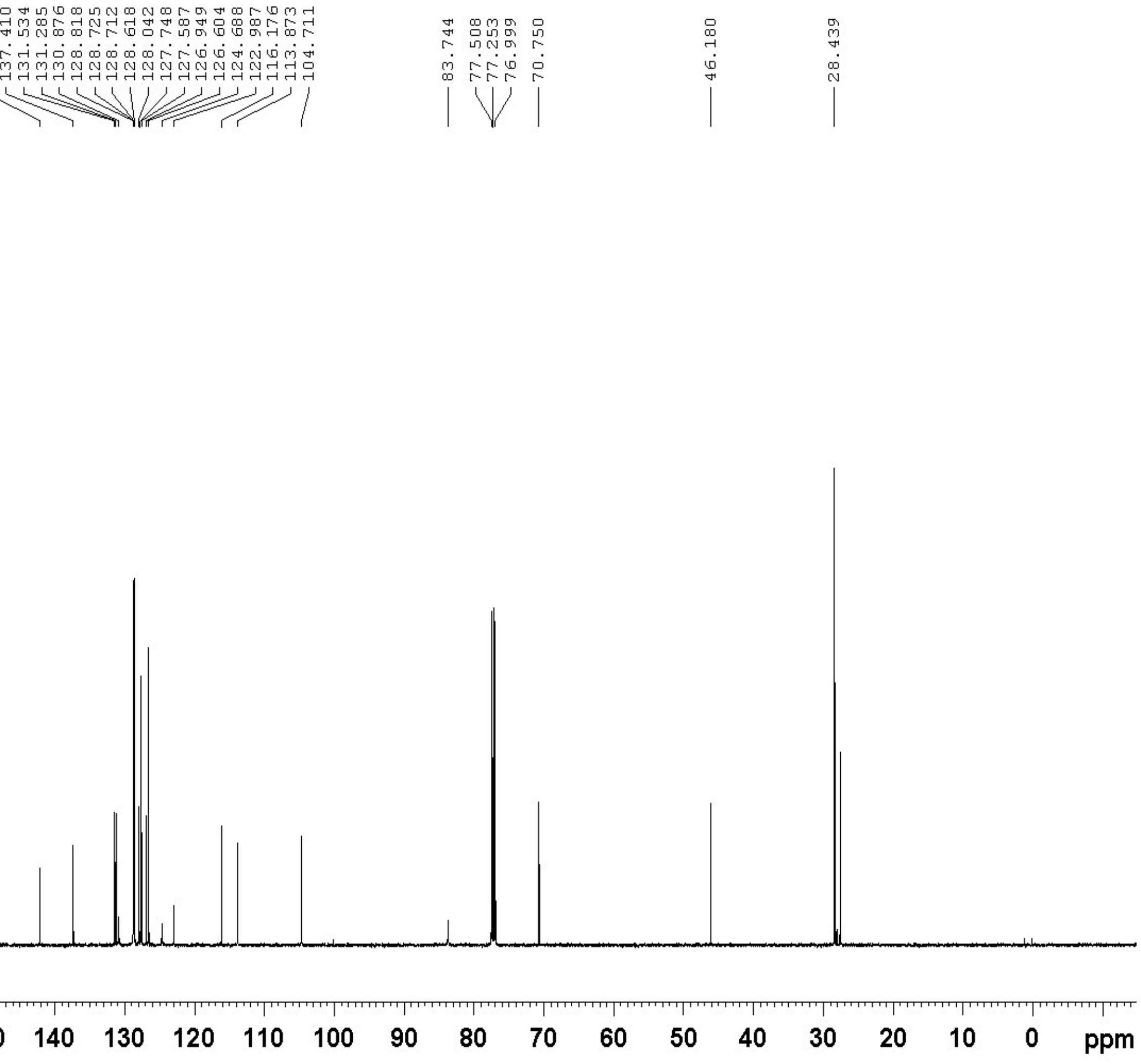

NAME	QZX-5-47-22	
EXPNO	2	
PROCNO	1	
Date_	20181017	
Time	17.49	
INSTRUM	spect	
PROBHD	5 mm PADUL 13C	
PULPROG	zgpg30	
TD	65536	
SOLVENT	CDCl3	
NS	601	
DS	4	
SWH	30030.029	Hz
FIDRES	0.458222	Hz
AQ	1.0912410	sec
RG	144	
DW	16.650	usec
DE	8.00	usec
TE	296.8	K
D1	2.00000000	sec
d11	0.03000000	sec
DELTA	1.89999998	sec
TD0	1	
======== CHANNEL f1 ========		
NUC1	13C	
P1	12.20	usec
PL1	4.00	dB
SFO1	125.7452168	MHz
======== CHANNEL f2 ========		
CPDPRG2	waltz16	
NUC2	1H	
PCPD2	80.00	usec
PL2	2.00	dB
PL12	18.00	dB
PL13	18.00	dB
SFO2	500.0320001	MHz
SI	32768	
SF	125.7326209	MHz
WDW	EM	
SSB	0	
LB	1.00	Hz
GB	0	
PC	1.40	

(*S*,*E*)-*tert*-butyl 5-chloro-3-(1,3-diphenylallyl)-1H-indole-1-carboxylate **(Boc-3i)**

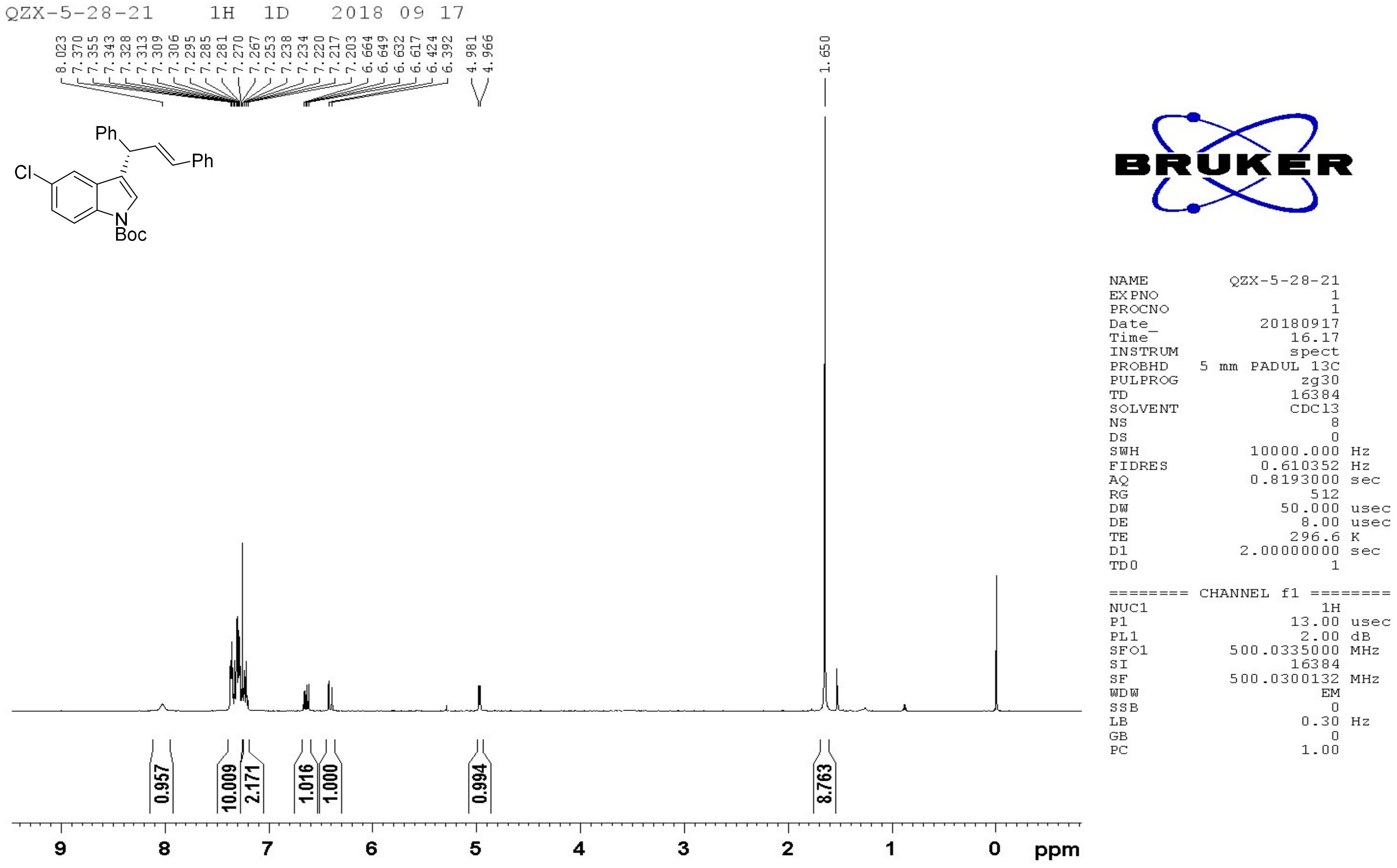

QZX-5-28-22   13C   2018 09 17

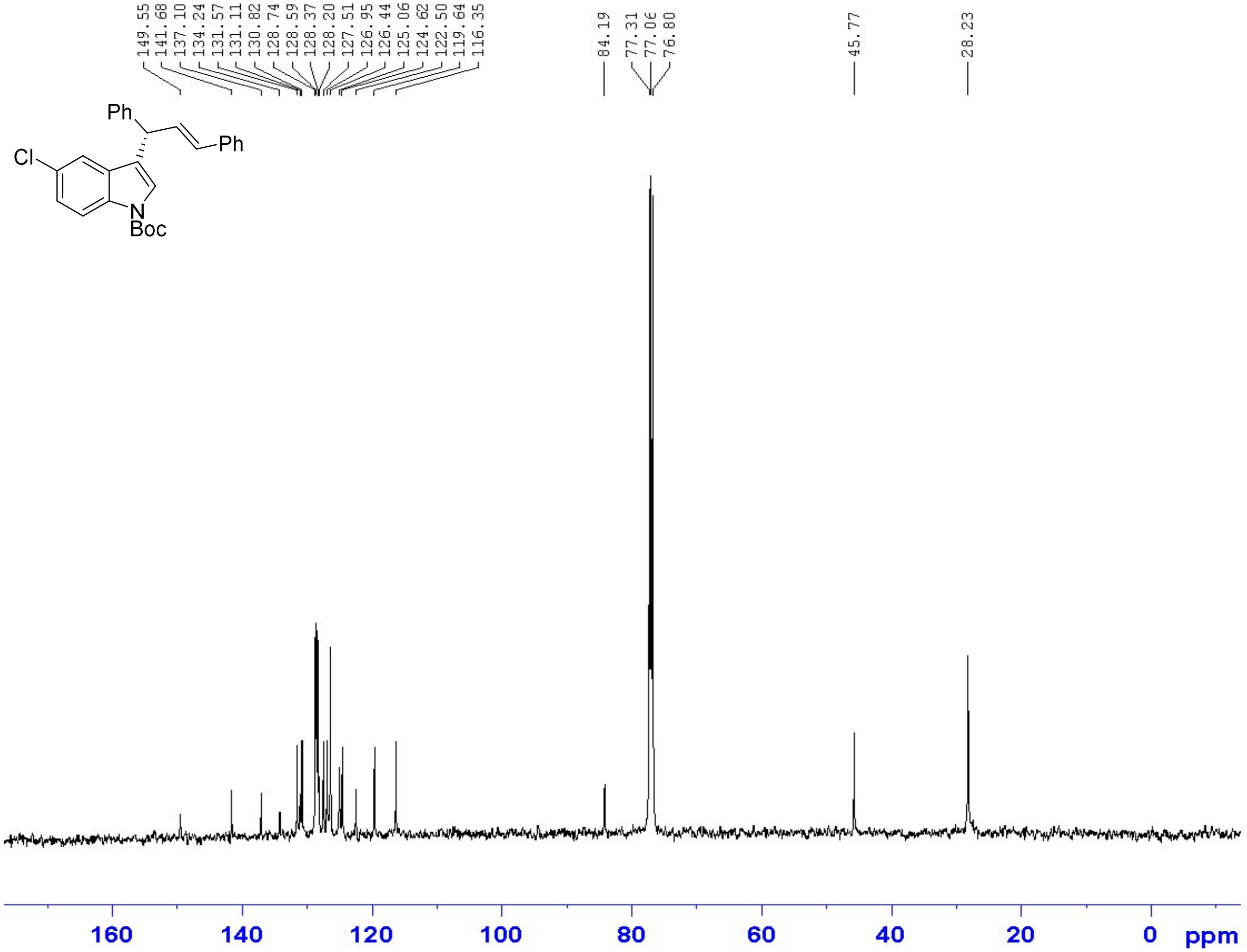

NAME QZX-5-28-22
EXPNO 2
PROCNO 1
Date_ 20180917
Time 17.34
INSTRUM spect
PROBHD 5 mm PADUL 13C
PULPROG zgpg30
TD 65536
SOLVENT CDCl3
NS 269
DS 1
SWH 32679.738 Hz
FIDRES 0.498653 Hz
AQ 1.0027661 sec
RG 3250
DW 15.300 usec
DE 6.00 usec
TE 298.4 K
D1 2.00000000 sec
d11 0.03000000 sec
DELTA 1.89999998 sec
TD0 20

======== CHANNEL f1 ========
NUC1 13C
P1 12.20 usec
PL1 3.00 dB
SFO1 125.7464750 MHz

======== CHANNEL f2 ========
CPDPRG2 waltz16
NUC2 1H
PCPD2 80.00 usec
PL2 2.00 dB
PL12 17.70 dB
PL13 17.70 dB
SFO2 500.0355000 MHz
SI 32768
SF 125.7326392 MHz
WDW EM
SSB 0
LB 10.00 Hz
GB 0
PC 1.00

(*S*,*E*)-*tert*-butyl 5-bromo-3-(1,3-diphenylallyl)-1H-indole-1-carboxylate **(Boc-3j)**

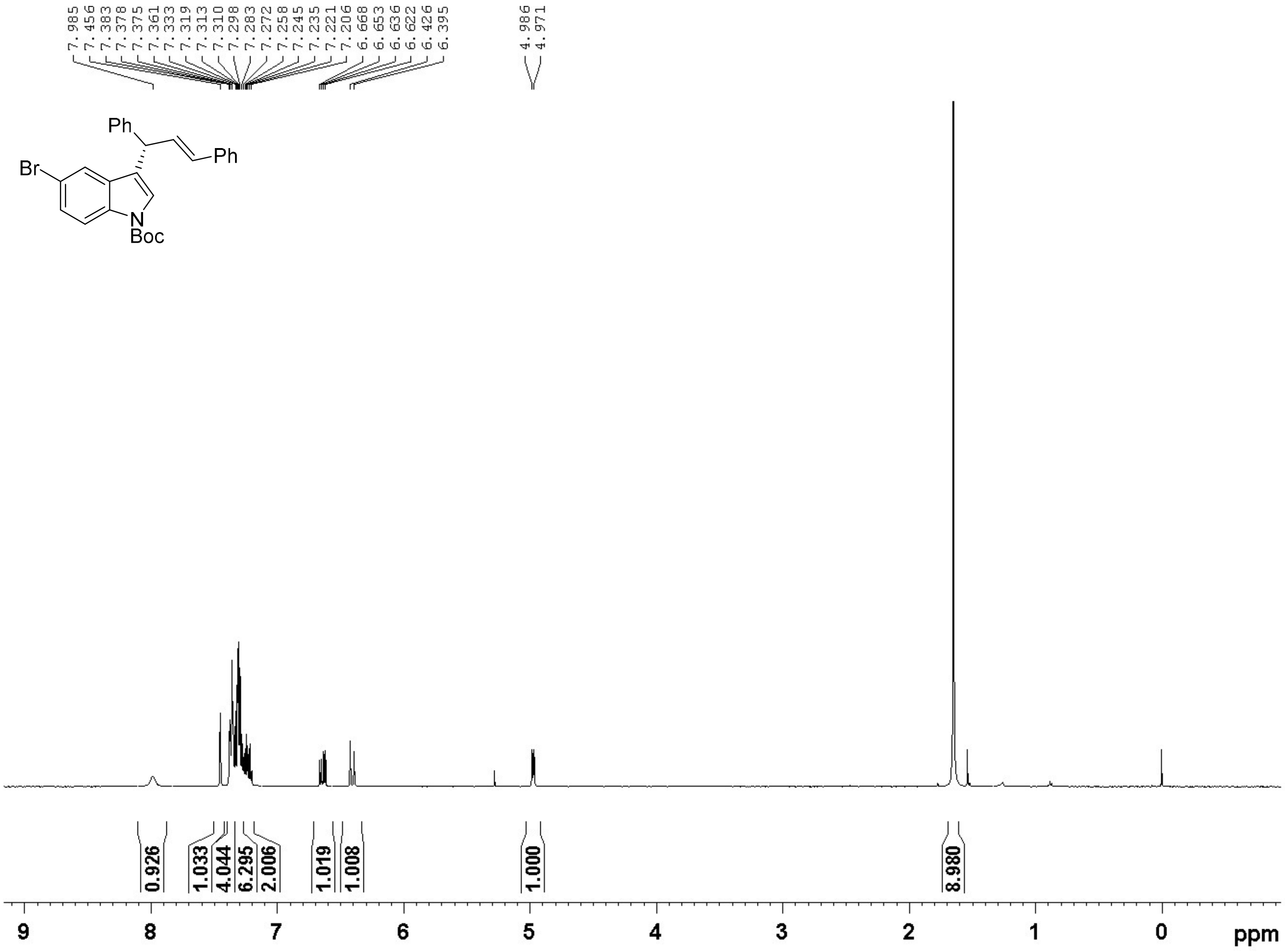

NAME	QZX-5-44-21
EXPNO	1
PROCNO	1
Date_	20181015
Time	16.22
INSTRUM	spect
PROBHD	5 mm PADUL 13C
PULPROG	zg30
TD	16384
SOLVENT	CDCl3
NS	8
DS	0
SWH	10000.000 Hz
FIDRES	0.610352 Hz
AQ	0.8193000 sec
RG	144
DW	50.000 usec
DE	6.00 usec
TE	295.7 K
D1	2.00000000 sec
TD0	1
======== CHANNEL f1 ========	
NUC1	1H
P1	13.00 usec
PL1	2.00 dB
SFO1	500.0335010 MHz
SI	16384
SF	500.0300171 MHz
WDW	EM
SSB	0
LB	0.30 Hz
GB	0
PC	1.00

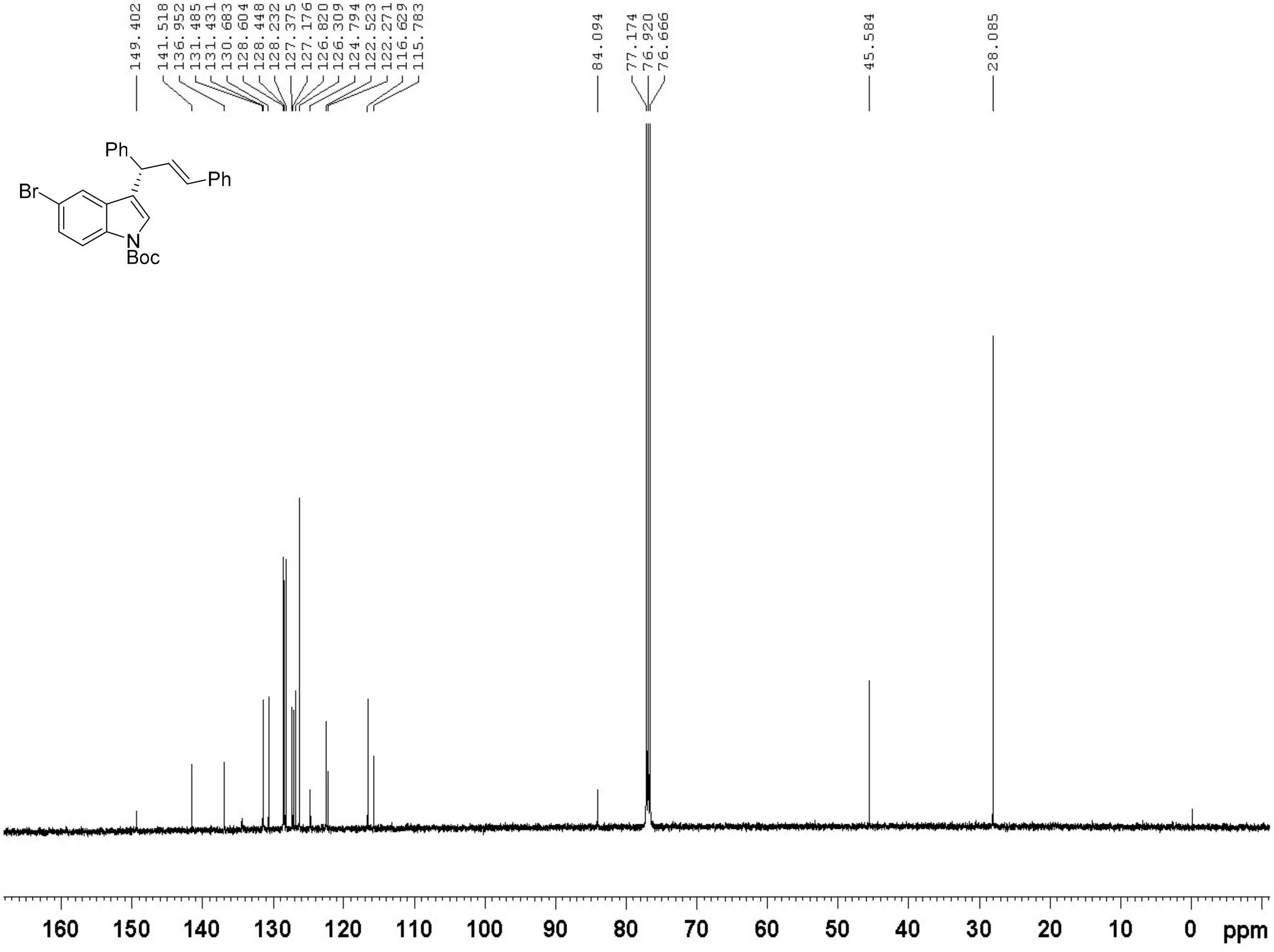


```
NAME          QZX-5-44-22
EXPNO                   2
PROCNO                  1
Date_            20181015
Time                23.30
INSTRUM             spect
PROBHD   5 mm PADUL 13C
PULPROG           zgpg30
TD                  65536
SOLVENT             CDCl3
NS                   1024
DS                      4
SWH             30030.029 Hz
FIDRES           0.458222 Hz
AQ              1.0912410 sec
RG                    144
DW                 16.650 usec
DE                   8.00 usec
TE                  298.0 K
D1             2.00000000 sec
d11            0.03000000 sec
DELTA          1.89999998 sec
TD0                     1

======== CHANNEL f1 ========
NUC1                  13C
P1                  12.20 usec
PL1                  4.00 dB
SFO1          125.7452168 MHz

======== CHANNEL f2 ========
CPDPRG2           waltz16
NUC2                   1H
PCPD2               80.00 usec
PL2                  2.00 dB
PL12                18.00 dB
PL13                18.00 dB
SFO2          500.0320001 MHz
SI                  32768
SF            125.7326563 MHz
WDW                    EM
SSB                     0
LB                   1.00 Hz
GB                      0
PC                   0.10
```

(*S,E*)-*tert*-butyl 3-(1,3-diphenylallyl)-6-methyl-1H-indole-1-carboxylate **(Boc-3k)**

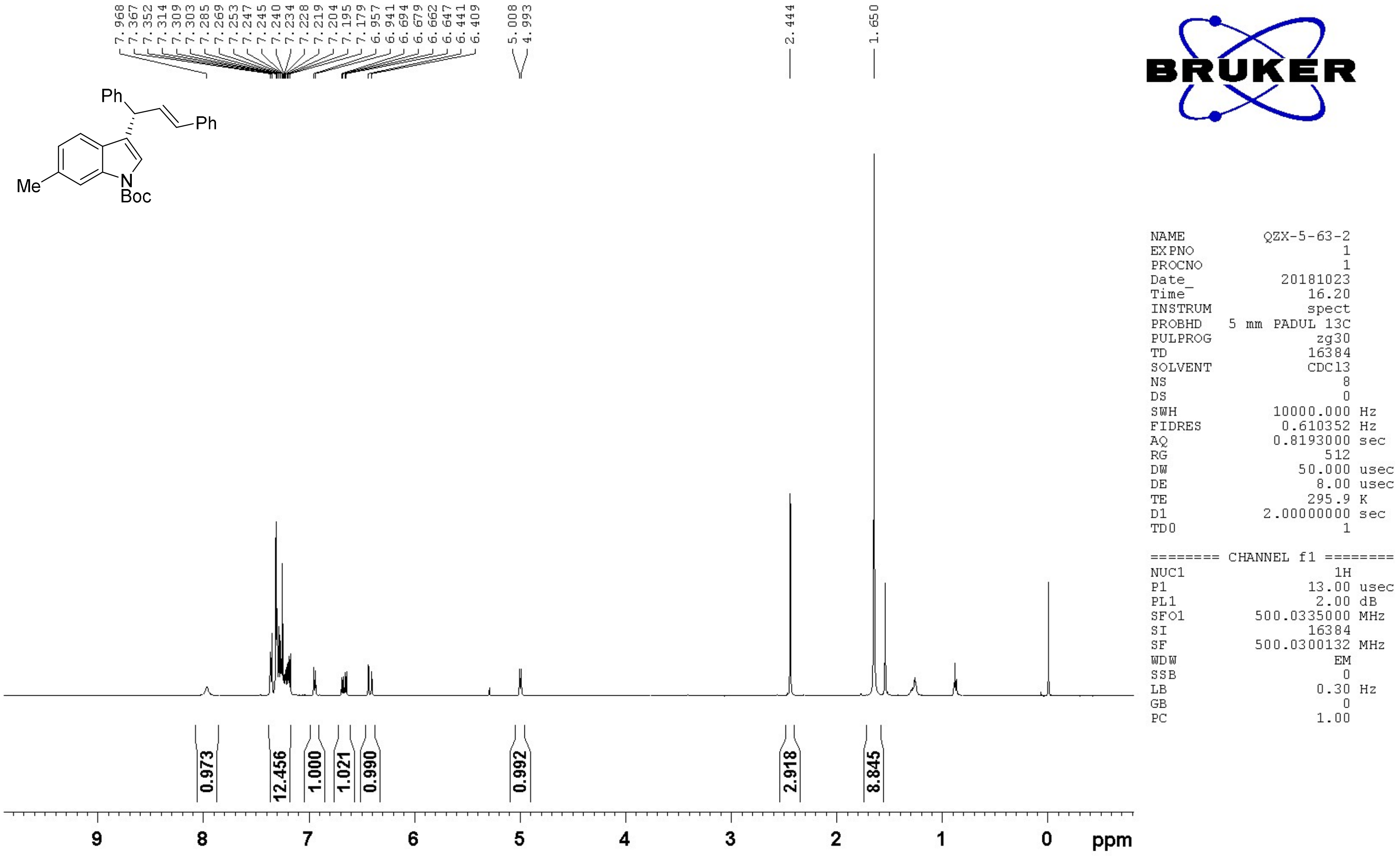

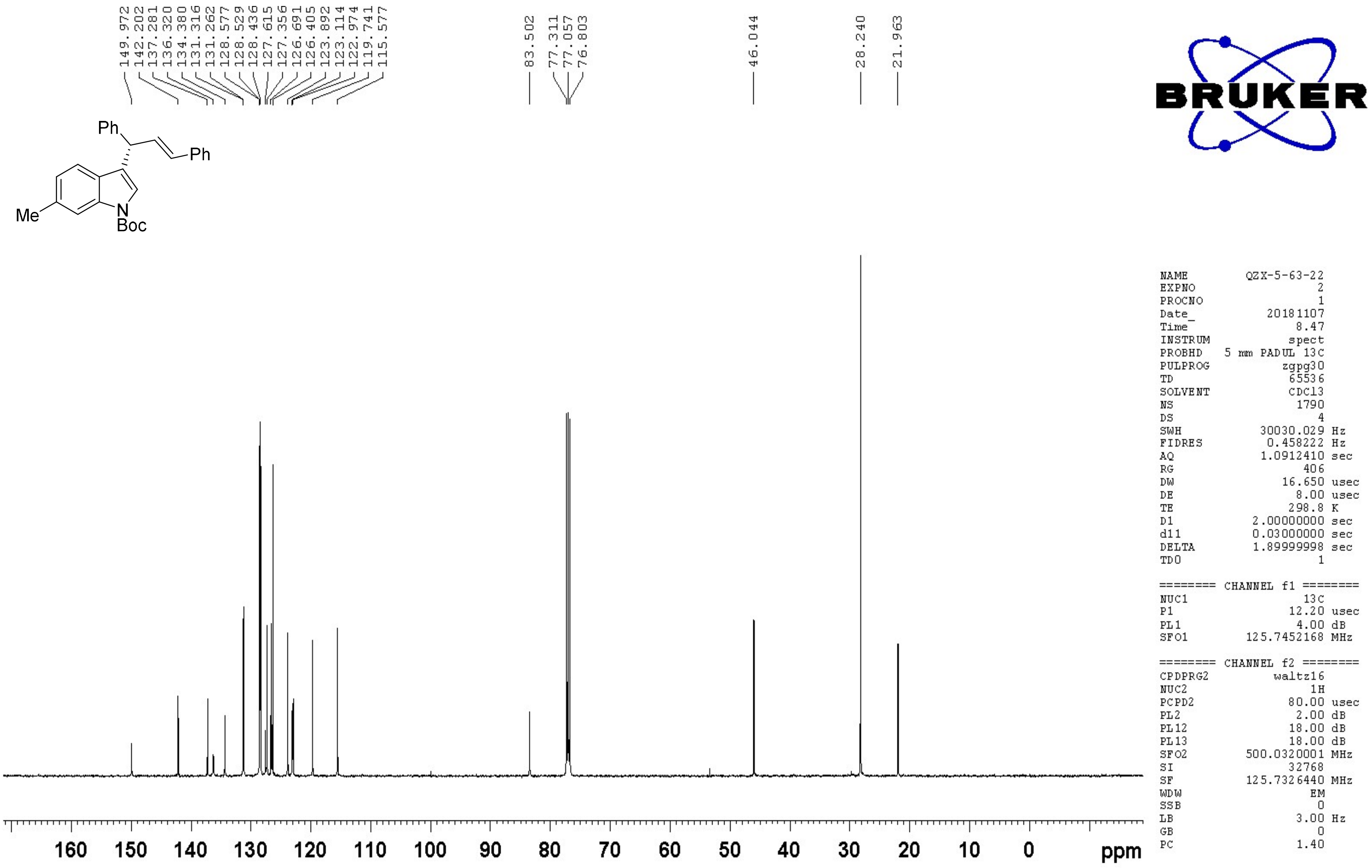
149.972
142.202
137.281
136.320
134.380
131.316
131.262
128.577
128.529
128.436
127.615
127.356
126.691
126.405
123.892
123.114
122.974
119.741
115.577
83.502
77.311
77.057
76.803
46.044
28.240
21.963
Ph
Ph
Me
N
Boc
NAME QZX-5-63-22
EXPNO 2
PROCNO 1
Date_ 20181107
Time 8.47
INSTRUM spect
PROBHD 5 mm PADUL 13C
PULPROG zgpg30
TD 65536
SOLVENT CDCl3
NS 1790
DS 4
SWH 30030.029 Hz
FIDRES 0.458222 Hz
AQ 1.0912410 sec
RG 406
DW 16.650 usec
DE 8.00 usec
TE 298.8 K
D1 2.00000000 sec
d11 0.03000000 sec
DELTA 1.89999998 sec
TD0 1
======== CHANNEL f1 ========
NUC1 13C
P1 12.20 usec
PL1 4.00 dB
SFO1 125.7452168 MHz
======== CHANNEL f2 ========
CPDPRG2 waltz16
NUC2 1H
PCPD2 80.00 usec
PL2 2.00 dB
PL12 18.00 dB
PL13 18.00 dB
SFO2 500.0320001 MHz
SI 32768
SF 125.7326440 MHz
WDW EM
SSB 0
LB 3.00 Hz
GB 0
PC 1.40
160 150 140 130 120 110 100 90 80 70 60 50 40 30 20 10 0 ppm

(*S*,*E*)-*tert*-butyl 6-chloro-3-(1,3-diphenylallyl)-1H-indole-1-carboxylate **(Boc-3l)**

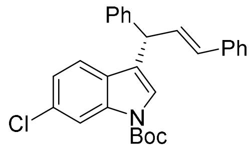

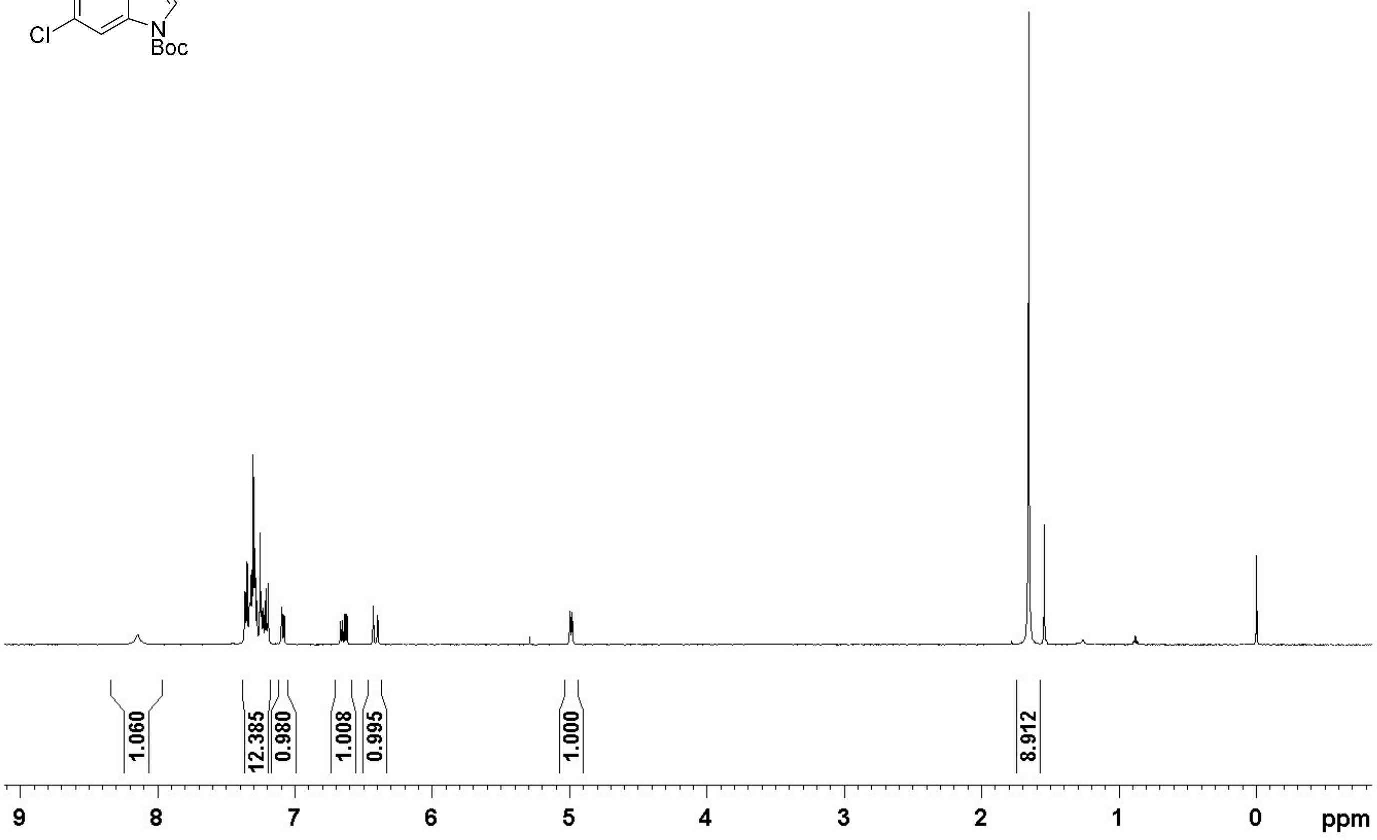


```
NAME            QZX-5-65-21
EXPNO                     1
PROCNO                    1
Date_              20181023
Time                  16.22
INSTRUM               spect
PROBHD     5 mm PADUL 13C
PULPROG                zg30
TD                    16384
SOLVENT               CDCl3
NS                        8
DS                        0
SWH              10000.000 Hz
FIDRES            0.610352 Hz
AQ               0.8193000 sec
RG                      512
DW                   50.000 usec
DE                     8.00 usec
TE                    295.9 K
D1               2.00000000 sec
TD0                       1

======== CHANNEL f1 ========
NUC1                     1H
P1                    13.00 usec
PL1                    2.00 dB
SFO1            500.0335000 MHz
SI                    16384
SF              500.0300132 MHz
WDW                      EM
SSB                       0
LB                     0.30 Hz
GB                        0
PC                     1.00
```

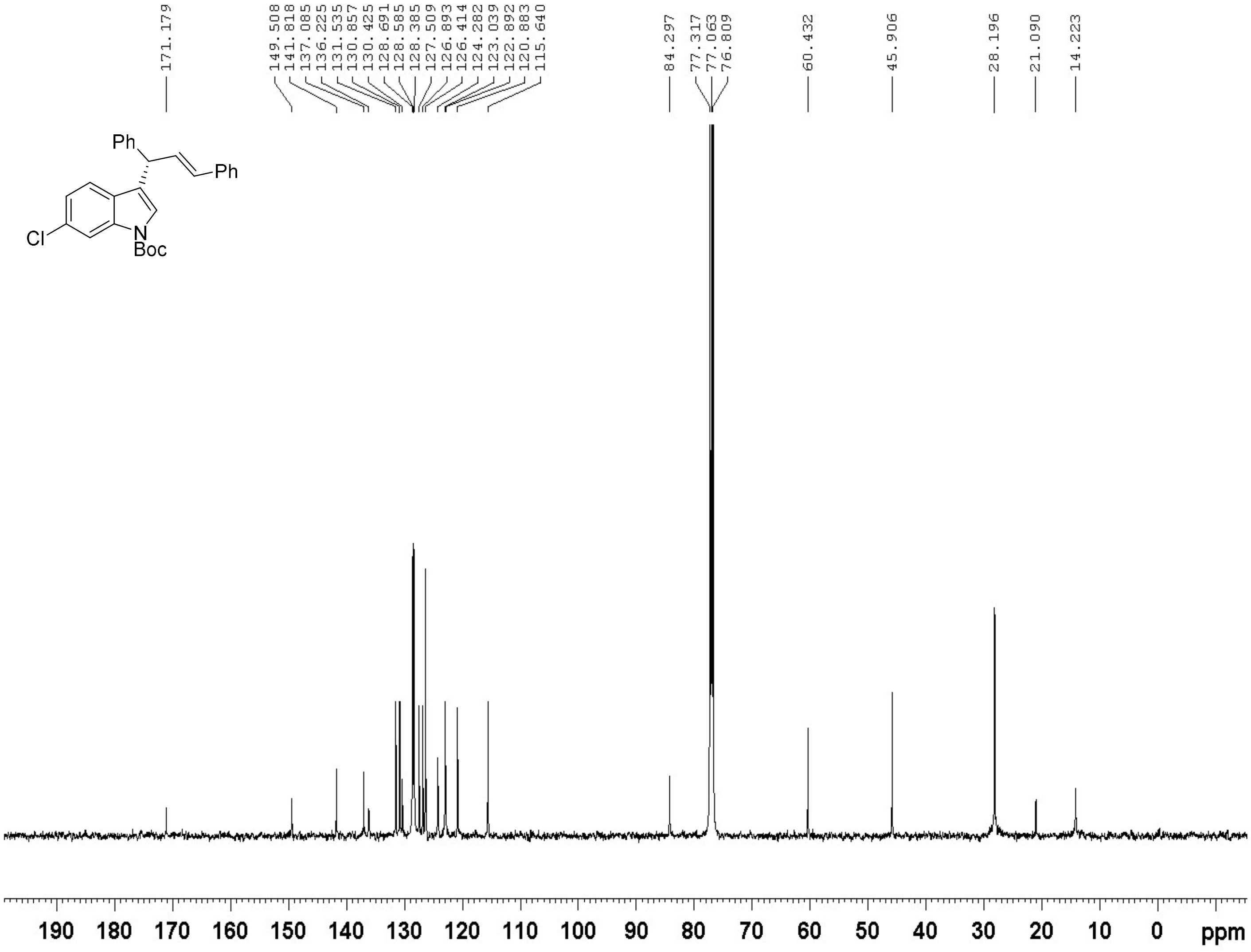

NAME	QZX-5-65-22	
EXPNO	2	
PROCNO	1	
Date_	20181026	
Time	17.48	
INSTRUM	spect	
PROBHD	5 mm PADUL 13C	
PULPROG	zgpg30	
TD	65536	
SOLVENT	CDCl3	
NS	909	
DS	1	
SWH	32679.738	Hz
FIDRES	0.498653	Hz
AQ	1.0027661	sec
RG	13000	
DW	15.300	usec
DE	6.00	usec
TE	297.0	K
D1	2.00000000	sec
d11	0.03000000	sec
DELTA	1.89999998	sec
TD0	10	
======== CHANNEL f1 ========		
NUC1	13C	
P1	12.20	usec
PL1	3.00	dB
SFO1	125.7464750	MHz
======== CHANNEL f2 ========		
CPDPRG2	waltz16	
NUC2	1H	
PCPD2	80.00	usec
PL2	2.00	dB
PL12	17.70	dB
PL13	17.70	dB
SFO2	500.0355000	MHz
SI	32768	
SF	125.7326392	MHz
WDW	EM	
SSB	0	
LB	6.00	Hz
GB	0	
PC	1.00	

(*S,E*)-*tert*-butyl 3-(1,3-diphenylallyl)-7-methyl-1H-indole-1-carboxylate **(Boc-3m)**

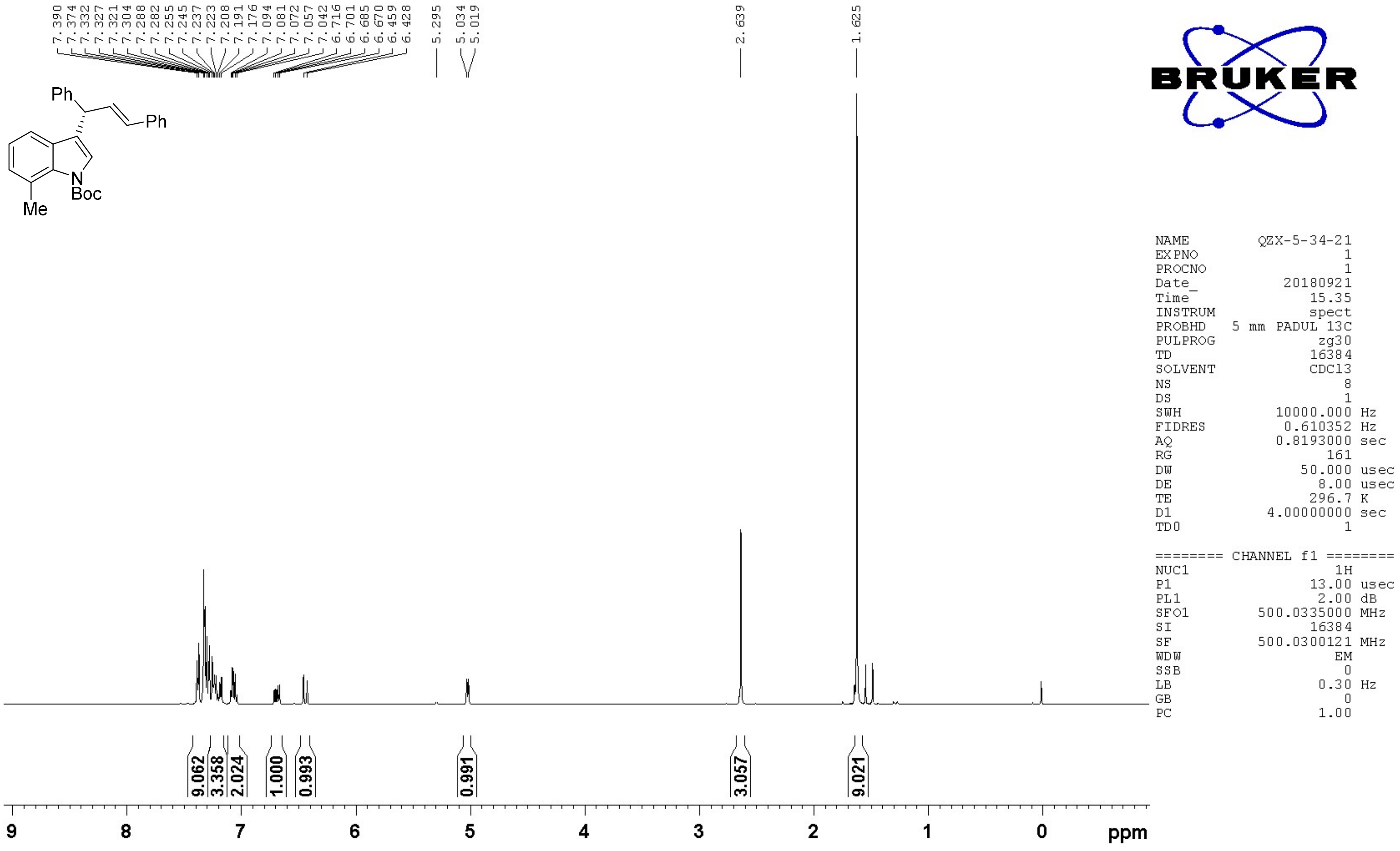

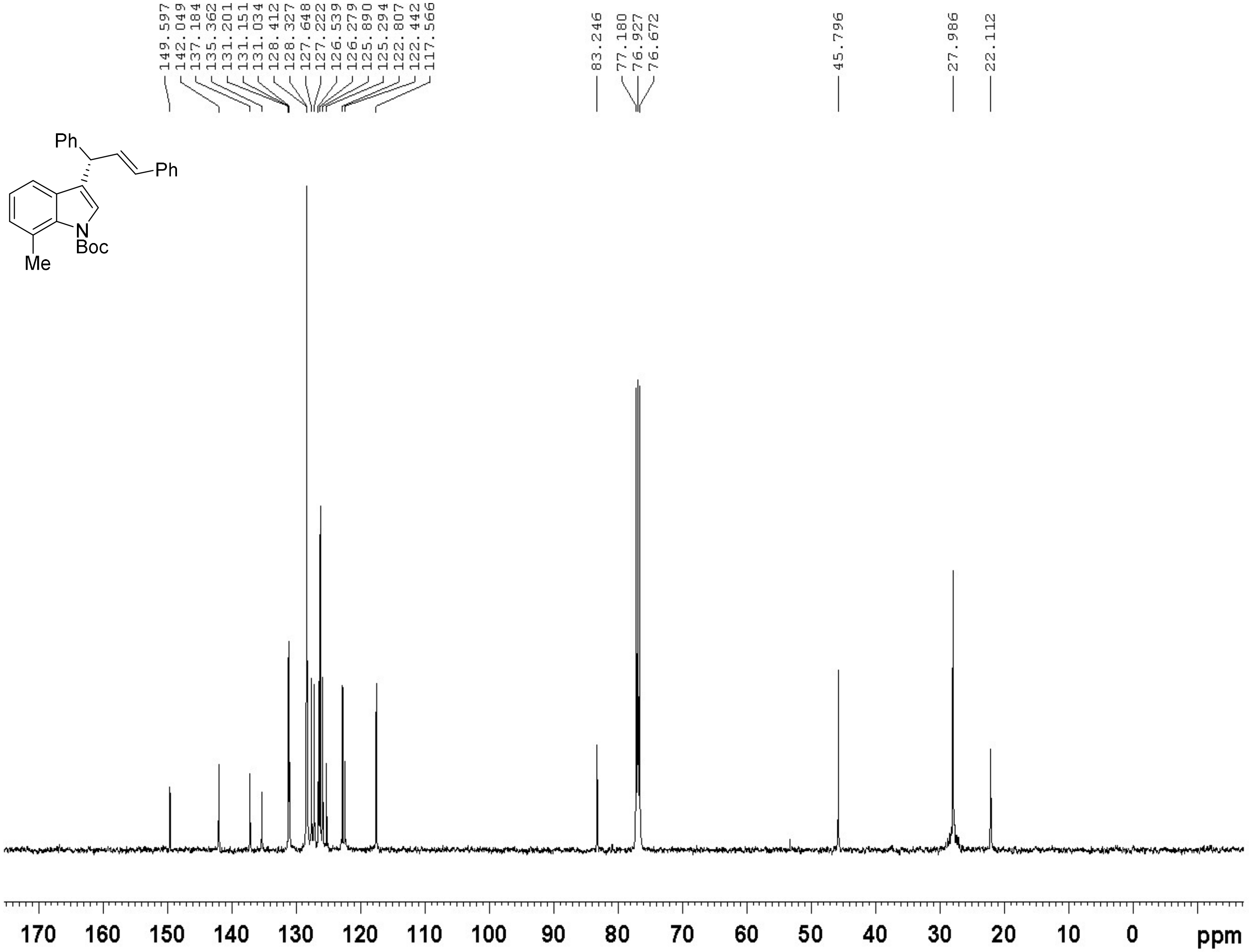


```
NAME          QZX-5-34-22
EXPNO                   2
PROCNO                  1
Date_            20180921
Time                17.39
INSTRUM             spect
PROBHD   5 mm PADUL 13C
PULPROG           zgpg30
TD                 65536
SOLVENT             CDCl3
NS                    282
DS                      1
SWH            32679.738 Hz
FIDRES          0.498653 Hz
AQ             1.0027661 sec
RG                   8200
DW                 15.300 usec
DE                   6.00 usec
TE                  298.7 K
D1             2.00000000 sec
d11            0.03000000 sec
DELTA          1.89999998 sec
TD0                    20

======== CHANNEL f1 ========
NUC1                  13C
P1                  12.20 usec
PL1                  3.00 dB
SFO1          125.7464750 MHz

======== CHANNEL f2 ========
CPDPRG2           waltz16
NUC2                   1H
PCPD2               80.00 usec
PL2                  2.00 dB
PL12                17.70 dB
PL13                17.70 dB
SFO2          500.0355000 MHz
SI                  32768
SF            125.7326612 MHz
WDW                    EM
SSB                     0
LB                   6.00 Hz
GB                      0
PC                   1.00
```

(*S,E*)-*tert*-butyl 3-(1,3-diphenylallyl)-7-methoxy-1H-indole-1-carboxylate **(Boc-3n)**

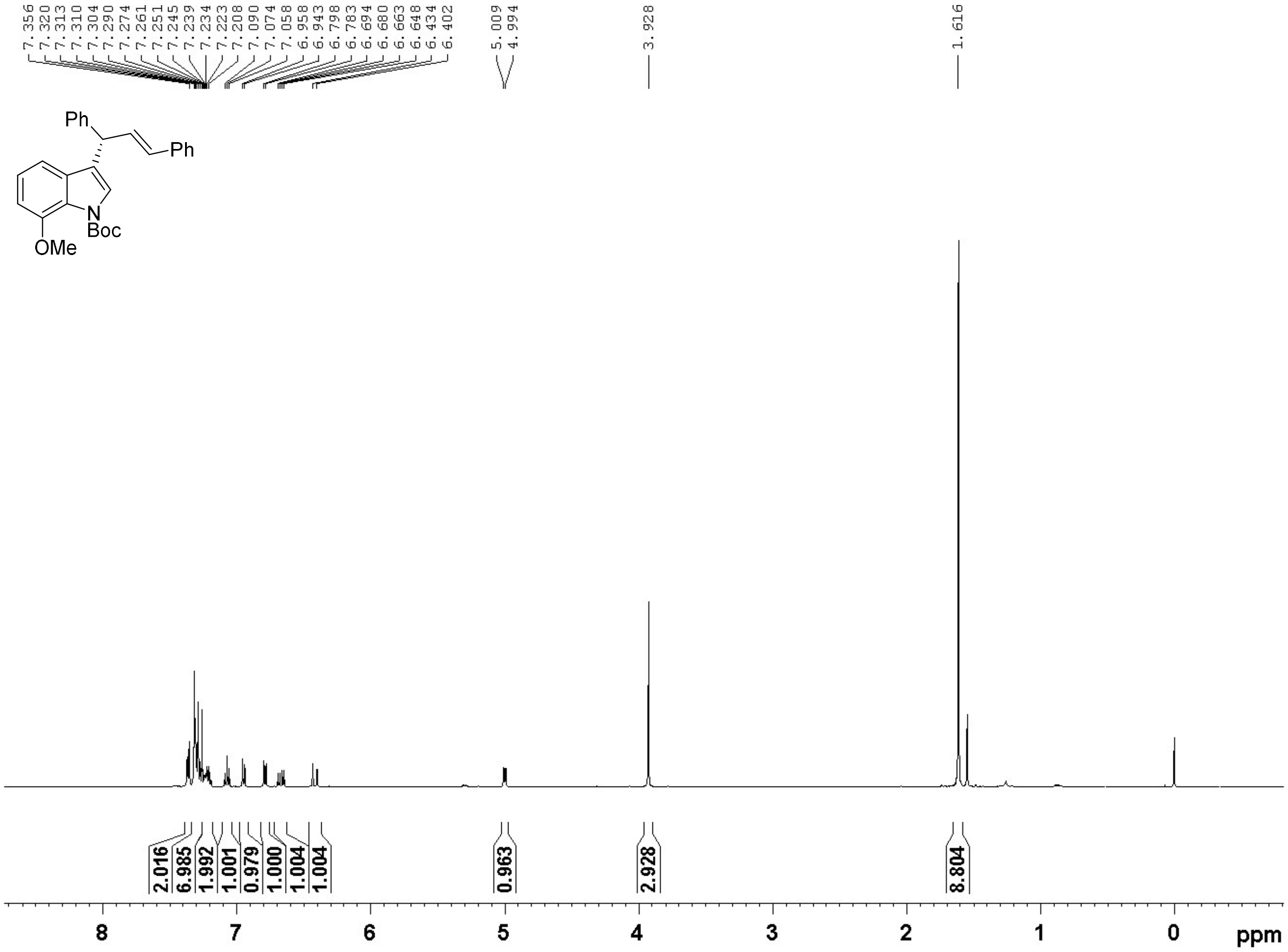

NAME	QZX-5-48-21
EXPNO	1
PROCNO	1
Date_	20181029
Time	16.32
INSTRUM	spect
PROBHD	5 mm PADUL 13C
PULPROG	zg30
TD	16384
SOLVENT	CDCl3
NS	8
DS	1
SWH	10000.000 Hz
FIDRES	0.610352 Hz
AQ	0.8193000 sec
RG	456
DW	50.000 usec
DE	8.00 usec
TE	298.1 K
D1	2.00000000 sec
TD0	1

======== CHANNEL f1 ========

NUC1	1H
P1	13.00 usec
PL1	2.00 dB
SFO1	500.0335000 MHz
SI	16384
SF	500.0300092 MHz
WDW	EM
SSB	0
LB	0.30 Hz
GB	0
PC	1.00

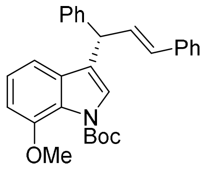

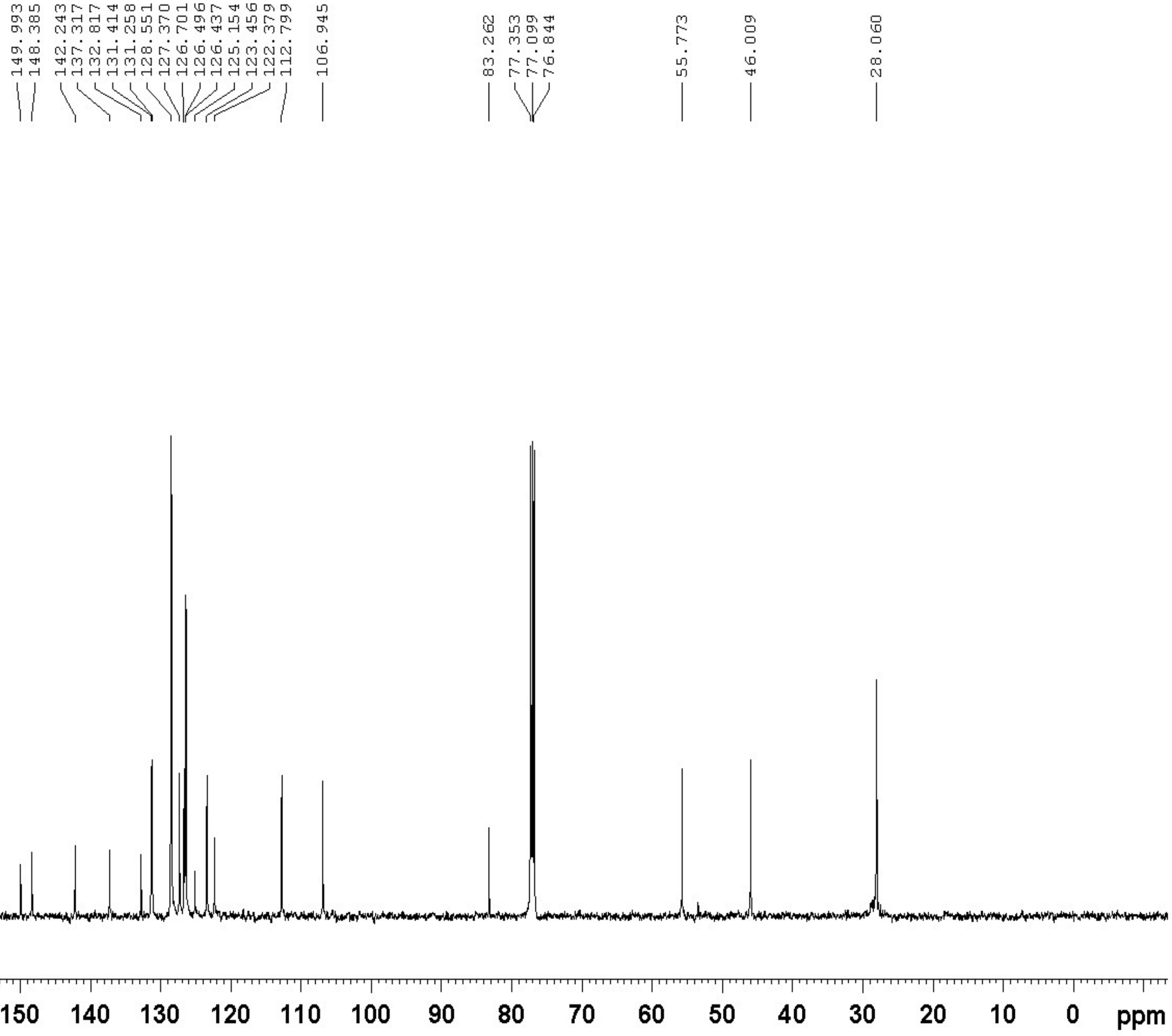

NAME	QZX-5-48-22
EXPNO	2
PROCNO	1
Date_	20181029
Time	15.50
INSTRUM	spect
PROBHD	5 mm PADUL 13C
PULPROG	zgpg30
TD	65536
SOLVENT	CDCl3
NS	162
DS	1
SWH	32679.738 Hz
FIDRES	0.498653 Hz
AQ	1.0027661 sec
RG	14600
DW	15.300 usec
DE	6.00 usec
TE	298.9 K
D1	2.00000000 sec
d11	0.03000000 sec
DELTA	1.89999998 sec
TD0	20

======== CHANNEL f1 ========

NUC1	13C
P1	12.20 usec
PL1	3.00 dB
SFO1	125.7464750 MHz

======== CHANNEL f2 ========

CPDPRG2	waltz16
NUC2	1H
PCPD2	80.00 usec
PL2	2.00 dB
PL12	17.70 dB
PL13	17.70 dB
SFO2	500.0355000 MHz
SI	32768
SF	125.7326392 MHz
WDW	EM
SSB	0
LB	6.00 Hz
GB	0
PC	1.00

(*S*,*E*)-*tert*-butyl 7-chloro-3-(1,3-diphenylallyl)-1H-indole-1-carboxylate **(Boc-3o)**

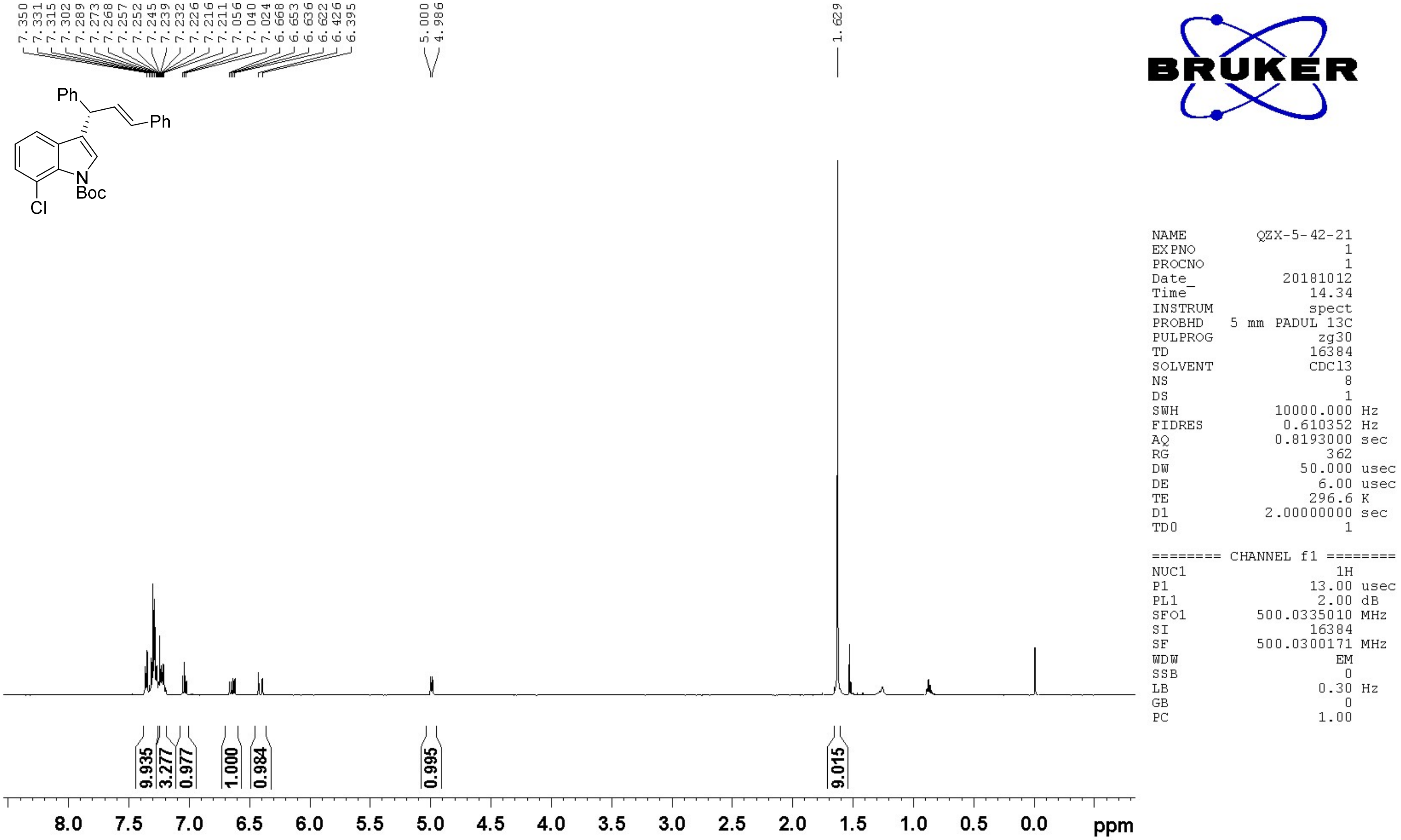

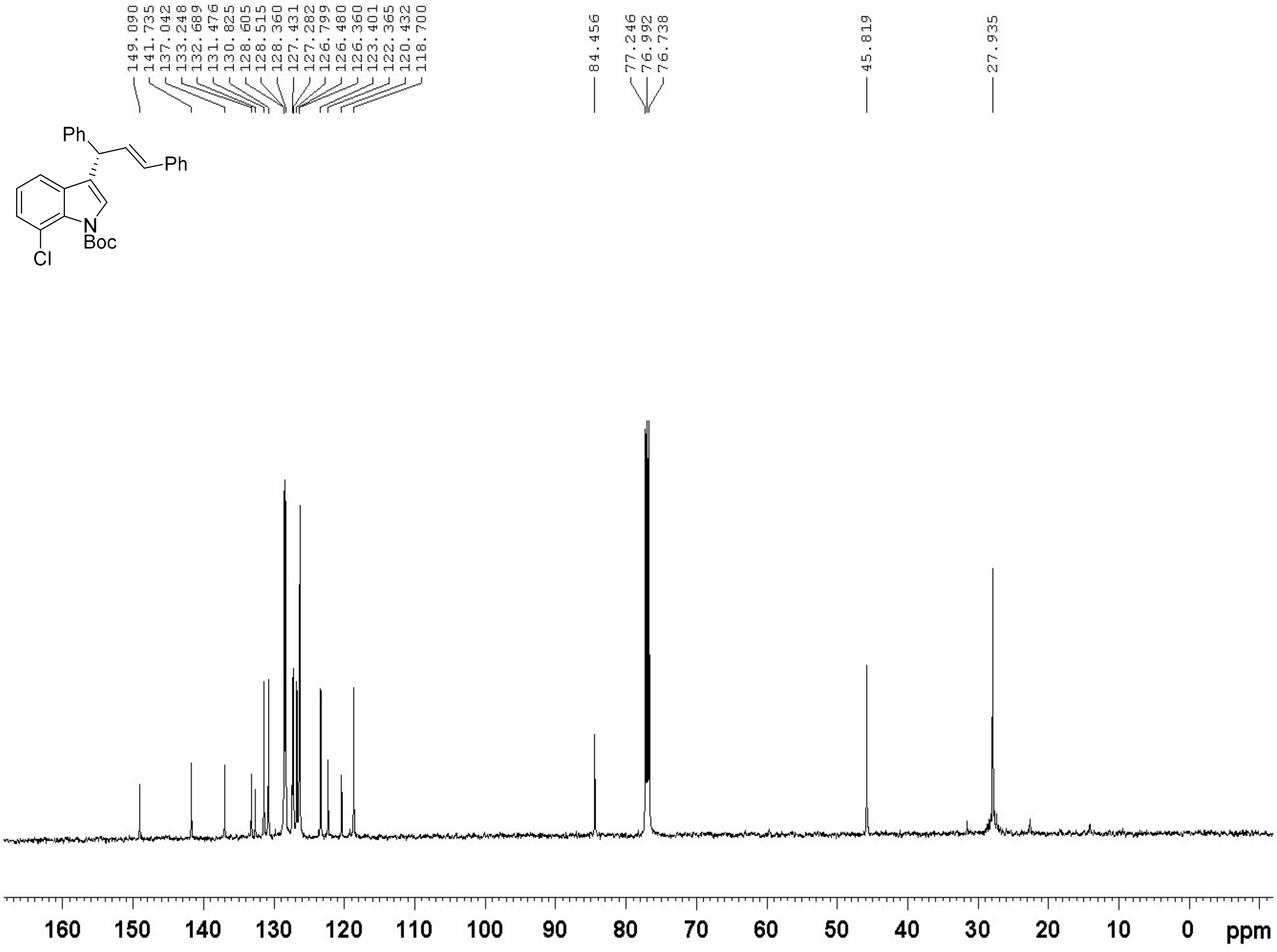

NAME	QZX-5-42-22
EXPNO	2
PROCNO	1
Date_	20181012
Time	18.19
INSTRUM	spect
PROBHD	5 mm PADUL 13C
PULPROG	zgpg30
TD	65536
SOLVENT	CDCl3
NS	534
DS	2
SWH	32679.738 Hz
FIDRES	0.498653 Hz
AQ	1.0027661 sec
RG	5790
DW	15.300 usec
DE	6.00 usec
TE	298.8 K
D1	2.00000000 sec
d11	0.03000000 sec
DELTA	1.89999998 sec
TD0	10

======== CHANNEL f1 ========

NUC1	13C
P1	12.20 usec
PL1	3.00 dB
SFO1	125.7464750 MHz

======== CHANNEL f2 ========

CPDPRG2	waltz16
NUC2	1H
PCPD2	80.00 usec
PL2	2.00 dB
PL12	17.70 dB
PL13	17.70 dB
SFO2	500.0355000 MHz
SI	32768
SF	125.7326504 MHz
WDW	EM
SSB	0
LB	6.00 Hz
GB	0
PC	2.00

(*S,E*)-*tert*-butyl 3-(1,3-di-*p*-tolylallyl)-1H-indole-1-carboxylate **(Boc-3p)**

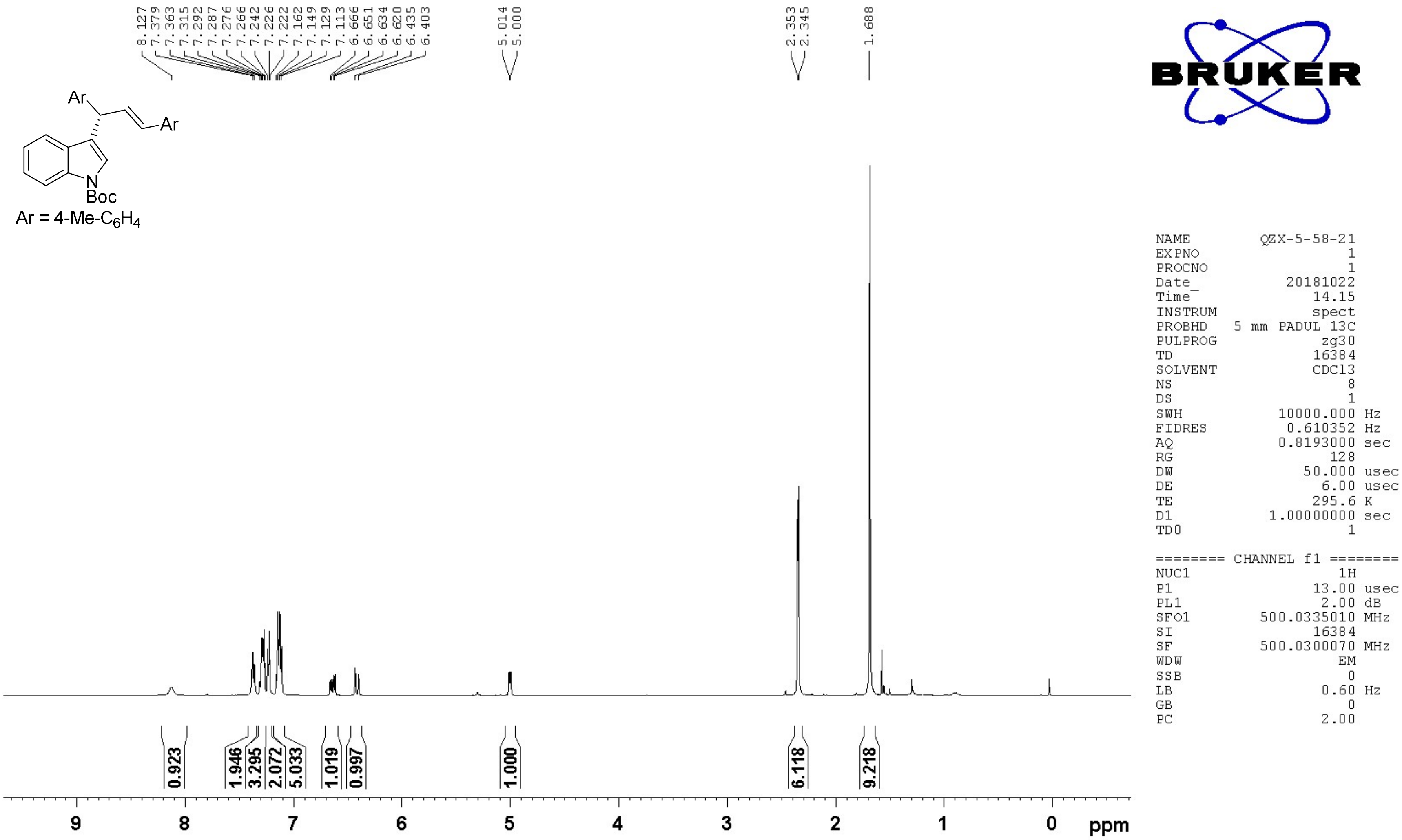

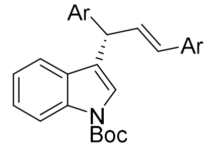

Ar = 4-Me-C_6H_4

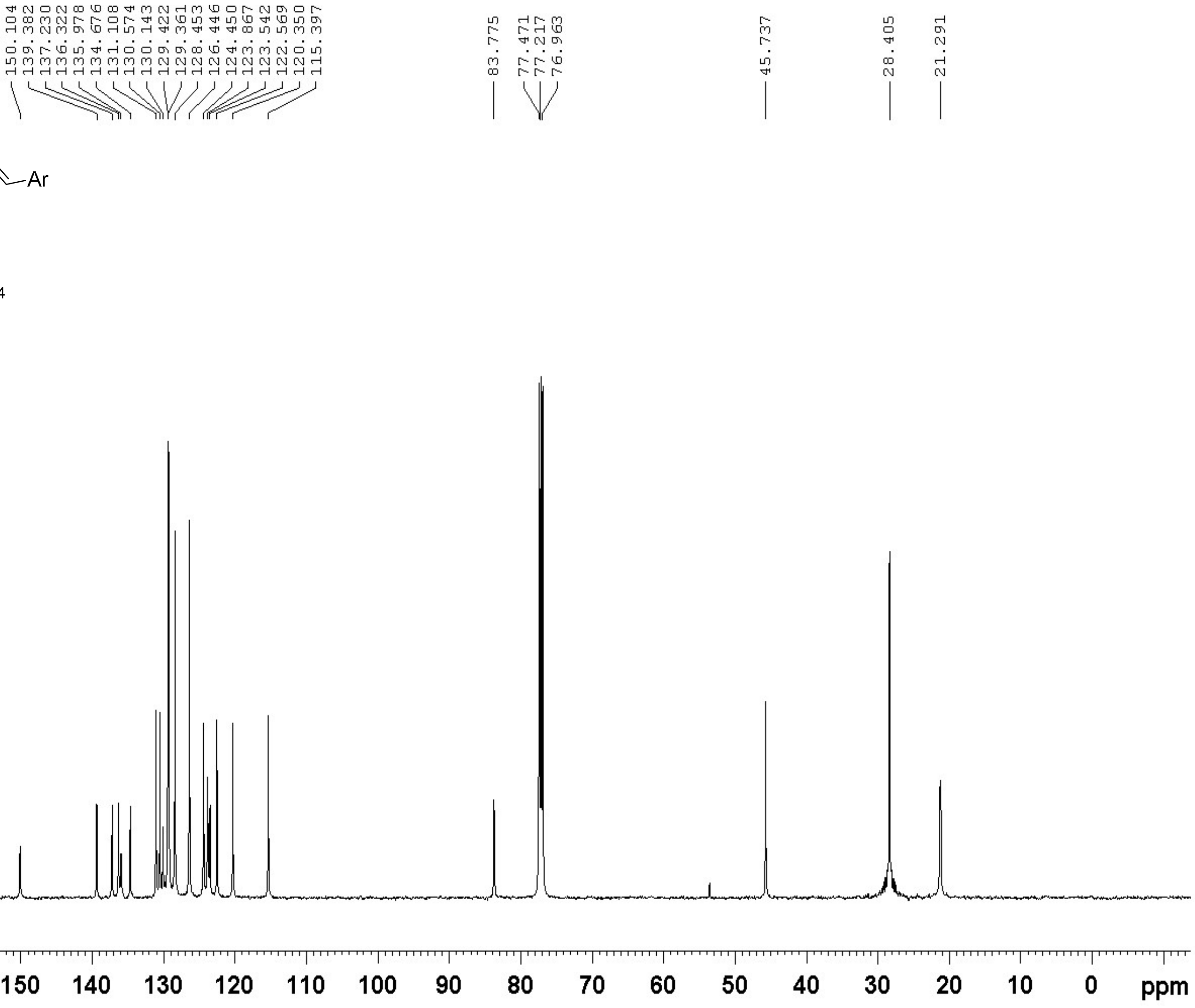

NAME	QZX-5-58-22
EXPNO	2
PROCNO	1
Date_	20181022
Time	13.28
INSTRUM	spect
PROBHD	5 mm PADUL 13C
PULPROG	zgpg30
TD	65536
SOLVENT	CDCl3
NS	1398
DS	2
SWH	32679.738 Hz
FIDRES	0.498653 Hz
AQ	1.0027661 sec
RG	8200
DW	15.300 usec
DE	6.00 usec
TE	297.8 K
D1	2.00000000 sec
d11	0.03000000 sec
DELTA	1.89999998 sec
TD0	10

======== CHANNEL f1 ========

NUC1	13C
P1	12.20 usec
PL1	3.00 dB
SFO1	125.7464750 MHz

======== CHANNEL f2 ========

CPDPRG2	waltz16
NUC2	1H
PCPD2	80.00 usec
PL2	2.00 dB
PL12	17.70 dB
PL13	17.70 dB
SFO2	500.0355000 MHz
SI	32768
SF	125.7326273 MHz
WDW	EM
SSB	0
LB	8.00 Hz
GB	0
PC	2.00

(*S,E*)-*tert*-butyl 3-(1,3-bis(4-methoxyphenyl)allyl)-1H-indole-1-carboxylate **(Boc-3q)**

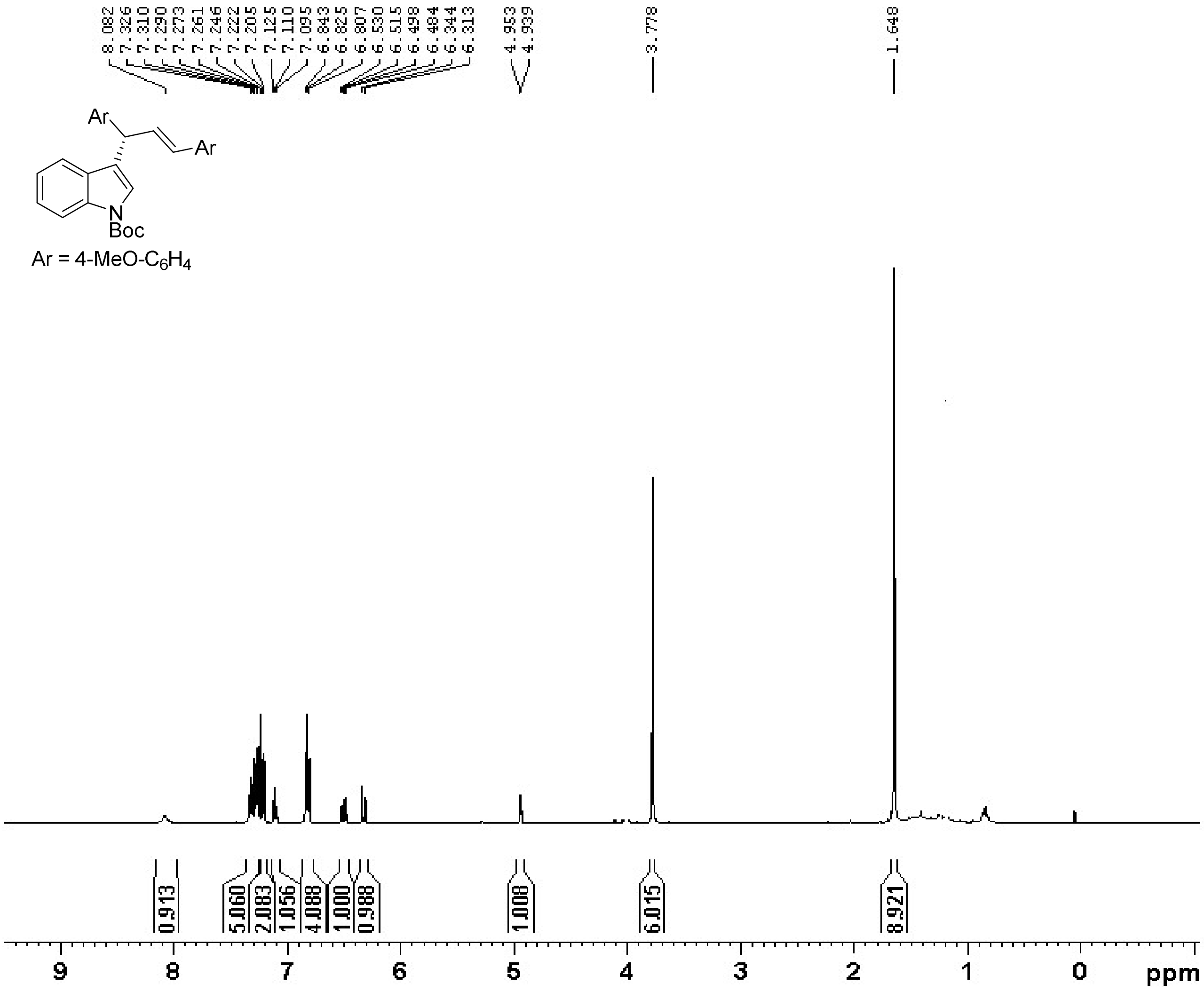

NAME QZX-5-195-1
EXPNO 1
PROCNO 1
Date_ 20190410
Time 11.18
INSTRUM spect
PROBHD 5 mm PABBO BB-
PULPROG zg30
TD 16384
SOLVENT CDCl3
NS 8
DS 0
SWH 10000.000 Hz
FIDRES 0.610352 Hz
AQ 0.8193000 sec
RG 456
DW 50.000 usec
DE 8.00 usec
TE 292.3 K
D1 2.00000000 sec
TD0 1

======== CHANNEL f1 ========
NUC1 1H
P1 13.00 usec
PL1 2.00 dB
SFO1 500.0335000 MHz
SI 16384
SF 500.0300171 MHz
WDW EM
SSB 0
LB 0.30 Hz
GB 0
PC 1.00

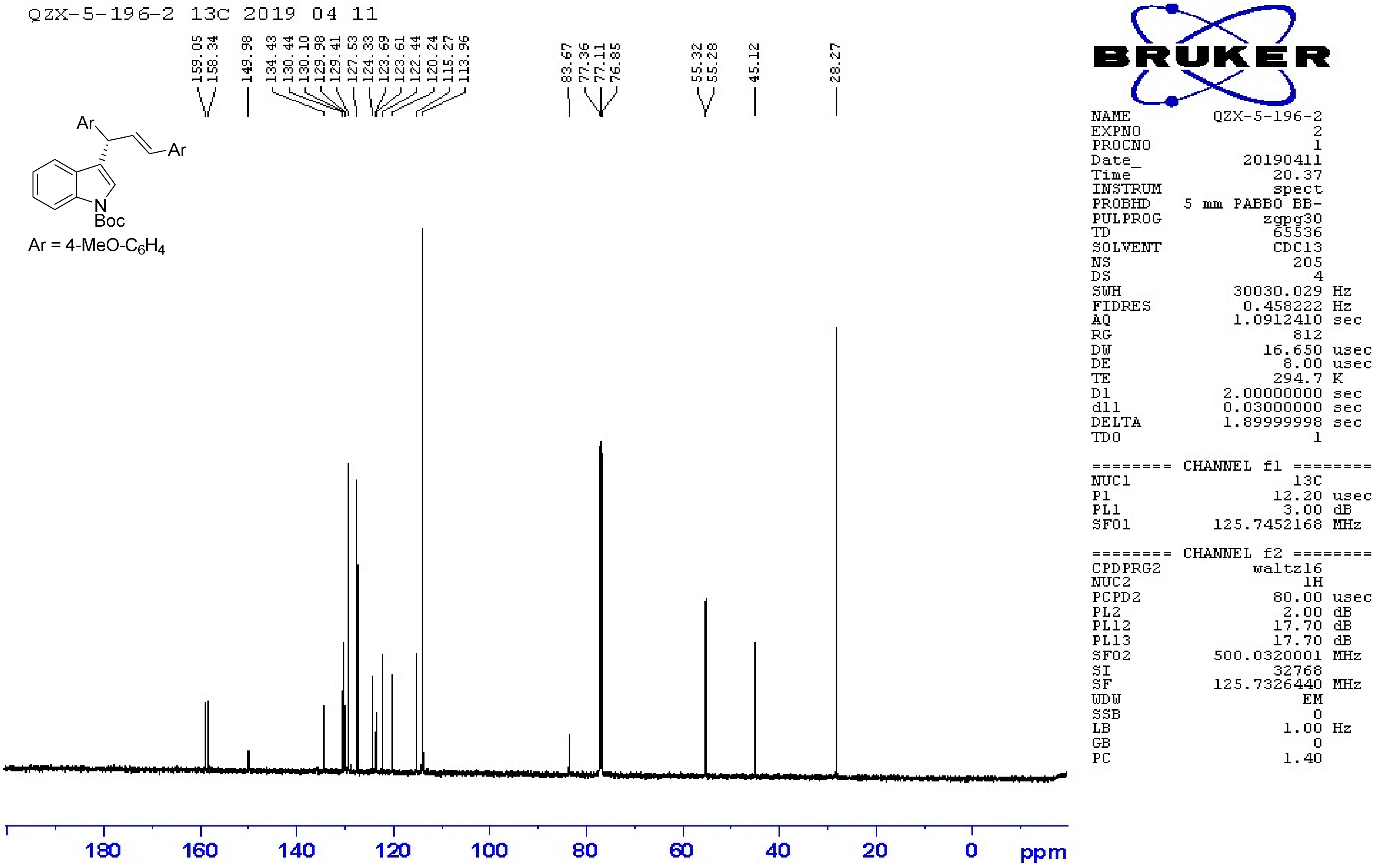

QZX-5-196-2 13C 2019 04 11
159.05
158.34
149.98
134.43
130.44
130.10
129.98
129.41
127.53
124.33
123.69
123.61
122.44
120.24
115.27
113.96
83.67
77.36
77.11
76.85
55.32
55.28
45.12
28.27
Ar
Ar
N
Boc
Ar = 4-MeO-C6H4
BRUKER
NAME QZX-5-196-2
EXPNO 2
PROCNO 1
Date_ 20190411
Time 20.37
INSTRUM spect
PROBHD 5 mm PABBO BB-
PULPROG zgpg30
TD 65536
SOLVENT CDCl3
NS 205
DS 4
SWH 30030.029 Hz
FIDRES 0.458222 Hz
AQ 1.0912410 sec
RG 812
DW 16.650 usec
DE 8.00 usec
TE 294.7 K
D1 2.00000000 sec
d11 0.03000000 sec
DELTA 1.89999998 sec
TD0 1
======== CHANNEL f1 ========
NUC1 13C
P1 12.20 usec
PL1 3.00 dB
SFO1 125.7452168 MHz
======== CHANNEL f2 ========
CPDPRG2 waltz16
NUC2 1H
PCPD2 80.00 usec
PL2 2.00 dB
PL12 17.70 dB
PL13 17.70 dB
SFO2 500.0320001 MHz
SI 32768
SF 125.7326440 MHz
WDW EM
SSB 0
LB 1.00 Hz
GB 0
PC 1.40
180
160
140
120
100
80
60
40
20
0
ppm

(*S*,*E*)-*tert*-butyl 3-(1,3-bis(4-chlorophenyl)allyl)-1H-indole-1-carboxylate **(Boc-3r)**

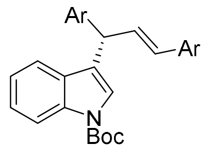

Ar = 4-Cl-C_6H_4

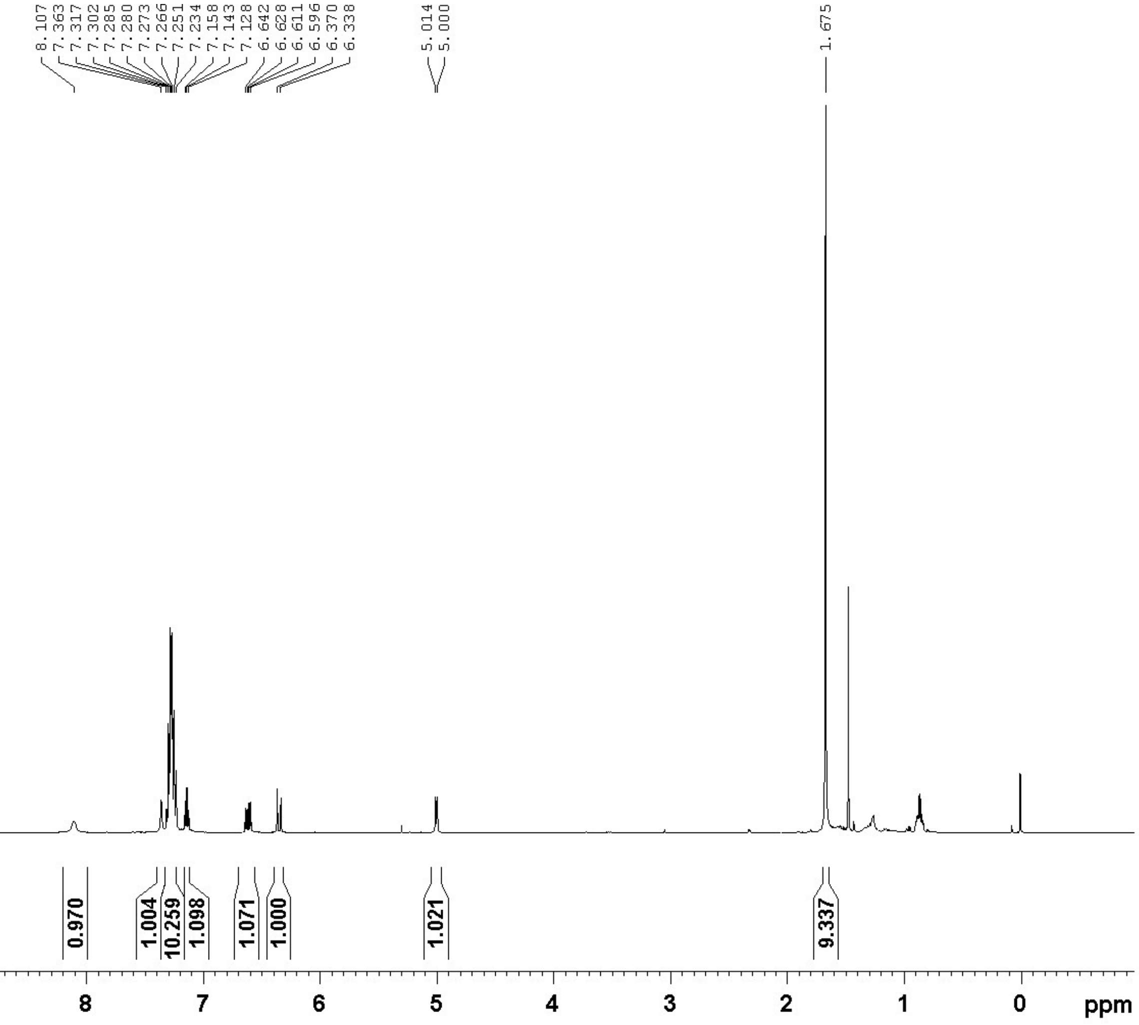

NAME	QZX-5-56-21
EXPNO	1
PROCNO	1
Date_	20181022
Time	14.18
INSTRUM	spect
PROBHD	5 mm PADUL 13C
PULPROG	zg30
TD	16384
SOLVENT	CDCl3
NS	8
DS	1
SWH	10000.000 Hz
FIDRES	0.610352 Hz
AQ	0.8193000 sec
RG	322
DW	50.000 usec
DE	6.00 usec
TE	295.5 K
D1	1.00000000 sec
TD0	1

======== CHANNEL f1 ========

NUC1	1H
P1	13.00 usec
PL1	2.00 dB
SFO1	500.0335010 MHz
SI	16384
SF	500.0300070 MHz
WDW	EM
SSB	0
LB	0.60 Hz
GB	0
PC	2.00

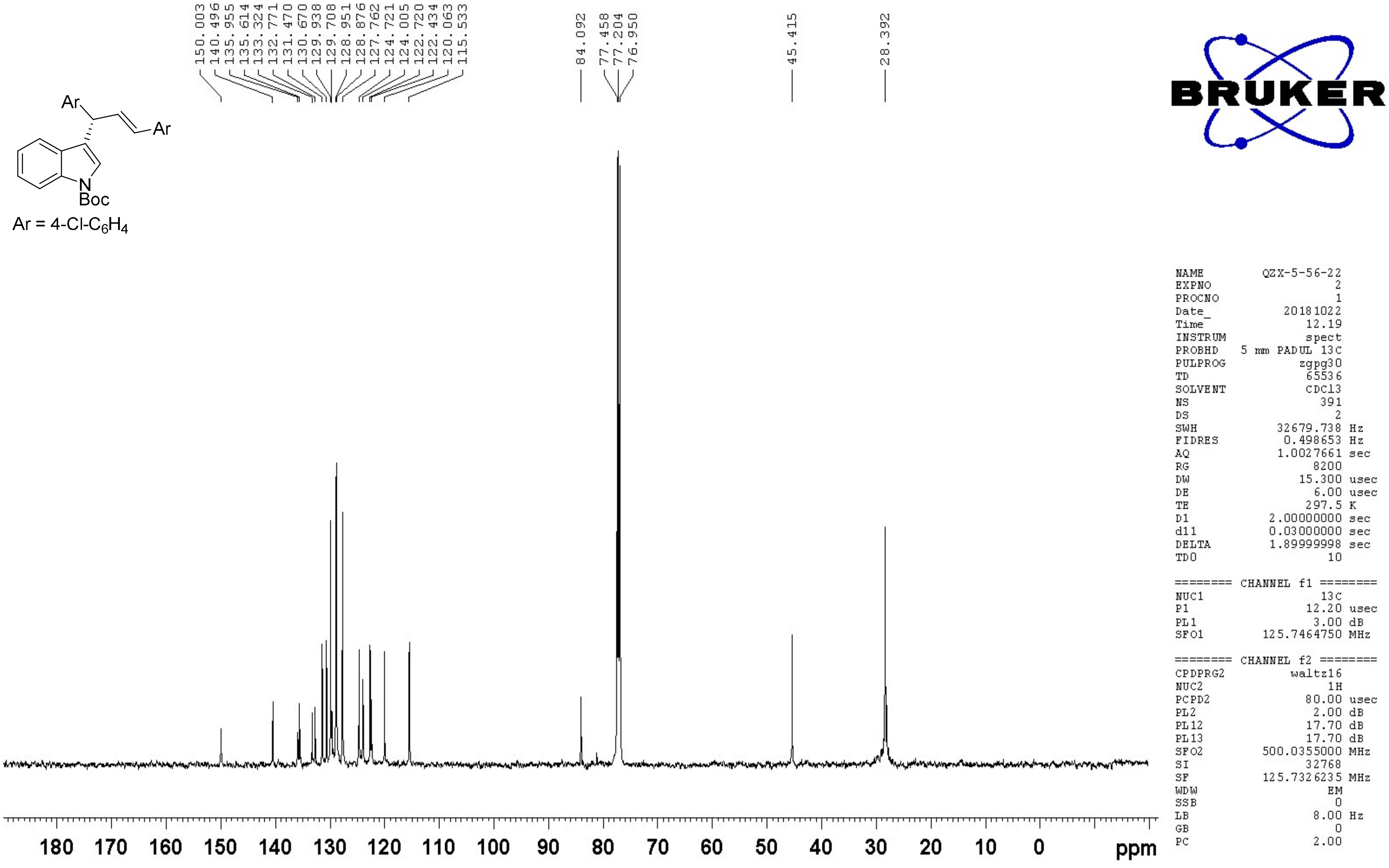
Ar
Ar
N
Boc
Ar = 4-Cl-C6H4
150.003
140.496
135.955
135.614
133.324
132.771
131.470
130.670
129.938
129.708
128.951
128.876
127.762
124.721
124.005
122.720
122.434
120.063
115.533
84.092
77.458
77.204
76.950
45.415
28.392
BRUKER
NAME QZX-5-56-22
EXPNO 2
PROCNO 1
Date_ 20181022
Time 12.19
INSTRUM spect
PROBHD 5 mm PADUL 13C
PULPROG zgpg30
TD 65536
SOLVENT CDCl3
NS 391
DS 2
SWH 32679.738 Hz
FIDRES 0.498653 Hz
AQ 1.0027661 sec
RG 8200
DW 15.300 usec
DE 6.00 usec
TE 297.5 K
D1 2.00000000 sec
d11 0.03000000 sec
DELTA 1.89999998 sec
TD0 10
======== CHANNEL f1 ========
NUC1 13C
P1 12.20 usec
PL1 3.00 dB
SFO1 125.7464750 MHz
======== CHANNEL f2 ========
CPDPRG2 waltz16
NUC2 1H
PCPD2 80.00 usec
PL2 2.00 dB
PL12 17.70 dB
PL13 17.70 dB
SFO2 500.0355000 MHz
SI 32768
SF 125.7326235 MHz
WDW EM
SSB 0
LB 8.00 Hz
GB 0
PC 2.00
180 170 160 150 140 130 120 110 100 90 80 70 60 50 40 30 20 10 0 ppm

(*S,E*)-*tert*-butyl 3-(1,3-bis(4-nitrophenyl)allyl)-1H-indole-1-carboxylate **(Boc-3s)**

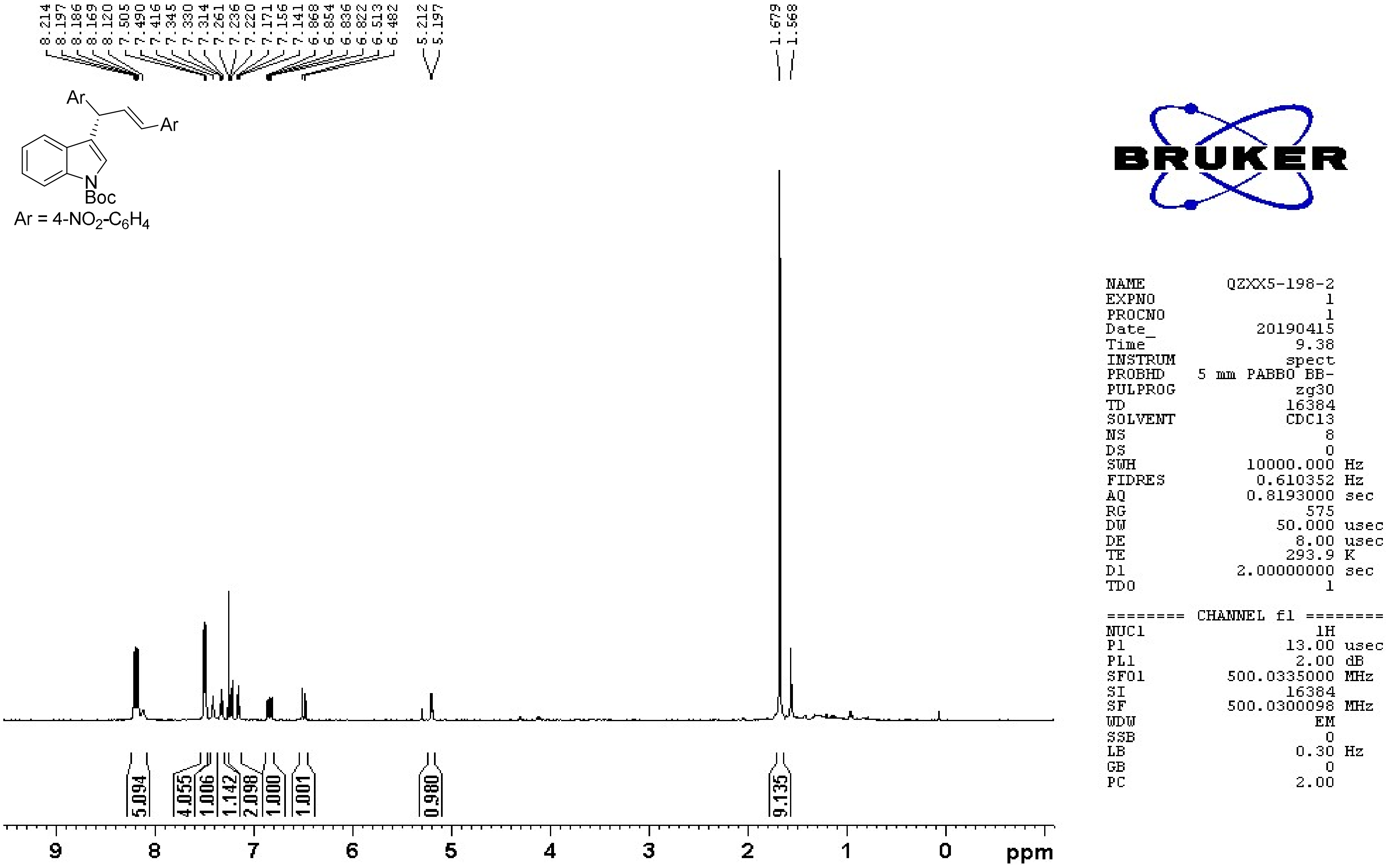

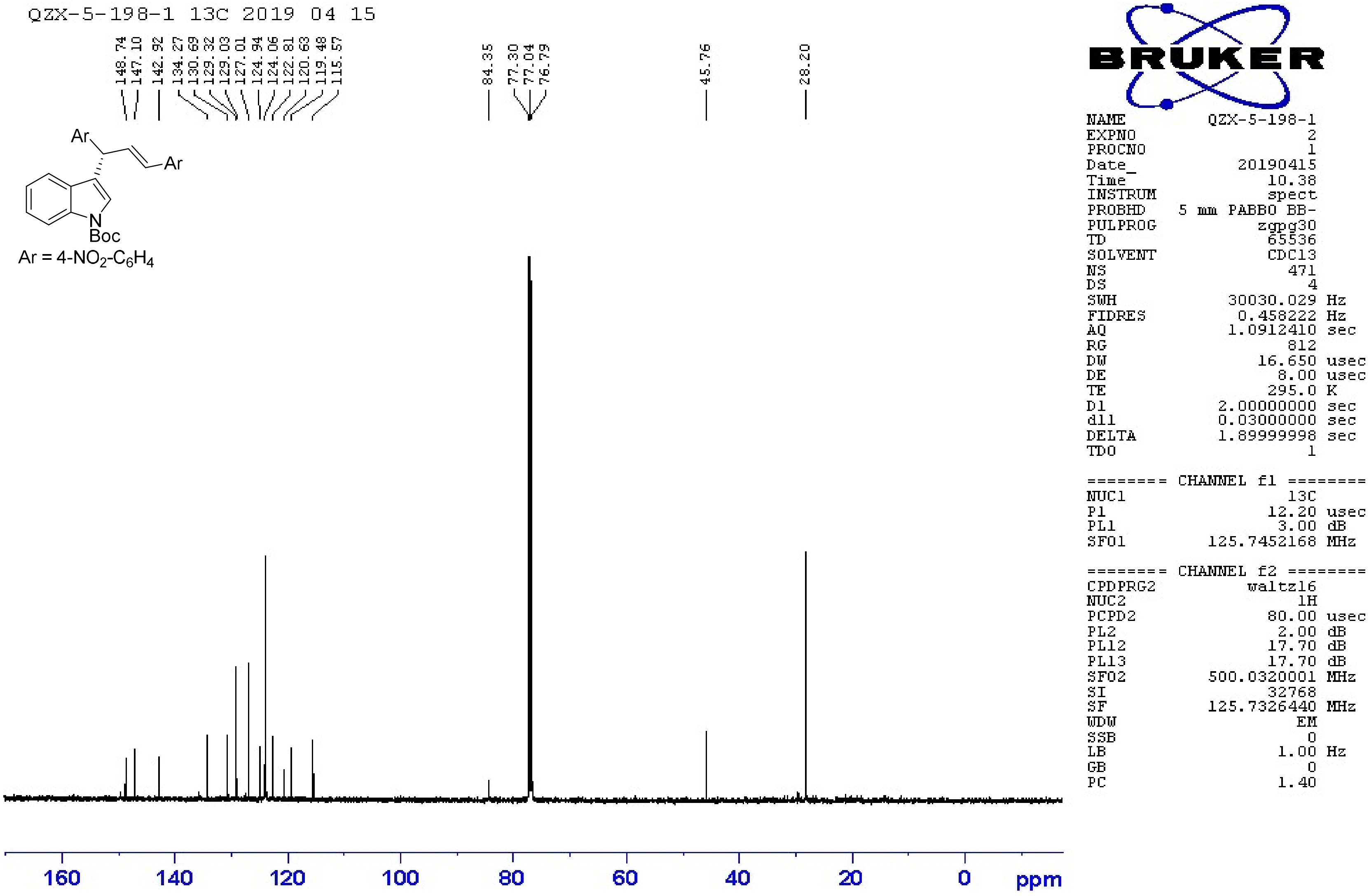
QZX-5-198-1 13C 2019 04 15
148.74
147.10
142.92
134.27
130.69
129.32
129.03
127.01
124.94
124.06
122.81
120.63
119.48
115.57
84.35
77.30
77.04
76.79
45.76
28.20
Ar
Ar
N
Boc
Ar = 4-NO2-C6H4
BRUKER
NAME QZX-5-198-1
EXPNO 2
PROCNO 1
Date_ 20190415
Time 10.38
INSTRUM spect
PROBHD 5 mm PABBO BB-
PULPROG zgpg30
TD 65536
SOLVENT CDCl3
NS 471
DS 4
SWH 30030.029 Hz
FIDRES 0.458222 Hz
AQ 1.0912410 sec
RG 812
DW 16.650 usec
DE 8.00 usec
TE 295.0 K
D1 2.00000000 sec
d11 0.03000000 sec
DELTA 1.89999998 sec
TD0 1
======== CHANNEL f1 ========
NUC1 13C
P1 12.20 usec
PL1 3.00 dB
SFO1 125.7452168 MHz
======== CHANNEL f2 ========
CPDPRG2 waltz16
NUC2 1H
PCPD2 80.00 usec
PL2 2.00 dB
PL12 17.70 dB
PL13 17.70 dB
SFO2 500.0320001 MHz
SI 32768
SF 125.7326440 MHz
WDW EM
SSB 0
LB 1.00 Hz
GB 0
PC 1.40
160
140
120
100
80
60
40
20
0
ppm

(*S*,*E*)-(3-(benzyloxy)prop-1-ene-1,3-diyl)dibenzene **(5a)**

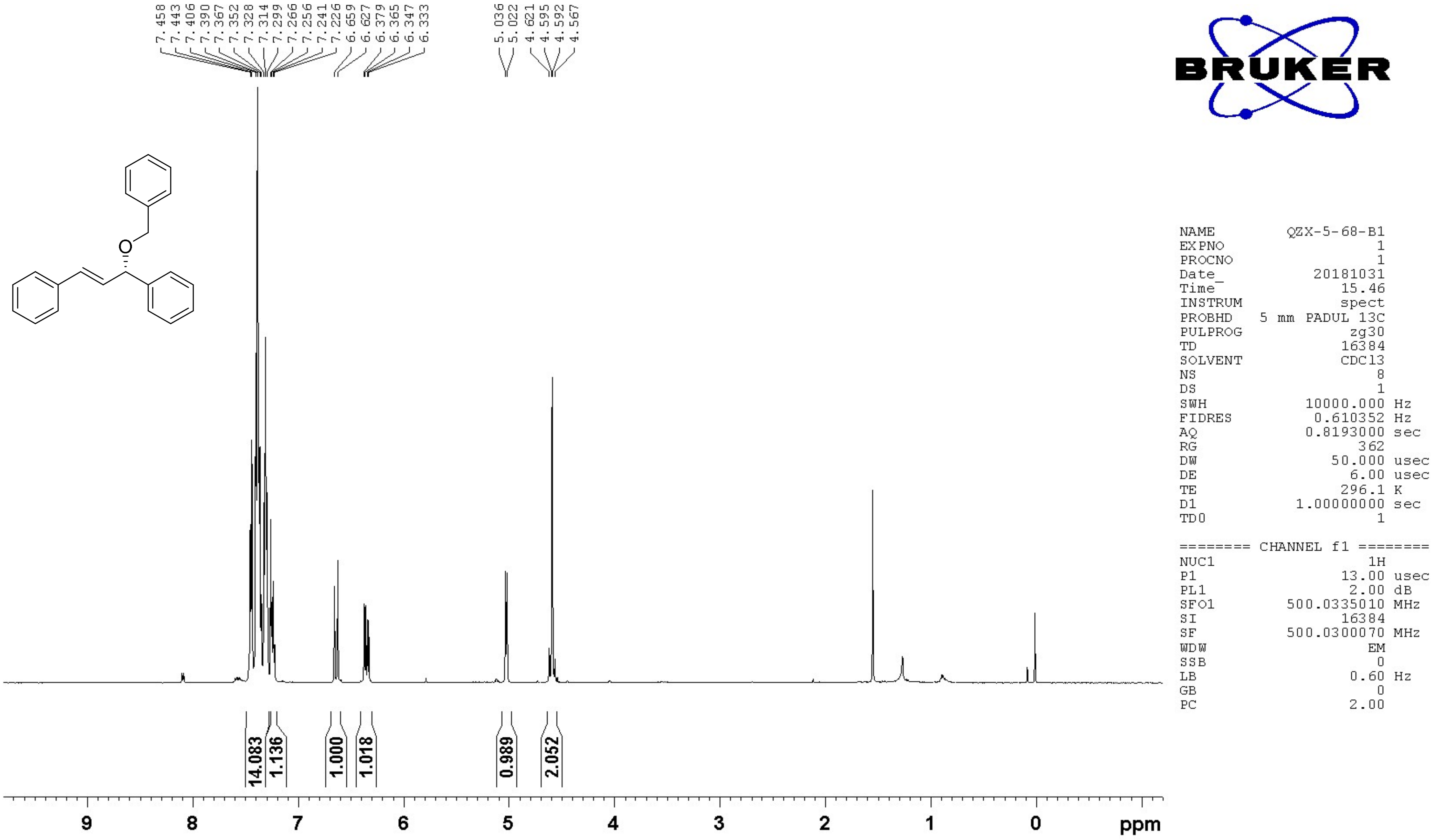

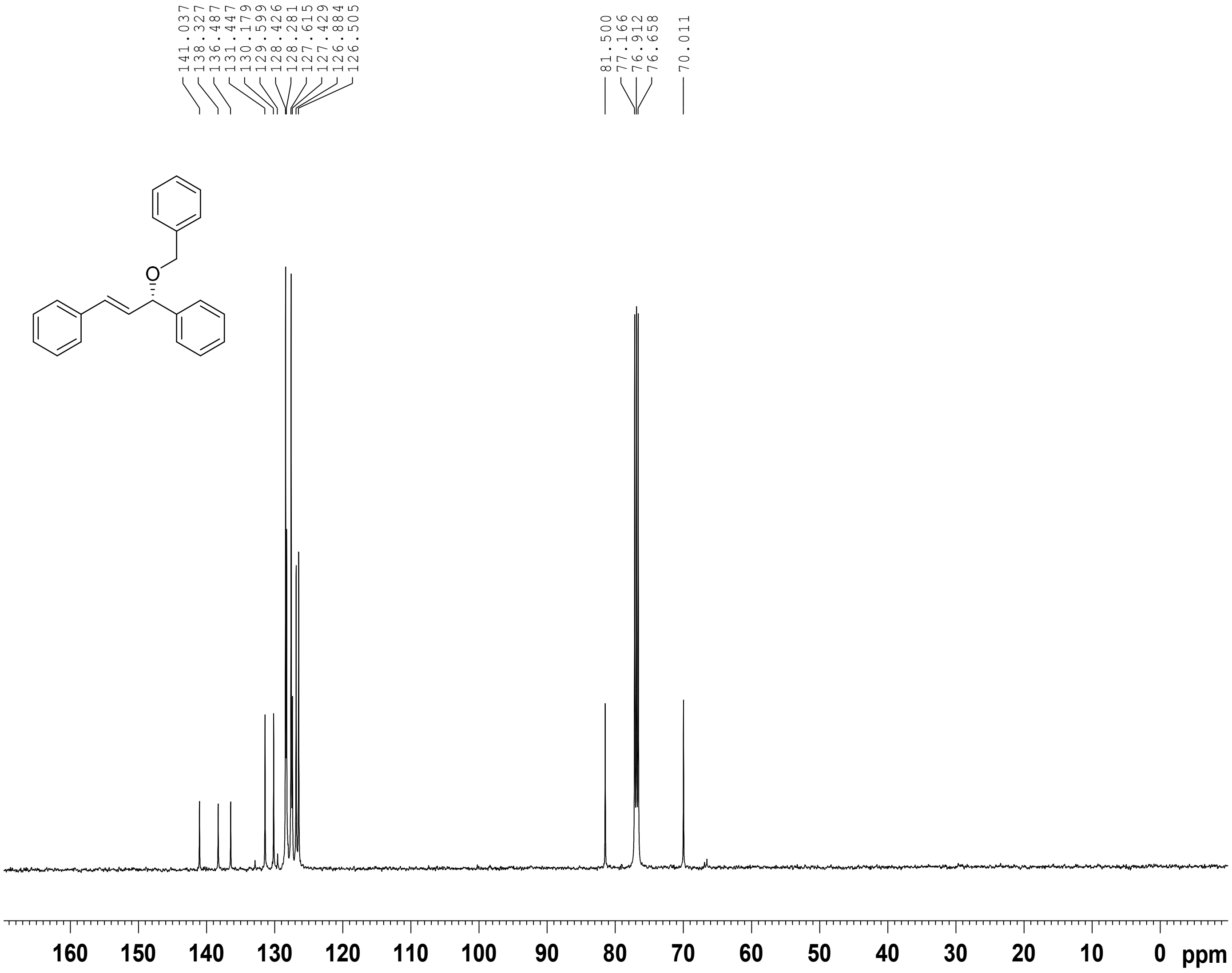

NAME	QZX-5-68B2
EXPNO	2
PROCNO	1
Date_	20181031
Time	14.06
INSTRUM	spect
PROBHD	5 mm PADUL 13C
PULPROG	zgpg30
TD	65536
SOLVENT	CDCl3
NS	1292
DS	2
SWH	32679.738 Hz
FIDRES	0.498653 Hz
AQ	1.0027661 sec
RG	5160
DW	15.300 usec
DE	6.00 usec
TE	298.1 K
D1	2.00000000 sec
d11	0.03000000 sec
DELTA	1.89999998 sec
TD0	10

======== CHANNEL f1 ========

NUC1	13C
P1	12.20 usec
PL1	3.00 dB
SFO1	125.7464750 MHz

======== CHANNEL f2 ========

CPDPRG2	waltz16
NUC2	1H
PCPD2	80.00 usec
PL2	2.00 dB
PL12	17.70 dB
PL13	17.70 dB
SFO2	500.0355000 MHz
SI	32768
SF	125.7326615 MHz
WDW	EM
SSB	0
LB	6.00 Hz
GB	0
PC	2.00

(*S*,*E*)-(3-((4-methoxybenzyl)oxy)prop-1-ene-1,3-diyl)dibenzene **(5b)**

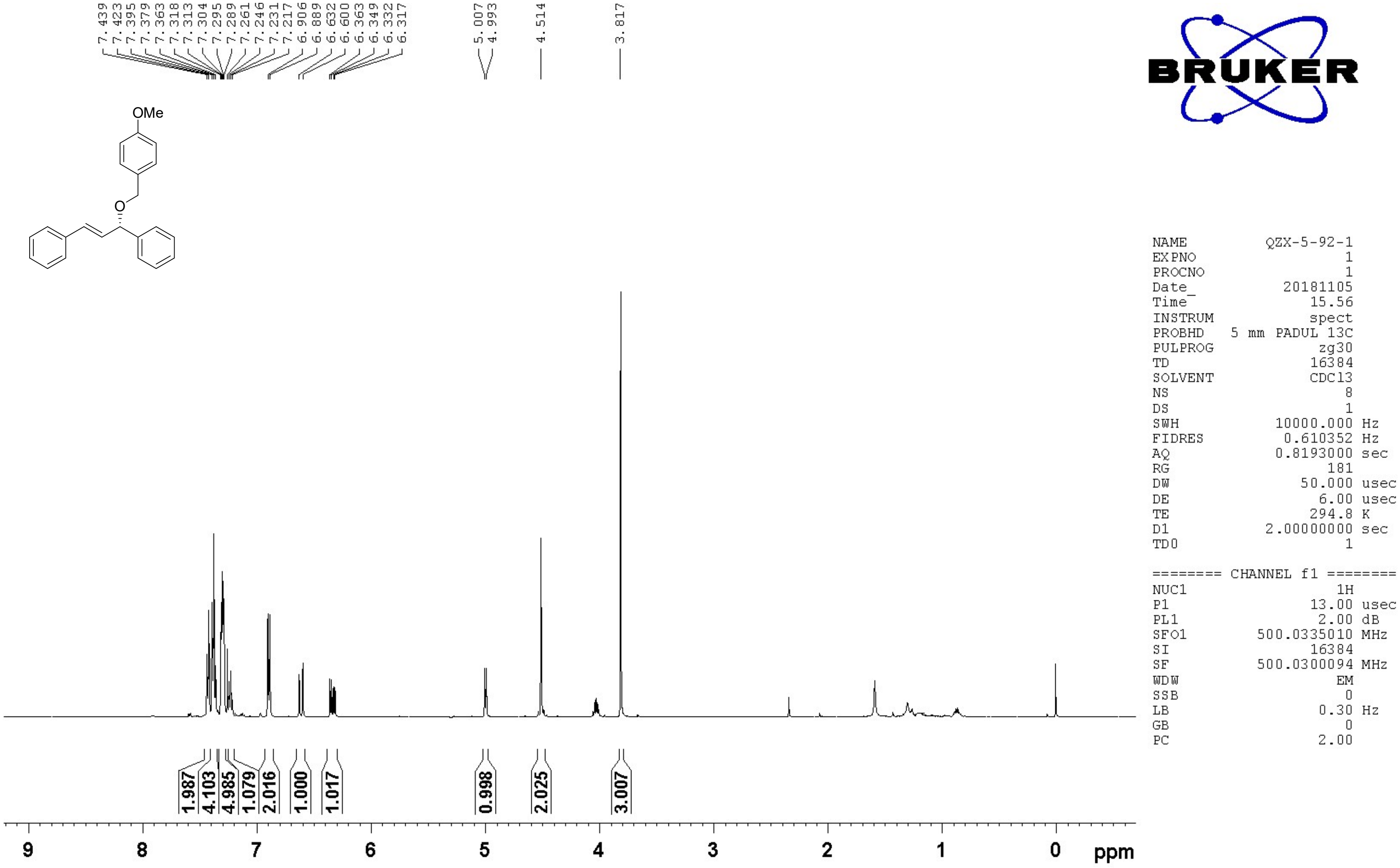

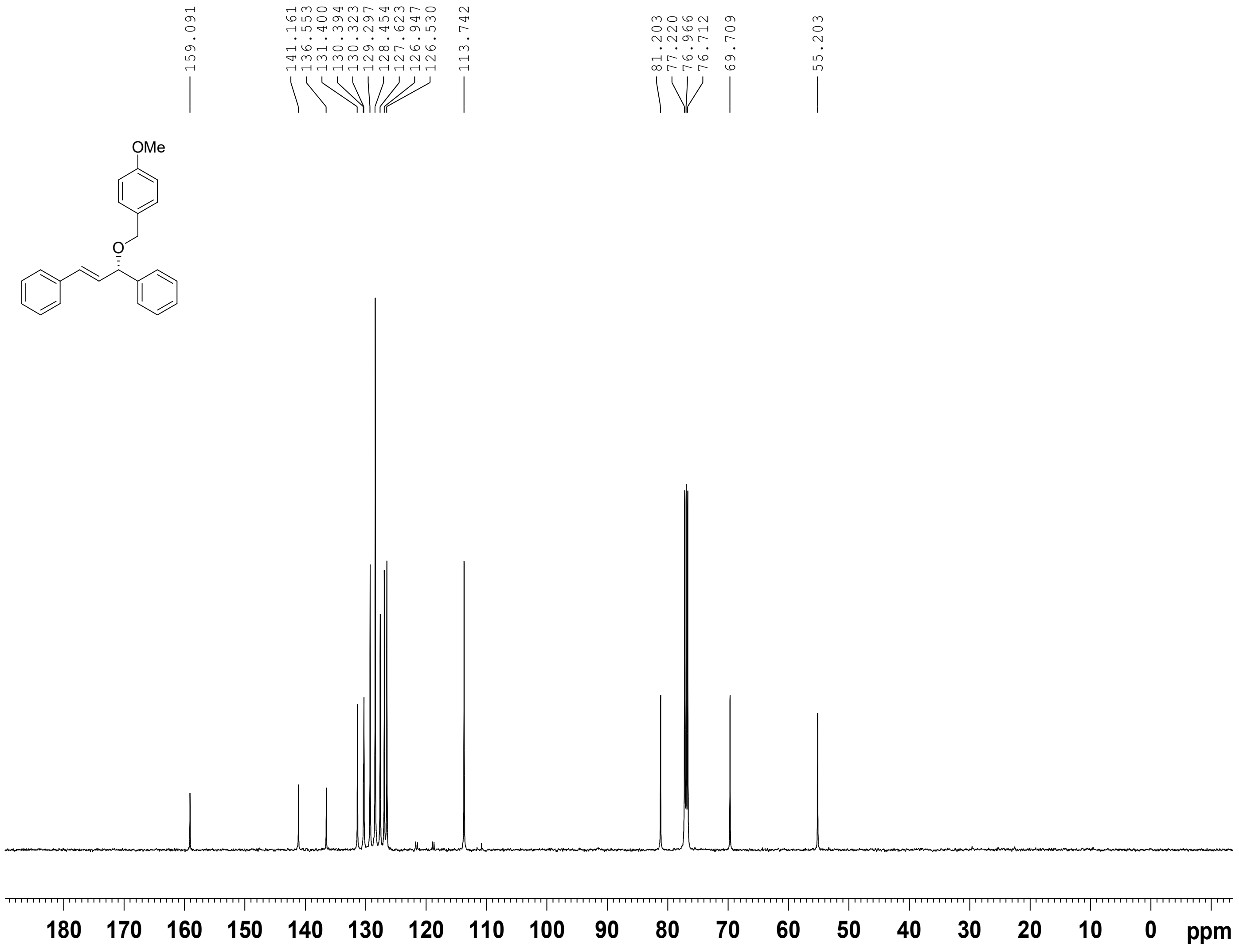


```
NAME            QZX-5-92-2
EXPNO                   21
PROCNO                   1
Date_             20181105
Time                 13.11
INSTRUM              spect
PROBHD   5 mm PADUL 13C
PULPROG             zgpg30
TD                   65536
SOLVENT              CDCl3
NS                     883
DS                       2
SWH              32679.738 Hz
FIDRES            0.498653 Hz
AQ               1.0027661 sec
RG                    8200
DW                  15.300 usec
DE                    6.00 usec
TE                   297.0 K
D1              2.00000000 sec
d11             0.03000000 sec
DELTA           1.89999998 sec
TD0                     10

======== CHANNEL f1 ========
NUC1                   13C
P1                   12.20 usec
PL1                   3.00 dB
SFO1           125.7464750 MHz

======== CHANNEL f2 ========
CPDPRG2            waltz16
NUC2                    1H
PCPD2                80.00 usec
PL2                   2.00 dB
PL12                 17.70 dB
PL13                 17.70 dB
SFO2           500.0355000 MHz
SI                   32768
SF             125.7326577 MHz
WDW                     EM
SSB                      0
LB                    6.00 Hz
GB                       0
PC                    2.00
```

(*S*,*E*)-(3-((4-bromobenzyl)oxy)prop-1-ene-1,3-diyl)dibenzene **(5c)**

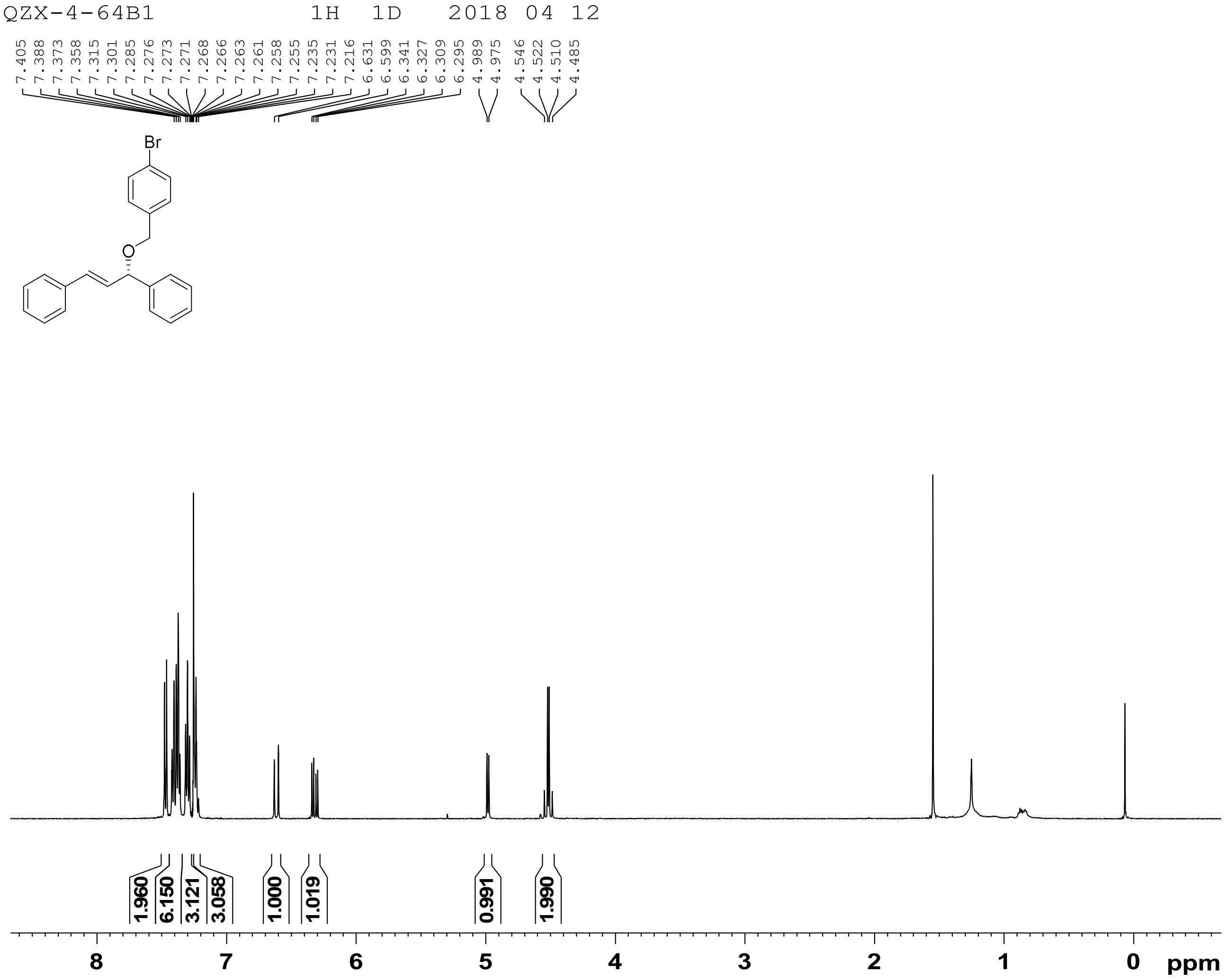


```
NAME           QZX-4-64B1
EXPNO                   1
PROCNO                  1
Date_            20180412
Time                16.20
INSTRUM             spect
PROBHD   5 mm PABBO BB-
PULPROG              zg30
TD                  16384
SOLVENT             CDCl3
NS                      8
DS                      0
SWH             10000.000 Hz
FIDRES           0.610352 Hz
AQ              0.8193000 sec
RG                    645
DW                 50.000 usec
DE                   8.00 usec
TE                  295.4 K
D1             2.00000000 sec
TD0                     1

======== CHANNEL f1 ========
NUC1                   1H
P1                  13.00 usec
PL1                  2.00 dB
SFO1          500.0335000 MHz
SI                  16384
SF            500.0300132 MHz
WDW                    no
SSB                     0
LB                   0.00 Hz
GB                      0
PC                   1.00
```

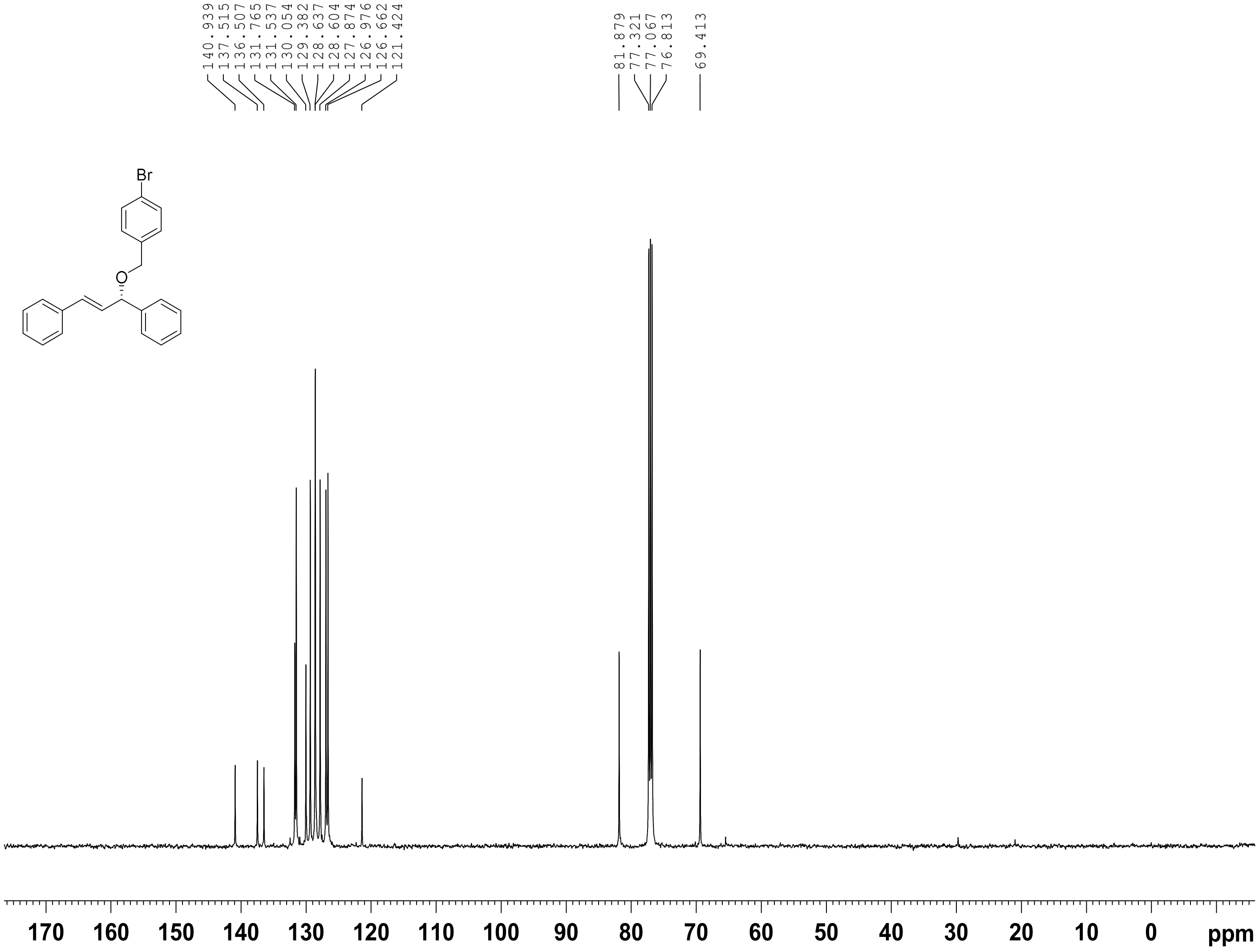

NAME	QZX-5-37B4
EXPNO	2
PROCNO	1
Date_	20181010
Time	2.15
INSTRUM	spect
PROBHD	5 mm PADUL 13C
PULPROG	zgpg30
TD	65536
SOLVENT	CDCl3
NS	1024
DS	4
SWH	30030.029 Hz
FIDRES	0.458222 Hz
AQ	1.0912410 sec
RG	144
DW	16.650 usec
DE	8.00 usec
TE	298.3 K
D1	2.00000000 sec
d11	0.03000000 sec
DELTA	1.89999998 sec
TD0	1
======== CHANNEL f1 ========	
NUC1	13C
P1	12.20 usec
PL1	4.00 dB
SFO1	125.7452168 MHz
======== CHANNEL f2 ========	
CPDPRG2	waltz16
NUC2	1H
PCPD2	80.00 usec
PL2	2.00 dB
PL12	18.00 dB
PL13	18.00 dB
SFO2	500.0320001 MHz
SI	32768
SF	125.7326420 MHz
WDW	EM
SSB	0
LB	6.00 Hz
GB	0
PC	1.40

(*S*,*E*)-(3-((3-bromobenzyl)oxy)prop-1-ene-1,3-diyl)dibenzene **(5d)**

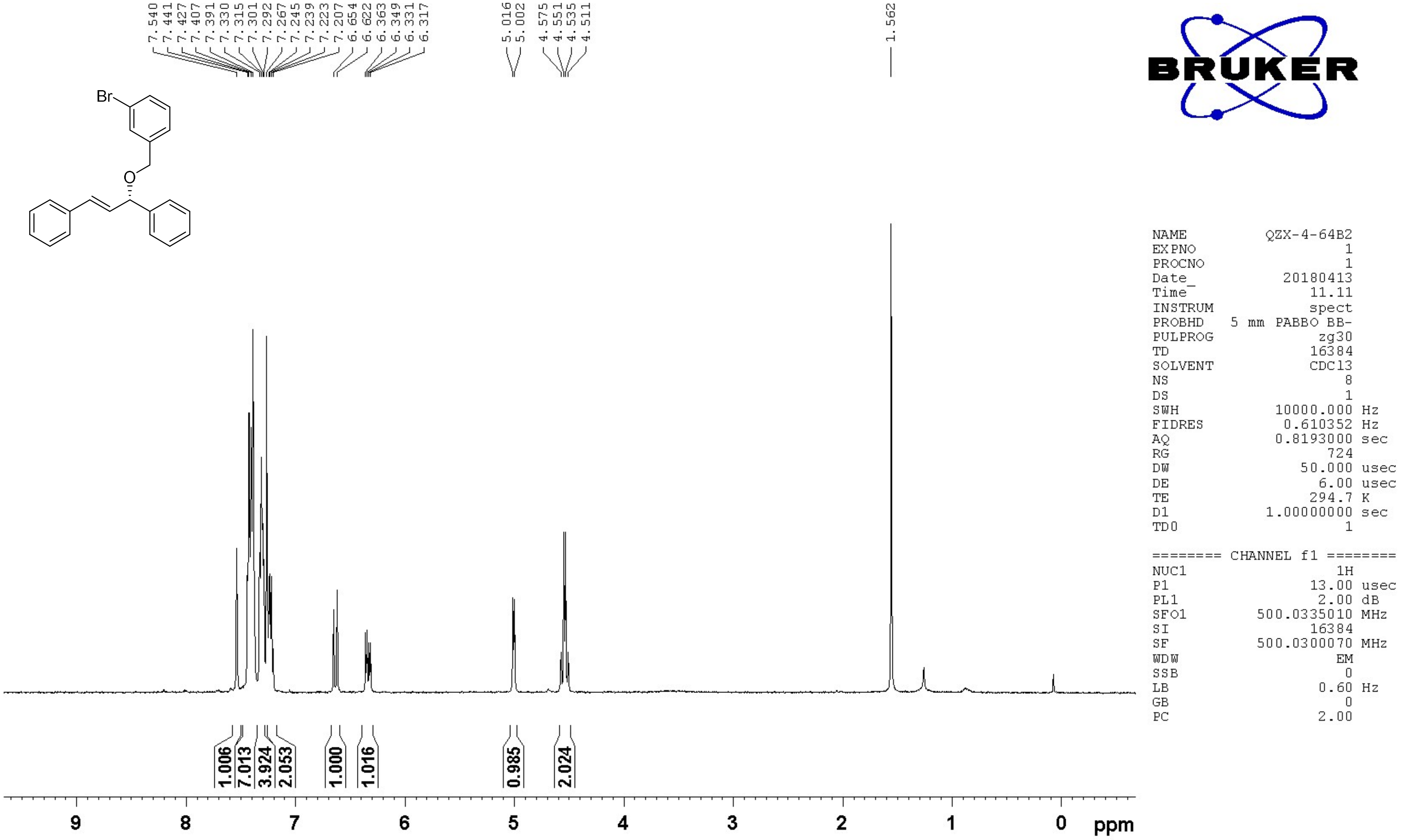

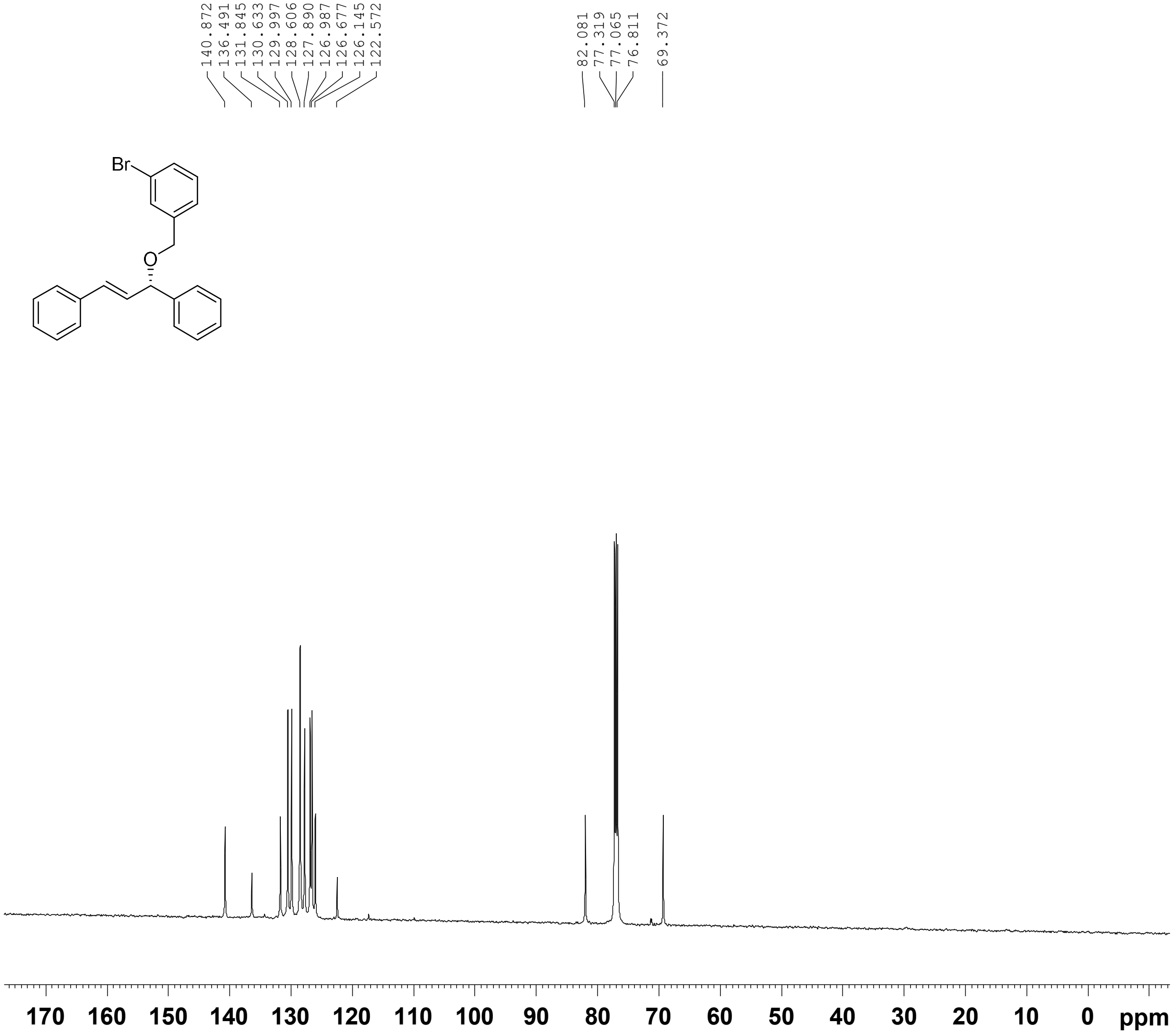

NAME	QZX-5-96-2-110
EXPNO	2
PROCNO	1
Date_	20181107
Time	13.51
INSTRUM	spect
PROBHD	5 mm PADUL 13C
PULPROG	zgpg30
TD	65536
SOLVENT	CDCl3
NS	1552
DS	1
SWH	32679.738
FIDRES	0.498653
AQ	1.0027661
RG	1820
DW	15.300
DE	6.00
TE	298.5
D1	2.00000000
d11	0.03000000
DELTA	1.89999998
TD0	20

======== CHANNEL f1 ===

NUC1	13C
P1	12.20
PL1	4.00
SFO1	125.7464750

======== CHANNEL f2 ===

CPDPRG2	waltz16
NUC2	1H
PCPD2	80.00
PL2	2.00
PL12	18.00

(*S*,*E*)-(3-((2-bromobenzyl)oxy)prop-1-ene-1,3-diyl)dibenzene **(5e)**

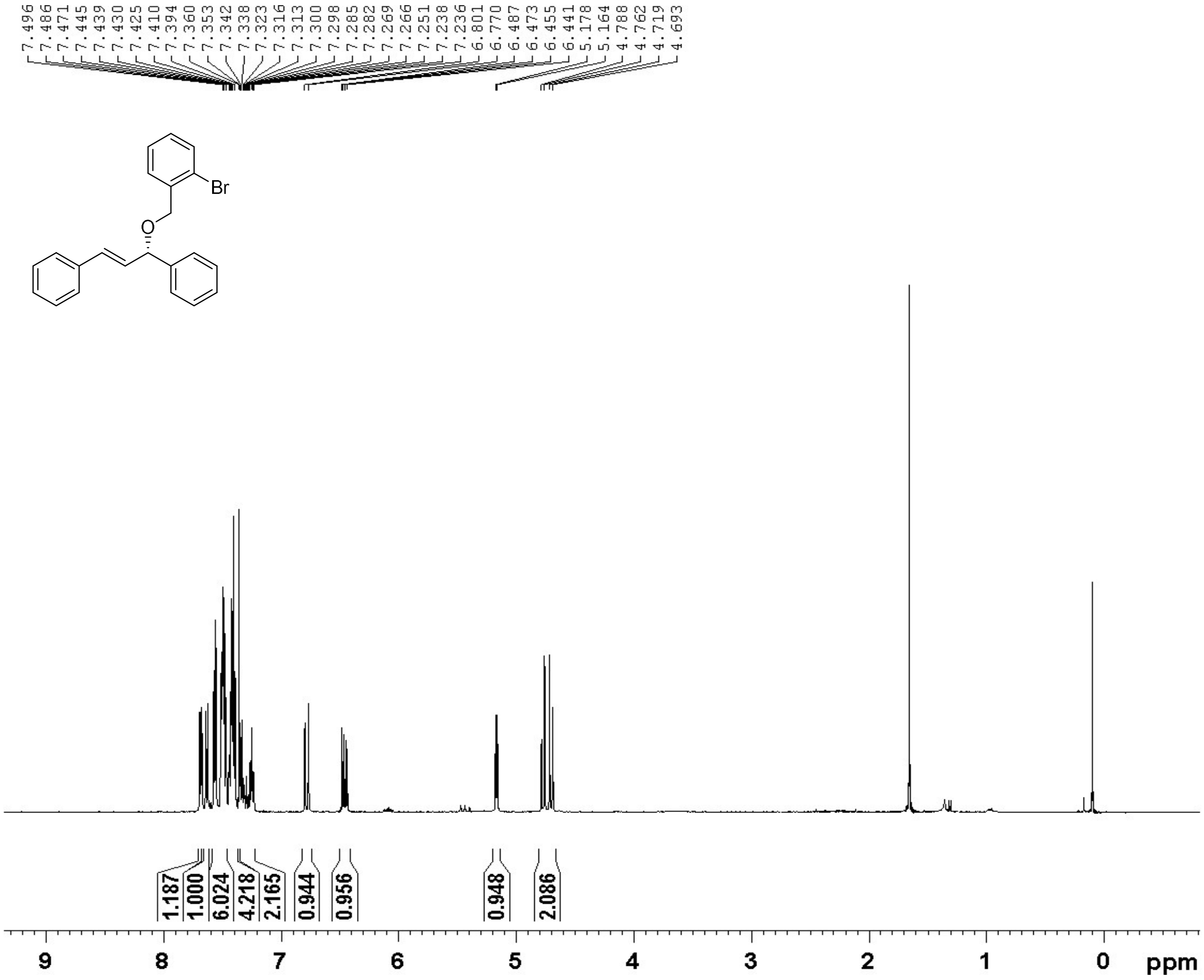

NAME	QZX-5-100-1
EXPNO	1
PROCNO	1
Date_	20181106
Time	17.49
INSTRUM	spect
PROBHD	5 mm PADUL 13C
PULPROG	zg30
TD	16384
SOLVENT	CDCl3
NS	8
DS	1
SWH	10000.000 Hz
FIDRES	0.610352 Hz
AQ	0.8193000 sec
RG	181
DW	50.000 usec
DE	6.00 usec
TE	295.6 K
D1	2.00000000 sec
TD0	1

======== CHANNEL f1 ========

NUC1	1H
P1	13.00 usec
PL1	2.00 dB
SFO1	500.0335010 MHz
SI	16384
SF	500.0299600 MHz
WDW	EM
SSB	0
LB	0.30 Hz
GB	0
PC	2.00

QZX-5-100-2 13C NMR 2018 11 08

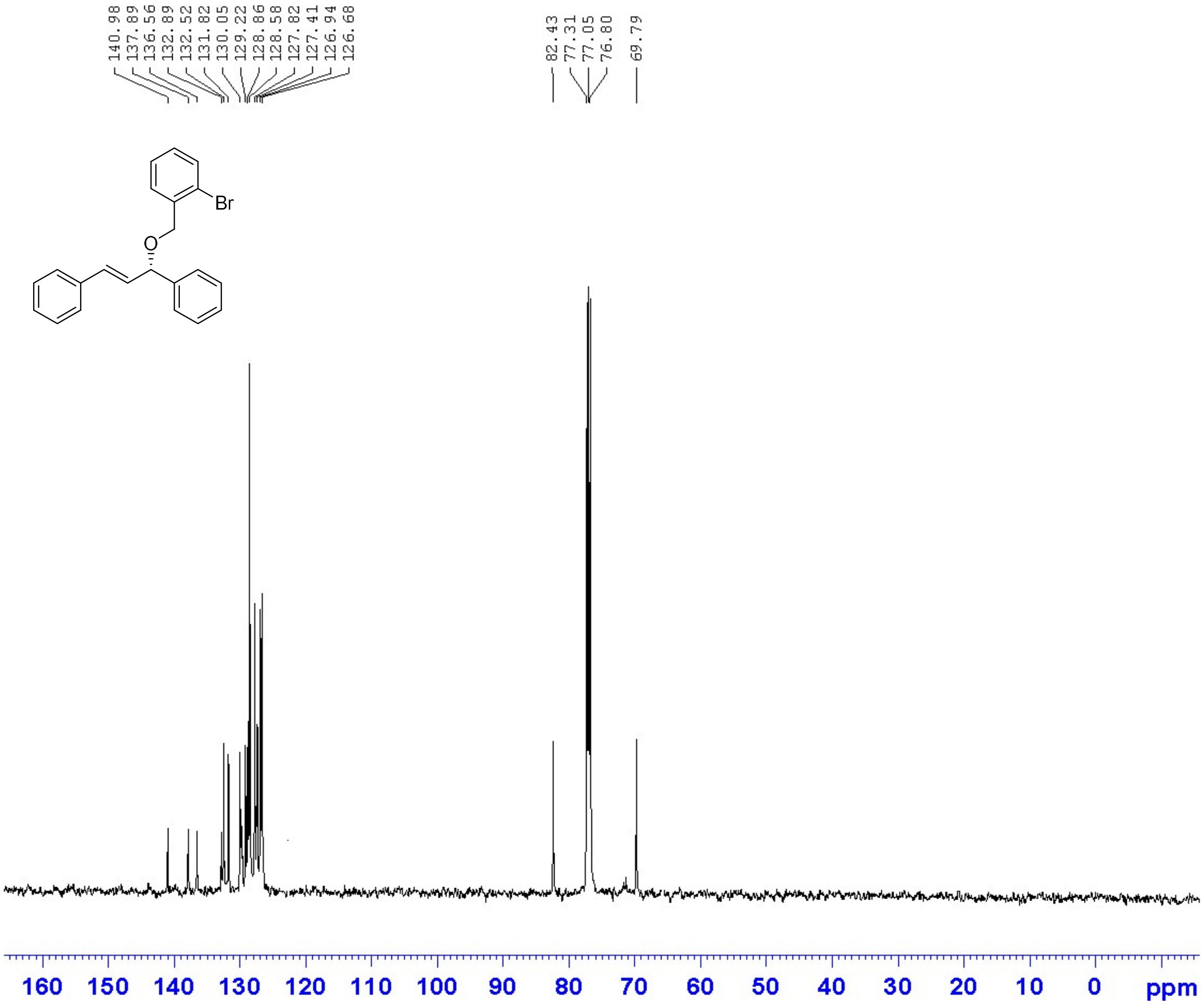

NAME	QZX-5-100-2	
EXPNO	2	
PROCNO	1	
Date_	20181108	
Time	11.47	
INSTRUM	spect	
PROBHD	5 mm PADUL 13C	
PULPROG	zgpg30	
TD	65536	
SOLVENT	CDCl3	
NS	260	
DS	2	
SWH	30030.029	Hz
FIDRES	0.458222	Hz
AQ	1.0912410	sec
RG	4100	
DW	16.650	usec
DE	8.00	usec
TE	297.1	K
D1	2.00000000	sec
d11	0.03000000	sec
DELTA	1.89999998	sec
TD0	10	

======== CHANNEL f1 ========

NUC1	13C	
P1	12.20	usec
PL1	4.00	dB
SFO1	125.7452170	MHz

======== CHANNEL f2 ========

CPDPRG2	waltz16	
NUC2	1H	
PCPD2	80.00	usec
PL2	2.00	dB
PL12	18.00	dB
PL13	18.00	dB
SFO2	500.0320000	MHz
SI	32768	
SF	125.7326440	MHz
WDW	EM	
SSB	0	
LB	10.00	Hz
GB	0	
PC	1.40	

(*S*,*E*)-2-(((1,3-diphenylallyl)oxy)methyl)naphthalene **(5f)**

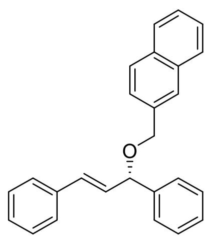

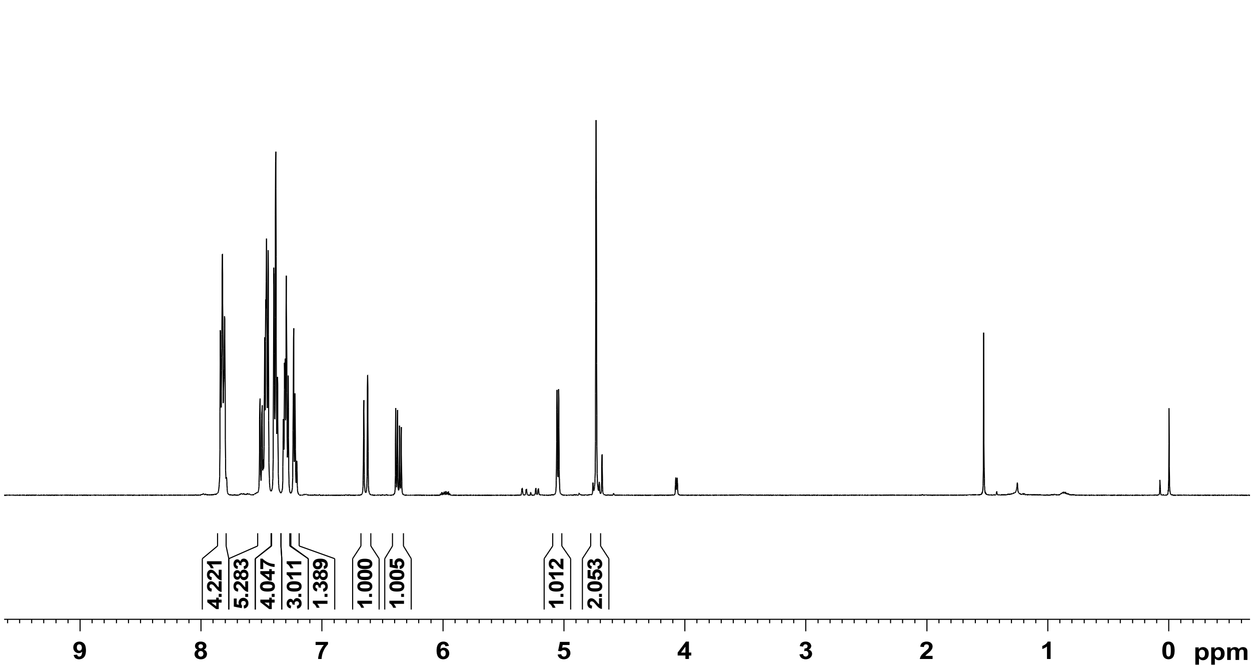


```
NAME           QZX-5-132
EXPNO                  1
PROCNO                 1
Date_           20181120
Time               15.32
INSTRUM            spect
PROBHD   5 mm PADUL 13C
PULPROG             zg30
TD                 16384
SOLVENT            CDCl3
NS                     8
DS                     1
SWH            10000.000 Hz
FIDRES          0.610352 Hz
AQ             0.8193000 sec
RG                   406
DW                50.000 usec
DE                  6.00 usec
TE                 295.7 K
D1            2.00000000 sec
TD0                    1

======== CHANNEL f1 ========
NUC1                  1H
P1                 13.00 usec
PL1                 2.00 dB
SFO1         500.0335010 MHz
SI                 16384
SF           500.0300214 MHz
WDW                   EM
SSB                    0
LB                  0.30 Hz
GB                     0
PC                  2.00
```

QZX-5-133 13C 1D 2018 11 20

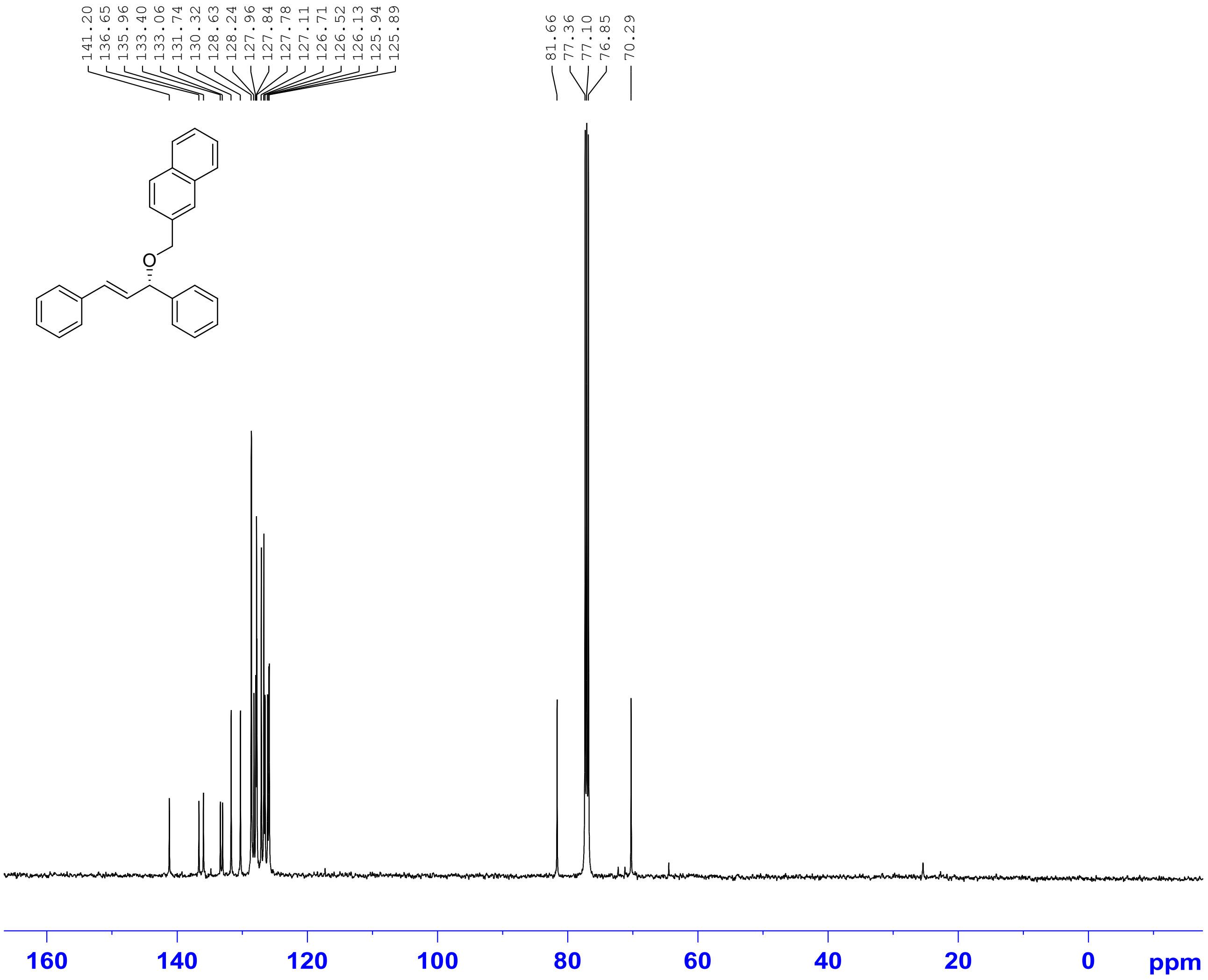

BRUKER

NAME	QZX-5-133
EXPNO	2
PROCNO	1
Date_	20181120
Time	12.06
INSTRUM	spect
PROBHD	5 mm PADUL 13C
PULPROG	zgpg30
TD	65536
SOLVENT	CDCl3
NS	668
DS	1
SWH	32679.738 Hz
FIDRES	0.498653 Hz
AQ	1.0027661 sec
RG	6500
DW	15.300 usec
DE	6.00 usec
TE	297.0 K
D1	2.00000000 sec
d11	0.03000000 sec
DELTA	1.89999998 sec
TD0	10
======== CHANNEL f1 ========	
NUC1	13C
P1	12.20 usec
PL1	4.00 dB
SFO1	125.7464750 MHz
======== CHANNEL f2 ========	
CPDPRG2	waltz16
NUC2	1H
PCPD2	80.00 usec
PL2	2.00 dB
PL12	18.00 dB
PL13	18.00 dB
SFO2	500.0355000 MHz
SI	32768
SF	125.7326395 MHz
WDW	EM
SSB	0
LB	6.00 Hz
GB	0
PC	2.00

(*S*,*E*)-2-(((1,3-diphenylallyl)oxy)methyl)pyridine **(5g)**

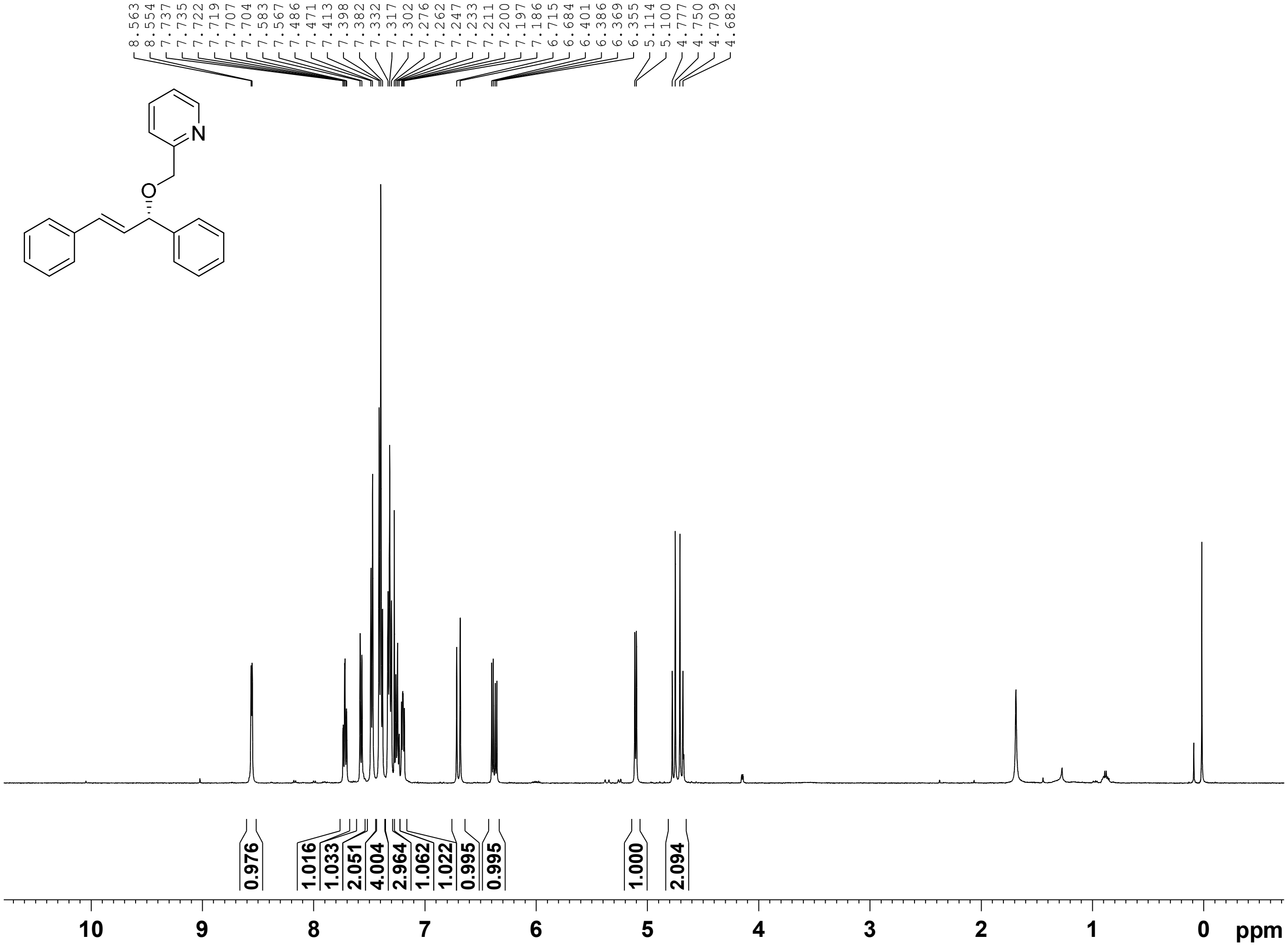


```
NAME          QZX-5-82-1
EXPNO                  1
PROCNO                 1
Date_           20181102
Time               11.31
INSTRUM            spect
PROBHD   5 mm PADUL 13C
PULPROG             zg30
TD                 16384
SOLVENT            CDCl3
NS                     8
DS                     1
SWH            10000.000 Hz
FIDRES          0.610352 Hz
AQ             0.8193000 sec
RG                   406
DW                50.000 usec
DE                  6.00 usec
TE                 294.3 K
D1            2.00000000 sec
TD0                    1

======== CHANNEL f1 ========
NUC1                  1H
P1                 13.00 usec
PL1                 2.00 dB
SFO1         500.0335010 MHz
SI                 16384
SF           500.0300016 MHz
WDW                   EM
SSB                    0
LB                  0.30 Hz
GB                     0
PC                  2.00
```

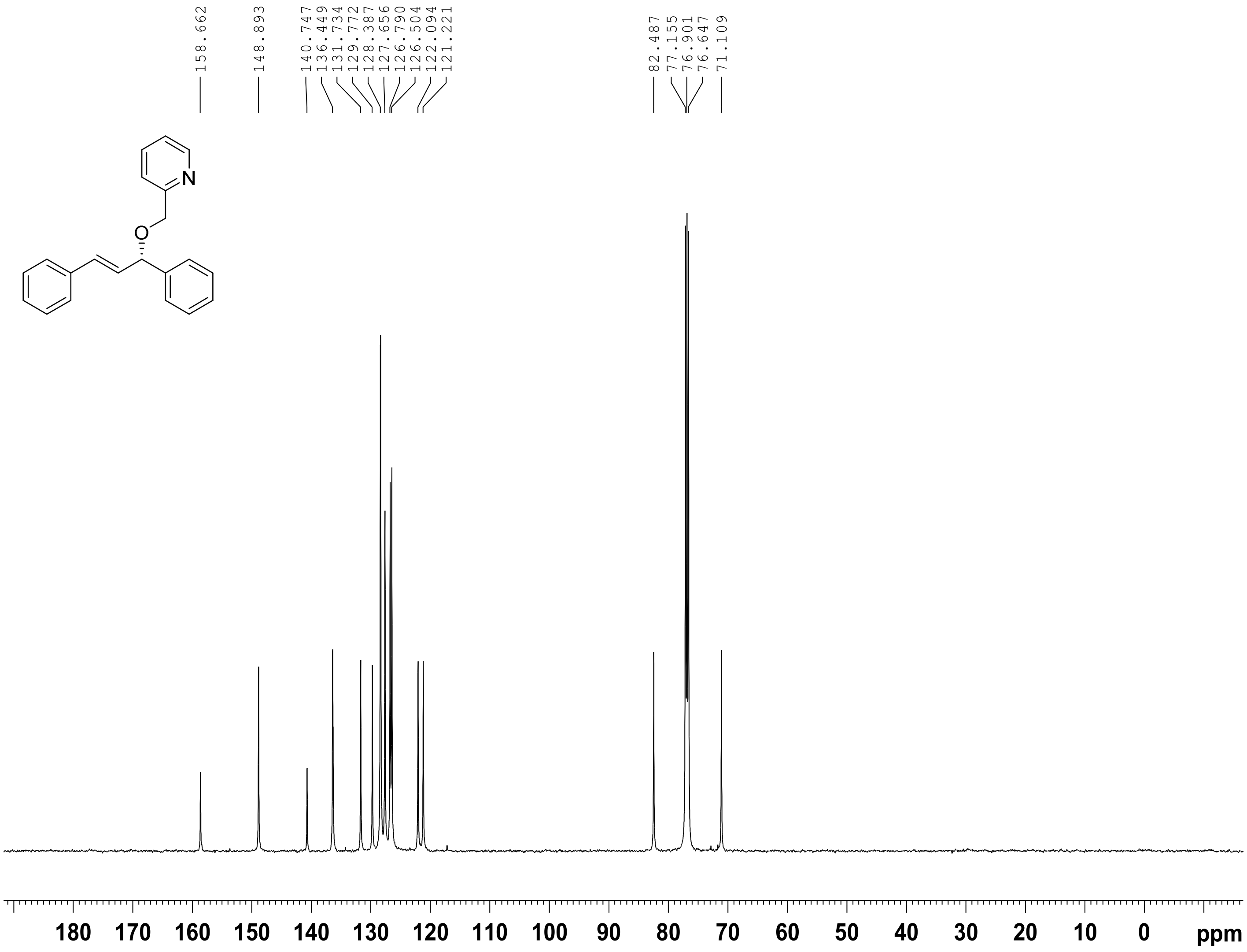


```
NAME          QZX-5-82-2
EXPNO                  2
PROCNO                 1
Date_           20181102
Time               12.26
INSTRUM            spect
PROBHD   5 mm PADUL 13C
PULPROG          zgpg30
TD                 65536
SOLVENT            CDCl3
NS                  2845
DS                     1
SWH            32679.738 Hz
FIDRES          0.498653 Hz
AQ             1.0027661 sec
RG                  4100
DW                15.300 usec
DE                  6.00 usec
TE                 297.3 K
D1            2.00000000 sec
d11           0.03000000 sec
DELTA         1.89999998 sec
TD0                   20

======== CHANNEL f1 ========
NUC1                 13C
P1                 12.20 usec
PL1                 3.00 dB
SFO1         125.7464750 MHz

======== CHANNEL f2 ========
CPDPRG2          waltz16
NUC2                  1H
PCPD2              80.00 usec
PL2                 2.00 dB
PL12               17.70 dB
PL13               17.70 dB
SFO2         500.0355000 MHz
SI                 32768
SF           125.7326647 MHz
WDW                   EM
SSB                    0
LB                 10.00 Hz
GB                     0
PC                  1.00
```

(*S*,*E*)-(3-ethoxyprop-1-ene-1,3-diyl)dibenzene **(5h)**

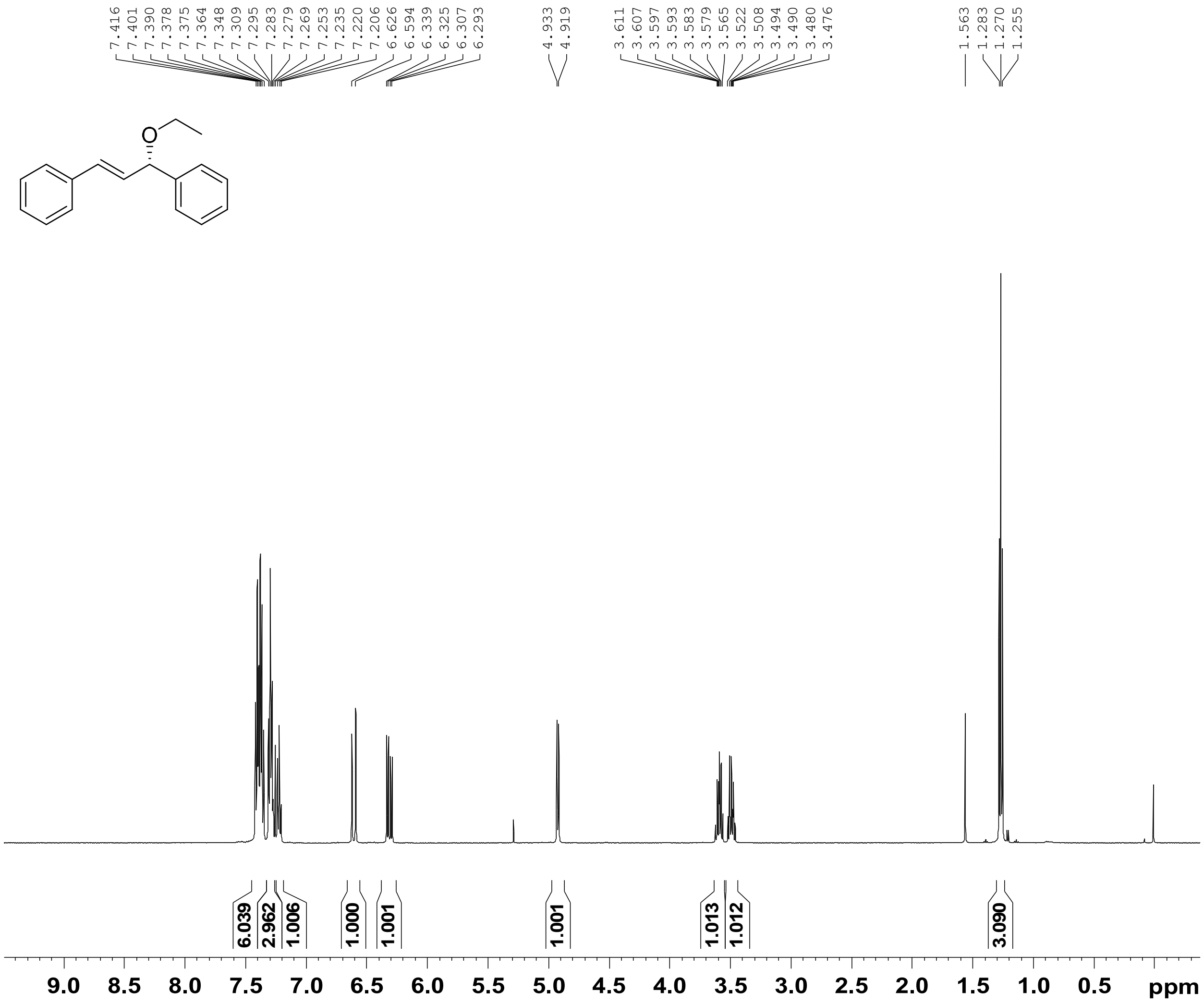

NAME	QZX-5-112-1
EXPNO	1
PROCNO	1
Date_	20181112
Time	14.13
INSTRUM	spect
PROBHD	5 mm PADUL 13C
PULPROG	zg30
TD	16384
SOLVENT	CDCl3
NS	8
DS	0
SWH	10000.000
FIDRES	0.610352
AQ	0.8193000
RG	256
DW	50.000
DE	8.00
TE	296.1
D1	2.00000000
TD0	1
======== CHANNEL f1 ===	
NUC1	1H
P1	13.00
PL1	2.00
SFO1	500.0335000
SI	16384
SF	500.0300132
WDW	EM
SSB	0
LB	0.30
GB	0
PC	1.00

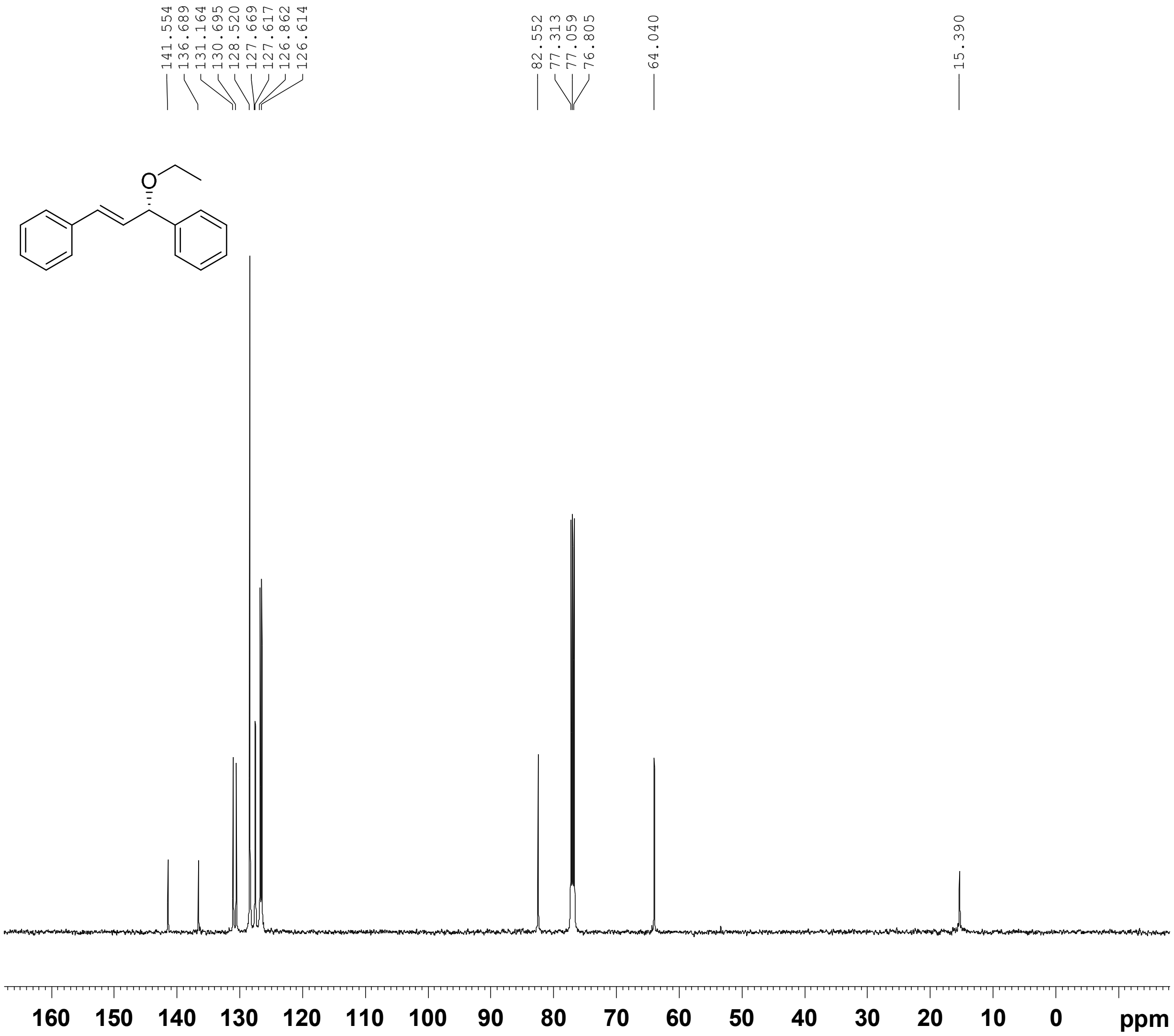

NAME	QZX-5-112-2
EXPNO	2
PROCNO	1
Date_	20181112
Time	13.22
INSTRUM	spect
PROBHD	5 mm PADUL 13C
PULPROG	zgpg30
TD	65536
SOLVENT	CDCl3
NS	300
DS	1
SWH	32679.738
FIDRES	0.498653
AQ	1.0027661
RG	8200
DW	15.300
DE	6.00
TE	297.6
D1	2.00000000
d11	0.03000000
DELTA	1.89999998
TD0	10

======== CHANNEL f1 ===

NUC1	13C
P1	12.20
PL1	3.00
SFO1	125.7464750

======== CHANNEL f2 ===

CPDPRG2	waltz16
NUC2	1H
PCPD2	80.00
PL2	2.00
PL12	17.70

(*S*,*E*)-(3-(but-3-en-1-yloxy)prop-1-ene-1,3-diyl)dibenzene **(5i)**

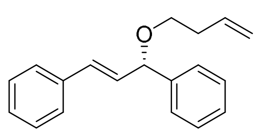

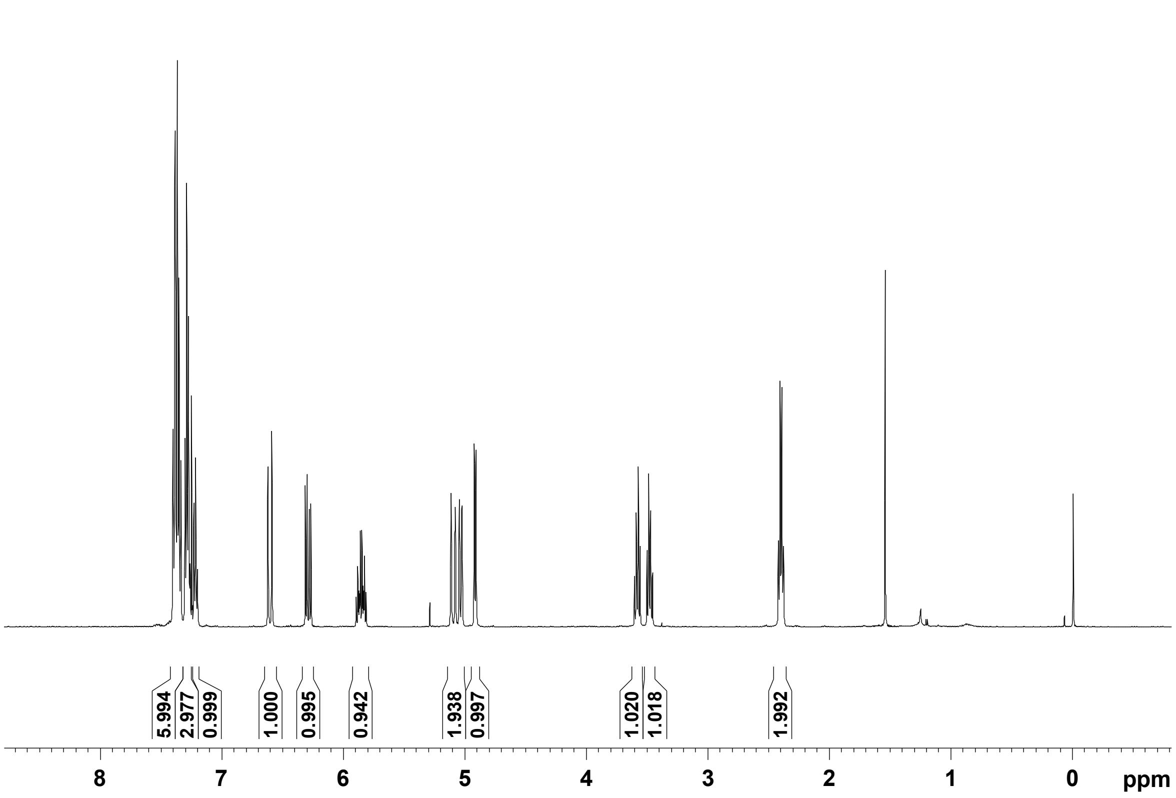


```
NAME        QZX-5-114-1
EXPNO                 1
PROCNO                1
Date_          20181112
Time              14.20
INSTRUM           spect
PROBHD   5 mm PADUL 13C
PULPROG            zg30
TD                16384
SOLVENT           CDCl3
NS                    8
DS                    0
SWH          10000.000 Hz
FIDRES        0.610352 Hz
AQ           0.8193000 sec
RG                  256
DW               50.000 usec
DE                 8.00 usec
TE                296.1 K
D1           2.00000000 sec
TD0                   1

======== CHANNEL f1 ========
NUC1                 1H
P1                13.00 usec
PL1                2.00 dB
SFO1        500.0335000 MHz
SI                16384
SF          500.0300153 MHz
WDW                  EM
SSB                   0
LB                 0.30 Hz
GB                    0
PC                 1.00
```

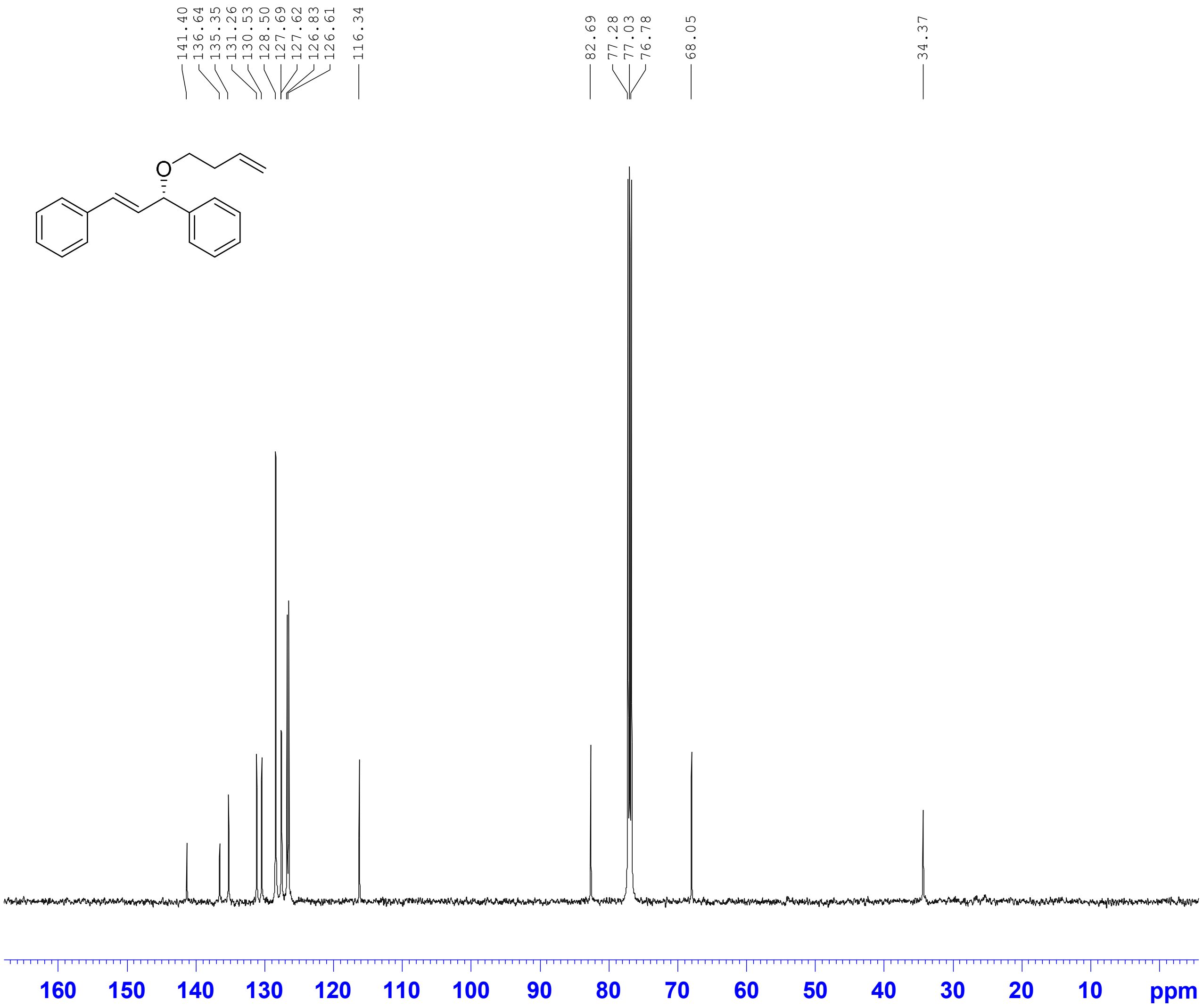

NAME	QZX-5-114
EXPNO	
PROCNO	
Date_	201811
Time	13.
INSTRUM	spe
PROBHD	5 mm PADUL 1
PULPROG	zgpg
TD	655
SOLVENT	CDC
NS	4
DS	
SWH	32679.7
FIDRES	0.4986
AQ	1.00276
RG	82
DW	15.3
DE	6.
TE	298
D1	2.000000
d11	0.030000
DELTA	1.899999
TD0	

======== CHANNEL f1 =

NUC1	1
P1	12.
PL1	3.
SFO1	125.74647

======== CHANNEL f2 =

CPDPRG2	waltz
NUC2	
PCPD2	80.
PL2	2.
PL12	17.
PL13	17.
SFO2	500.03550
SI	327
SF	125.73264
WDW	
SSB	

(*S*,*E*)-2-((1,3-diphenylallyl)oxy)-2,3-dihydro-1H-indene **(5j)**

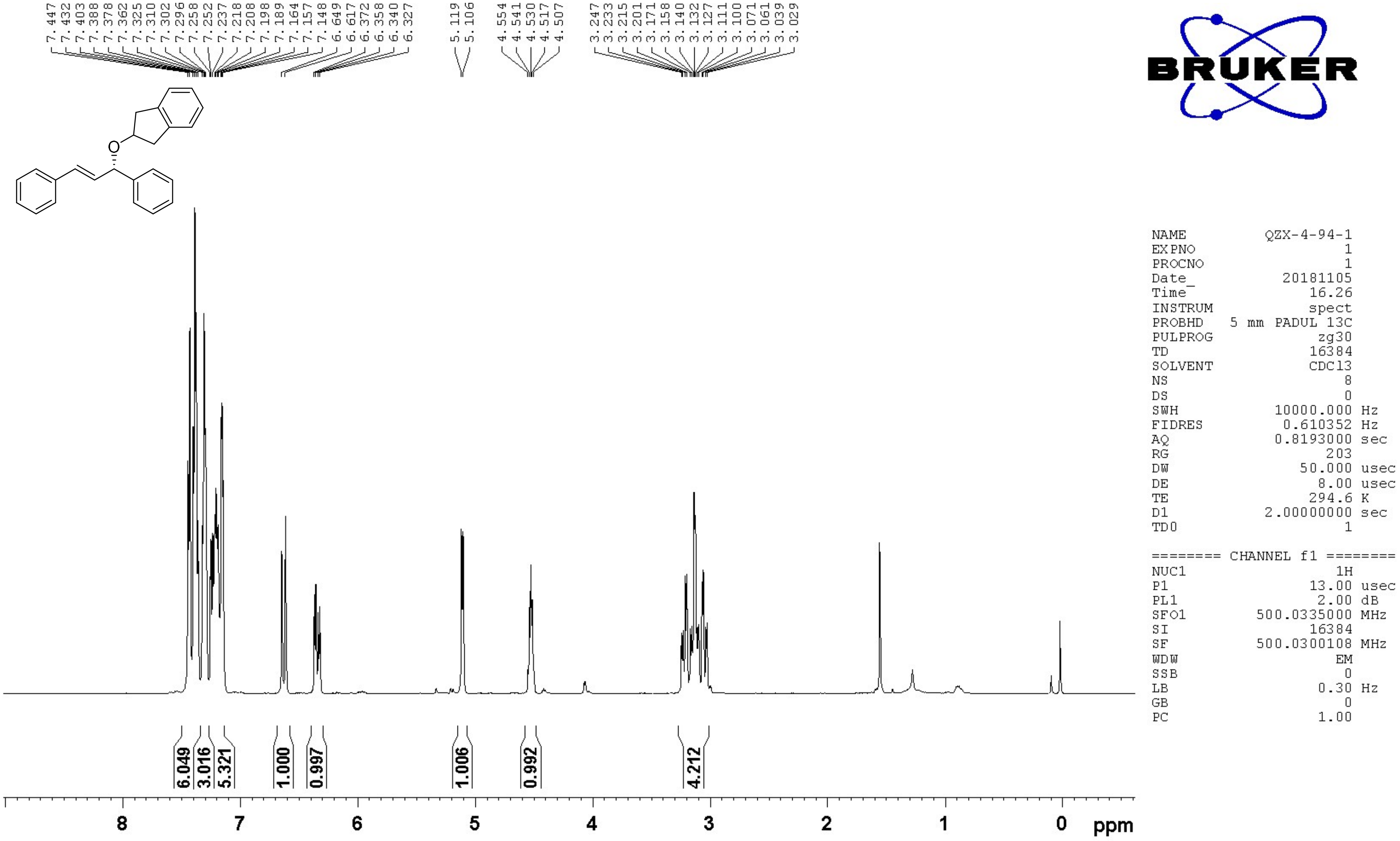

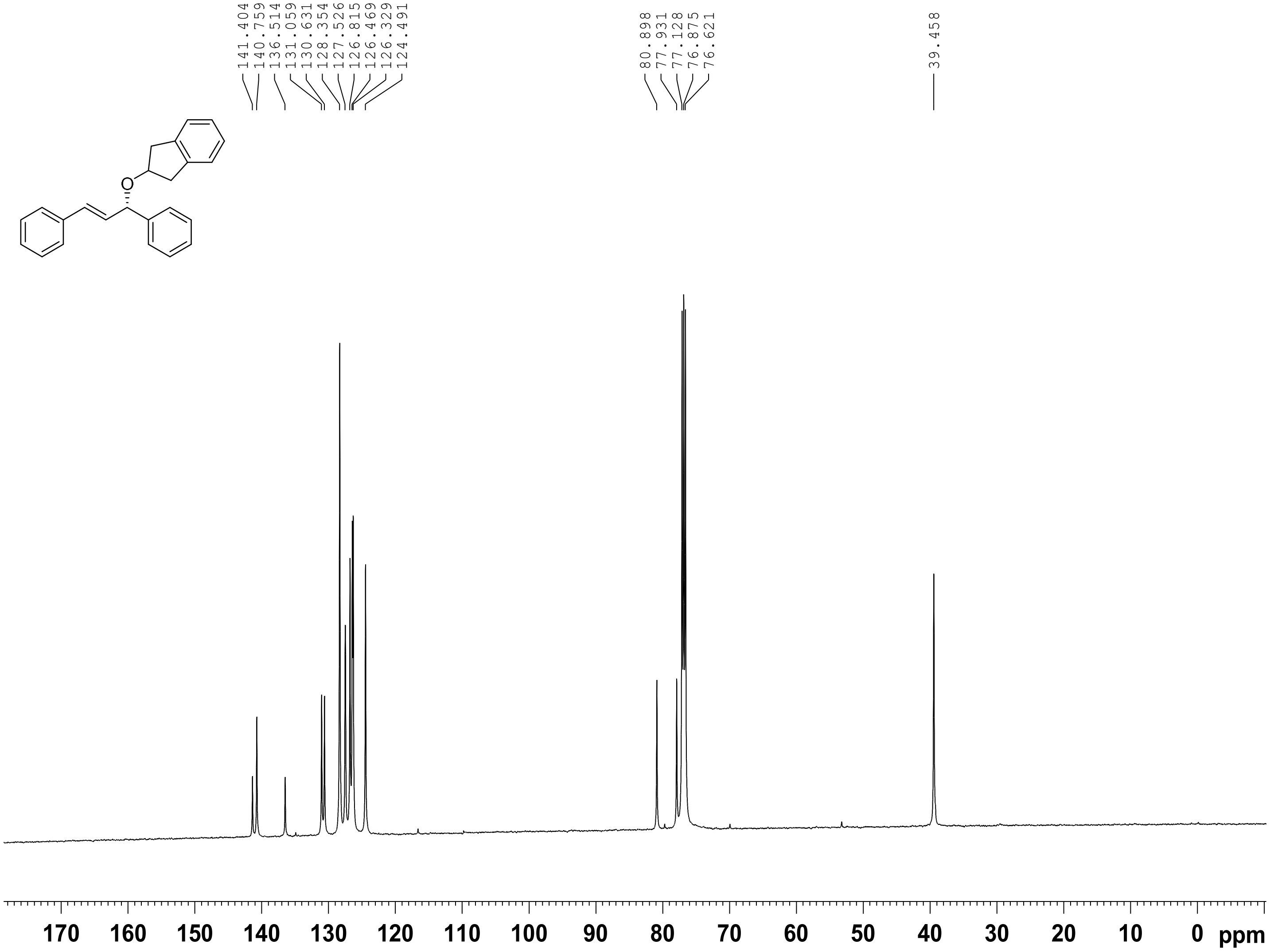

NAME	QZX-5-94-2
EXPNO	2
PROCNO	1
Date_	20181105
Time	22.01
INSTRUM	spect
PROBHD	5 mm PADUL 13C
PULPROG	zgpg30
TD	65536
SOLVENT	CDCl3
NS	12613
DS	1
SWH	32679.738 Hz
FIDRES	0.498653 Hz
AQ	1.0027661 sec
RG	2050
DW	15.300 usec
DE	6.00 usec
TE	299.9 K
D1	2.00000000 sec
d11	0.03000000 sec
DELTA	1.89999998 sec
TD0	20

======== CHANNEL f1 ========

NUC1	13C
P1	12.20 usec
PL1	4.00 dB
SFO1	125.7464750 MHz

======== CHANNEL f2 ========

CPDPRG2	waltz16
NUC2	1H
PCPD2	80.00 usec
PL2	2.00 dB
PL12	18.00 dB
PL13	18.00 dB
SFO2	500.0355000 MHz
SI	32768
SF	125.7326639 MHz
WDW	EM
SSB	0
LB	10.00 Hz
GB	0
PC	1.00

Copies of HPLC traces

(*S,E*)-*tert*-butyl 3-(1,3-diphenylallyl)-1H-indole-1-carboxylate **(Boc-3a)**

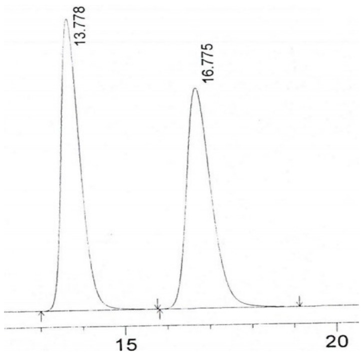

Peak	Ret. Time	Area	Area %	Height	Height %
1	13.778	4250591	50.013	139794	57.013
2	16.775	4248406	49.987	105401	42.987

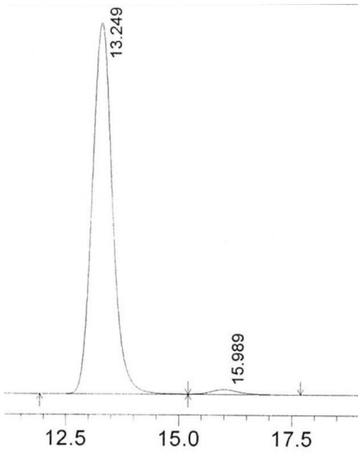

Peak	Ret. Time	Area	Area %	Height	Height %
1	13.249	21205207	98.074	679904	98.659
2	15.989	416506	1.926	9238	1.341

(S,E)-tert-butyl 3-(1,3-diphenylallyl)-2-methyl-1H-indole-1-carboxylate **(Boc-3b)**

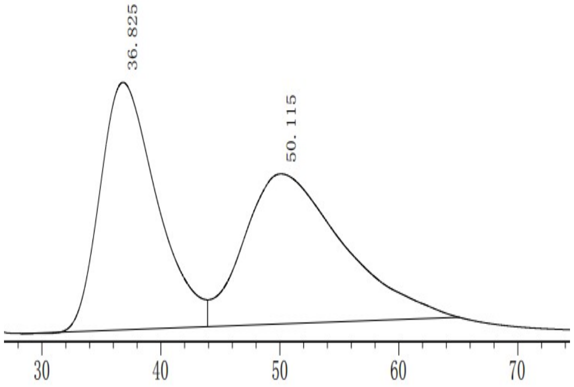

Peak	Ret. Time	Area	Area %	Height	Height %
1	36.825	329364306	48.528	990281	62.255
2	50.115	349345117	51.472	600406	37.745

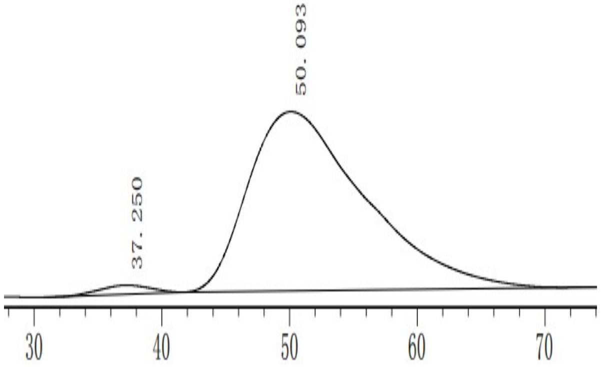

Peak	Ret. Time	Area	Area %	Height	Height %
1	37.250	7230666	2.121	26236	4.782
2	50.093	333726253	97.879	522399	95.218

(S,E)-tert-butyl 3-(1,3-diphenylallyl)-2-phenyl-1H-indole-1-carboxylate **(Boc-3c)**

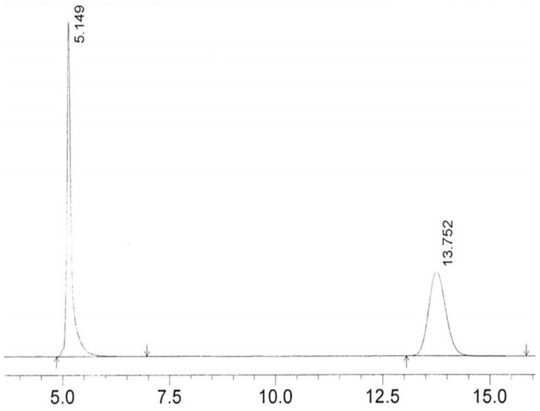

Peak	Ret. Time	Area	Area %	Height	Height %
1	5.149	5320216	50.946	739199	79.969
2	13.752	5122640	49.054	185163	20.031

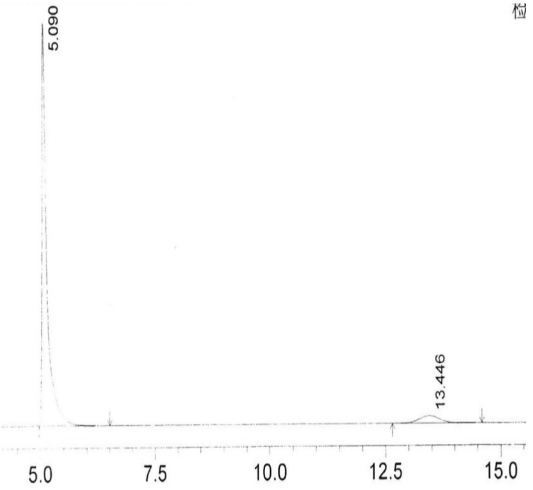

Peak	Ret. Time	Area	Area %	Height	Height %
1	5.090	5551490	92.948	756768	98.227
2	13.446	421177	7.052	13656	1.773

(S,E)-tert-butyl 3-(1,3-diphenylallyl)-4-methyl-1H-indole-1-carboxylate **(Boc-3d)**

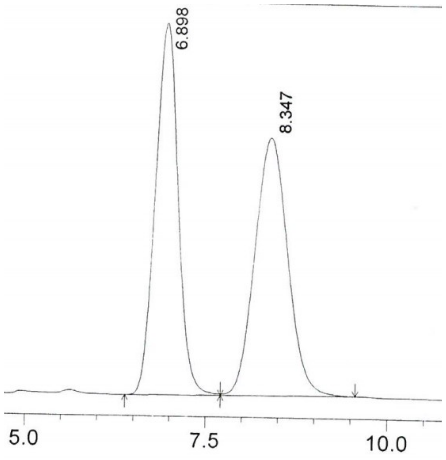

Peak	Ret. Time	Area	Area %	Height	Height %
1	6.898	7004288	50.085	319016	59.047
2	8.347	6980641	49.915	221261	40.953

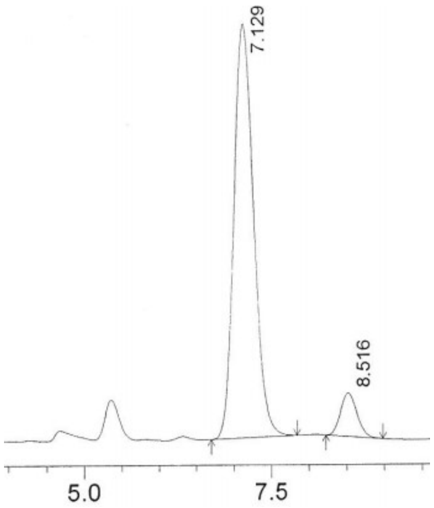

Peak	Ret. Time	Area	Area %	Height	Height %
1	7.129	1463468	91.970	80010	90.512
2	8.516	127782	8.030	8387	9.488

(S,E)-tert-butyl 3-(1,3-diphenylallyl)-4-methoxy-1H-indole-1-carboxylate **(Boc-3e)**

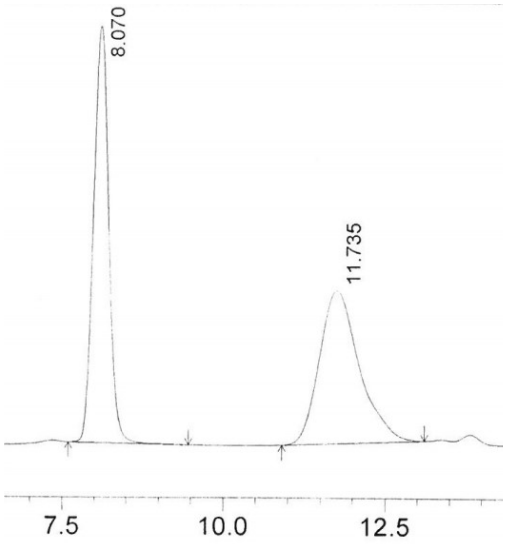

Peak	Ret. Time	Area	Area %	Height	Height %
1	8.070	965945	50.784	59128	73.051
2	11.735	936112	49.216	21812	26.949

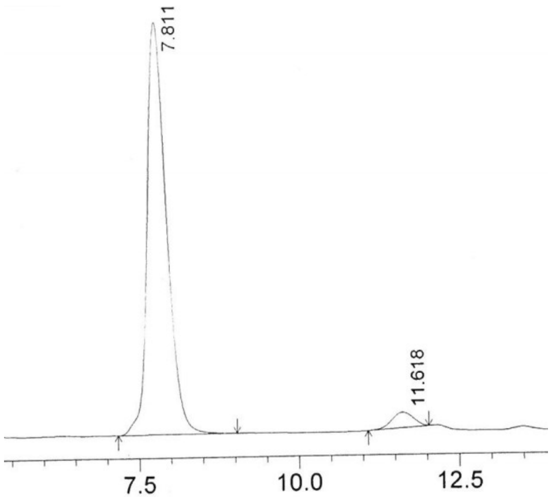

Peak	Ret. Time	Area	Area %	Height	Height %
1	7.811	4215430	96.013	199195	96.382
2	11.618	175031	3.987	7478	3.618

(S,E)-tert-butyl 3-(1,3-diphenylallyl)-5-methyl-1H-indole-1-carboxylate **(Boc-3f)**

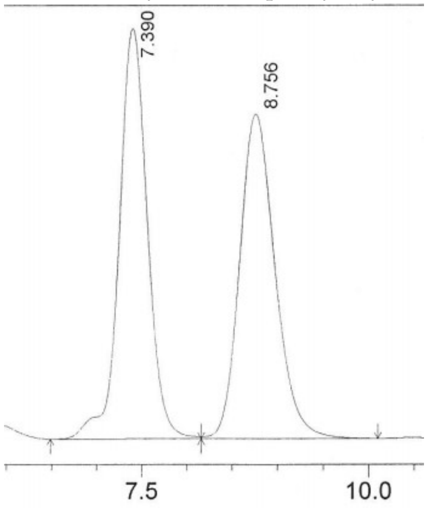

Peak	Ret. Time	Area	Area %	Height	Height %
1	7.390	2437609	50.506	112059	55.866
2	8.756	2388786	49.494	88526	44.134

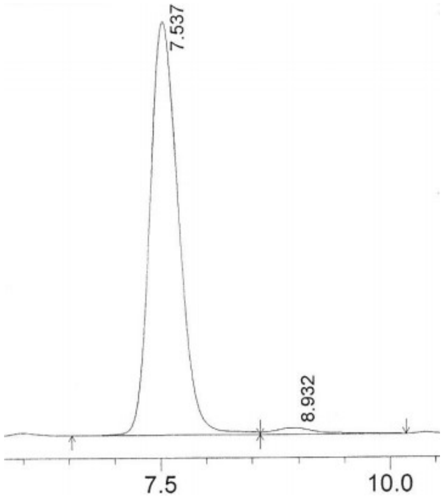

Peak	Ret. Time	Area	Area %	Height	Height %
1	7.537	4395346	97.646	201772	98.428
2	8.932	105949	2.354	3222	1.572

(S,E)-tert-butyl 3-(1,3-diphenylallyl)-5-methoxy-1H-indole-1-carboxylate **(Boc-3g)**

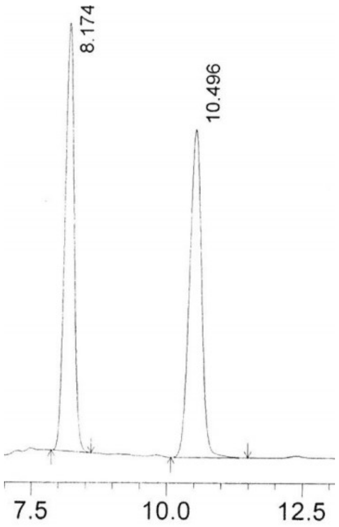

Peak	Ret. Time	Area	Area %	Height	Height %
1	8.174	499655	49.585	43036	56.643
2	10.496	508014	50.415	32942	43.357

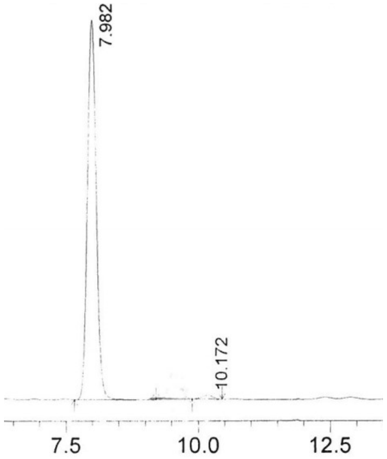

Peak	Ret. Time	Area	Area %	Height	Height %
1	7.982	3812142	98.762	330367	99.007
2	10.172	47805	1.238	3314	0.993

(S,E)-tert-butyl 5-(benzyloxy)-3-(1,3-diphenylallyl)-1H-indole-1-carboxylate **(Boc-3h)**

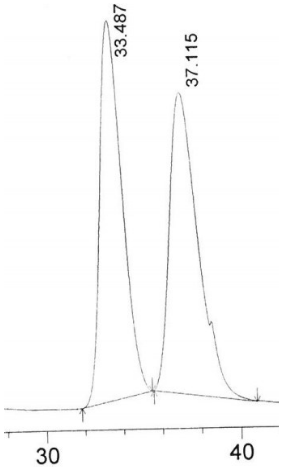

Peak	Ret. Time	Area	Area %	Height	Height %
1	33.487	4041463	49.717	50931	55.784
2	37.115	4087427	50.283	40369	44.216

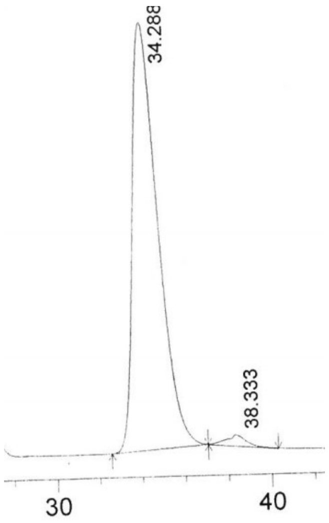

Peak	Ret. Time	Area	Area %	Height	Height %
1	34.288	11710212	97.859	143917	97.369
2	38.333	256153	2.141	3889	2.631

(S,E)-tert-butyl 5-chloro-3-(1,3-diphenylallyl)-1H-indole-1-carboxylate **(Boc-3i)**

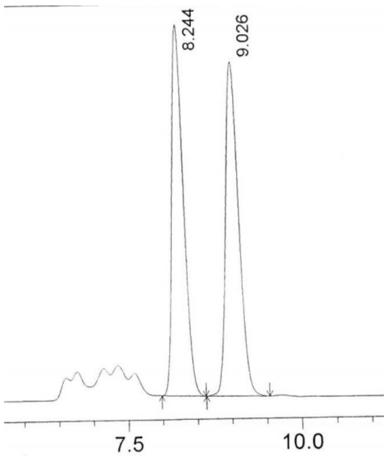

Peak	Ret. Time	Area	Area %	Height	Height %
1	8.244	3545510	49.613	310887	52.628
2	9.026	3600754	50.387	279842	47.372

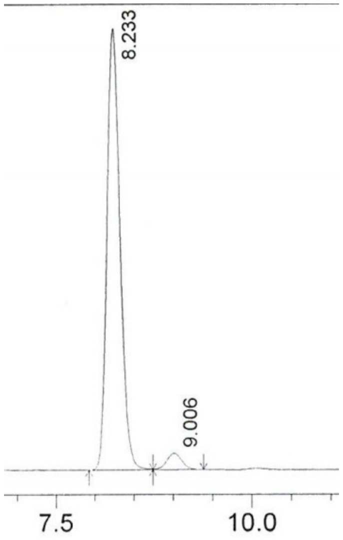

Peak	Ret. Time	Area	Area %	Height	Height %
1	8.233	5776688	95.919	499490	96.420
2	9.006	245749	4.081	18546	3.580

(S,E)-tert-butyl 5-bromo-3-(1,3-diphenylallyl)-1H-indole-1-carboxylate **(Boc-3j)**

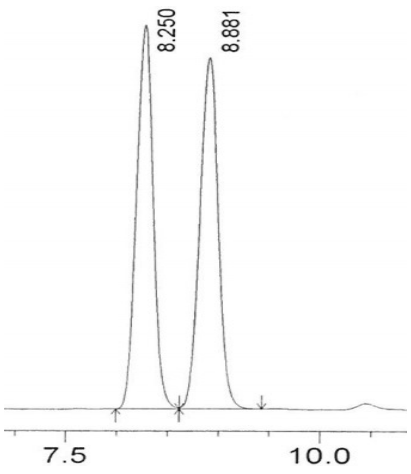

Peak	Ret. Time	Area	Area %	Height	Height %
1	8.250	16516382	49.858	1466632	52.196
2	8.881	16610272	50.142	1343201	47.804

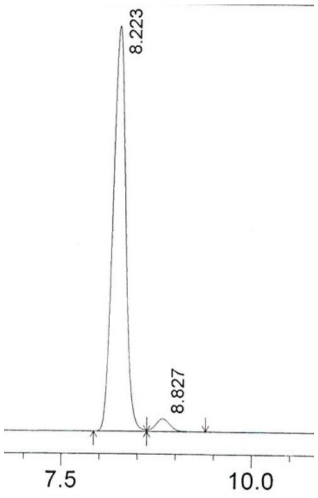

Peak	Ret. Time	Area	Area %	Height	Height %
1	8.223	17519449	96.597	1533033	96.950
2	8.827	617248	3.403	48224	3.050

(S,E)-tert-butyl 3-(1,3-diphenylallyl)-6-methyl-1H-indole-1-carboxylate **(Boc-3k)**

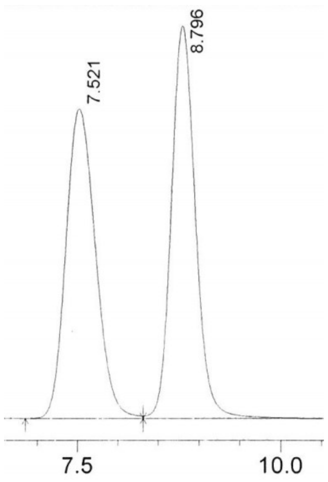

Peak	Ret. Time	Area	Area %	Height	Height %
1	7.521	3893077	49.525	153328	44.088
2	8.796	3967787	50.475	194451	55.912

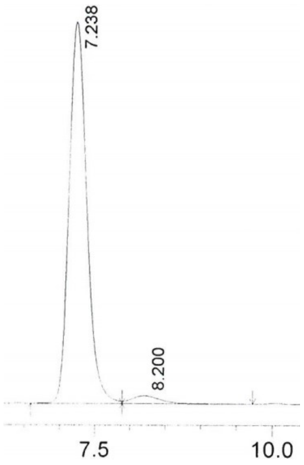

Peak	Ret. Time	Area	Area %	Height	Height %
1	7.238	508344	96.686	291960	97.954
2	8.200	174263	3.314	6098	2.046

(S,E)-tert-butyl 6-chloro-3-(1,3-diphenylallyl)-1H-indole-1-carboxylate **(Boc-3l)**

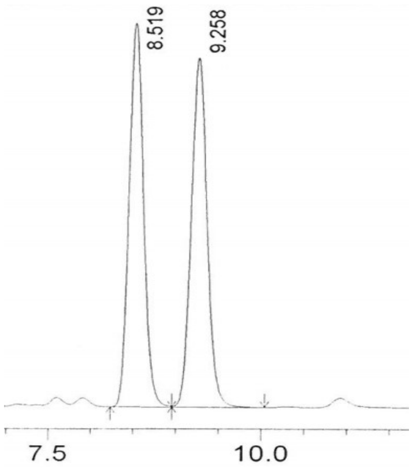

Peak	Ret. Time	Area	Area %	Height	Height %
1	8.519	3981849	49.849	338253	52.394
2	9.258	4005980	50.151	307343	47.606

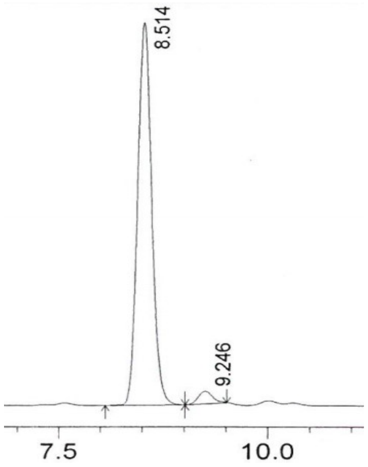

Peak	Ret. Time	Area	Area %	Height	Height %
1	8.514	5040852	96.650	432094	96.772
2	9.246	174734	3.350	14413	3.228

(S,E)-tert-butyl 3-(1,3-diphenylallyl)-7-methyl-1H-indole-1-carboxylate **(Boc-3m)**

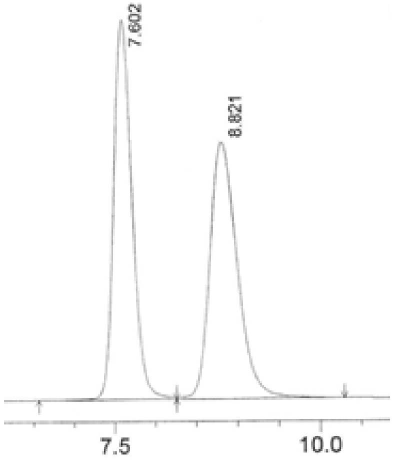

Peak	Ret. Time	Area	Area %	Height	Height %
1	7.602	2135453	50.282	136080	59.733
2	8.821	2111510	49.718	91732	40.267

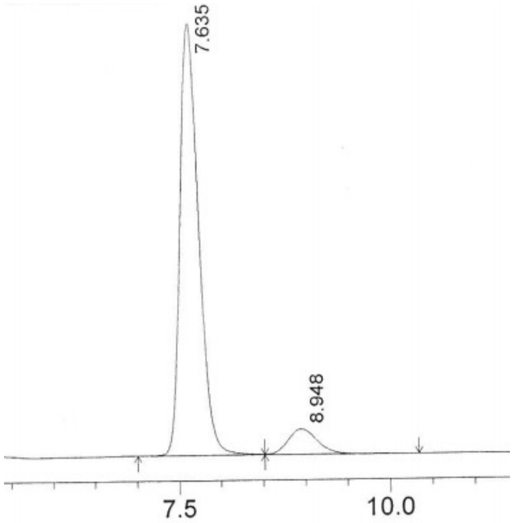

Peak	Ret. Time	Area	Area %	Height	Height %
1	7.635	3842017	91.440	247842	94.445
2	8.948	359657	8.560	14578	5.555

(S,E)-tert-butyl 3-(1,3-diphenylallyl)-7-methoxy-1H-indole-1-carboxylate (**Boc-3n**)

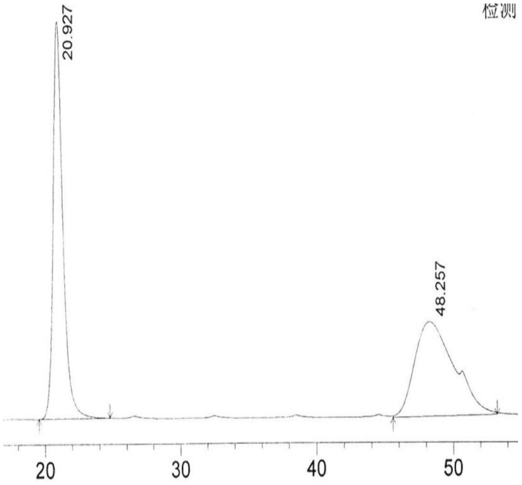

Peak	Ret. Time	Area	Area %	Height	Height %
1	20.927	4802293	50.865	96032	80.715
2	48.257	4639052	49.135	22945	19.285

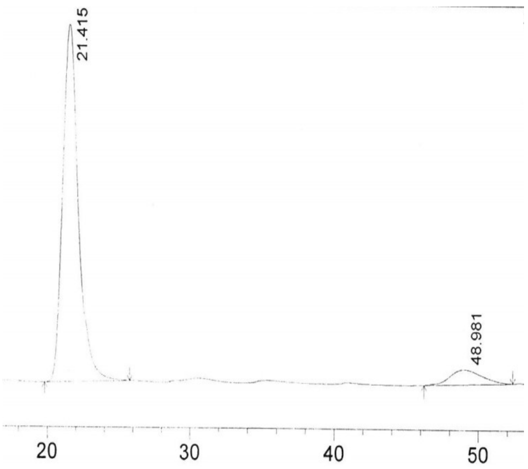

Peak	Ret. Time	Area	Area %	Height	Height %
1	21.415	6433551	92.057	84877	95.947
2	48.981	555095	7.943	3585	4.053

(S,E)-tert-butyl 7-chloro-3-(1,3-diphenylallyl)-1H-indole-1-carboxylate **(Boc-3o)**

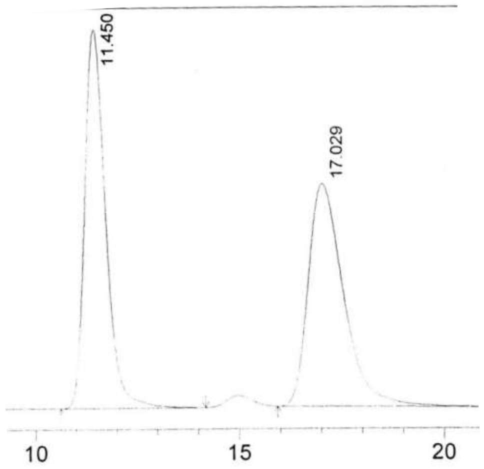

Peak	Ret. Time	Area	Area %	Height	Height %
1	11.450	8703837	50.225	244188	62.926
2	17.029	8625914	49.775	143865	37.074

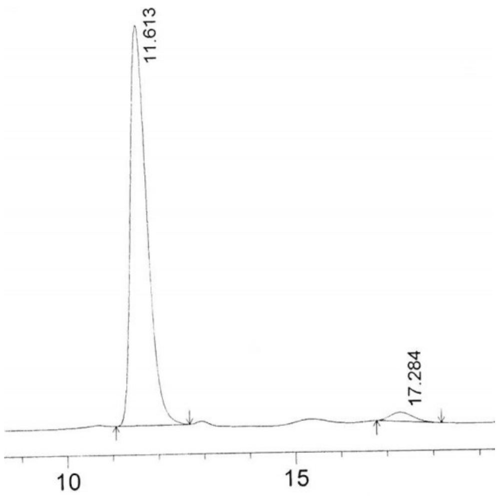

Peak	Ret. Time	Area	Area %	Height	Height %
1	11.613	1688124	96.943	66968	97.778
2	17.284	53242	3.057	1522	2.222

(S,E)-tert-butyl 3-(1,3-di-p-tolylallyl)-1H-indole-1-carboxylate **(Boc-3p)**

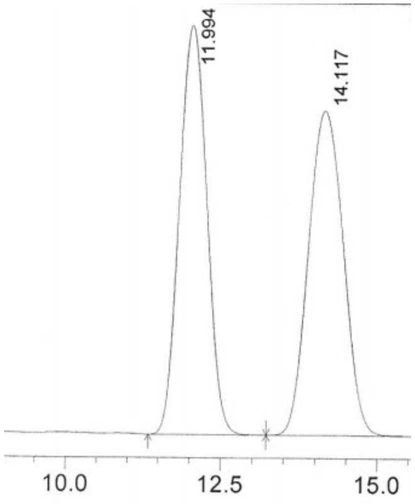

Peak	Ret. Time	Area	Area %	Height	Height %
1	11.994	11718021	49.997	382010	55.781
2	14.117	11719430	50.003	302830	44.219

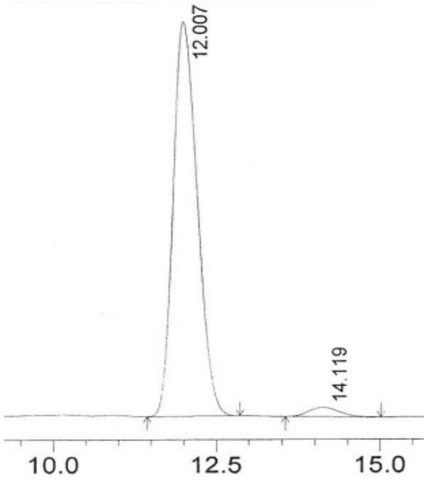

Peak	Ret. Time	Area	Area %	Height	Height %
1	12.007	5051359	97.172	195196	97.697
2	14.119	147018	2.828	4602	2.303

(*S,E*)-*tert*-butyl 3-(1,3-bis(4-methoxyphenyl)allyl)-1H-indole-1-carboxylate **(Boc-3q)**

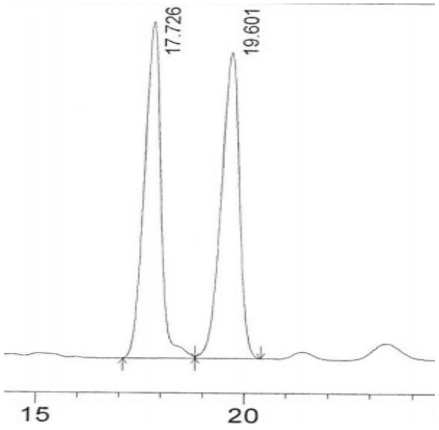

Peak	Ret. Time	Area	Area %	Height	Height %
1	17.726	90379	50.266	3247	52.349
2	19.601	89422	49.734	2955	47.651

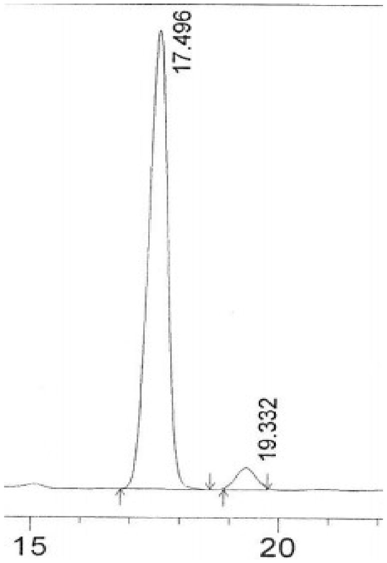

Peak	Ret. Time	Area	Area %	Height	Height %
1	17.496	532874	95.084	20045	95.435
2	19.332	27548	4.916	959	4.565

(S,E)-tert-butyl 3-(1,3-bis(4-chlorophenyl)allyl)-1H-indole-1-carboxylate **(Boc-3r)**

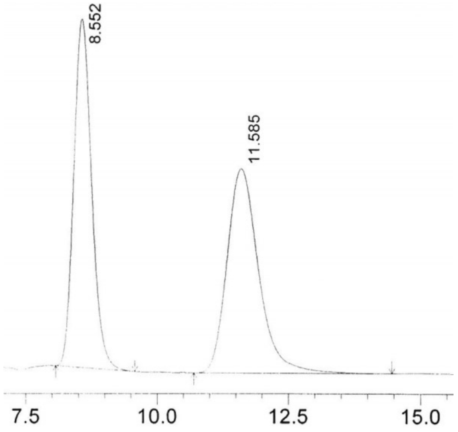

Peak	Ret. Time	Area	Area %	Height	Height %
1	8.552	4443538	49.069	186696	63.038
2	11.585	4612086	50.931	109468	36.962

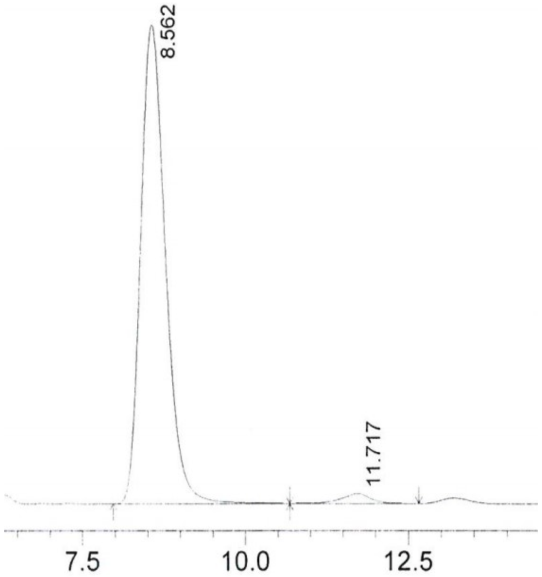

Peak	Ret. Time	Area	Area %	Height	Height %
1	8.562	11518499	97.517	435315	97.973
2	11.717	293328	2.483	9007	2.027

(*S*,*E*)-*tert*-butyl 3-(1,3-bis(4-nitrophenyl)allyl)-1H-indole-1-carboxylate **(Boc-3s)**

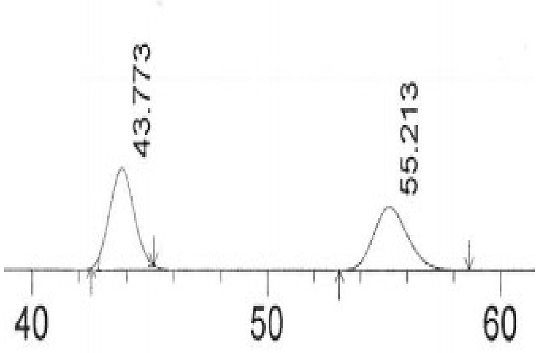

Peak	Ret. Time	Area	Area %	Height	Height %
1	43.773	2146291	50.445	31472	60.401
2	55.213	2108414	49.555	20633	39.599

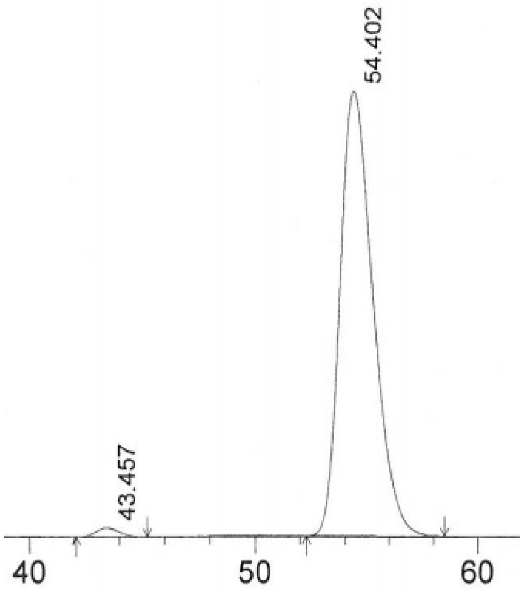

Peak	Ret. Time	Area	Area %	Height	Height %
1	43.457	137173	1.440	1918	2.068
2	54.402	9386617	98.560	90856	97.932

(*S,E*)-(3-(benzyloxy)prop-1-ene-1,3-diyl)dibenzene **(5a)**

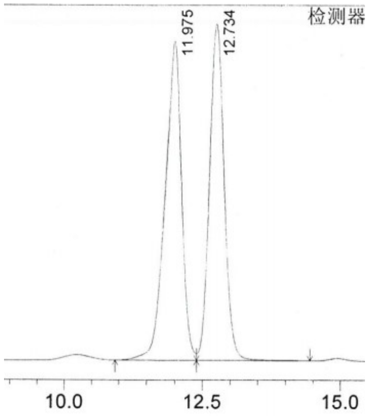

Peak	Ret. Time	Area	Area %	Height	Height %
1	11.975	6416557	50.948	315871	48.670
2	12.734	6177851	49.052	333130	51.330

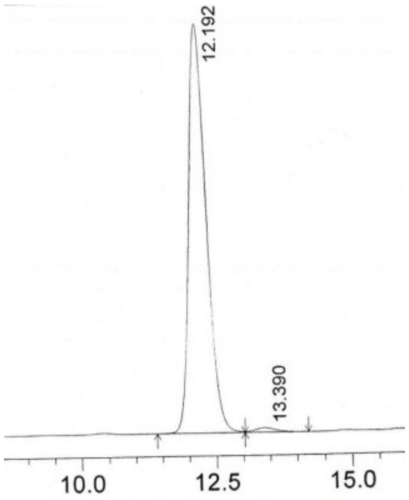

Peak	Ret. Time	Area	Area %	Height	Height %
1	12.192	658197	98.732	525427	98.947
2	13.390	151040	1.268	5589	1.053

(*S,E*)-(3-((4-methoxybenzyl)oxy)prop-1-ene-1,3-diyl)dibenzene **(5b)**

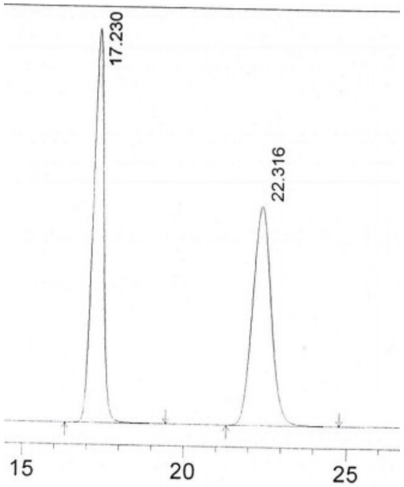

Peak	Ret. Time	Area	Area %	Height	Height %
1	17.230	5667242	50.295	252607	64.298
2	22.316	5600754	49.705	140260	35.702

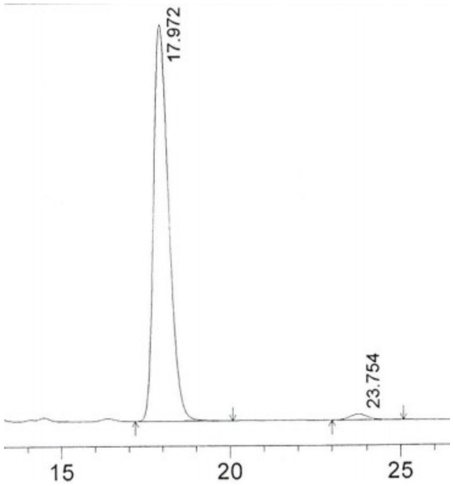

Peak	Ret. Time	Area	Area %	Height	Height %
1	17.972	10708555	98.329	342942	98.550
2	23.754	181974	1.671	5045	1.450

(*S*,*E*)-(3-((4-bromobenzyl)oxy)prop-1-ene-1,3-diyl)dibenzene **(5c)**

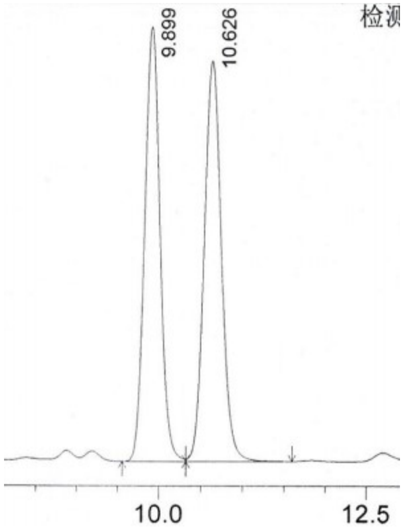

Peak	Ret. Time	Area	Area %	Height	Height %
1	9.899	8886295	49.727	679426	52.045
2	10.626	8983806	50.273	626038	47.955

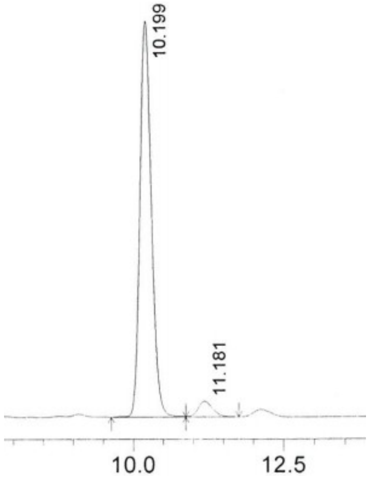

Peak	Ret. Time	Area	Area %	Height	Height %
1	10.199	3391587	95.324	252047	96.201
2	11.181	166370	4.676	9954	3.799

(*S*,*E*)-(3-((3-bromobenzyl)oxy)prop-1-ene-1,3-diyl)dibenzene **(5d)**

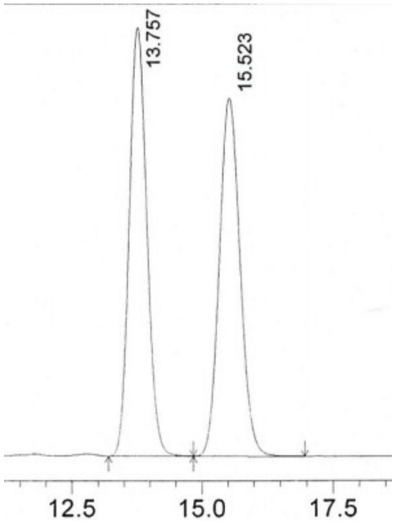

Peak	Ret. Time	Area	Area %	Height	Height %
1	13.757	3070496	50.252	130963	54.504
2	15.523	3039662	49.748	109320	45.496

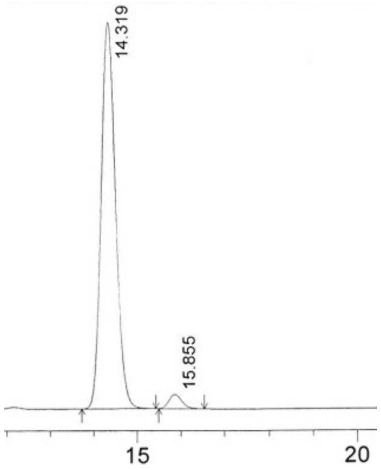

Peak	Ret. Time	Area	Area %	Height	Height %
1	14.319	21896961	96.823	944200	96.461
2	15.855	718547	3.177	34641	3.539

(*S*,*E*)-(3-((2-bromobenzyl)oxy)prop-1-ene-1,3-diyl)dibenzene **(5e)**

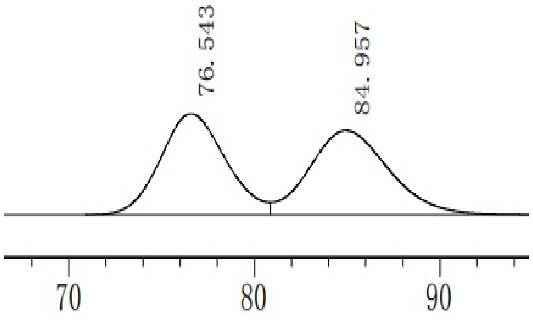

Peak	Ret. Time	Area	Area %	Height	Height %
1	76.543	28036962	49.527	114691	54571
2	84.957	28576969	50.473	95477	45429

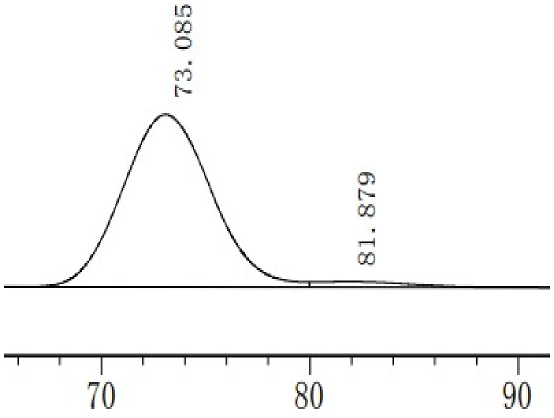

Peak	Ret. Time	Area	Area %	Height	Height %
1	73.085	36549174	96.522	121904	96.785
2	81.879	1317145	3.478	4049	3.215

(*S,E*)-2-(((1,3-diphenylallyl)oxy)methyl)naphthalene **(5f)**

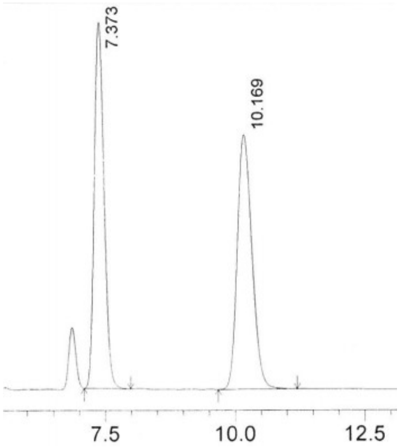

Peak	Ret. Time	Area	Area %	Height	Height %
1	7.373	3964171	48.883	300525	58.990
2	10.169	4145322	51.6	208921	41.010

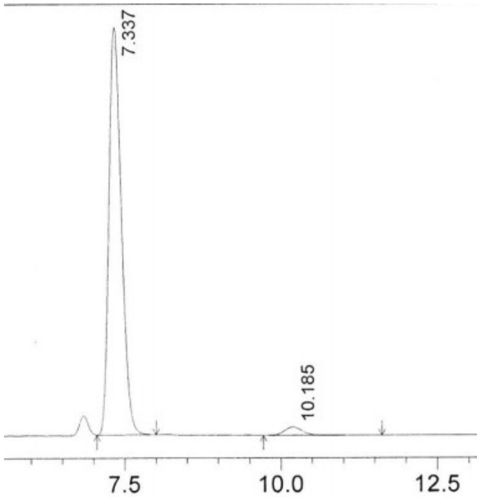

Peak	Ret. Time	Area	Area %	Height	Height %
1	7.337	20240838	97.161	1454739	98.007
2	10.185	591461	2.839	29590	1.993

(*S,E*)-2-(((1,3-diphenylallyl)oxy)methyl)pyridine **(5g)**

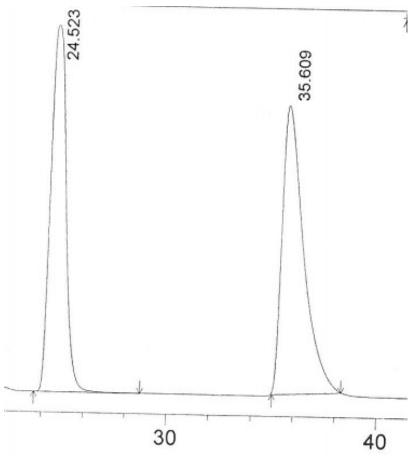

Peak	Ret. Time	Area	Area %	Height	Height %
1	24.523	150263716	47.804	2932793	55.968
2	35.609	164069148	52.196	2307289	44.032

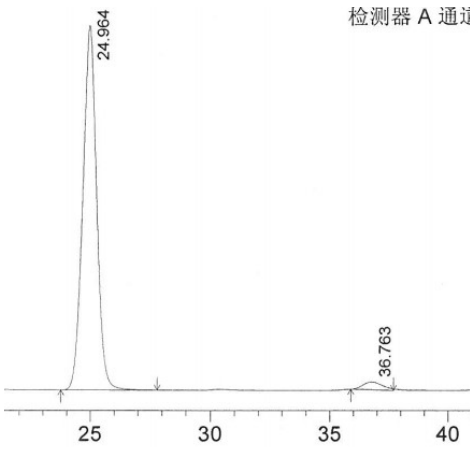

Peak	Ret. Time	Area	Area %	Height	Height %
1	24.964	6813133	96.989	168183	97.875
2	36.763	211507	3.011	3652	2.125

(*S*,*E*)-(3-ethoxyprop-1-ene-1,3-diyl)dibenzene **(5h)**

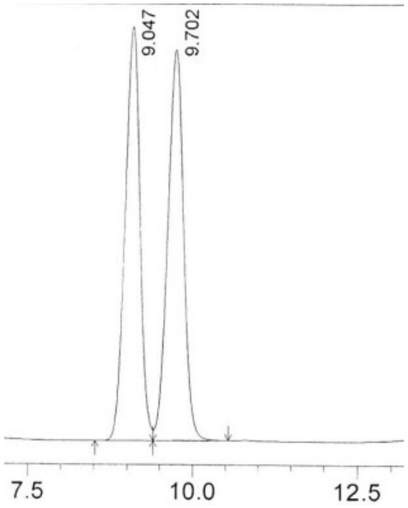

Peak	Ret. Time	Area	Area %	Height	Height %
1	9.047	2938211	49.762	186671	51.408
2	9.702	2966270	50.238	176442	48.592

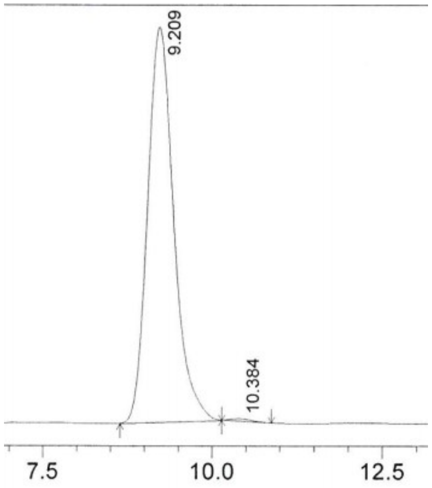

Peak	Ret. Time	Area	Area %	Height	Height %
1	9.209	18852987	99.542	698922	99.432
2	10.384	86782	0.458	3994	0.568

(*S,E*)-(3-(but-3-en-1-yloxy)prop-1-ene-1,3-diyl)dibenzene **(5i)**

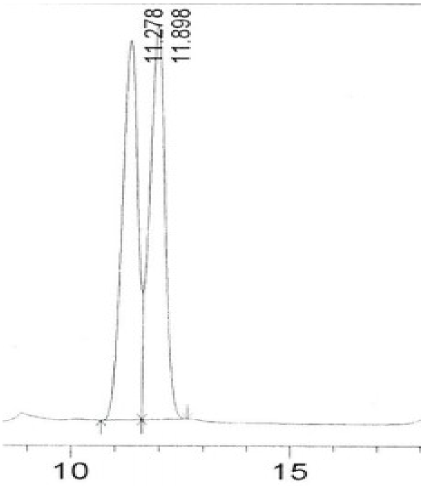

Peak	Ret. Time	Area	Area %	Height	Height %
1	11.278	1447127	48.887	59091	49.090
2	11.898	1513033	51.113	61282	50.910

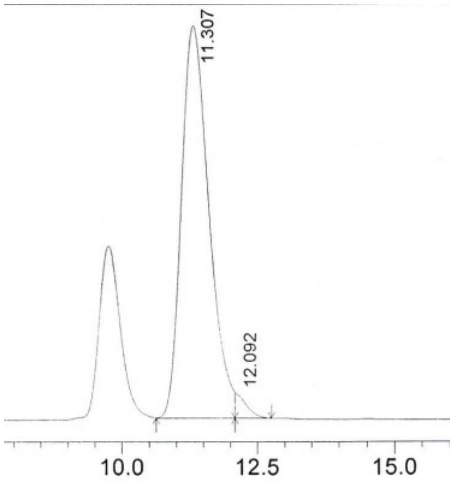

Peak	Ret. Time	Area	Area %	Height	Height %
1	11.307	8919784	97.533	250581	93.922
2	12.092	225589	2.467	16217	6.078

(S,E)-2-((1,3-diphenylallyl)oxy)-2,3-dihydro-1H-indene ***(5j)***

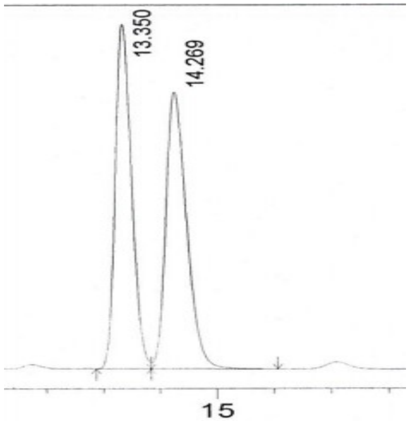

Peak	Ret. Time	Area	Area %	Height	Height %
1	13.350	5062897	49.764	252559	55.462
2	14.269	5110848	50.236	202815	44.538

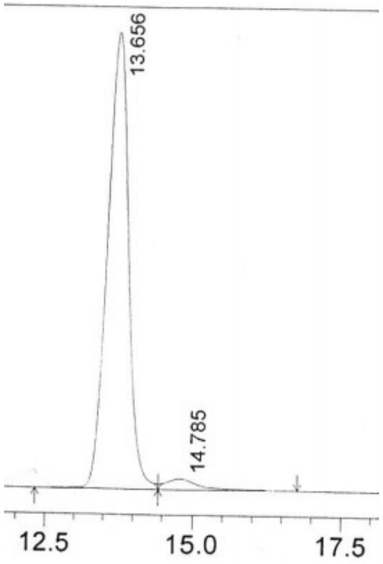

Peak	Ret. Time	Area	Area %	Height	Height %
1	13.656	11609781	96.515	469529	97.698
2	14.785	419259	3.485	11061	2.302
